# Impact of Temporal Resolution on Autocorrelative Features of Cerebral Physiology from Invasive and Non-Invasive Sensors in Acute Traumatic Neural Injury: Insights from the CAHR-TBI Cohort

**DOI:** 10.3390/s25092762

**Published:** 2025-04-27

**Authors:** Nuray Vakitbilir, Rahul Raj, Donald E. G. Griesdale, Mypinder Sekhon, Francis Bernard, Clare Gallagher, Eric P. Thelin, Logan Froese, Kevin Y. Stein, Andreas H. Kramer, Marcel J. H. Aries, Frederick A. Zeiler

**Affiliations:** 1 Department of Biomedical Engineering, Price Faculty of Engineering, University of Manitoba, Winnipeg, MB R3T 5V6, Canada; steink34@myumanitoba.ca (K.Y.S.); frederick.zeiler@umanitoba.ca (F.A.Z.); 2 Department of Neurosurgery, University of Helsinki, 00100 Helsinki, Finland; 3 Helsinki University Hospital, 00100 Helsinki, Finland; 4 Department of Anesthesiology, Pharmacology, and Therapeutics, University of British Columbia, Vancouver, BC V6T 1Z4, Canada; donald.griesdale@vch.ca; 5 Division of Critical Care, Department of Medicine, University of British Columbia, Vancouver, BC V6T 1Z4, Canada; mypindersekhon@gmail.com; 6 Section of Critical Care, Department of Medicine, University of Montreal, Montreal, QC H3T 1J4, Canada; bernard.francis@gmail.com; 7 Section of Neurosurgery, University of Calgary, Calgary, AB T2N 1N4, Canada; galclare@gmail.com; 8 Department of Clinical Neurosciences, University of Calgary, Calgary, AB T2N 1N4, Canada; andreas.kramer@albertahealthservices.ca; 9 Hotchkiss Brain Institute, University of Calgary, Calgary, AB T2N 1N4, Canada; 10 Department of Neurology, Karolinska University Hospital, 171 77 Stockholm, Sweden; eric.thelin@ki.se (E.P.T.); log.froese@gmail.com (L.F.); 11 Department of Clinical Neuroscience, Karolinska Institutet, 171 77 Stockholm, Sweden; 12 Department of Critical Care Medicine, University of Calgary, Calgary, AB T2N 1N4, Canada; 13 Department of Intensive Care, Maastricht University Medical Center+, 6229 Maastricht, The Netherlands; 14 School of Mental Health and Neurosciences, University Maastricht, 6211 Maastricht, The Netherlands; 15 Section of Neurosurgery, Department of Surgery, Rady Faculty of Health Sciences, University of Manitoba, Winnipeg, MB R3T 5V6, Canada; 16 Pan Am Clinic Foundation, Winnipeg, MB R3M 3E4, Canada

**Keywords:** high frequency signals, ARIMA, time-series analysis, cerebral physiology, stationarity analysis, multimodal signal analysis

## Abstract

Therapeutic management during the acute phase of traumatic brain injury (TBI) relies on continuous multimodal cerebral physiologic monitoring to detect and prevent secondary injury. These high-resolution data streams come from various invasive/non-invasive sensor technologies and challenge clinicians, as they are difficult to integrate into management algorithms and prognostic models. Data reduction techniques, like moving average filters, simplify data but may fail to address statistical autocorrelation and could introduce new properties, affecting model utility and interpretation. This study uses the CAnadian High-Resolution TBI (CAHR-TBI) dataset to examine the impact of temporal resolution changes (1 min to 24 h) on autoregressive integrated moving average (ARIMA) modeling for raw and derived cerebral physiologic signals. Stationarity tests indicated that the majority of the signals required first-order differencing to address persistent trends. A grid search identified optimal ARIMA parameters (*p*,*d*,*q*) for each signal and resolution. Subgroup analyses revealed population-specific differences in temporal structure, and small-scale forecasting using optimal parameters confirmed model adequacy. Variations in optimal structures across signals and patients highlight the importance of tailoring ARIMA models for precise interpretation and performance. Findings show that both raw and derived indices exhibit intrinsic ARIMA components regardless of resolution. Ignoring these features risks compromising the significance of models developed from such data. This underscores the need for careful resolution considerations in temporal modeling for TBI care.

## 1. Introduction

Traumatic brain injury (TBI), affecting 50 million people globally each year, involves both primary damage from the initial impact and secondary injuries, which develop over hours to days and worsen brain damage through processes like neuroinflammation [1]. These secondary injuries can lead to edema, increased intracranial pressure (ICP), and disruptions in cerebral blood flow (CBF), ultimately resulting in ischemia and tissue death [2,3,4]. Cerebral autoregulation, the brain’s ability to maintain stable CBF despite fluctuations in blood pressure, is critical in the management of TBI [5]. When impaired, it can lead to hypoperfusion or hyperperfusion, both of which exacerbate neuronal damage [6]. At the bedside, continuous monitoring of cerebral autoregulation relies on sensor-derived physiological signals, which serve as proxies for autoregulatory function [7,8]. Continuous multimodal monitoring of physiological signals from sensors, including arterial blood pressure (ABP), ICP, cerebral perfusion pressure (CPP), regional cerebral oxygen saturation (PbtO_2_), and brain tissue oxygenation (rSO_2_), is essential for capturing dynamic changes in cerebral physiology following TBI [9]. These advanced sensor systems enable real-time, high-frequency data collection, offering clinicians a better understanding of cerebral hemodynamics and autoregulation. Various indices derived from these sensor-based signals help assess cerebrovascular reactivity (CVR), including the pressure reactivity index (PRx; the correlation between ICP and mean arterial pressure (MAP)), the pulse amplitude index (PAx; the correlation between pulse amplitude of ICP (AMP) and MAP), a cerebral autoregulation index (RAC; the correlation [R] between AMP [A] and CPP [C]), and an index of cerebral compensatory reserve (RAP; the correlation [R] between AMP [A] and ICP [P]) [10,11].

Real-time sensor-driven monitoring of cerebral autoregulation offers valuable insights into brain perfusion and its response to changing physiological conditions, ultimately enhancing patient management and guiding personalized therapeutic strategies [6,12]. High-resolution physiological data from these sensors provide a continuous, detailed assessment of cerebrovascular function, allowing for precision medicine approaches in neurocritical care [13,14,15]. However, the large volume and high temporal resolution of sensor-derived data pose challenges for real-time clinical decision-making and integration into predictive analytics [15,16]. While data reduction techniques, such as non-overlapping moving average filters, help simplify the data into more manageable lower-resolution summary metrics, they may overlook important statistical autocorrelative features or introduce new ones, potentially affecting the interpretation and utility of any models that include such data streams [17,18,19]. Without accounting for such autocorrelative features in future models, the statistical significance comes into question given the priors of such techniques are often violated. This highlights the importance of understanding how different temporal resolutions impact the statistical characteristics of sensor-based physiological signals.

In this context, Sainbhi et al., 2024 [20], explored the effect of varying data resolution on the autocorrelative features of cerebral autoregulation signals, utilizing the Box–Jenkin’s time-series statistical structure, i.e., the autoregressive integrated moving average (ARIMA) model. Their findings underscore the impact of resolution on the statistical structure of these signals, a key consideration for clinical practice, where data resolution often needs to be balanced against real-time decision-making demands. Further studies have shown that ARIMA models are particularly well-suited for capturing autoregression and moving average components in physiological data, helping to identify trends and forecast outcomes in critical care settings [21,22]. However, these models can be sensitive to temporal resolution, as changes in resolution may obscure important autocorrelations and trends. Therefore, understanding how temporal resolution influences the ARIMA structure of physiological signals is crucial for improving clinical monitoring systems and prognostic models. This is particularly the case if such features violate the statistical priors of many modeling techniques in which these signals (raw or downsampled) are used. If not properly accounted for in such trajectory or prognostic models, these autocorrelative features impact both model significance and output accuracy.

The aim of the study is to characterize the underlying temporal dependencies, specifically autocorrelation, within high-resolution cerebral monitoring data. Building on the work of Sainbhi et al., 2024 [20], we leverage a multi-center dataset from the CAnadian High Resolution TBI (CAHR-TBI) Research Collaborative to investigate whether autocorrelative structure persists across different temporal resolutions and patient populations. The focus is on identifying patterns such as trends, autoregressive components, and moving average behavior, not to predict future values, but to understand how intrinsic temporal features manifest in signals like ICP, CPP, and derived indices such as PRx, PAx, and RAP.

To this end, the ARIMA model is used as a diagnostic framework rather than a forecasting tool. ARIMA is well-suited for this purpose as its structure (defined by parameters *p*, *d*, and *q*) explicitly quantifies autocorrelation (*p*), nonstationarity or trend (*d*), and short-term error correction (*q*) in time series data. By applying ARIMA models across varying temporal resolutions from 1 min to 24 h, we evaluate how these temporal features evolve with signal downsampling. Additionally, we examine whether the optimal ARIMA configurations (*p*,*d*,*q*) differ across patient subgroups defined by factors such as age, sex, hypoxia, hypotension, pupil reactivity, and Marshall CT classification. To further demonstrate the utility of the identified parameters, we also conducted small-scale forecasting on a subset of patients using their optimized ARIMA configurations. This allowed us to assess whether individualized model parameters, derived from retrospective modeling, could generalize to real-time prediction tasks with reasonable accuracy.

Rather than seeking to optimize predictive accuracy, this study emphasizes how autocorrelation and time-dependent structure in cerebral signals vary across individuals and monitoring resolutions. In doing so, it seeks to validate prior findings from single-center studies, examining whether key ARIMA features of cerebral physiologic data streams hold across multi-center datasets and addressing implications for earlier literature that attempted to temporally model cerebral physiology. These insights underscore the inherent trade-offs in sensor-based monitoring: while high-resolution sensor data enhance clinical insights and model accuracy, they also introduce significant computational and practical challenges; conversely, lower-resolution data, although more feasible for continuous long-term monitoring, may compromise the granularity of cerebral autoregulation assessments. Signal stationarity and optimal ARIMA model configurations are assessed at both the patient level and across individual sensor signals, spanning temporal resolutions from one minute to twenty-four hours.

## 2. Materials and Methods

### 2.1. Study Participants

This retrospective study leverages existing archived human subject data from the CAnadian High-Resolution TBI (CAHR-TBI) Research Collaborative [9]. Locally, at each CAHR-TBI participating center, high-frequency physiologic data were prospectively archived from patients aged 18 years and older with moderate-to-severe TBI admitted to the intensive care unit (ICU). The data was then retrospectively accessed and compiled to form the CAHR-TBI data set. Data collected spanned different time periods, depending on the center or origin; Foothills Medical Centre—University of Calgary (2011–2021), Health Sciences Centre Winnipeg—University of Manitoba (2019–2023), Maastricht University Medical Center—University of Maastricht (2017–2022), and Vancouver General Hospital—University of British Columbia (2014–2019).

Patients who satisfied all of the following requirements were added to the database: they had to be at least eighteen years old, diagnosed with moderate-to-severe TBI with a Glasgow Coma Scale (GCS) less than thirteen, admitted to one of the participating hospitals’ ICUs, have access to both invasive ICP and ABP monitoring, and aim for data collection to start within 24 h of presentation.

### 2.2. Data Collection

The Intensive Care Monitoring “Plus” (ICM+) data acquisition software (version 8.5, Cambridge Enterprise Ltd., Cambridge, UK, http://icmplus.neurosurg.cam.ac.uk (accessed on 10 October 2024)) was used to record all available high-frequency full wave-form physiologic data in digital time-series from the patients’ bedside ICU monitors. This was performed either by direct digital data transfer or analog-to-digital signal conversion (Data Translations, DT9804 or DT9826). ABP was measured continuously with a pressure transducer (Edwards, Irvine, CA, USA; Baxter Healthcare Corp. CardioVascular Group, Irvine, CA, USA) zeroed at the level of the tragus via a radial or femoral line.

ICP was monitored using an intraparenchymal pressure sensor probe (Codman ICP MicroSensor, Codman & Shurtlef Inc., Raynham, MA, USA; NEU-ROVENT-TEMP, RAUMEDIC, Helmbrechts, Germany; Camino ICP Monitor, Natus, Middleton, WI, USA) inserted into the patient’s frontal lobe or with an external venous drain (EVD; Medtronic, Minneapolis, MN, USA) in the lateral ventricle. PbtO_2_ (Licox Brain Tissue Oxygen Monitoring System; Integra LifeSciences Corp., Plainsboro, NJ, USA) monitoring was not present in all patients or all centers and was initiated at the discretion of the local treating team. Similarly, rSO_2_ was measured using near-infrared spectroscopy (NIRS) regional oximetry of the left and right frontal lobes (INVOS 5100C or 7100, Covidien-Medtronic, Minneapolis, MN, USA) where possible in a subgroup of patients, focusing only on viable rSO_2_ signal streams/channels (i.e., no underlying lesion, hematoma, or scalp issue). Channels were cross-referenced with lesion markups to ensure that only viable NIRS channels were analyzed.

### 2.3. Signal Processing

ICM+ software (version 8.5) was used for all post-acquisition signal processing. Removing signal artifacts and eliminating data segments with irregular waveforms or implausible values was performed by qualified personnel who were blinded to the study objectives and patient information. Based on radiographic data, a single rSO_2_ signal was chosen for patients undergoing NIRS monitoring in order to prevent interference from underlying hematomas or contusions.

Following the artifact removal, the basic amplitude of the ICP pulse waveform was calculated by Fourier analysis over consecutive 10-s data frames in order to compute AMP [23,24]. ICP and ABP were downsampled using a 10-s non-overlapping moving average filter to emphasize the slow-wave vasogenic oscillations linked to cerebral autoregulation. CPP was computed as follows: [CPP = MAP − ICP].

Moving Pearson correlation coefficients were determined using 30 consecutive 10-s mean windows updating continuously every minute to derive CVR indices that are PRx (the correlation between ICP and MAP), PAx (the correlation between AMP and MAP), RAC (the correlation between AMP and CPP), and RAP (the correlation between AMP and ICP) [10,11]. However, the overlapping nature of these windows introduces inherent autocorrelation. To address this in the ARIMA analysis, non-overlapping windows were utilized to create lower resolutions, mitigating autocorrelation and improving the interpretability of trend and variability measurements. Two NIRS-based CVR indicators were similarly generated, in accordance with recent literature; COx (the correlation between rSO_2_ and CPP) and COx-a (the correlation between Rso_2_ and ABP) [24,25]. Finally, all data was reduced to minute-by-minute resolution and exported as comma-separated value (CSV) format for every patient.

### 2.4. Data Analysis

#### 2.4.1. Temporal Resolution Reduction

All analyses were performed using custom Python scripts (v3.12.2, Python Software Foundation, Python Language Reference, https://www.python.org/ (accessed on 1 November 2023)) with pandas (v2.2.2) and statsmodels (v0.14.2) libraries. The temporal resolution of the patient data was progressively reduced in a non-overlapping manner from 1 min (the highest resolution) to 5 min and through multiple intermediate resolutions (10 min, 30 min, 60 min, 2 h, 3 h, 4 h, 5 h, 12 h) down to 24 h to allow for the application of various ARIMA models at different time resolutions. To address missing values, linear interpolation was applied when fewer than five consecutive data points were missing. If the missing segment exceeded five consecutive points, the entire segment was excluded from further analysis to maintain the integrity of time-series modeling.

#### 2.4.2. Evaluation of Stationarity

Stationarity, a property of time-series data where statistical characteristics such as mean and variance remain constant over time, is critical for reliable modeling and forecasting using methods like ARIMA. To evaluate stationarity, we applied the Augmented Dickey–Fuller (ADF) and Kwiatkowski–Phillips–Schmidt–Shin (KPSS) tests to each signal for every patient at each resolution [20]. The ADF test checks for the presence of a unit root to evaluate the stationarity of the series, while the KPSS test assesses whether the data is trend-stationary [26].

To determine whether differencing was necessary, we first ran both tests on the raw (undifferenced) signals. When both tests agreed that a signal was non-stationary (i.e., ADF failed to reject the null and KPSS rejected the null), we proceeded with first-order differencing. The differenced data were then retested with the same tests to confirm whether stationarity was achieved. If signals still exhibited signs of non-stationarity after first-order differencing, we did not manually impose a second-order differencing step. Instead, we allowed the ARIMA grid search procedure to evaluate models across both first-order (d = 1) and second-order (d = 2) differencing values. This approach enabled a data-driven determination of the appropriate differencing order, depending on the model fit criteria (AIC, BIC, LL) rather than a fixed stationarity assessment threshold.

All differencing steps were performed using a custom Python script, which also tracked whether stationarity was ultimately achieved. The decision to retain the first-order differenced series for ARIMA modeling was based on the majority outcome across ADF and KPSS tests, prioritizing statistical stationarity while avoiding over-differencing, which can obscure meaningful temporal dynamics. The full set of ADF and KPSS test results for both raw and differenced signals are summarized in Tables S1 and S2, respectively. First-order differencing was performed on each signal for every patient at each temporal resolution using a custom Python script. However, stationarity could not be reliably evaluated for certain patients due to insufficient data points at lower resolutions. In such cases, ‘Not available’ (NA) is reported. Similarly, NA is noted for signals that were not recorded for specific patients.

#### 2.4.3. ARIMA Analysis

The ARIMA model consists of three components: the autoregressive order (*p*), the differencing order (*d*), and the moving average order (*q*), collectively represented as (*p*,*d*,*q*). The p-order represents the number of previous signal values the model depends on, the d-order indicates the differencing steps needed for stationarity in the presence of trend or seasonality, and the q-order refers to the number of prior error terms, with an order of zero meaning the component is excluded from the model. The general form of the ARIMA model is shown in Equation (1), where Δ*^d^X_t_* represents the differenced series, *X_t_* is the value at time *t*, *c* is the constant term, *∅* and *θ* are the coefficients while *p* and *q* are the autoregressive and moving average orders, *e* are the lagged errors [27,28].

(1) $${∆}^{d}X_{t}=c+\sum_{i=1}^{p}\emptyset_{i}{X\;}_{t-i}+\sum_{j=1}^{q}\theta_{j}e_{q-j}+e_{t},$$

For the ARIMA analysis, “ARIMA” function from statsmodels package in Python was utilized. A grid search was conducted to determine the optimal ARIMA parameters (*p*,*d*,*q*) by exploring various combinations of *p* [1–10], *d* [0,1], and *q* [0–10], building on earlier findings from our research group [20].

Each combination was evaluated and stored in CSV files for each patient across different time resolutions using three model selection criteria: Akaike Information Criterion (AIC), Bayesian Information Criterion (BIC), and Log-Likelihood (LL). Lower AIC and BIC values and higher LL values indicate better model performance, balancing goodness-of-fit and model complexity. The best (*p*,*d*,*q*) configuration for each patient-signal pair was selected based on the lowest AIC value, as it offers a good trade-off between accuracy and overfitting. Tables S3 and S4 provide a single patient example of the grid search results from the ARIMA model for raw and derived physiological signals, respectively, with AIC, BIC, and LL values for each physiological variable.

We applied this procedure across various temporal resolutions (from 1 min to 24 h data) for both raw and derived physiological signals. The resulting model metrics were stored in CSV files for each patient and time resolution. A custom Python script was developed to automate the extraction of the optimal parameters from these files. This script scans each output directory, selects the best models based on the defined criteria, and aggregates them into summary CSV files for downstream analysis.

**Figure 1:**
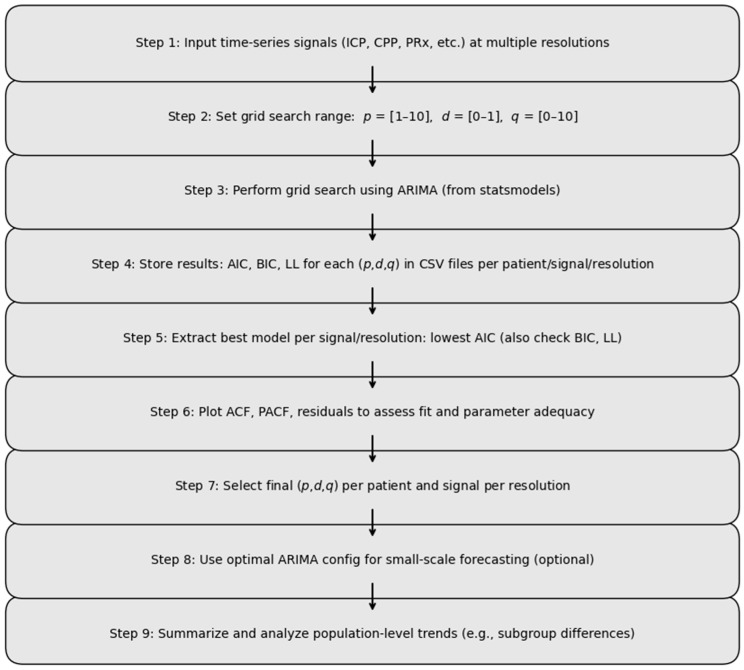
Visual summary of the ARIMA parameter optimization workflow. The diagram illustrates the sequential steps used to identify optimal ARIMA model parameters for physiological time series data. These include: (1) input and preprocessing of multi-resolution cerebral signals (e.g., ICP, CPP, PRx), (2) definition of grid search space for parameters (*p*,*d*,*q*), (3) grid search execution using the ARIMA function from the statsmodels package, (4) recording of model metrics (AIC, BIC, LL) for each configuration, (5) selection of optimal parameters based on performance criteria, and (6) diagnostic evaluation using ACF, PACF, and residual analysis. ACF, autocorrelation function; AIC, Akaike information criterion; ARIMA, autoregressive integrated moving average; BIC, Bayesian information criterion; CPP, cerebral perfusion pressure; ICP, intracranial pressure; LL, log-likelihood; PRx, pressure reactivity index; PACF, partial autocorrelation function.

Model diagnostic checks included plotting the residuals and evaluating the autocorrelation (ACF) and partial autocorrelation (PACF) of the residuals using the “plot_acf” and “plot_pacf” functions in statsmodels. Adequate model fit was confirmed by the presence of normally distributed, uncorrelated residuals and rapidly declining autocorrelations [29]. The overall process is listed in the flow diagram presented in Figure 1.

Additionally, the computational time required to execute the grid search for each signal, at each temporal resolution per patient, was meticulously recorded for performance analysis for each (*p*,*d*,*q*) combination and stored in the CSV files, with population median computational times for min-by-min resolution data provided in Table S5.

#### 2.4.4. Subgroup Analysis of ARIMA Model Performance

To investigate whether optimal ARIMA model selection metrics (AIC, BIC, and LL) differ meaningfully between patient subgroups, we conducted statistical tests on the best-fitting models for each signal and patient. Each subgroup was defined based on clinically relevant characteristics, including sex (male vs. female), age group (<40 vs. ≥40 years), presence of hypoxia (yes vs. no) and hypotension (yes vs. no), pupillary response (bilateral reactive, unilateral unreactive, bilateral unreactive), and Marshall CT classification (<5 vs. ≥5).

For subgroup comparisons with only two levels (e.g., sex, age, hypoxia, hypotension, and Marshall CT score), we assessed normality using the Shapiro–Wilk test. If both subgroups exhibited normal distributions, we applied a two-sample t-test to compare the mean values of AIC, BIC, and LL. If normality was not met in either group, a non-parametric Mann–Whitney U test was used instead. For the pupil response, which includes three categories, we used a one-way ANOVA if all subgroups satisfied normality assumptions. If normality was violated in one or more groups, a Kruskal–Wallis test was applied to determine if there were statistically significant differences in model metrics among the three pupillary response types.

A *p*-value threshold of 0.05 was used across all tests to assess statistical significance. A *p*-value below the threshold indicates a statistically significant difference in the model performance metric across the subgroups, suggesting that ARIMA model performance may vary meaningfully with patient characteristics.

#### 2.4.5. Forecasting with Optimized ARIMA Parameters—Pilot Analysis

To demonstrate the practical applicability and feasibility of implementing individualized forecasting strategies in clinical settings through our ARIMA model optimization framework, we conducted a forecasting analysis using ICP time-series data sampled at a 1-min resolution. For this demonstration, we selected the optimal ARIMA parameters (*p*,*d*,*q*) identified for each patient-signal pair based on the AIC. We chose AIC for the selection of optimum (*p*,*d*,*q*) parameters as it balances model goodness-of-fit with model complexity, penalizing overfitting more effectively than LL and often favoring simpler models compared to the BIC. These models were applied to ICP forecasting in 13 patients who had the most complete and highest-quality data, with their recordings being free from significant missing data chunks or artifacts that could disrupt the continuity and reliability of the time series, ensuring consistent and uninterrupted signal flow. All data were recorded at the same site.

We applied the AIC-selected ARIMA parameters to fit models on short ICP data segments and generated one-step-ahead forecasts. This exercise serves to validate whether the optimized parameters identified through retrospective modeling generalize to future prediction tasks. Forecast performance was quantitatively assessed using Pearson correlation coefficients to evaluate linear agreement between predicted and observed values and Bland–Altman analysis to estimate systematic bias and limits of agreement, providing insight into the accuracy and consistency of the individualized forecasting models.

## 3. Results

### 3.1. Patient Population

This study utilized 376 moderate-to-severe TBI patient records from the CAHR-TBI research collaborative, 123 of which were from the University of Calgary, 125 from the University of Manitoba, 51 from the University of Maastricht, and 77 from the University of British Columbia. There were 288 males (78%), with a median age of 38 years (IQR = 24–55). All patients underwent invasive monitoring of their ICP and ABP, but only 146 (40%) had rSO_2_ monitoring, and 116 (31%) had PbtO_2_ monitoring; general information on the demographics is provided in Table 1. The signals that are examined in the research, if they exist for the specific patient, are as follows: MAP, ICP, CPP, PRx, PAx, RAC, RAP, cerebral oximetry index of left and right hemispheres (COx_L and COx_R, respectively), cerebral oximetry index with ABP of left and right hemispheres (COx-a_L and COx-a_R, respectively), rSO_2__L, rSO_2__R, and PbtO_2_.

### 3.2. Results of Evaluation of Stationarity

The assessment of the time series stationarity on the original data revealed that, although the ADF test classified the majority of the physiologic signals as stationary, the KPSS test identified them as non-stationary, regardless of the signal type. However, as the temporal resolution decreased, the ADF test also began to show non-stationarity, suggesting that applying ARIMA models, particularly when deriving median optimal parameters, would likely be less effective (see Table S6). Two potential reasons for this are: (1) a lack of data points at lower temporal resolutions, and (2) that taking larger averages of the data introduces more trend, which begins to affect the ARIMA orders. Table 2 presents KPSS test results on the original (non-differenced) data for the whole study cohort.

After first-order differencing across all resolutions, both the ADF and KPSS tests indicated that the signals were stationary. The shift towards non-stationarity observed in the tests reflects limitations or changes in how the signal’s aggregated behavior is interpreted at different resolutions. However, the ADF test showed a shift towards non-stationarity as the resolution decreased, as shown in Table S7, while the KPSS test revealed a sharp shift to non-stationarity only at the 1-day resolution, as presented in Table 3, likely due to a lack of data points at lower resolutions.

### 3.3. ARIMA Analysis Results

The grid search with the ARIMA model, applied to first-order differenced data, demonstrated variations in the optimal combinations of autocorrelative features (*p*,*d*,*q*) for each signal for each patient at every temporal resolution, based on the lowest AIC, lowest BIC, or highest LL values. The overall grid search took approximately 102 days to complete, covering all temporal resolutions, model parameters, and signals. For the selection of optimal ARIMA models, AIC values are prioritized over BIC and LL values due to the behavior observed in the latter two criteria. The LL values predominantly suggested a high *p*-order (approximately 10), which tends to overfit the data by capturing even minor fluctuations. On the other hand, the BIC values, which heavily penalize model complexity, consistently pointed to a *p*-order of 1, indicating a model that might be too simplistic and overlook essential autoregressive structures in the data. No specific trends in the (*p*,*d*,*q*) values were observed across all patient data at any temporal resolution. However, significant variation in the model parameters was noted not only between different variables but also within the same variable across different temporal resolutions, highlighting the sensitivity of the (*p*,*d*,*q*) values to resolution changes. Additionally, a few variables in some patient data had a *d*-order of 2, indicating the need for second-order differencing.

Table 4 provides a comprehensive summary of the median ARIMA model parameters optimized for each combination of physiological signals and temporal resolutions across the population. In general, the p-order ranged between 2 and 5 across various temporal resolutions, while the q-order showed a decreasing trend from 5 to lower values, such as 1 and 0, at lower resolutions. Additionally, the table highlights that at lower resolutions, certain signals required second-order differencing, with the d-order increasing to 2.

**Figure 2:**
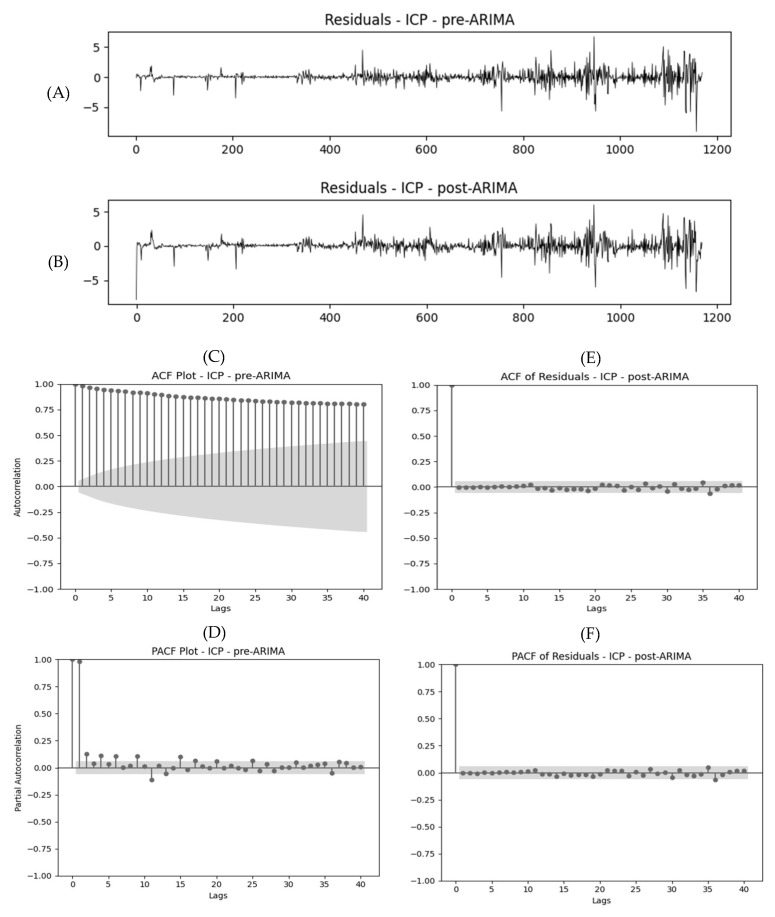
Residuals, ACF, and PACF plots for the minutely ICP signal—pre- and post-ARIMA. (**A**,**C**,**D**) The original ICP time series data, (**B**,**E**,**F**) along with its ACF and PACF plots. These plots reveal substantial autocorrelation, with numerous lags exhibiting statistically significant correlations, indicating strong temporal dependencies within the raw signal. The shaded area around zero represents the 95% confidence interval; spikes extending beyond this range suggest that the corresponding lags are statistically significant. (**E**) ACF and (**F**) PACF plots of the ICP signal after fitting and differencing with the ARIMA model, using the median optimal parameters derived from our grid search across the population—specifically, an autoregressive (*p*) order of 10, a differencing (*d*) order of 1, and a moving average (*q*) order of 7. Post-modeling, the ACF and PACF plots show a pronounced reduction in significant lags, suggesting that much of the temporal structure in the original signal has been effectively accounted for by the ARIMA model. The residuals appear randomly distributed with no obvious patterns or remaining autocorrelation, supporting the adequacy of the fitted model. ACF, autocorrelation function; ARIMA, autoregressive integrated moving average; ICP, intracranial pressure; PACF, partial autocorrelation function.

Figure 2 illustrates the residuals, ACF, and PACF plots, respectively, of min-by-min ICP signals of a patient before and after applying the optimal ARIMA model with parameters of (10,2,7). The ACF and PACF plots of the ICP signal, before applying the ARIMA model, exhibit significant autocorrelation, as indicated by lags exceeding the confidence intervals. However, following the application of the ARIMA model with optimal parameters, this autocorrelation is significantly reduced, as shown in the post-ARIMA graphs in Figure 1.

### 3.4. Subgroup Analysis

To explore whether ARIMA model selection metrics differed significantly across patient subgroups, we performed statistical comparisons of AIC, BIC, and LL values for each signal. Subgroups were defined based on clinically relevant variables such as sex, age group, presence of hypoxia and hypotension, pupillary response, and Marshall CT score. These analyses aimed to identify whether model performance varied systematically with specific patient characteristics, potentially reflecting underlying physiological or pathophysiological differences. Below, we summarize the key findings across the subgroup comparisons.

The distributions of the selected (*p*,*d*,*q*) parameters across subgroups were predominantly non-normal, as determined by the Shapiro–Wilk test. Consequently, Mann–Whitney U and Kruskal–Wallis tests were primarily used across all resolutions, except for certain subgroups where BIC distributions met normality assumptions and required a *t*-test.

At the 1-min temporal resolution, no statistically significant differences were found in the distribution of optimal ARIMA parameters (*p*,*d*,*q*) based on hypoxia or hypotension status across any signal, as shown in Table 5. Additionally, sex and age group effects were largely non-significant for most signals, except for PAx, RAC, and RAP, which remained significant up to the 60-min resolution. Among these, RAC showed the most significant *p*-value of 0.001. A p-value below the 0.05 significance threshold indicates that the autocorrelation characteristics of the signal differ meaningfully across the compared subgroups, suggesting possible physiological divergence. The results suggest that the selection of ARIMA parameters for all signals, except CPP and PbtO_2_, varies meaningfully with Marshall CT classification across all temporal resolutions, potentially reflecting distinct autoregulatory dynamics in patients with more severe brain injury. Similarly, subgroup differences in parameter distributions for PRx, PAx, and RAP based on pupil status and age may indicate altered autoregulatory behavior in specific populations.

### 3.5. Forecasting Results for Optimum ARIMA Parameters—Pilot Analysis

To assess the practical viability of individualized ARIMA modeling for real-time prediction, we conducted a forecasting analysis using 1-min resolution ICP time-series data from 13 patients. Each model was fitted using the previously selected optimal ARIMA parameters (*p*,*d*,*q*) based on AIC, and one-step-ahead forecasts were generated. Forecast performance was evaluated quantitatively through Pearson correlation analysis and Bland–Altman agreement metrics.

Pearson correlation coefficients between predicted and observed values ranged from weakly negative to moderately positive (−0.004–0.38), with most clustering between 0.1 and 0.35, indicating modest predictive agreement, as shown in Table 6. Bland–Altman analysis showed that mean differences between forecasts and actual ICP values were close to zero in most patients (ranging from −0.29 to 0.02), suggesting low systematic bias. Limits of agreement (LoA) varied across patients, with typical spreads of ±1 to ±3 mmHg.

**Figure 3:**
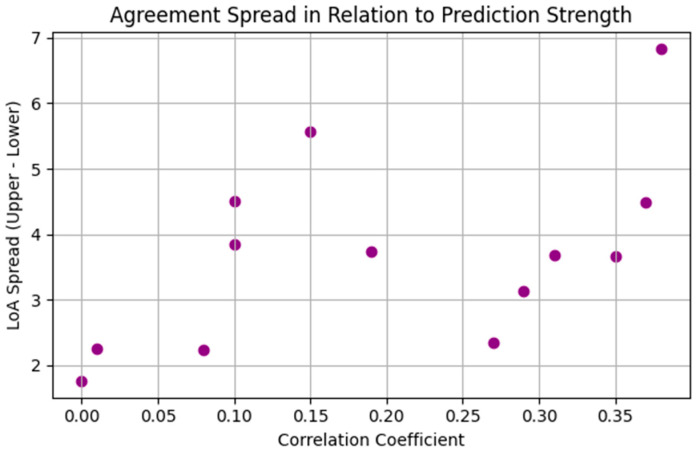
Scatter plot showing the relationship between Pearson correlation coefficients and Bland–Altman agreement spread (upper—lower limits of agreement) across 13 patients for one-step-ahead ICP forecasts using individualized ARIMA models. LoA, limits of agreement.

To further explore the relationship between predictive performance and agreement consistency across patients, Figure 3 presents a scatter plot comparing correlation coefficients with the spread between Bland–Altman LoA. This analysis revealed no strong or consistent relationship between the strength of correlation and the agreement spread. Notably, some patients with relatively high correlation values, e.g., >0.35, still exhibited wide LoA spreads (>6 mmHg), suggesting substantial variability in prediction error despite reasonable alignment with observed trends. On the other hand, several patients showed narrow LoA spreads (as low as ±1.5 mmHg) despite weak or near-zero correlation, indicating that while the forecasts lacked strong trend-following ability, they remained close to the mean observed values with minimal systematic bias. This pattern may reflect scenarios where ICP values remained relatively stable, leading the model to perform adequately in terms of magnitude but poorly in capturing fluctuations.

While the primary objective of this study was to characterize intrinsic temporal dependencies, such as trends, autoregressive behavior, and short-term memory, in cerebral physiologic signals, the small-scale forecasting analysis highlights an important secondary insight: individualized ARIMA parameters, optimized through retrospective modeling, may support more accurate and patient-specific prediction strategies, particularly in contexts where understanding temporal structure enhances clinical utility.

## 4. Discussion

This study set out to examine how patterns over time, specifically autocorrelation, are embedded in high-resolution cerebral monitoring data. Unlike studies that use modeling to predict future values, our goal was not to forecast, but to understand how brain signals behave over time and whether this behavior changes depending on the level of data detail (temporal resolution) or patient characteristics. To do this, we applied ARIMA model, not as a forecasting tool, but as a way to measure how much a signal depends on its own past values. By fitting ARIMA models to signals like ICP, CPP, and derived indices such as PRx, PAx, and RAP, we explored how these time-based features shift when data is sampled at different intervals ranging from every minute to once every 24 h for different patients. We also tested whether different patient groups (based on age, sex, brain injury severity, pupil response, hypoxia, or hypotension) showed systematic differences in these temporal patterns.

In addition, the study aimed to validate prior single-center findings on the autoregressive properties of sensor-based cerebral physiologic data streams and to explore their implications for past research that attempted to model cerebral physiology over time using sensor data. ADF and KPSS tests were employed to assess the stationarity of each sensor-derived physiological signal at different temporal resolutions. For the ARIMA model, a grid search was carried out for various model parameters as *p* [1–10], *d* [1,2], and *q* [0–10] for all signals, with performance evaluated using AIC, BIC, and LL values. For the given temporal resolution and physiological signal, the optimal ARIMA model was identified from all of the patients considering the lowest AIC and BIC and the highest LL values.

The assessment of time series stationarity on the original 1-min resolution data revealed contrasting outcomes between the ADF and KPSS tests. While the ADF test classified the majority of physiological signals as stationary, the KPSS test identified them as non-stationary, suggesting the presence of trend stationarity. This discrepancy highlights that many signals may follow a deterministic trend, which is not captured by the ADF test but observed by the KPSS test. As the temporal resolution decreased, the ADF test also began to indicate non-stationarity, implying that lower resolution data introduces more complex patterns that make stationarity difficult to maintain. This underscores the importance of considering both tests together to fully understand the stationarity characteristics of physiological signals. The combined use of ADF and KPSS tests provides a more nuanced view of stationarity, ensuring that both stochastic and deterministic trends are accounted for. These findings suggest that applying ARIMA models, especially when determining median optimal parameters, becomes more challenging and potentially less effective with lower resolutions.

After applying first-order differencing across all resolutions, both the ADF and KPSS tests confirmed that the signals were stationary, indicating that differencing effectively removed trends and ensured stationarity. However, for some patients at lower resolutions, the ADF test returned to displaying non-stationarity in various signals, whereas the KPSS test indicated only very slight returns to non-stationarity indicating that progressive averaging introduces patterns, even with first-order differencing, and fails to resolve non-stationarity. These results carry important implications for earlier studies, particularly studies that have relied on temporally averaged data without sufficiently accounting for the reintroduction of trends and non-stationarity. It underscores the risk that reduced averaging might conceal meaningful signal dynamics and lead to incorrect conclusions about underlying physiological processes. For future work, this highlights the need to carefully consider the impact of temporal resolution when analyzing time series data, especially in studies using high-frequency physiological signals. While differencing may initially address trends, it is essential to account for the fact that averaging at lower resolutions can reintroduce non-stationary behaviors. This suggests that more robust preprocessing techniques or alternative models that account for non-stationarity at varying resolutions should be explored to ensure accurate signal interpretation.

The grid search with the ARIMA model, applied to first-order differenced data, revealed variations in the optimal combinations of autocorrelative features (*p*,*d*,*q*) for each signal across patients and at every temporal resolution. These variations were determined based on the lowest AIC, BIC, or the highest LL values. AIC was prioritized over BIC and LL for selecting ARIMA models, as LL suggested high p-orders that risked overfitting, while BIC favored overly simplistic models with low p-orders that might overlook significant autoregressive features. AIC values allow for a balanced model fit by effectively managing complexity, which helps avoid both overfitting and underfitting while reflecting the data’s inherent patterns more realistically. Additionally, no specific trends in the (*p*,*d*,*q*) values emerged consistently across all patient data at any temporal resolution. However, a substantial difference in these parameters was observed, not only between different variables but also within the same variable across different resolutions. This signifies the sensitivity of ARIMA model parameters to changes in temporal resolution. Notably, a few physiologic signals required a *d*-order of 2, signifying the need for second-order differencing, further emphasizing the complexity and variability of physiologic signals across different temporal scales.

The model adequacy with the parameters chosen with AIC was also demonstrated by comparing the plots of residuals, ACF, and PACF before and after applying the ARIMA model. Overall, the findings indicate that autocorrelation persists in cerebral physiologic time-series data, even at lower temporal resolutions such as the hourly and daily mean values, necessitating models to consider autoregressive, integrative, and moving average orders for interpretable outputs. This raises issues with the findings of previous studies that reduced temporal resolution and modeled physiology without accounting for persistent autocorrelation, as it likely led to biased estimates, reduced model accuracy, and weakened the statistical significance of findings. Failure to address these issues may have resulted in oversimplified interpretations of physiological patterns. For future work, this highlights the importance of using models that explicitly account for temporal dependencies, even at lower resolutions, to improve predictive accuracy and produce more robust conclusions about cerebral physiology.

In addition to exploring temporal dependencies across resolutions, we investigated whether the optimal ARIMA model parameters (*p*,*d*,*q*) varied systematically across different clinical subgroups. This analysis revealed that certain patient characteristics were indeed associated with differences in time-series structure. Specifically, Marshall CT classification consistently influenced ARIMA configurations for nearly all signals (except CPP and PbtO_2_) across all resolutions, suggesting that patients with more severe brain injury may exhibit fundamentally different temporal dynamics in their cerebral physiology. Furthermore, PAx, RAP, and RAC showed statistically significant subgroup differences based on age and sex, with these effects persisting even at lower temporal resolutions down to 60-min resolutions. On the other hand, no significant differences were observed for hypoxia and hypotension status across any signal at any resolution, suggesting that these acute systemic factors may not substantially alter the underlying autoregressive behavior of cerebral signals over time. Additionally, the majority of subgroups were non-normal and were tested with non-parametric methods or either Mann–Whitney U or Kruskal–Wallis tests.

To evaluate the practical relevance of the ARIMA model configurations derived through retrospective modeling, we conducted a small-scale forecasting exercise using ICP data at 1-min resolution. One-step-ahead forecasts were generated for 13 patients using their individualized optimal ARIMA parameters. Correlation coefficients between forecasted and observed values ranged from −0.004 to 0.38, with most patients clustering between 0.1 and 0.35, indicating modest predictive agreement. Bland–Altman analyses showed low systematic bias, with mean forecast errors near zero in the majority of patients and typical limits of agreement spanning ±1 to ±3 mmHg. These results suggest that individualized ARIMA configurations, while modest in predictive strength, maintain consistent agreement with actual values and offer clinically relevant short-term forecasts. Future work is needed to explore how data splitting strategies, such as time-series split and blocked time-series split, and model memory settings, influence predictive performance. Additionally, extending ARIMA and other time-series models to support both point and interval predictions will be important for robust forecasting of cerebral physiological signals.

On a different note, it is important to acknowledge the limitation posed by spatial heterogeneity in cerebral perfusion, particularly in the context of TBI, where regional blood flow may vary significantly due to focal injuries, edema, or disrupted autoregulation. Our study utilized a single-source measurement of CPP, typically derived from ABP and ICP, which reflects a global estimate of perfusion rather than region-specific dynamics. However, cerebral perfusion is inherently anisotropic and spatially heterogeneous, which is a critical characteristic that influences physiologic responses and treatment efficacy. Recent modeling studies [30] underscore the importance of accounting for distinct regions of high, moderate, low, or absent perfusion, emphasizing the need for advanced techniques to capture and characterize these spatial variations in the injured brain. This spatial variation can profoundly influence tissue viability and response to therapeutic interventions. Although current invasive monitoring technologies in clinical TBI settings limit us to a single probe location, future work incorporating multimodal imaging or multi-site sensor data could help identify whether autoregressive structures and temporal dependencies observed in CPP or related signals vary across spatial domains. Such efforts would enhance our understanding of the interplay between localized pathophysiological processes and global cerebral dynamics and could improve the personalization and efficacy of treatment strategies based on physiological modeling.

For live-time calculations at the bedside, the computational cost of running a grid search for ARIMA parameters can vary significantly depending on the temporal resolution of the signals being modeled. At higher temporal resolutions, the cost of computation can range from several seconds to a minute for each grid search, depending on the number of signals and the range of parameters being evaluated. Given the high frequency of data points in these cases, the total computational time can extend from a day to several days when processing multiple signals. However, as the temporal resolution decreases, the computational load is reduced, and the entire process could be completed within several hours. This reduction occurs as fewer data points need to be analyzed at lower resolutions, making the grid search for optimal ARIMA parameters less computationally intensive. To make this approach feasible at the bedside, careful consideration of the parameters to be optimized and the signals to be processed is essential, particularly for very high temporal resolutions where the computational demands are much greater.

## 5. Limitations

This study was conducted as an exploratory investigation, with the primary aim of evaluating how different temporal resolutions of cerebral physiological signals influence autocorrelative features, such as trends, autoregressive terms, and moving average orders. The scope of this study was limited to understanding these dynamics, and as such, it did not involve the validation of the selected ARIMA models for forecasting future data. While the ARIMA models developed here were based on the data at hand, and while they offer valuable insights into the time-series structure, they may not be directly applicable to newly acquired datasets without further validation. This study also did not involve running comparative models or implementing cross-validation approaches, as the primary objective was to perform an external validation of previous findings rather than optimize forecasting performance. Including such analyses would have extended the scope and complexity of the manuscript, detracting from its central aim.

Additionally, this study did not include a comprehensive assessment to identify potentially simpler models with lower p-orders and q-orders than the median optimal model found during the analysis. The goal was to determine if there existed a specific temporal resolution where the autocorrelative structure could be ignored, rather than to focus on model optimization. Furthermore, this study does not analyze how different severities of traumatic brain injury (TBI) might influence the time-series characteristics of cerebral physiological signals. While our primary focus was on investigating the effects of temporal resolution on autocorrelative features, we acknowledge that TBI severity could introduce additional variability and influence these characteristics. Future work should incorporate TBI severity as a factor, which could enhance the clinical relevance of the findings.

Therefore, future research will be necessary to explore these unresolved aspects, including the validation of the ARIMA models on larger and more diverse datasets, as well as the refinement of model selection criteria to potentially identify more efficient models. These follow-up studies would be essential to confirm the generalizability of the findings and to ensure that the models can be effectively applied to future data for predictive purposes.

## 6. Conclusions

This study explored how different sensor-derived data resolutions impacted the application of ARIMA modeling to cerebral physiological signals, emphasizing the role of multimodal sensor systems in capturing high-frequency neurophysiological data. Additionally, it aimed to validate previous single-center findings regarding the autoregressive properties of these sensor-based data streams and assess their implications for earlier efforts to develop time-series models of cerebral physiology. Stationarity tests (ADF, KPSS) showed discrepancies, with ADF indicating most signals were stationary and KPSS suggesting non-stationarity. As resolution decreased, non-stationarity became more pronounced, complicating ARIMA modeling. First-order differencing addressed this, but many signals still showed non-stationarity at lower resolutions.

A systematic grid search for ARIMA parameters (*p*,*d*,*q*) revealed significant variability across patients and signal types, reinforcing the need for patient-specific modeling approaches in sensor-based cerebral monitoring. AIC provided the best balance between model complexity and fit, indicating its utility as a selection criterion for optimizing ARIMA models in physiological data analysis. The findings highlighted both the sensitivity of ARIMA models to resolution changes and the persistence of autocorrelations in sensor-acquired data, even at lower resolutions.

The findings highlighted the importance of applying at least first-order differencing to both raw and derived sensor-based cerebral physiologic signals. Furthermore, our findings help clarify that even as data is downsampled to lower resolutions, time-dependent structure remains in the signals. This has important implications for studies analyzing cerebral physiology: regardless of the model or method used, underlying autocorrelation persists, and failing to account for it could affect clinical interpretations. Thus, our study emphasizes the necessity of accounting for autoregressive, integrative, and moving average components when developing robust time-series models for cerebral physiology. Additionally, the observation that ARIMA model parameters vary across patient subgroups suggests that the temporal dynamics of cerebral physiology are not one-size-fits-all, and may reflect biologically meaningful differences in autoregulatory behavior. These insights contribute to the refinement of sensor-driven monitoring systems, ensuring accurate trend detection and predictive modeling in neurocritical care.

## Figures and Tables

**Table 1:** Demographic data.

Variable	Median (Interquartile Range) or Number (%)
Number of patients	376
Duration of recording (minutes)	5904 (3204–11,160)
Age (years)	38 (24–55)
Male sex	288 (78%)
GCS	6 (4–7)
GCS-motor	4 (1–5)
**Pupils**	
Bilateral reactive	185 (50.11%)
Bilateral unreactive	111 (30.01%)
Unilateral unreactive	63 (17.07%)
**Marshall CT Classification**	
V + VI	112 (30.35%)
IV	85 (23.03%)
III	43 (11.65%)
II	100 (27.10%)
Hypoxic episode	61 (26.50%)
Hypotensive episode	35 (15.08%)
Mean MAP (mmHg)	86.9 (81.1–92.9)
Mean ICP (mmHg)	11.9 (8.17–14.8)

CT, computerized tomography; GCS, Glasgow Coma Score; ICP, intracranial pressure; MAP, mean arterial pressure.

**Table 2:** Percentage comparison of stationarity and non-stationarity based on the KPSS test on the original (non-differenced) data.

Temporal Resolution		1-min	5-min	10-min	30-min	1-h	2-h	3-h	4-h	5-h	6-h	12-h	1-day
MAP	Stationarity	14.5	22.3	29.7	44.8	53.5	62.1	67.6	70.1	67.8	72.3	78.7	88.9
	Non-stationary	85.2	77.4	70.0	55.0	46.2	37.6	32.1	29.6	31.9	27.4	20.9	10.6
	NA	0.3	0.3	0.3	0.3	0.3	0.3	0.3	0.3	0.3	0.3	0.4	0.5
ICP	Stationarity	10.3	24.0	30.8	44.5	50.6	58.9	63.5	67.1	71.1	72.3	80.1	87.5
	Non-stationary	89.7	76.0	69.2	55.5	49.4	41.1	36.5	32.9	28.9	27.7	19.9	12.5
	NA	0.0	0.0	0.0	0.0	0.0	0.0	0.0	0.0	0.0	0.0	0.0	0.0
CPP	Stationarity	15.9	30.7	38.9	49.9	55.5	64.7	68.2	71.0	71.1	72.6	79.8	87.0
	Non-stationary	83.8	69.0	60.8	49.9	44.2	35.0	31.5	28.7	28.6	27.1	19.9	12.5
	NA	0.3	0.3	0.3	0.3	0.3	0.3	0.3	0.3	0.3	0.3	0.4	0.5
PRx	Stationarity	18.1	28.2	33.6	46.5	56.1	62.4	66.8	72.5	73.9	77.3	82.6	88.4
	Non-stationary	81.6	71.5	66.1	53.3	43.6	37.3	32.9	27.2	25.8	22.4	17.0	11.1
	NA	0.3	0.3	0.3	0.3	0.3	0.3	0.3	0.3	0.3	0.3	0.4	0.5
PAx	Stationarity	18.1	25.1	32.2	45.6	56.4	65.9	70.0	72.2	72.9	77.9	83.3	90.7
	Non-stationary	81.6	74.6	67.5	54.1	43.4	33.8	29.7	27.5	26.7	21.8	16.3	8.8
	NA	0.3	0.3	0.3	0.3	0.3	0.3	0.3	0.3	0.3	0.3	0.4	0.5
RAC	Stationarity	15.6	23.7	31.1	41.1	50.0	60.9	65.6	69.2	72.0	75.1	81.9	89.4
	Non-stationary	84.1	76.0	68.6	58.6	49.7	38.8	34.1	30.5	27.7	24.6	17.7	10.2
	NA	0.3	0.3	0.3	0.3	0.3	0.3	0.3	0.3	0.3	0.3	0.4	0.5
RAP	Stationarity	18.9	28.8	34.5	45.6	53.5	62.7	67.1	71.3	72.6	75.4	80.9	88.9
	Non-stationary	81.1	71.2	65.5	54.4	46.5	37.3	32.9	28.7	27.4	24.6	19.1	11.1
	NA	0.0	0.0	0.0	0.0	0.0	0.0	0.0	0.0	0.0	0.0	0.0	0.0
COx_L	Stationarity	13.1	15.6	17.6	21.5	24.3	25.7	25.9	27.2	28.0	28.0	27.0	27.8
	Non-stationary	24.0	21.5	19.3	14.7	11.3	9.9	9.4	8.1	7.6	6.9	4.6	2.3
	NA	63.0	62.8	63.0	63.7	64.5	64.4	64.7	64.7	64.4	65.1	68.4	69.9
COx_R	Stationarity	13.6	17.3	20.2	22.7	22.5	26.2	27.4	27.8	28.9	27.7	28.4	26.4
	Non-stationary	23.4	19.8	16.8	13.6	13.0	9.0	7.6	7.2	6.4	6.9	3.5	3.7
	NA	63.0	62.8	63.0	63.7	64.5	64.7	65.0	65.0	64.7	65.4	68.1	69.9
COx-a_L	Stationarity	14.5	17.0	17.1	21.2	21.4	23.0	25.3	25.7	25.2	26.2	27.0	26.4
	Non-stationary	21.4	19.0	18.8	13.9	13.0	11.4	8.8	8.4	9.1	7.5	3.5	3.2
	NA	64.1	64.0	64.1	64.9	65.6	65.6	65.9	65.9	65.7	66.4	69.5	70.4
COx-a_R	Stationarity	17.0	20.1	21.3	21.8	22.0	24.5	26.2	27.2	26.7	26.2	26.6	27.3
	Non-stationary	18.9	15.9	14.6	13.3	12.4	9.6	7.6	6.6	7.3	7.2	4.3	2.3
	NA	64.1	64.0	64.1	64.9	65.6	65.9	66.2	66.2	66.0	66.7	69.1	70.4
rSO_2__L	Stationarity	2.8	7.8	10.4	14.4	15.3	16.9	20.3	21.6	21.3	23.4	23.0	23.6
	Non-stationary	34.5	29.6	26.9	22.1	20.5	18.7	15.0	13.8	14.3	11.5	8.5	6.5
	NA	62.7	62.6	62.7	63.5	64.2	64.4	64.7	64.7	64.4	65.1	68.4	69.9
rSO_2__R	Stationarity	4.5	10.1	11.8	15.0	17.6	19.5	22.4	22.5	22.8	23.7	25.2	23.6
	Non-stationary	32.6	27.1	25.2	21.2	17.9	15.7	12.6	12.6	12.5	10.9	6.7	6.5
	NA	63.0	62.8	63.0	63.7	64.5	64.7	65.0	65.0	64.7	65.4	68.1	69.9
PbtO_2_	Stationarity	4.7	10.1	12.0	14.4	15.3	18.4	20.6	21.3	21.6	22.1	24.1	27.8
	Non-stationary	25.1	19.6	17.6	15.0	13.9	11.1	8.8	7.2	6.7	6.5	5.0	3.7
	NA	70.2	70.4	70.3	70.5	70.8	70.6	70.6	71.6	71.7	71.3	70.9	68.5

COx_L, cerebral oximetry index of left hemisphere; COx_R, cerebral oximetry index of right hemisphere; COx-a_L, COx with ABP of left hemisphere; COx-a_R, COx with ABP of the right hemisphere; CPP, cerebral perfusion pressure; ICP, intracranial pressure; KPSS, Kwiatkowski–Phillips–Schmidt–Shin; MAP, mean arterial pressure; NA, not applicable; PAx, pulse amplitude index; PbtO_2_, cerebral oxygen saturation; PRx, pressure reactivity index; RAC, a cerebral autoregulation index; RAP, index of cerebral compensatory reserve; rSO_2__L, brain tissue oxygenation of left hemisphere; rSO_2__R, brain tissue oxygenation of the right hemisphere.

**Table 3:** Percentage comparison of stationarity and non-stationarity based on KPSS test on the first-order differenced data.

Temporal Resolution		1-min	5-min	10-min	30-min	1-h	2-h	3-h	4-h	5-h	6-h	12-h	1-day
MAP	Stationarity	97.1	98.4	97.6	95.5	93.6	88.0	86.4	84.0	82.4	81.4	68.1	44.4
	Non-stationary	2.7	1.3	1.9	4.0	4.8	9.3	9.8	10.6	10.1	9.3	11.2	12.0
	NA	0.3	0.3	0.5	0.5	1.6	2.7	3.7	5.3	7.4	9.3	20.7	43.6
ICP	Stationarity	92.3	94.7	97.1	95.2	92.3	87.8	87.0	85.1	81.9	81.1	66.2	44.4
	Non-stationary	7.4	5.1	2.4	4.0	5.3	9.0	8.8	8.5	10.1	8.5	11.2	10.4
	NA	0.3	0.3	0.5	0.8	2.4	3.2	4.3	6.4	8.0	10.4	22.6	45.2
CPP	Stationarity	94.7	95.7	96.8	95.7	90.2	87.0	83.2	81.6	81.1	78.7	63.8	42.6
	Non-stationary	4.8	3.7	2.4	3.2	6.9	8.8	11.4	10.9	9.8	9.6	12.8	11.7
	NA	0.5	0.5	0.8	1.1	2.9	4.3	5.3	7.4	9.0	11.7	23.4	45.7
PRx	Stationarity	97.9	96.3	96.3	92.8	89.6	87.8	86.2	82.2	75.8	76.1	62.8	42.6
	Non-stationary	1.6	3.2	2.9	6.1	7.4	8.0	8.5	10.1	15.2	12.5	13.8	11.2
	NA	0.5	0.5	0.8	1.1	2.9	4.3	5.3	7.7	9.0	11.4	23.4	46.3
PAx	Stationarity	98.4	94.7	96.0	91.8	87.8	85.9	83.5	80.6	80.3	79.0	60.9	41.2
	Non-stationary	1.1	4.8	3.2	7.2	9.3	9.8	11.2	11.7	10.6	9.6	15.7	12.5
	NA	0.5	0.5	0.8	1.1	2.9	4.3	5.3	7.7	9.0	11.4	23.4	46.3
RAC	Stationarity	98.1	96.3	94.9	91.8	88.8	87.5	83.5	80.1	79.8	79.5	63.0	39.6
	Non-stationary	1.3	3.2	4.3	7.2	8.2	8.2	11.2	12.2	11.2	9.0	13.6	14.1
	NA	0.5	0.5	0.8	1.1	2.9	4.3	5.3	7.7	9.0	11.4	23.4	46.3
RAP	Stationarity	98.1	95.7	95.2	93.4	89.1	84.8	86.4	81.6	77.4	77.4	61.4	43.6
	Non-stationary	1.6	4.0	4.3	5.9	8.5	12.0	9.3	12.0	14.4	12.2	16.0	10.9
	NA	0.3	0.3	0.5	0.8	2.4	3.2	4.3	6.4	8.2	10.4	22.6	45.5
COx_L	Stationarity	29.5	30.1	29.5	27.4	28.2	25.8	26.1	24.2	23.9	22.3	20.2	12.5
	Non-stationary	1.1	0.3	0.3	2.1	0.8	2.7	1.9	3.2	2.4	3.5	2.9	5.6
	NA	69.4	69.7	70.2	70.5	71.0	71.5	72.1	72.6	73.7	74.2	76.9	81.9
COx_R	Stationarity	35.9	34.8	33.5	33.5	31.4	30.1	29.5	27.4	24.5	22.3	19.9	12.5
	Non-stationary	0.5	1.3	2.1	1.9	2.9	3.7	4.3	5.3	7.2	8.0	4.3	2.4
	NA	63.6	63.8	64.4	64.6	65.7	66.2	66.2	67.3	68.4	69.7	75.8	85.1
COx-a_L	Stationarity	35.4	34.3	33.8	32.2	30.3	29.0	28.2	27.9	26.6	24.7	19.9	12.2
	Non-stationary	0.8	1.6	1.9	3.2	4.0	4.5	5.1	4.5	4.8	5.1	5.3	4.3
	NA	63.8	64.1	64.4	64.6	65.7	66.5	66.8	67.6	68.6	70.2	74.7	83.5
COx-a_R	Stationarity	35.6	34.6	32.7	33.8	29.8	29.5	30.1	26.6	26.1	23.1	19.9	12.5
	Non-stationary	0.0	1.1	2.4	1.3	4.8	4.5	4.0	6.6	5.9	7.4	4.5	2.4
	NA	64.4	64.4	64.9	64.9	65.4	66.0	66.0	66.8	68.1	69.4	75.5	85.1
rSO_2__L	Stationarity	34.8	33.5	33.2	32.7	31.9	29.3	25.5	29.0	26.9	25.0	20.2	11.2
	Non-stationary	0.5	1.9	1.9	2.4	2.7	4.3	7.7	3.5	4.5	4.8	5.1	5.6
	NA	64.6	64.6	64.9	64.9	65.4	66.5	66.8	67.6	68.6	70.2	74.7	83.2
rSO_2__R	Stationarity	35.9	35.6	34.6	34.8	34.3	31.1	31.6	31.9	30.6	29.0	19.7	11.4
	Non-stationary	1.1	1.1	1.9	1.6	1.9	4.5	4.0	3.2	2.7	3.5	6.1	4.5
	NA	63.0	63.3	63.6	63.6	63.8	64.4	64.4	64.9	66.8	67.6	74.2	84.0
PbtO_2_	Stationarity	35.4	35.6	35.4	34.6	34.0	32.2	32.2	29.3	30.1	29.0	23.1	13.6
	Non-stationary	1.1	0.8	0.8	1.6	1.6	2.4	2.1	4.5	2.4	2.9	3.7	4.0
	NA	63.6	63.6	63.8	63.8	64.4	65.4	65.7	66.2	67.6	68.1	73.1	82.4

COx_L, cerebral oximetry index of left hemisphere; COx_R, cerebral oximetry index of the right hemisphere; COx-a_L, COx with ABP of left hemisphere; COx-a_R, COx with ABP of right hemisphere; KPSS, Kwiatkowski–Phillips–Schmidt–Shin; CPP, cerebral perfusion pressure; ICP, intracranial pressure; MAP, mean arterial pressure; NA, not applicable; PAx, pulse amplitude index; PbtO_2_, cerebral oxygen saturation; PRx, pressure reactivity index; RAC, a cerebral autoregulation index; RAP, index of cerebral compensatory reserve; rSO_2__L, brain tissue oxygenation of the left hemisphere; rSO_2__R, brain tissue oxygenation of the right hemisphere.

**Table 4:** AIC-based median optimal model parameters of all signals across all resolutions.

Signal	1-min	5-min	10-min	30-min	1-h	2-h	3-h	4-h	5-h	6-h	12-h	1-day
MAP	(4,1,5)|32,437.6512	(4,1,4)|7566.1234	(3,1,4)|3771.6399	(3,1,4)|1340.8527	(3,1,3)|689.3287	(3,1,2)|359.4441	(4,1,2)|234.2886	(4,2,2)|175.9431	(4,2,2)|145.8193	(4,2,2)|113.2266	(5,2,2)|53.1833	(4,1,1)|21.1179
ICP	(5,1,6)|19,465.3870	(4,1,4)|5151.5914	(3,1,4)|2715.2870	(3,1,3)|972.4728	(3,1,3)|517.8965	(3,1,2)|260.7877	(3,1,2)|175.4765	(4,1,2)|131.0412	(4,1,1)|109.9212	(4,1,1)|85.5492	(5,1,1)|38.9008	(4,1,1)|15.3208
CPP	(4,1,5)|31,589.4163	(4,1,4)|7020.9794	(3,1,4)|3614.7909	(3,1,3)|1295.3518	(3,1,3)|637.6638	(3,1,2)|331.0755	(4,1,2)|220.7812	(4,2,2)|162.8390	(4,2,2)|136.4757	(4,2,2)|107.2640	(5,2,2)|48.0941	(4,1,1)|18.5961
PRx	(5,1,3)|−2705.5415	(3,1,3)|214.4056	(2,1,2)|−28.8594	(2,1,2)|−111.1637	(2,1,2)|−83.3895	(3,1,2)|−55.5690	(3,1,1)|−40.2198	(3,1,1)|−32.0176	(3,1,1)|−28.0848	(3,1,1)|−23.6506	(4,1,1)|−13.6186	(3,1,1)|−10.1447
PAx	(5,1,3)|−3317.5719	(3,1,2)|51.3212	(2,1,2)|−116.8506	(2,1,2)|−136.3830	(2,1,2)|−94.4192	(2,1,2)|−60.2984	(3,1,1)|−43.6922	(3,1,1)|−35.0696	(3,1,1)|−29.8718	(3,1,1)|−25.3364	(4,1,1)|−14.9201	(3,1,1)|−11.7097
RAC	(5,1,4)|−4173.7097	(3,1,3)|−45.2354	(2,1,2)|−132.4741	(2,1,2)|−120.6020	(2,1,2)|−80.1245	(3,1,1)|−49.4206	(3,1,1)|−35.5625	(3,1,1)|−27.7896	(3,1,1)|−24.4844	(3,1,1)|−20.5554	(4,1,1)|−12.2832	(3,1,1)|−10.0164
RAP	(5,1,4)|−6564.4567	(3,1,3)|−408.7564	(2,1,2)|−277.6390	(2,1,2)|−169.5420	(2,1,2)|−111.5923	(2,1,1)|−66.3179	(2,1,1)|−48.1936	(3,1,1)|−37.3456	(3,1,1)|−32.6441	(3,1,1)|−26.6239	(3,1,1)|−15.1382	(3,1,0)|−11.0359
COx_L	(3,1,3)|−7845.9869	(2,1,2)|−1136.6393	(2,1,2)|−706.4331	(2,1,2)|−342.2114	(2,1,1)|−200.3693	(3,1,1)|−110.7139	(3,1,1)|−81.1365	(3,1,1)|−63.0780	(3,1,1)|−55.8275	(3,1,1)|−45.7815	(3,1,1)|−26.8666	(3,1,0)|−16.0338
COx_R	(3,1,3)|−8619.7456	(2,1,2)|−1306.8703	(2,1,2)|−797.8962	(2,1,2)|−375.7313	(2,1,2)|−218.8351	(3,1,1)|−121.9732	(2,1,1)|−86.2215	(3,1,1)|−69.3222	(3,1,1)|−59.4612	(3,1,1)|−51.1055	(4,1,0)|−29.3271	(3,1,0)|−18.0534
COx-a_L	(4,1,3)|−2998.3556	(2,1,2)|−170.0081	(2,1,2)|−232.0380	(2,1,2)|−184.5377	(2,1,2)|−122.1370	(3,1,1)|−78.0123	(3,1,1)|−58.9056	(3,1,1)|−46.0589	(3,1,1)|−42.9210	(3,1,1)|−34.6218	(3,1,1)|−20.9843	(3,1,0)|−14.4151
COx-a_R	(4,1,3)|−3823.8320	(2,1,2)|−353.2426	(2,1,2)|−328.9916	(2,1,2)|−219.8479	(2,1,2)|−144.4329	(2,1,1)|−89.4807	(2,1,1)|−65.2765	(3,1,1)|−53.3349	(3,1,1)|−47.8409	(3,1,1)|−39.3885	(3,1,1)|−23.2328	(3,1,0)|−16.0076
rSO_2__L	(4,1,5)|6470.1141	(4,1,4)|2234.8637	(4,1,3)|1301.1568	(3,1,3)|517.5479	(3,1,2)|286.2400	(4,1,2)|151.0798	(4,1,2)|109.8429	(4,1,2)|80.0802	(4,2,1)|68.8821	(4,1,1)|53.3372	(4,1,1)|23.1638	(3,1,1)|7.8787
rSO_2__R	(5,1,5)|5483.7903	(4,1,4)|1743.9004	(3,1,3)|983.0151	(3,1,3)|455.9297	(3,1,2)|253.7859	(3,1,1)|151.4239	(3,1,2)|104.2982	(4,2,2)|71.2784	(4,1,1)|66.7480	(4,1,1)|54.0833	(4,1,1)|24.2109	(3,1,1)|6.6337
PbtO_2_	(4,1,5)|22,868.8849	(4,1,4)|5600.2201	(3,1,4)|2845.0132	(3,1,3)|1109.2239	(3,1,3)|569.8618	(3,1,2)|293.0777	(3,1,2)|204.1008	(3,2,2)|142.8140	(3,2,1)|126.6797	(4,2,2)|96.4337	(5,2,1)|42.9938	(4,1,1)|16.8122

COx_L, cerebral oximetry index of the left hemisphere; COx_R, cerebral oximetry index of the right hemisphere; COx-a_L, COx with ABP of the left hemisphere; COx-a_R, COx with ABP of the right hemisphere; CPP, cerebral perfusion pressure; ICP, intracranial pressure; MAP, mean arterial pressure; PAx, pulse amplitude index; PbtO_2_, cerebral oxygen saturation; PRx, pressure reactivity index; RAC, a cerebral autoregulation index; RAP, index of cerebral compensatory reserve; rSO_2__L, brain tissue oxygenation of the left hemisphere; rSO_2__R, brain tissue oxygenation of the right hemisphere.

**Table 5:** Significant findings from subgroup comparisons for a 1-min temporal resolution.

Signal	Group	Subgroup 1	Subgroup 2	Metric	Test	*p*-Value
PAx	Age	≥40	<40	AIC	Mann–Whitney U	0.03753
PAx	Age	≥40	<40	BIC	Mann–Whitney U	0.0396
PAx	Age	≥40	<40	LL	Mann–Whitney U	0.0367
RAC	Age	≥40	<40	AIC	Mann–Whitney U	0.00155
RAC	Age	≥40	<40	BIC	Mann–Whitney U	0.00158
RAC	Age	≥40	<40	LL	Mann–Whitney U	0.00152
RAP	Age	≥40	<40	AIC	Mann–Whitney U	0.04067
RAP	Age	≥40	<40	BIC	Mann–Whitney U	0.04049
RAP	Age	≥40	<40	LL	Mann–Whitney U	0.04013
MAP	Marshall CT score	≥5	<5	AIC	Mann–Whitney U	0.00012
MAP	Marshall CT score	≥5	<5	BIC	Mann–Whitney U	0.00012
MAP	Marshall CT score	≥5	<5	LL	Mann–Whitney U	0.00011
ICP	Marshall CT score	≥5	<5	AIC	Mann–Whitney U	0.00025
ICP	Marshall CT score	≥5	<5	BIC	Mann–Whitney U	0.00025
ICP	Marshall CT score	≥5	<5	LL	Mann–Whitney U	0.00028
PRx	Marshall CT score	≥5	<5	AIC	Mann–Whitney U	0.01395
PRx	Marshall CT score	≥5	<5	BIC	Mann–Whitney U	0.01425
PRx	Marshall CT score	≥5	<5	LL	Mann–Whitney U	0.01381
PAx	Marshall CT score	≥5	<5	AIC	Mann–Whitney U	0.0085
PAx	Marshall CT score	≥5	<5	BIC	Mann–Whitney U	0.00908
PAx	Marshall CT score	≥5	<5	LL	Mann–Whitney U	0.00835
RAC	Marshall CT score	≥5	<5	AIC	Mann–Whitney U	0.00097
RAC	Marshall CT score	≥5	<5	BIC	Mann–Whitney U	0.00097
RAC	Marshall CT score	≥5	<5	LL	Mann–Whitney U	0.00096
RAP	Marshall CT score	≥5	<5	AIC	Mann–Whitney U	2.00 × 10^−5^
RAP	Marshall CT score	≥5	<5	BIC	Mann–Whitney U	1.00 × 10^−5^
RAP	Marshall CT score	≥5	<5	LL	Mann–Whitney U	2.00 × 10^−5^
COx_L	Marshall CT score	≥5	<5	AIC	Mann–Whitney U	0.00868
COx_L	Marshall CT score	≥5	<5	BIC	Mann–Whitney U	0.00884
COx_L	Marshall CT score	≥5	<5	LL	Mann–Whitney U	0.00868
COx_R	Marshall CT score	≥5	<5	AIC	Mann–Whitney U	0.0214
COx_R	Marshall CT score	≥5	<5	BIC	Mann–Whitney U	0.0214
COx_R	Marshall CT score	≥5	<5	LL	Mann–Whitney U	0.02035
COx-a_L	Marshall CT score	≥5	<5	AIC	Mann–Whitney U	0.00361
COx-a_L	Marshall CT score	≥5	<5	BIC	Mann–Whitney U	0.00369
COx-a_L	Marshall CT score	≥5	<5	LL	Mann–Whitney U	0.00361
COx-a_R	Marshall CT score	≥5	<5	AIC	Mann–Whitney U	0.00918
COx-a_R	Marshall CT score	≥5	<5	BIC	Mann–Whitney U	0.00952
COx-a_R	Marshall CT score	≥5	<5	LL	Mann–Whitney U	0.00884
rSO_2__L	Marshall CT score	≥5	<5	AIC	Mann–Whitney U	0.00022
rSO_2__L	Marshall CT score	≥5	<5	BIC	Mann–Whitney U	0.00023
rSO_2__L	Marshall CT score	≥5	<5	LL	Mann–Whitney U	0.00025
rSO_2__R	Marshall CT score	≥5	<5	AIC	Mann–Whitney U	0.03532
rSO_2__R	Marshall CT score	≥5	<5	BIC	Mann–Whitney U	0.03478
rSO_2__R	Marshall CT score	≥5	<5	LL	Mann–Whitney U	0.03532
PRx	Pupils	Unilateral Unreactive	Bilat Reactive	AIC	Kruskal–Wallis	0.01611
PRx	Pupils	Unilateral Unreactive	Bilat Reactive	BIC	Kruskal–Wallis	0.01645
PRx	Pupils	Unilateral Unreactive	Bilat Reactive	LL	Kruskal–Wallis	0.016
PAx	Pupils	Unilateral Unreactive	Bilat Reactive	AIC	Kruskal–Wallis	0.03663
PAx	Pupils	Unilateral Unreactive	Bilat Reactive	BIC	Kruskal–Wallis	0.03469
PAx	Pupils	Unilateral Unreactive	Bilat Reactive	LL	Kruskal–Wallis	0.03681
RAP	Pupils	Unilateral Unreactive	Bilat Reactive	AIC	Kruskal–Wallis	0.02379
RAP	Pupils	Unilateral Unreactive	Bilat Reactive	BIC	Kruskal–Wallis	0.02388
RAP	Pupils	Unilateral Unreactive	Bilat Reactive	LL	Kruskal–Wallis	0.02363
RAP	Sex	M	F	AIC	Mann–Whitney U	0.04272
RAP	Sex	M	F	BIC	Mann–Whitney U	0.04272
RAP	Sex	M	F	LL	Mann–Whitney U	0.04319

AIC, Akaike Information Criterion; BIC, Bayesian Information Criterion; CT, computed tomography; COx_L, cerebral oximetry index of the left hemisphere; COx_R, cerebral oximetry index of the right hemisphere; COx-a_L, COx with ABP of the left hemisphere; COx-a_R, COx with ABP of the right hemisphere; ICP, intracranial pressure; F, female; M, male; MAP, mean arterial pressure; LL, Log-Likelihood; PAx, pulse amplitude index; PRx, pressure reactivity index; RAC, a cerebral autoregulation index; RAP, index of cerebral compensatory reserve; rSO_2__L, brain tissue oxygenation of the left hemisphere; rSO_2__R, brain tissue oxygenation of the right hemisphere.

**Table 6:** ICP forecasting results with the optimized ARIMA parameters for the small sample size.

Patient	Pearson Correlation	BA Mean	BA Lower LoA	BA Upper LoA
1	0.38	0.01	−3.41	3.42
2	0.29	−0.02	−1.58	1.55
3	0.00	−0.07	−0.95	0.81
4	0.15	−0.07	−2.85	2.71
5	0.35	0.02	−1.82	1.85
6	0.27	−0.02	−1.19	1.16
7	0.19	0.02	−1.85	1.89
8	0.10	0.00	−1.92	1.92
9	0.37	0.02	−2.22	2.26
10	0.01	0.00	−1.13	1.13
11	0.08	−0.01	−1.13	1.11
12	0.31	0.00	−1.84	1.85
13	0.10	−0.29	−2.54	1.97

BA, Bland–Altman; LoA, limits of agreement.

## Data Availability

Data accessed for the purposes of this study will not be publicly available, as it consists of sensitive patient data protected under each participating site’s health jurisdiction.

## Supplementary Materials

The following supporting information can be downloaded at: https://www.mdpi.com/article/10.3390/s25092762/s1, Table S1: *p*-values of ADF and KPSS tests on original data of all patients, Table S2: *p*-values of ADF and KPSS tests on first-order differenced data of all patients, Table S3: Grid search results of one patient for the raw physiologic signals with respect to AIC, BIC and LL values, Table S4: Grid search results of one patient for the derived physiologic signals with respect to AIC, BIC and LL values, Table S5: Median computational time of each (*p*,*d*,*q*) parameter combination across all patients for all physiologic signals at 1-min resolution, Table S6: Percentage comparison of stationarity and non-stationarity based on ADF test on the original data, Table S7: Percentage comparison of stationarity and non-stationarity based on ADF test on the first-order differenced data.
