# Supplementary material for: Impact of Temporal Resolution on Autocorrelative Features of Cerebral Physiology from Invasive and Non-Invasive Sensors in Acute Traumatic Neural Injury: Insights from the CAHR-TBI Cohort
Source: Sensors (Basel). 2025 Apr 27;25(9):2762. doi: 10.3390/s25092762 (PMC12074187; doi:10.3390/s25092762)
Supplement: Supplementary file 1 [file sensors-25-02762-s001.zip › sensors-3525098-supplementary.pdf]

Table of Contents

Table S1 - p-values of ADF and KPSS tests on original data of all patients 2
Table S2 - p-values of ADF and KPSS tests on first-order differenced data of all patients 17
Table S3 - Grid search results of one patient for the raw physiologic signals with respect to AIC, BIC and LL values 33
Table S4 - Grid search results of one patient for the derived physiologic signals with respect to AIC, BIC and LL values 38
Table S5 - Median computational time of each (*p*,*d*,*q*) parameter combination across all patients for all physiologic signals at 1-min resolution 49
Table S6 - Percentage comparison of stationarity and non-stationarity based on ADF test on the original data 50
Table S7 - Percentage comparison of stationarity and non-stationarity based on ADF test on the first-order differenced data 51

ACF, autocorrelation function; AIC, Akaike information criterion; ARIMA, autoregressive integrated moving average; BIC, bayesian information criterion; COx_L, cerebral oximetry index of left hemisphere; COx_R, cerebral oximetry index of right hemisphere; COx-a_L, COx with arterial blood pressure (ABP) of left hemisphere; COx-a_R, COx with ABP of right hemisphere; CPP, cerebral perfusion pressure; ICP, intracranial pressure; KPSS, Kwiatkowski-Phillips-Schmidt-Shin; LL, Log-Likelihood; MAP, mean arterial pressure; NA, not applicable; PAx, pulse amplitude index; $PbtO_2$, cerebral oxygen saturation; PRx, pressure reactivity index; RAC, a cerebral autoregulation index; RAP, index of cerebral compensatory reserve; rSO_2_L, brain tissue oxygenation of left hemisphere; rSO_2_R, brain tissue oxygenation of right hemisphere.

Table S1 - p-values of ADF and KPSS tests on original data of all patients

p-values of ADF test on original data														
**Patient**	**MAP**	**ICP**	**CPP**	**PRx**	**PAx**	**RAC**	**RAP**	**$PbtO_2$**	**COx_L**	**COx_R**	**COx-a_L**	**COx-a_R**	**rSO_2_L**	**rSO_2_R**
1	7.74E-08	7.13E-01	3.27E-03	8.62E-16	2.66E-10	3.12E-10	6.46E-14	NA	NA	NA	NA	NA	NA	NA
2	2.38E-07	8.29E-21	3.42E-06	1.84E-26	1.90E-24	3.06E-13	6.43E-27	NA	NA	NA	NA	NA	NA	NA
3	1.56E-02	9.47E-03	2.07E-02	1.46E-05	2.14E-01	1.90E-04	2.45E-04	NA	NA	NA	NA	NA	NA	NA
4	3.99E-12	7.62E-02	2.20E-05	2.54E-10	2.62E-07	2.38E-11	2.37E-22	3.93E-02	2.54E-23	2.70E-27	0.00E+00	1.73E-27	1.82E-01	2.16E-01
5	1.08E-02	4.01E-07	2.22E-01	2.93E-26	1.52E-27	6.39E-17	1.15E-21	NA	NA	NA	NA	NA	NA	NA
6	2.02E-08	1.45E-05	5.17E-17	1.24E-11	7.76E-12	2.68E-09	4.39E-13	NA	NA	NA	NA	NA	NA	NA
7	8.40E-04	1.41E-01	1.07E-01	2.69E-09	1.07E-18	1.32E-10	6.14E-10	NA	NA	NA	NA	NA	NA	NA
8	3.54E-09	4.02E-15	4.24E-07	2.92E-14	1.27E-13	1.75E-15	4.72E-17	NA	NA	NA	NA	NA	NA	NA
9	4.82E-10	6.98E-08	2.65E-09	1.25E-08	9.30E-15	3.83E-09	3.75E-13	NA	NA	NA	NA	NA	NA	NA
10	8.43E-21	1.42E-27	8.86E-19	5.52E-29	2.97E-23	2.52E-27	2.28E-20	NA	NA	NA	NA	NA	NA	NA
11	7.65E-03	3.02E-04	2.76E-02	6.06E-22	6.35E-18	5.36E-09	5.72E-10	NA	NA	NA	NA	NA	NA	NA
12	1.62E-20	4.01E-05	1.69E-11	0.00E+00	3.98E-30	2.03E-30	1.17E-27	NA	NA	NA	NA	NA	NA	NA
13	1.80E-19	3.32E-14	7.43E-14	6.80E-26	4.44E-17	1.00E-22	1.49E-16	NA	NA	NA	NA	NA	NA	NA
14	NA	2.49E-04	NA	NA	NA	NA	5.30E-08	NA	NA	NA	NA	NA	NA	NA
15	1.14E-10	3.25E-15	9.09E-11	2.33E-26	5.53E-30	1.37E-11	9.58E-08	NA	NA	NA	NA	NA	NA	NA
16	3.93E-07	1.68E-05	3.66E-07	0.00E+00	0.00E+00	4.08E-21	1.60E-19	NA	NA	NA	NA	NA	NA	NA
17	1.95E-10	3.73E-20	6.01E-11	5.47E-26	1.83E-18	2.05E-21	5.96E-22	5.02E-14	2.31E-23	1.18E-24	0.00E+00	5.19E-24	1.08E-04	4.17E-04
18	7.85E-06	2.26E-04	1.80E-07	3.34E-30	4.37E-17	2.30E-15	1.76E-15	1.08E-15	2.05E-20	6.73E-28	1.06E-17	3.18E-27	9.32E-11	2.25E-02
19	2.07E-19	1.38E-10	1.01E-17	2.83E-29	7.15E-21	1.03E-22	1.78E-11	7.84E-21	7.58E-10	2.41E-14	4.58E-14	1.23E-11	5.00E-03	9.25E-02
20	2.09E-06	4.00E-06	3.74E-06	5.08E-16	1.12E-17	4.16E-11	1.23E-09	9.48E-01	NA	1.20E-29	NA	0.00E+00	NA	2.41E-03
21	1.37E-14	1.39E-06	1.79E-13	2.25E-30	2.23E-26	8.02E-20	4.59E-12	1.32E-11	2.71E-20	7.25E-18	2.87E-21	3.21E-17	5.76E-10	1.02E-12
22	6.97E-13	2.28E-30	1.00E-15	2.08E-30	4.94E-29	4.10E-26	0.00E+00	4.71E-01	0.00E+00	0.00E+00	2.03E-30	0.00E+00	5.63E-14	2.25E-05
23	3.15E-17	8.24E-11	5.05E-10	8.59E-22	1.03E-18	7.22E-10	1.07E-14	NA	NA	NA	NA	NA	NA	NA
24	7.57E-07	1.43E-18	1.73E-10	1.00E-25	1.91E-22	2.69E-20	2.89E-19	3.87E-19	2.35E-30	2.70E-24	0.00E+00	0.00E+00	3.50E-09	1.08E-05
25	6.16E-13	9.33E-06	7.35E-17	1.37E-25	2.69E-20	4.40E-16	2.24E-13	1.18E-15	NA	NA	NA	NA	NA	NA
26	9.11E-25	9.94E-07	6.35E-26	7.00E-25	1.66E-23	8.12E-30	7.18E-22	NA	0.00E+00	0.00E+00	0.00E+00	0.00E+00	2.52E-08	3.45E-17
27	5.25E-20	7.31E-07	6.69E-17	7.95E-21	9.29E-20	1.68E-19	2.22E-09	2.05E-08	3.94E-26	4.22E-30	4.25E-24	4.21E-30	3.10E-08	3.67E-03
28	1.28E-07	8.23E-02	3.15E-05	2.39E-07	6.25E-06	1.37E-06	3.76E-06	1.87E-01	1.14E-24	1.20E-20	7.85E-28	7.42E-27	1.67E-06	6.33E-02
29	1.65E-12	5.84E-12	5.74E-16	0.00E+00	0.00E+00	0.00E+00	0.00E+00	NA	NA	NA	NA	NA	NA	NA
30	1.16E-09	5.49E-01	4.42E-01	4.39E-02	6.51E-05	2.18E-04	9.55E-02	9.68E-01	2.11E-02	1.12E-01	6.43E-30	9.89E-04	1.36E-03	6.91E-01
31	1.96E-05	9.18E-04	1.02E-03	1.81E-07	1.06E-08	6.59E-09	3.22E-04	NA	1.93E-10	2.15E-15	3.48E-11	7.72E-17	8.96E-01	4.61E-01
32	1.36E-14	NA	NA	NA	NA	NA	NA	NA	NA	NA	2.03E-30	1.73E-26	9.02E-06	1.64E-04
33	2.70E-23	5.90E-15	2.98E-16	1.31E-16	7.18E-18	1.55E-14	1.70E-17	NA	NA	NA	NA	NA	NA	NA
34	5.97E-03	5.22E-10	1.03E-07	3.09E-13	5.72E-15	1.81E-10	9.15E-03	NA	NA	NA	NA	NA	NA	NA
35	8.29E-05	1.81E-06	7.62E-03	1.74E-23	5.67E-11	4.95E-06	1.43E-16	NA	NA	NA	NA	NA	NA	NA
36	9.46E-09	9.72E-19	4.77E-21	1.46E-13	1.00E-10	3.97E-09	1.58E-07	NA	NA	NA	NA	NA	NA	NA
37	4.98E-11	5.84E-07	2.10E-10	3.13E-22	6.55E-23	9.46E-24	4.77E-27	NA	NA	NA	NA	NA	NA	NA
38	1.79E-05	9.65E-11	7.90E-05	1.11E-12	6.73E-10	5.12E-09	1.18E-13	NA	NA	NA	NA	NA	NA	NA
39	1.05E-03	2.85E-05	2.82E-04	2.81E-29	2.08E-13	6.05E-14	8.12E-13	NA	NA	NA	NA	NA	NA	NA
40	4.20E-17	3.62E-27	7.49E-17	9.24E-19	2.97E-22	2.18E-13	1.39E-16	NA	NA	NA	NA	NA	NA	NA
41	4.91E-23	0.00E+00	1.03E-21	4.06E-23	2.14E-23	3.34E-19	1.98E-24	NA	NA	NA	NA	NA	NA	NA
42	1.67E-16	1.54E-06	3.42E-10	2.02E-18	1.89E-17	4.73E-21	1.89E-25	NA	NA	NA	NA	NA	NA	NA
43	3.00E-05	3.17E-04	5.42E-03	5.35E-04	6.10E-06	2.57E-04	1.27E-06	NA	NA	NA	NA	NA	NA	NA
44	2.61E-03	3.42E-09	1.29E-03	1.70E-10	3.33E-13	9.80E-11	2.08E-13	NA	NA	NA	NA	NA	NA	NA
45	5.76E-08	7.50E-11	2.39E-14	9.99E-20	1.66E-28	8.42E-28	0.00E+00	NA	NA	NA	NA	NA	NA	NA
46	9.21E-12	2.94E-17	2.30E-08	1.05E-22	3.26E-22	1.42E-13	7.60E-08	NA	NA	NA	NA	NA	NA	NA

47	2.21E-03	1.57E-27	7.13E-06	2.15E-30	3.61E-24	1.81E-20	4.00E-17	NA	NA	NA	NA	NA	NA	NA
48	1.11E-04	4.56E-17	3.11E-05	2.20E-26	7.23E-18	5.07E-14	2.06E-15	NA	NA	NA	NA	NA	NA	NA
49	1.33E-05	6.53E-16	1.51E-12	2.65E-15	1.04E-22	4.07E-17	5.32E-22	NA	NA	NA	NA	NA	NA	NA
50	4.22E-11	5.47E-11	6.67E-10	6.15E-08	2.07E-06	1.08E-04	0.00E+00	NA	NA	NA	NA	NA	NA	NA
51	1.54E-11	1.82E-12	6.64E-07	1.69E-17	2.83E-17	4.39E-16	7.74E-08	NA	NA	NA	NA	NA	NA	NA
52	1.37E-10	1.90E-18	1.15E-06	3.36E-29	2.95E-14	3.63E-23	1.05E-25	NA	NA	NA	NA	NA	NA	NA
53	6.09E-17	4.60E-15	3.40E-19	7.94E-27	1.58E-19	4.63E-22	9.06E-23	NA	NA	NA	NA	NA	NA	NA
54	9.16E-02	2.23E-06	7.01E-03	1.39E-07	1.51E-19	3.72E-16	5.60E-13	NA	NA	NA	NA	NA	NA	NA
55	2.69E-14	2.23E-28	4.48E-14	0.00E+00	3.61E-29	1.30E-14	2.93E-14	NA	NA	NA	NA	NA	NA	NA
56	7.80E-02	5.17E-02	5.20E-01	1.78E-02	4.26E-03	3.61E-02	2.58E-05	NA	NA	NA	NA	NA	NA	NA
57	4.32E-07	4.75E-25	1.22E-07	2.11E-22	0.00E+00	2.01E-18	5.18E-18	NA	NA	NA	NA	NA	NA	NA
58	3.18E-17	6.15E-05	2.23E-07	0.00E+00	3.00E-29	7.31E-21	1.56E-25	NA	NA	NA	NA	NA	NA	NA
59	1.24E-08	2.06E-30	7.58E-15	8.43E-23	1.50E-26	6.16E-21	2.65E-20	NA	NA	NA	NA	NA	NA	NA
60	9.58E-17	2.04E-12	1.73E-15	4.44E-26	0.00E+00	0.00E+00	3.12E-20	NA	NA	NA	NA	NA	NA	NA
61	2.16E-19	3.65E-09	3.38E-24	5.07E-13	1.71E-10	2.57E-24	0.00E+00	NA	NA	NA	NA	NA	NA	NA
62	1.60E-23	2.21E-11	8.53E-20	4.12E-13	3.58E-28	4.75E-20	8.72E-15	NA	NA	NA	NA	NA	NA	NA
63	6.98E-16	8.37E-25	3.62E-25	6.25E-24	7.73E-25	6.71E-24	2.83E-26	NA	NA	NA	NA	NA	NA	NA
64	7.30E-16	0.00E+00	6.48E-18	1.52E-17	2.24E-28	2.26E-25	5.56E-25	NA	NA	NA	NA	NA	NA	NA
65	8.11E-07	0.00E+00	1.23E-18	3.49E-19	1.43E-22	7.64E-25	7.94E-29	NA	NA	NA	NA	NA	NA	NA
66	1.01E-14	2.04E-21	1.94E-14	3.67E-28	2.35E-30	2.09E-26	6.80E-26	NA	NA	NA	NA	NA	NA	NA
67	2.78E-11	3.12E-07	8.54E-12	1.94E-21	1.65E-16	3.76E-06	8.57E-14	NA	NA	NA	NA	NA	NA	NA
68	2.36E-04	4.56E-05	1.69E-06	9.31E-20	8.21E-21	1.92E-06	1.40E-09	NA	NA	NA	NA	NA	NA	NA
69	1.04E-11	9.60E-13	1.54E-13	2.49E-14	8.79E-25	2.73E-25	2.35E-07	NA	NA	NA	NA	NA	NA	NA
70	3.61E-07	2.94E-01	5.71E-05	3.20E-23	1.08E-18	6.05E-20	1.45E-11	NA	NA	NA	NA	NA	NA	NA
71	9.75E-26	3.36E-24	8.99E-22	2.05E-30	3.85E-30	9.63E-24	1.40E-28	NA	NA	NA	NA	NA	NA	NA
72	2.57E-20	5.42E-18	1.27E-11	6.34E-20	7.03E-21	1.11E-20	3.16E-23	NA	NA	NA	NA	NA	NA	NA
73	1.07E-10	1.28E-08	1.16E-13	9.08E-20	2.36E-22	8.30E-07	2.08E-07	NA	NA	NA	NA	NA	NA	NA
74	7.48E-12	4.36E-29	2.78E-12	6.14E-29	2.59E-22	3.41E-25	2.32E-29	NA	NA	NA	NA	NA	NA	NA
75	4.09E-08	1.07E-01	1.54E-01	2.26E-09	5.75E-15	3.46E-14	1.77E-23	NA	NA	NA	NA	NA	NA	NA
76	3.09E-20	1.47E-21	2.77E-16	6.00E-21	2.53E-20	1.37E-09	1.13E-10	NA	NA	NA	NA	NA	NA	NA
77	4.24E-05	4.76E-17	6.20E-04	5.59E-25	2.91E-16	1.15E-14	3.80E-09	NA	NA	NA	NA	NA	NA	NA
78	7.93E-13	7.69E-03	1.85E-12	5.76E-07	1.12E-07	1.37E-04	2.14E-08	NA	NA	NA	NA	NA	NA	NA
79	2.29E-04	7.34E-06	5.62E-06	1.86E-29	1.06E-16	2.02E-19	9.89E-11	NA	NA	NA	NA	NA	NA	NA
80	3.53E-15	1.88E-27	2.98E-21	7.20E-21	4.61E-18	1.43E-10	4.92E-06	NA	NA	NA	NA	NA	NA	NA
81	6.91E-09	1.13E-05	1.10E-04	5.28E-19	2.95E-14	2.29E-12	2.91E-18	NA	NA	NA	NA	NA	NA	NA
82	1.14E-09	4.79E-06	9.20E-10	3.00E-10	8.01E-10	8.77E-09	1.13E-11	NA	NA	NA	NA	NA	NA	NA
83	5.01E-18	4.80E-18	1.58E-21	3.41E-22	3.10E-16	1.25E-14	3.14E-16	NA	NA	NA	NA	NA	NA	NA
84	8.68E-05	4.03E-11	1.47E-05	4.09E-30	7.03E-29	5.75E-25	2.48E-27	NA	NA	NA	NA	NA	NA	NA
85	2.38E-07	2.89E-10	9.58E-08	2.81E-16	6.76E-15	9.81E-11	7.97E-23	1.40E-09	NA	NA	NA	NA	NA	NA
86	1.31E-10	1.10E-21	1.67E-12	1.27E-26	0.00E+00	3.19E-30	7.04E-22	NA	NA	NA	NA	NA	NA	NA
87	4.23E-17	2.11E-23	9.63E-17	2.47E-23	4.21E-25	2.71E-28	1.21E-26	NA	NA	NA	NA	NA	NA	NA
88	3.76E-02	1.42E-03	2.05E-04	3.58E-17	3.22E-29	4.91E-06	7.08E-07	NA	NA	NA	NA	NA	NA	NA
89	5.42E-04	1.45E-05	3.23E-07	1.54E-08	1.59E-09	1.02E-06	1.30E-05	NA	NA	NA	NA	NA	NA	NA
90	8.61E-26	4.64E-06	8.61E-26	0.00E+00	7.32E-30	2.15E-27	2.31E-26	NA	NA	NA	NA	NA	NA	NA
91	6.52E-12	3.69E-15	5.03E-22	9.35E-20	1.40E-27	2.95E-21	4.33E-12	NA	NA	NA	NA	NA	NA	NA
92	2.40E-04	1.17E-20	1.47E-11	8.48E-11	9.90E-16	1.02E-09	7.27E-11	NA	NA	NA	NA	NA	NA	NA
93	2.02E-14	7.73E-06	3.63E-15	2.85E-15	9.62E-20	4.30E-19	1.81E-21	NA	NA	NA	NA	NA	NA	NA
94	2.24E-06	2.01E-11	1.86E-07	5.94E-16	2.43E-13	1.79E-16	1.55E-15	NA	NA	NA	NA	NA	NA	NA
95	0.00E+00	2.30E-16	0.00E+00	4.84E-29	5.74E-29	2.03E-30	6.01E-21	NA	NA	NA	NA	NA	NA	NA

96	5.79E-11	4.66E-21	4.05E-13	5.48E-25	2.17E-21	2.71E-21	3.01E-25	NA	NA	NA	NA	NA	NA	NA
97	1.00E-02	1.33E-06	1.39E-03	3.05E-24	1.62E-26	7.90E-13	1.54E-07	NA	NA	NA	NA	NA	NA	NA
98	2.47E-02	6.94E-03	2.33E-01	2.93E-26	6.79E-25	1.61E-26	7.76E-17	NA	NA	NA	NA	NA	NA	NA
99	1.38E-04	1.36E-04	1.84E-11	4.23E-13	5.16E-09	7.12E-08	5.59E-04	2.81E-11	NA	NA	NA	NA	NA	NA
100	5.84E-09	2.54E-12	4.03E-07	1.29E-09	7.08E-18	7.30E-18	1.24E-20	2.86E-18	NA	NA	NA	NA	NA	NA
101	5.65E-15	1.82E-24	4.28E-18	1.15E-17	2.07E-30	6.53E-24	1.21E-13	9.15E-20	NA	NA	NA	NA	NA	NA
102	3.38E-04	2.57E-09	5.75E-06	1.95E-08	7.29E-08	2.37E-05	3.45E-06	1.69E-01	NA	NA	NA	NA	NA	NA
103	7.75E-28	2.28E-04	2.68E-27	5.94E-30	6.35E-23	4.91E-19	3.72E-19	1.93E-23	NA	NA	NA	NA	NA	NA
104	7.68E-09	2.11E-14	3.00E-05	1.13E-27	1.75E-27	1.90E-27	1.04E-17	NA	NA	NA	NA	NA	NA	NA
105	1.30E-13	6.31E-13	1.33E-13	2.52E-18	7.05E-15	2.31E-13	5.11E-08	1.56E-01	NA	NA	NA	NA	NA	NA
106	1.64E-27	4.73E-17	8.52E-25	3.66E-25	4.46E-29	1.31E-21	1.62E-16	8.25E-30	NA	NA	NA	NA	NA	NA
107	8.73E-06	6.48E-09	7.11E-05	6.74E-28	3.30E-11	3.28E-12	1.88E-08	5.45E-03	NA	NA	NA	NA	NA	NA
108	1.61E-14	4.70E-09	1.53E-18	1.39E-15	1.67E-20	1.38E-24	2.39E-15	9.05E-01	NA	NA	NA	NA	NA	NA
109	3.21E-03	7.48E-21	2.27E-13	3.70E-10	2.98E-15	4.34E-21	2.48E-11	6.03E-01	NA	NA	NA	NA	NA	NA
110	3.67E-21	2.83E-10	8.23E-20	9.13E-26	2.03E-21	5.00E-20	3.05E-18	5.30E-22	NA	NA	NA	NA	NA	NA
111	2.59E-08	1.47E-05	1.03E-08	2.47E-04	6.32E-05	4.81E-04	5.57E-09	2.19E-17	1.29E-19	2.07E-30	9.92E-29	2.04E-30	1.07E-01	6.41E-04
112	6.08E-24	7.74E-08	4.39E-25	0.00E+00	1.48E-29	2.12E-24	3.33E-13	2.17E-23	NA	1.56E-27	NA	0.00E+00	NA	5.65E-05
113	1.64E-20	5.60E-12	1.34E-16	7.63E-23	2.17E-14	1.25E-16	5.79E-20	NA	7.22E-16	0.00E+00	1.40E-19	2.03E-30	2.28E-02	3.68E-02
114	2.46E-08	6.19E-01	3.09E-01	2.21E-06	3.88E-14	1.67E-03	2.82E-03	2.68E-01	NA	NA	NA	NA	NA	NA
115	5.46E-13	1.43E-27	1.31E-13	8.27E-23	6.27E-16	3.43E-25	7.74E-29	NA	NA	NA	NA	NA	NA	NA
116	5.97E-08	7.19E-16	6.52E-18	4.34E-23	5.25E-20	1.91E-17	2.67E-28	9.41E-13	NA	NA	NA	NA	NA	NA
117	2.18E-19	1.24E-04	1.24E-02	6.57E-07	8.70E-06	5.34E-08	3.96E-09	5.25E-30	NA	NA	NA	NA	NA	NA
118	5.06E-01	9.40E-01	7.64E-01	8.98E-04	4.53E-01	5.58E-02	1.52E-01	9.73E-01	NA	NA	NA	NA	NA	NA
119	7.07E-10	9.98E-06	9.88E-09	2.66E-27	3.06E-10	7.37E-06	2.58E-23	8.74E-09	NA	NA	NA	NA	NA	NA
120	6.46E-19	2.25E-13	9.17E-23	1.06E-27	2.22E-27	2.95E-18	3.97E-18	2.25E-01	0.00E+00	0.00E+00	7.33E-22	0.00E+00	1.68E-03	5.01E-02
121	2.62E-06	4.53E-07	1.34E-05	2.68E-14	4.49E-21	5.35E-21	3.74E-12	8.72E-05	2.67E-11	1.94E-10	4.36E-09	2.96E-10	9.68E-05	1.17E-02
122	2.39E-08	4.54E-08	6.59E-08	4.80E-11	6.38E-16	8.84E-08	0.00E+00	1.97E-13	6.25E-27	0.00E+00	0.00E+00	0.00E+00	3.38E-05	2.69E-04
123	1.56E-12	9.12E-10	4.40E-13	5.28E-08	2.66E-13	1.25E-09	3.05E-07	8.31E-01	2.83E-24	2.21E-08	8.59E-30	1.82E-21	2.24E-03	8.13E-05
124	3.20E-14	3.34E-03	2.49E-12	2.02E-16	1.96E-15	3.00E-06	2.10E-12	NA	NA	NA	NA	NA	NA	NA
125	3.66E-04	5.18E-01	3.96E-08	1.26E-13	2.78E-11	4.96E-10	7.48E-04	NA	1.55E-11	5.23E-24	1.30E-11	6.48E-23	7.24E-01	2.58E-01
126	9.93E-09	9.87E-01	1.17E-05	3.81E-10	6.55E-14	7.97E-12	1.83E-06	NA	NA	0.00E+00	NA	0.00E+00	NA	3.13E-03
127	5.40E-05	3.50E-02	4.03E-06	9.23E-06	1.02E-09	3.93E-07	1.23E-08	NA	NA	NA	NA	NA	NA	NA
128	6.68E-08	3.59E-02	2.82E-08	6.41E-11	9.58E-10	1.26E-09	3.36E-11	NA	NA	NA	NA	NA	NA	NA
129	2.27E-04	7.13E-02	9.07E-08	7.96E-10	3.77E-07	1.43E-08	2.88E-03	NA	3.92E-13	1.83E-03	2.78E-12	1.38E-03	8.73E-02	4.54E-01
130	6.39E-07	4.28E-02	3.40E-05	6.27E-05	1.99E-23	2.69E-23	2.38E-08	NA	1.11E-14	6.97E-24	3.06E-21	1.09E-09	5.13E-01	6.76E-02
131	2.13E-05	2.77E-05	6.80E-10	6.13E-19	5.35E-14	1.07E-16	1.80E-19	NA	1.49E-25	2.66E-26	1.67E-26	1.26E-26	6.47E-04	2.79E-03
132	4.35E-12	1.24E-01	2.54E-10	8.97E-13	3.24E-10	1.58E-08	5.27E-05	NA	NA	NA	NA	NA	NA	NA
133	1.39E-02	8.88E-01	2.26E-02	2.03E-11	6.82E-04	1.79E-03	4.35E-04	NA	NA	NA	NA	NA	NA	NA
134	1.78E-04	7.92E-03	1.37E-03	8.35E-11	1.31E-11	9.34E-12	7.85E-11	NA	NA	NA	NA	NA	NA	NA
135	1.32E-17	1.24E-01	1.91E-16	1.33E-07	2.52E-28	1.98E-26	3.37E-11	NA	NA	NA	NA	NA	NA	NA
136	2.26E-05	2.13E-02	2.13E-04	1.78E-08	8.86E-11	5.71E-12	2.76E-12	NA	NA	NA	NA	NA	NA	NA
137	2.37E-03	2.43E-17	3.12E-03	2.01E-23	1.45E-11	4.03E-07	3.77E-03	NA	NA	NA	NA	NA	NA	NA
138	2.81E-08	2.42E-13	2.65E-13	4.31E-26	6.55E-23	6.56E-22	6.57E-23	NA	NA	NA	NA	NA	NA	NA
139	9.93E-01	4.67E-01	9.71E-01	1.56E-08	2.43E-05	2.59E-07	1.72E-07	NA	NA	NA	NA	NA	NA	NA
140	2.08E-10	3.59E-04	7.85E-12	1.05E-10	1.42E-13	6.22E-08	5.33E-08	NA	NA	NA	NA	NA	NA	NA
141	1.08E-10	2.04E-10	4.94E-15	1.56E-10	6.17E-10	2.30E-09	1.29E-08	NA	NA	NA	NA	NA	NA	NA
142	1.35E-04	1.58E-05	5.28E-04	3.01E-05	6.26E-06	9.73E-06	1.65E-05	NA	NA	NA	NA	NA	NA	NA
143	1.12E-02	5.40E-16	5.72E-05	9.41E-08	2.99E-12	1.41E-12	3.77E-09	NA	NA	NA	NA	NA	NA	NA
144	1.68E-10	7.85E-11	6.88E-12	1.51E-19	1.80E-09	3.23E-06	2.72E-10	NA	NA	NA	NA	NA	NA	NA

145	1.57E-08	5.99E-01	4.17E-04	1.94E-17	1.69E-17	4.49E-08	2.27E-13	NA	NA	NA	NA	NA	NA	NA
146	5.96E-10	5.44E-07	1.34E-12	0.00E+00	9.19E-23	1.23E-19	7.99E-19	NA	NA	NA	NA	NA	NA	NA
147	2.72E-05	2.41E-02	5.98E-03	9.84E-24	6.21E-24	4.28E-11	1.14E-03	NA	NA	NA	NA	NA	NA	NA
148	1.59E-05	5.68E-02	6.26E-05	6.94E-03	1.03E-05	3.61E-04	7.97E-05	NA	NA	NA	NA	NA	NA	NA
149	1.84E-05	2.54E-05	3.54E-04	2.69E-01	1.10E-01	3.44E-02	2.72E-11	NA	NA	NA	NA	NA	NA	NA
150	6.50E-04	3.43E-05	3.72E-03	4.85E-05	6.08E-09	2.73E-09	2.05E-08	NA	NA	NA	NA	NA	NA	NA
151	6.29E-01	3.02E-03	3.06E-01	8.24E-10	2.82E-06	3.79E-08	7.45E-03	NA	NA	NA	NA	NA	NA	NA
152	4.36E-03	4.96E-02	8.56E-04	4.51E-04	1.14E-09	9.01E-11	8.56E-09	NA	NA	NA	NA	NA	NA	NA
153	1.21E-12	3.54E-05	6.44E-11	2.00E-15	9.98E-15	9.23E-14	6.92E-14	NA	1.55E-27	4.45E-30	3.39E-16	3.60E-30	3.75E-01	1.47E-01
154	1.10E-08	1.39E-04	1.76E-06	7.52E-08	5.66E-09	8.35E-07	1.22E-11	NA	NA	NA	NA	NA	NA	NA
155	1.27E-04	2.30E-02	2.04E-06	3.13E-06	3.81E-14	2.67E-05	1.53E-01	NA	NA	NA	NA	NA	NA	NA
156	1.67E-02	1.22E-03	7.56E-04	8.47E-20	2.35E-02	2.86E-08	3.91E-10	NA	NA	NA	NA	NA	NA	NA
157	2.41E-08	2.68E-04	7.88E-12	2.99E-16	2.16E-16	6.65E-22	3.71E-09	NA	NA	NA	NA	NA	NA	NA
158	3.93E-01	4.31E-03	7.95E-01	4.43E-02	6.32E-02	3.02E-03	1.41E-01	NA	3.68E-04	5.40E-02	4.41E-03	8.07E-02	3.25E-06	1.19E-06
159	1.29E-05	3.23E-01	1.10E-05	6.59E-05	6.20E-03	3.07E-03	1.52E-03	NA	6.95E-06	1.36E-06	9.53E-06	2.69E-06	5.22E-01	8.76E-04
160	1.49E-05	1.40E-01	6.00E-08	1.13E-03	1.09E-02	2.31E-04	1.54E-02	NA	NA	0.00E+00	NA	5.91E-19	NA	1.83E-02
161	2.58E-04	9.84E-02	5.41E-11	4.62E-07	1.49E-21	3.27E-19	1.26E-08	NA	NA	NA	NA	NA	NA	NA
162	8.74E-03	4.66E-01	1.33E-03	1.21E-16	9.72E-13	4.25E-14	4.74E-12	NA	NA	NA	NA	NA	NA	NA
163	2.96E-19	1.94E-04	9.30E-23	3.16E-18	4.43E-13	4.18E-11	4.14E-05	NA	NA	NA	NA	NA	NA	NA
164	2.72E-02	6.06E-01	9.47E-03	1.68E-07	6.62E-16	1.57E-18	1.75E-15	NA	NA	NA	NA	NA	NA	NA
165	9.90E-04	2.52E-03	1.97E-03	2.38E-24	1.19E-15	9.16E-06	1.16E-04	NA	NA	NA	NA	NA	NA	NA
166	2.72E-12	1.30E-13	1.80E-12	1.50E-14	3.87E-12	5.79E-11	2.24E-09	NA	NA	NA	NA	NA	NA	NA
167	3.95E-11	2.24E-07	1.07E-13	7.43E-06	2.10E-06	1.78E-06	2.94E-21	NA	1.12E-08	0.00E+00	9.13E-10	0.00E+00	2.06E-03	2.21E-02
168	1.22E-06	5.16E-02	5.30E-03	6.44E-25	1.11E-15	1.50E-10	3.58E-02	NA	1.36E-26	NA	9.57E-28	NA	1.81E-03	NA
169	1.59E-04	1.09E-01	6.50E-21	1.30E-09	3.50E-17	2.36E-12	1.74E-11	NA	1.12E-10	0.00E+00	2.32E-09	6.72E-30	2.23E-05	9.02E-01
170	9.69E-08	8.98E-02	7.10E-07	1.75E-09	1.48E-16	1.14E-14	2.07E-09	NA	NA	NA	NA	NA	NA	NA
171	9.16E-04	1.53E-02	3.04E-04	5.66E-05	4.78E-15	1.55E-10	1.94E-14	NA	1.10E-29	2.01E-10	7.97E-24	5.58E-13	8.85E-17	2.21E-23
172	3.76E-04	5.08E-04	5.49E-04	6.71E-06	2.09E-06	1.70E-11	5.51E-04	NA	0.00E+00	0.00E+00	0.00E+00	0.00E+00	7.74E-01	3.36E-01
173	7.28E-11	5.49E-02	2.02E-09	9.51E-28	9.60E-19	1.18E-14	3.83E-05	NA	NA	NA	NA	NA	NA	NA
174	7.94E-24	1.18E-07	7.56E-29	2.93E-17	8.29E-19	5.70E-21	2.84E-15	NA	4.13E-26	0.00E+00	0.00E+00	0.00E+00	6.13E-01	7.88E-25
175	7.40E-13	1.33E-20	7.05E-13	2.84E-16	6.94E-19	9.53E-14	1.40E-14	NA	NA	NA	NA	NA	NA	NA
176	6.64E-03	7.75E-04	8.55E-05	1.20E-10	1.68E-08	5.02E-15	1.34E-06	NA	NA	NA	NA	NA	NA	NA
177	1.22E-06	2.47E-02	1.09E-03	1.68E-16	5.32E-07	8.21E-07	3.85E-02	NA	NA	NA	NA	NA	NA	NA
178	3.65E-02	2.30E-07	2.02E-03	2.14E-03	3.11E-02	1.60E-02	9.13E-01	NA	NA	NA	NA	NA	NA	NA
179	3.07E-03	2.70E-09	2.72E-03	6.88E-10	3.33E-10	3.35E-06	1.91E-23	NA	NA	NA	NA	NA	NA	NA
180	2.57E-03	5.50E-06	6.40E-06	3.72E-16	2.85E-07	1.52E-14	8.44E-07	NA	NA	NA	NA	NA	NA	NA
181	4.43E-07	3.17E-01	1.27E-04	1.36E-13	2.27E-21	4.92E-21	8.30E-19	NA	NA	NA	NA	NA	NA	NA
182	7.80E-18	7.86E-04	1.16E-20	4.01E-25	7.47E-20	1.78E-23	5.78E-10	NA	NA	NA	NA	NA	NA	NA
183	2.70E-01	7.94E-06	9.60E-01	1.97E-19	9.37E-14	9.33E-15	2.82E-07	NA	NA	NA	NA	NA	NA	NA
184	2.08E-09	3.21E-08	1.52E-10	1.37E-21	6.28E-23	7.65E-20	2.97E-19	NA	NA	NA	NA	NA	NA	NA
185	2.43E-06	1.33E-07	2.26E-05	8.55E-10	7.48E-12	1.33E-06	7.97E-20	NA	NA	NA	NA	NA	NA	NA
186	1.15E-04	2.49E-01	8.20E-03	8.54E-03	1.01E-02	8.59E-03	8.48E-04	NA	NA	NA	NA	NA	NA	NA
187	3.23E-01	9.88E-01	1.72E-01	1.06E-03	2.06E-12	1.52E-13	2.13E-09	NA	NA	2.93E-20	NA	5.96E-20	NA	1.75E-01
188	2.27E-08	1.22E-04	4.49E-06	4.32E-08	2.60E-11	1.49E-08	2.40E-20	NA	8.08E-30	NA	5.35E-29	NA	2.76E-04	NA
189	2.16E-02	4.16E-02	1.94E-03	6.63E-18	7.97E-12	1.17E-08	1.15E-03	NA	1.49E-12	9.73E-15	5.74E-28	1.70E-07	4.24E-07	4.67E-04
190	1.96E-02	4.54E-08	8.52E-03	8.34E-13	3.96E-21	2.66E-14	1.45E-10	NA	NA	NA	NA	NA	NA	NA
191	1.02E-03	2.58E-01	1.82E-02	1.79E-23	1.74E-05	6.30E-04	8.57E-02	NA	2.36E-07	1.14E-21	4.63E-06	3.78E-22	1.88E-03	1.58E-01
192	4.42E-03	4.65E-01	1.04E-01	1.02E-15	6.80E-16	1.66E-06	2.08E-02	NA	1.12E-14	2.00E-11	1.75E-16	1.23E-13	9.63E-02	5.69E-02
193	1.30E-10	7.32E-09	2.42E-10	2.23E-13	9.89E-11	7.41E-06	1.43E-07	NA	1.33E-18	3.37E-20	6.21E-22	1.40E-28	2.12E-04	8.61E-01

194	9.98E-01	6.43E-01	9.77E-01	6.60E-08	1.26E-09	9.69E-08	2.46E-05	NA	NA	NA	NA	NA	NA	NA
195	9.86E-01	1.95E-02	9.15E-01	1.12E-06	1.19E-04	1.24E-03	3.39E-11	NA	1.38E-10	2.93E-30	1.40E-10	1.98E-24	6.40E-01	2.85E-02
196	8.19E-10	1.20E-15	1.07E-08	1.75E-06	2.71E-07	2.31E-04	3.92E-29	NA	NA	NA	NA	NA	NA	NA
197	8.05E-08	1.48E-10	6.85E-06	2.92E-05	2.25E-10	1.34E-05	2.90E-07	NA	NA	1.71E-11	NA	2.98E-11	6.51E-01	4.58E-04
198	9.43E-04	1.57E-01	6.55E-07	2.94E-21	3.38E-22	8.67E-22	1.46E-18	NA	1.63E-17	9.60E-25	3.61E-21	3.75E-23	5.48E-02	1.40E-04
199	2.80E-05	2.35E-11	1.02E-03	2.13E-07	1.18E-11	5.67E-12	6.56E-10	NA	1.78E-17	2.27E-05	4.71E-16	7.23E-19	3.35E-01	1.14E-02
200	6.29E-06	3.59E-11	7.40E-02	7.19E-02	8.19E-02	7.02E-03	7.28E-17	NA	5.31E-13	4.54E-09	6.04E-23	3.75E-24	3.64E-01	2.44E-02
201	6.68E-18	5.92E-15	8.28E-22	2.82E-18	1.24E-20	1.13E-13	5.12E-12	NA	1.29E-18	4.94E-30	8.20E-20	2.26E-30	6.21E-04	3.11E-05
202	1.50E-04	1.77E-04	8.97E-03	1.14E-08	1.01E-08	1.25E-08	2.51E-17	NA	0.00E+00	0.00E+00	1.32E-29	3.96E-30	9.93E-01	9.98E-01
203	1.32E-06	1.03E-08	1.98E-06	6.83E-19	1.14E-10	2.75E-19	8.93E-30	NA	1.64E-29	NA	4.45E-17	NA	4.23E-04	NA
204	1.80E-07	1.08E-01	3.22E-01	2.89E-08	2.69E-04	4.75E-04	3.05E-03	NA	1.29E-01	1.41E-02	3.67E-15	1.04E-13	3.46E-03	9.26E-02
205	4.86E-01	1.43E-01	6.75E-01	9.41E-07	1.57E-16	1.00E-02	2.76E-11	NA	1.11E-25	2.72E-19	2.15E-23	7.18E-19	1.36E-04	4.20E-03
206	5.14E-06	3.83E-12	4.16E-07	1.81E-21	1.65E-11	2.60E-17	2.98E-15	NA	1.34E-19	7.32E-28	1.00E-18	8.35E-18	3.65E-02	9.01E-04
207	1.96E-03	2.90E-03	4.05E-03	2.33E-06	1.78E-16	4.91E-17	1.50E-06	NA	2.44E-09	2.83E-30	1.06E-27	0.00E+00	4.85E-02	1.53E-01
208	1.30E-09	2.41E-10	9.28E-17	7.34E-12	8.46E-12	1.95E-08	8.10E-27	NA	0.00E+00	0.00E+00	0.00E+00	0.00E+00	5.27E-04	7.79E-04
209	1.60E-11	3.52E-09	6.07E-11	3.02E-29	2.05E-19	3.30E-12	2.92E-10	NA	0.00E+00	0.00E+00	0.00E+00	0.00E+00	1.25E-04	4.32E-03
210	1.35E-10	8.97E-13	1.66E-10	3.36E-11	1.37E-20	1.66E-12	3.61E-14	NA	8.43E-27	1.16E-15	2.45E-30	1.93E-26	1.27E-06	5.50E-06
211	5.05E-12	1.31E-12	1.62E-13	1.55E-27	2.90E-26	4.61E-24	5.67E-16	NA	9.03E-21	5.27E-29	0.00E+00	3.38E-21	1.87E-02	2.15E-02
212	3.56E-19	1.43E-03	2.73E-17	1.55E-19	8.98E-14	6.08E-12	2.50E-20	NA	5.38E-19	4.72E-17	2.97E-20	9.26E-14	2.31E-04	1.83E-02
213	3.42E-09	1.21E-10	9.79E-10	5.13E-17	1.10E-12	7.04E-14	2.45E-27	NA	1.45E-09	4.06E-28	1.48E-10	0.00E+00	8.75E-03	6.76E-02
214	2.82E-06	1.29E-07	3.78E-06	4.60E-09	1.05E-23	5.75E-12	3.42E-10	NA	4.93E-29	1.56E-28	2.06E-30	1.65E-18	4.41E-03	7.30E-03
215	7.47E-06	6.61E-04	1.40E-05	6.84E-17	1.46E-19	2.28E-16	1.21E-15	NA	1.54E-20	1.87E-27	2.25E-19	1.47E-15	6.97E-08	1.04E-01
216	5.56E-07	3.35E-02	1.09E-02	7.88E-07	3.82E-08	3.87E-11	4.49E-10	NA	1.20E-10	1.07E-07	2.63E-10	3.50E-10	2.26E-01	1.17E-01
217	2.04E-02	3.34E-09	2.24E-04	1.99E-12	1.36E-08	7.27E-09	3.49E-06	NA	1.04E-26	3.77E-17	5.07E-27	1.96E-15	1.17E-01	2.21E-01
218	3.32E-02	8.50E-05	2.79E-01	4.77E-11	2.19E-11	2.51E-14	7.43E-27	NA	4.58E-20	4.46E-15	6.30E-12	5.49E-21	9.34E-01	6.77E-01
219	3.56E-02	1.05E-04	2.91E-02	4.45E-08	9.01E-15	1.30E-15	1.55E-15	NA	5.40E-25	3.35E-16	0.00E+00	7.39E-23	4.54E-01	5.50E-01
220	7.34E-01	6.28E-01	1.33E-01	3.70E-07	1.01E-12	9.07E-07	3.20E-07	NA	NA	NA	NA	NA	NA	NA
221	5.83E-11	4.48E-07	4.43E-15	2.14E-30	1.49E-11	1.49E-11	3.51E-11	NA	3.50E-30	1.53E-22	7.68E-30	3.49E-19	1.20E-01	4.30E-03
222	5.25E-06	5.82E-12	5.79E-11	4.15E-15	5.06E-11	2.44E-17	2.89E-18	NA	7.26E-12	6.36E-13	2.50E-12	1.60E-14	1.92E-01	4.95E-02
223	1.01E-08	3.27E-01	1.02E-06	2.15E-11	6.15E-16	6.09E-17	3.11E-09	NA	1.19E-17	1.09E-25	3.93E-19	1.22E-27	4.54E-01	2.65E-02
224	2.27E-03	6.40E-01	4.19E-05	1.57E-13	2.17E-13	1.35E-12	1.15E-06	NA	8.21E-24	6.58E-16	1.88E-24	2.81E-12	3.93E-01	6.29E-01
225	6.54E-10	2.01E-02	4.54E-08	5.12E-25	1.70E-21	1.86E-18	4.72E-06	NA	3.23E-26	2.26E-30	8.91E-26	2.51E-30	2.79E-05	2.23E-04
226	1.37E-01	4.62E-02	1.28E-01	6.63E-05	1.10E-06	4.43E-12	1.10E-09	NA	2.42E-21	1.90E-10	3.52E-12	3.76E-13	9.64E-01	9.28E-01
227	4.47E-10	2.21E-05	7.46E-08	3.39E-09	3.89E-07	3.48E-07	7.52E-05	NA	1.25E-06	3.07E-06	2.68E-05	1.69E-05	1.27E-02	9.80E-03
228	1.54E-02	1.69E-02	1.07E-02	8.89E-05	1.54E-03	5.75E-04	2.95E-02	NA	7.04E-06	8.20E-05	2.40E-12	7.04E-03	4.80E-01	7.95E-01
229	4.54E-08	1.92E-07	1.26E-07	1.05E-13	1.28E-12	2.13E-12	8.28E-25	NA	0.00E+00	1.79E-20	3.48E-25	4.63E-23	1.46E-03	1.37E-01
230	1.72E-08	2.49E-03	3.27E-07	9.12E-04	1.25E-11	2.89E-13	2.58E-03	NA	3.25E-19	2.33E-30	2.08E-21	0.00E+00	7.37E-22	1.56E-01
231	5.11E-04	8.28E-02	2.40E-03	7.24E-14	3.46E-15	1.40E-15	1.29E-02	NA	4.43E-26	7.89E-28	2.18E-26	4.63E-28	1.56E-05	2.13E-06
232	7.79E-16	6.65E-13	2.85E-16	7.99E-25	1.32E-11	2.27E-10	1.66E-29	NA	1.62E-19	1.25E-21	1.26E-21	2.79E-26	2.87E-09	5.01E-04
233	1.04E-07	1.54E-05	1.54E-07	3.93E-08	6.62E-13	2.89E-13	5.09E-11	NA	8.29E-28	NA	1.20E-27	NA	1.10E-02	NA
234	5.17E-05	7.18E-02	1.47E-04	1.84E-03	3.62E-10	2.41E-03	1.96E-12	7.35E-01	2.41E-13	2.73E-06	1.70E-14	7.40E-07	1.70E-03	4.22E-01
235	2.82E-02	3.85E-02	6.13E-03	3.15E-21	8.67E-07	1.35E-16	8.60E-20	2.81E-01	NA	NA	NA	NA	NA	NA
236	2.85E-24	2.03E-04	2.94E-10	2.67E-17	2.33E-04	2.17E-03	2.81E-15	2.11E-03	7.14E-21	8.77E-21	2.81E-22	4.48E-22	8.62E-05	1.46E-02
237	4.04E-08	2.45E-02	3.28E-09	9.58E-10	2.24E-03	1.13E-02	2.25E-03	NA	1.45E-20	NA	1.08E-20	NA	2.53E-01	NA
238	6.48E-12	2.42E-04	9.02E-13	2.36E-30	9.92E-21	3.61E-22	1.31E-08	NA	0.00E+00	0.00E+00	0.00E+00	0.00E+00	2.67E-01	1.43E-02
239	7.88E-05	2.63E-05	6.68E-06	4.79E-16	2.39E-15	6.61E-13	3.55E-07	1.71E-02	9.23E-20	2.66E-22	7.40E-09	5.46E-28	8.29E-11	3.08E-03
240	5.73E-24	1.11E-03	0.00E+00	6.00E-20	8.97E-11	5.16E-07	3.93E-08	NA	1.68E-17	2.03E-30	0.00E+00	6.17E-30	6.73E-01	3.80E-01
241	5.28E-06	3.72E-12	1.45E-04	0.00E+00	6.52E-30	7.67E-26	1.32E-29	3.60E-06	2.59E-30	2.84E-30	0.00E+00	0.00E+00	1.93E-04	1.14E-03
242	2.81E-01	5.06E-01	4.82E-03	5.09E-12	1.32E-03	1.66E-02	1.11E-04	NA	5.64E-14	3.42E-16	3.71E-05	4.56E-11	1.08E-01	4.58E-03

243	3.65E-09	4.83E-23	4.45E-11	1.82E-22	2.45E-20	2.77E-19	0.00E+00	NA	4.43E-29	1.16E-22	3.63E-29	5.50E-23	1.10E-04	2.69E-04
244	1.08E-03	1.34E-01	5.13E-03	4.66E-07	6.45E-20	1.98E-06	2.55E-06	NA	1.10E-20	5.65E-19	2.78E-25	3.94E-19	1.12E-01	1.77E-04
245	2.22E-02	9.86E-12	4.47E-02	3.71E-13	3.14E-13	1.49E-11	9.38E-20	4.14E-04	2.30E-08	0.00E+00	1.32E-27	0.00E+00	1.19E-01	9.44E-07
246	2.91E-08	1.13E-16	8.97E-05	1.10E-12	2.61E-23	9.20E-14	7.44E-08	NA	7.20E-05	4.40E-06	1.40E-10	9.47E-16	2.42E-02	1.56E-03
247	2.78E-02	1.03E-13	1.63E-02	7.19E-26	2.53E-10	7.51E-04	4.29E-05	2.41E-01	2.69E-24	NA	1.25E-17	NA	2.29E-03	NA
248	2.41E-13	1.35E-06	8.75E-10	6.77E-28	1.09E-27	8.35E-18	0.00E+00	NA	0.00E+00	2.03E-30	0.00E+00	0.00E+00	4.17E-04	2.50E-03
249	1.77E-16	6.48E-19	1.43E-13	1.03E-22	4.99E-13	1.93E-10	3.60E-21	8.55E-01	8.95E-23	1.30E-21	4.13E-19	2.21E-19	7.16E-04	1.37E-01
250	9.74E-03	2.01E-05	1.28E-11	1.25E-13	7.51E-10	5.04E-09	1.73E-19	2.07E-02	3.24E-10	4.53E-09	2.44E-26	1.20E-27	4.80E-03	3.64E-07
251	2.09E-24	4.57E-01	3.90E-16	1.52E-11	6.53E-11	1.28E-05	1.68E-19	NA	NA	2.88E-24	NA	1.00E-16	NA	4.02E-01
252	1.71E-03	1.27E-08	2.93E-05	0.00E+00	3.94E-21	7.98E-20	2.43E-18	5.60E-07	1.69E-24	6.74E-16	2.09E-30	6.54E-15	5.21E-04	3.41E-03
253	1.03E-07	9.89E-11	2.88E-08	2.29E-24	1.23E-23	3.16E-11	0.00E+00	8.27E-07	2.79E-27	0.00E+00	1.16E-26	0.00E+00	9.29E-03	2.07E-02
254	5.15E-03	4.31E-01	9.40E-02	9.56E-20	2.38E-18	1.08E-08	9.08E-04	NA	1.37E-13	4.32E-23	1.91E-13	2.22E-14	4.07E-01	1.80E-03
255	2.71E-07	1.50E-02	9.11E-07	0.00E+00	6.60E-25	1.41E-09	5.58E-12	NA	8.60E-28	NA	1.42E-28	NA	7.16E-02	NA
256	5.00E-18	4.52E-02	1.34E-17	2.56E-24	3.51E-28	1.23E-28	8.01E-06	NA	7.90E-22	1.18E-25	1.76E-11	8.12E-27	1.10E-03	2.33E-01
257	2.28E-14	1.25E-10	4.26E-20	1.96E-18	1.67E-12	2.02E-24	6.30E-23	NA	2.08E-23	2.41E-26	3.25E-21	2.18E-29	2.60E-05	5.11E-01
258	8.84E-10	4.00E-13	6.79E-11	6.75E-17	4.97E-14	5.77E-12	3.03E-12	NA	2.15E-14	6.60E-09	0.00E+00	1.36E-17	2.52E-05	7.00E-08
259	1.04E-15	1.54E-02	2.48E-18	1.60E-07	5.00E-07	5.61E-07	5.75E-12	NA	0.00E+00	0.00E+00	0.00E+00	0.00E+00	3.86E-06	7.52E-08
260	1.29E-23	1.76E-27	2.25E-23	2.79E-30	1.05E-29	3.08E-28	4.83E-30	7.15E-01	4.86E-29	2.69E-22	4.79E-20	2.06E-19	1.45E-08	5.86E-09
261	7.16E-13	2.09E-05	9.55E-12	1.95E-17	3.07E-17	1.05E-14	6.84E-10	NA	4.08E-26	3.08E-13	8.45E-26	7.45E-16	1.87E-01	1.28E-03
262	4.21E-10	1.10E-04	4.68E-17	2.40E-16	1.85E-20	2.62E-10	3.67E-09	NA	8.56E-02	1.81E-21	1.95E-06	1.98E-25	9.84E-01	1.99E-01
263	4.47E-08	1.43E-19	6.55E-11	9.42E-11	7.58E-09	4.81E-08	7.86E-10	1.81E-02	1.60E-15	1.74E-19	1.07E-29	2.56E-27	4.85E-03	1.05E-02
264	6.74E-18	9.94E-09	2.48E-29	1.10E-10	2.85E-26	2.85E-13	1.21E-15	5.31E-03	2.35E-16	3.93E-13	4.01E-26	1.37E-24	1.05E-01	1.42E-01
265	1.49E-18	2.04E-23	1.77E-14	6.15E-24	5.79E-23	1.60E-20	3.04E-28	2.57E-07	1.75E-27	3.03E-20	8.66E-22	3.42E-22	4.93E-06	3.74E-06
266	1.53E-09	3.95E-01	1.48E-09	4.30E-22	8.12E-13	2.65E-22	1.07E-19	NA	4.26E-18	NA	2.75E-18	NA	1.41E-05	NA
267	1.84E-07	5.96E-06	3.06E-14	3.25E-25	1.48E-14	6.65E-16	2.67E-30	NA	4.66E-19	0.00E+00	2.91E-21	0.00E+00	6.27E-05	3.90E-02
268	2.77E-04	3.20E-02	1.80E-02	5.97E-06	5.20E-06	3.30E-04	4.92E-06	NA	4.40E-01	5.29E-01	1.22E-19	2.45E-28	5.69E-03	1.88E-03
269	1.00E-09	1.15E-02	8.34E-11	2.25E-15	1.06E-18	6.29E-18	1.28E-10	NA	2.26E-30	3.00E-30	5.58E-29	2.11E-30	9.15E-02	3.16E-02
270	1.80E-10	7.08E-05	3.40E-09	0.00E+00	6.47E-14	1.18E-21	4.37E-17	NA	6.14E-29	2.41E-30	0.00E+00	0.00E+00	1.11E-01	3.97E-01
271	1.04E-12	2.16E-05	7.87E-14	6.41E-19	1.24E-08	3.00E-15	2.29E-20	NA	2.50E-28	3.37E-28	7.93E-29	2.57E-28	2.55E-02	1.98E-04
272	5.66E-05	6.84E-14	6.00E-08	4.83E-14	1.00E-13	6.98E-09	2.21E-09	NA	6.31E-22	5.63E-30	9.64E-28	0.00E+00	2.63E-02	2.80E-06
273	4.53E-07	4.81E-01	1.21E-02	6.16E-14	2.33E-16	9.14E-12	5.58E-02	NA	8.44E-19	5.19E-23	6.28E-19	1.48E-16	9.43E-01	2.81E-05
274	1.44E-11	3.50E-02	1.24E-09	2.12E-12	8.40E-05	9.85E-07	7.55E-05	NA	2.12E-30	5.20E-23	8.99E-29	8.83E-24	1.27E-03	1.26E-01
275	5.71E-06	1.74E-28	9.64E-06	3.23E-11	1.52E-16	1.61E-08	3.24E-19	6.99E-05	2.50E-25	0.00E+00	1.37E-26	0.00E+00	3.51E-12	5.18E-06
276	6.38E-13	1.49E-04	6.31E-12	4.23E-27	9.77E-21	2.20E-17	4.25E-08	NA	4.81E-14	0.00E+00	1.54E-15	4.10E-17	1.80E-06	6.40E-05
277	1.37E-03	1.17E-02	8.77E-10	7.08E-20	2.78E-07	1.92E-09	7.83E-05	NA	5.17E-16	3.56E-15	5.71E-25	2.33E-30	5.67E-05	3.47E-05
278	1.67E-09	1.46E-06	8.19E-10	3.89E-12	2.58E-09	8.85E-10	2.53E-14	NA	0.00E+00	1.66E-23	0.00E+00	2.71E-26	6.51E-01	2.53E-03
279	5.69E-15	4.35E-01	4.08E-10	3.68E-19	1.88E-14	2.94E-11	1.04E-08	NA	8.07E-28	1.47E-29	3.60E-28	8.63E-30	5.90E-04	2.78E-03
280	5.09E-16	5.32E-05	5.60E-13	2.66E-30	3.03E-25	5.76E-18	1.28E-23	NA	3.53E-22	1.52E-24	3.01E-25	2.90E-30	1.98E-01	1.41E-01
281	1.40E-01	1.09E-05	2.28E-01	2.21E-03	2.95E-04	2.10E-04	1.04E-03	NA	7.85E-06	1.27E-06	1.09E-05	5.58E-07	1.86E-02	1.63E-01
282	3.01E-22	4.13E-06	2.39E-21	1.75E-12	6.92E-11	2.06E-10	0.00E+00	NA	5.15E-10	6.39E-11	1.77E-17	0.00E+00	1.96E-01	1.35E-02
283	2.85E-07	1.67E-09	1.05E-10	7.39E-11	7.48E-12	3.90E-06	6.86E-28	NA	NA	NA	NA	NA	NA	NA
284	1.29E-05	7.93E-02	9.73E-04	2.81E-03	3.37E-02	2.23E-02	7.04E-12	NA	NA	NA	NA	NA	NA	NA
285	5.19E-08	9.15E-01	1.02E-10	8.09E-13	1.81E-11	3.15E-12	1.93E-26	NA	7.02E-21	4.30E-24	1.90E-24	0.00E+00	1.31E-02	2.77E-03
286	1.00E-29	2.88E-19	4.82E-18	2.76E-18	3.11E-12	2.89E-10	4.45E-11	NA	2.32E-10	4.80E-27	2.44E-21	1.35E-25	1.34E-02	1.64E-11
287	5.69E-12	5.84E-22	4.69E-12	1.25E-12	4.93E-13	5.04E-12	6.81E-08	NA	0.00E+00	0.00E+00	1.03E-25	0.00E+00	1.00E-14	1.29E-13
288	4.44E-21	2.87E-05	1.96E-20	9.71E-18	8.12E-23	1.95E-28	9.94E-14	NA	1.30E-22	0.00E+00	9.44E-26	2.44E-30	1.83E-18	9.10E-09
289	3.01E-09	2.59E-11	5.65E-11	3.77E-06	1.05E-05	2.23E-04	3.02E-09	NA	0.00E+00	9.40E-20	0.00E+00	2.03E-24	1.30E-10	1.52E-15
290	3.17E-07	3.55E-02	7.44E-08	5.43E-25	8.95E-26	6.61E-26	6.27E-12	NA	1.16E-05	1.48E-28	3.63E-06	5.50E-30	4.69E-11	9.86E-11
291	1.53E-12	1.14E-10	6.82E-13	1.79E-06	1.03E-13	1.12E-11	1.29E-28	NA	3.72E-20	1.21E-27	1.70E-29	1.10E-28	4.12E-07	6.32E-07

292	3.22E-07	5.91E-10	6.90E-07	8.29E-08	4.29E-10	1.62E-13	1.36E-10	NA	NA	1.18E-21	NA	1.69E-29	NA	5.05E-07
293	8.73E-12	1.43E-02	6.64E-10	1.72E-13	2.33E-09	1.18E-05	5.23E-10	NA	4.45E-24	3.74E-16	1.57E-26	9.36E-25	7.08E-07	1.42E-03
294	4.19E-07	8.22E-12	9.77E-05	4.47E-05	1.30E-06	3.43E-03	7.11E-11	NA	5.81E-23	3.31E-22	9.86E-23	2.93E-25	2.71E-07	1.36E-09
295	3.70E-11	1.46E-18	2.05E-10	6.79E-15	7.71E-22	1.69E-14	5.37E-30	NA	0.00E+00	0.00E+00	0.00E+00	0.00E+00	5.00E-05	1.41E-05
296	2.84E-12	1.02E-22	1.02E-15	5.13E-17	1.08E-15	7.07E-19	0.00E+00	NA	5.60E-25	3.83E-20	4.47E-30	2.13E-24	6.56E-10	1.54E-03
297	7.74E-15	1.39E-13	7.08E-17	4.90E-20	2.31E-29	1.64E-28	5.19E-30	NA	4.63E-21	6.95E-13	7.76E-15	6.34E-14	5.68E-03	1.25E-01
298	3.27E-15	1.06E-09	1.72E-13	3.52E-14	1.33E-13	6.48E-09	7.32E-27	NA	0.00E+00	4.25E-13	3.63E-30	5.75E-13	2.34E-07	6.46E-05
299	1.24E-06	1.60E-07	1.76E-05	1.84E-06	2.61E-07	6.65E-05	5.17E-04	NA	7.10E-25	NA	2.31E-19	NA	8.35E-09	NA
300	5.77E-03	1.51E-04	1.01E-02	1.35E-15	3.02E-05	1.53E-10	1.31E-15	NA	NA	NA	NA	NA	NA	NA
301	1.17E-21	4.83E-12	5.16E-20	1.53E-10	2.79E-09	4.70E-07	6.45E-13	NA	NA	NA	NA	NA	NA	NA
302	2.09E-23	2.47E-08	1.77E-26	2.45E-20	2.29E-19	4.50E-21	3.70E-11	NA	NA	NA	NA	NA	NA	NA
303	1.24E-06	7.25E-08	8.22E-13	2.59E-14	1.11E-15	1.60E-18	9.75E-19	NA	NA	NA	NA	NA	NA	NA
304	1.07E-02	9.58E-06	2.69E-06	1.15E-17	1.37E-26	2.06E-26	1.59E-12	NA	NA	NA	NA	NA	NA	NA
305	9.14E-15	2.93E-04	4.50E-17	1.44E-26	1.12E-27	2.61E-17	1.31E-06	9.55E-06	NA	NA	NA	NA	NA	NA
306	1.20E-14	2.73E-02	1.97E-07	2.72E-14	3.76E-22	1.94E-20	8.10E-16	1.36E-07	NA	NA	NA	NA	NA	NA
307	6.89E-09	7.96E-15	1.49E-12	6.76E-10	4.75E-12	1.35E-11	4.01E-18	1.76E-10	NA	NA	NA	NA	NA	NA
308	1.45E-09	6.97E-06	2.07E-04	7.56E-21	1.53E-24	5.42E-13	2.45E-11	7.16E-01	NA	NA	NA	NA	NA	NA
309	2.19E-01	1.89E-01	3.53E-01	3.40E-04	1.31E-05	7.29E-09	1.70E-13	7.16E-01	NA	NA	NA	NA	NA	NA
310	1.33E-04	3.32E-01	2.82E-01	2.24E-04	5.42E-08	3.92E-11	9.42E-03	1.92E-06	NA	NA	NA	NA	NA	NA
311	6.52E-03	2.28E-01	1.63E-01	1.75E-04	3.64E-12	5.72E-12	1.05E-04	4.31E-01	NA	NA	NA	NA	NA	NA
312	1.28E-11	9.78E-15	9.59E-15	2.91E-10	7.21E-08	1.08E-05	5.76E-13	1.70E-03	NA	NA	NA	NA	NA	NA
313	6.33E-01	8.82E-09	9.60E-01	3.57E-06	1.06E-24	2.36E-08	1.19E-07	4.52E-02	NA	NA	NA	NA	NA	NA
314	1.18E-14	1.76E-08	6.10E-07	2.07E-15	1.34E-09	7.40E-12	1.27E-10	3.32E-08	NA	NA	NA	NA	NA	NA
315	5.80E-04	8.67E-04	1.44E-05	2.19E-27	2.66E-14	8.75E-11	1.04E-17	NA	NA	NA	NA	NA	NA	NA
316	8.79E-09	1.44E-17	4.93E-06	3.30E-06	1.15E-08	9.94E-11	1.25E-11	1.03E-05	NA	NA	NA	NA	NA	NA
317	6.90E-17	1.28E-14	1.77E-18	1.46E-20	0.00E+00	2.08E-21	3.05E-19	5.81E-14	NA	NA	NA	NA	NA	NA
318	1.92E-08	2.05E-08	9.45E-10	1.75E-16	3.80E-17	3.43E-20	2.07E-27	8.67E-12	NA	NA	NA	NA	NA	NA
319	7.33E-17	4.22E-17	2.50E-23	3.49E-30	1.65E-25	1.23E-20	1.28E-26	8.65E-02	NA	NA	NA	NA	NA	NA
320	1.39E-06	1.56E-14	6.38E-08	1.09E-14	5.25E-28	9.94E-21	5.44E-08	3.95E-03	NA	NA	NA	NA	NA	NA
321	9.73E-04	2.95E-05	9.99E-06	3.83E-10	1.71E-08	2.49E-07	2.39E-03	9.78E-05	NA	NA	NA	NA	NA	NA
322	5.44E-05	3.76E-09	8.85E-06	2.97E-07	9.50E-10	2.66E-07	3.62E-09	7.07E-03	NA	NA	NA	NA	NA	NA
323	1.22E-06	1.44E-28	8.92E-22	1.95E-12	7.97E-15	5.07E-08	6.61E-20	1.77E-17	NA	NA	NA	NA	NA	NA
324	2.07E-06	1.47E-17	1.19E-07	3.47E-29	4.61E-23	4.49E-24	9.71E-17	5.78E-04	NA	NA	NA	NA	NA	NA
325	2.33E-12	2.50E-01	1.22E-05	2.32E-10	9.83E-04	3.20E-03	2.03E-05	2.81E-18	NA	NA	NA	NA	NA	NA
326	9.54E-12	1.03E-04	1.78E-12	1.47E-08	1.36E-14	4.56E-09	1.10E-15	4.05E-04	NA	NA	NA	NA	NA	NA
327	4.90E-08	1.27E-22	1.47E-18	1.46E-14	2.16E-14	2.98E-09	5.96E-06	3.13E-10	NA	NA	NA	NA	NA	NA
328	4.16E-06	5.42E-03	9.98E-07	5.81E-09	3.13E-25	4.89E-09	1.71E-04	6.94E-02	NA	NA	NA	NA	NA	NA
329	3.28E-01	5.98E-01	5.83E-03	9.38E-03	1.50E-04	3.03E-04	3.39E-04	6.32E-01	NA	NA	NA	NA	NA	NA
330	1.89E-11	7.42E-13	7.46E-16	6.81E-14	5.72E-12	4.81E-05	7.30E-04	1.02E-20	NA	NA	NA	NA	NA	NA
331	2.79E-15	2.15E-14	2.73E-18	1.36E-09	3.06E-08	6.68E-07	1.44E-22	1.02E-20	NA	NA	NA	NA	NA	NA
332	2.23E-02	1.37E-02	4.56E-04	4.75E-10	2.98E-12	3.71E-07	4.20E-13	9.17E-03	NA	NA	NA	NA	NA	NA
333	5.72E-04	3.60E-07	2.60E-04	7.33E-05	5.47E-06	7.53E-08	1.50E-06	1.09E-02	NA	NA	NA	NA	NA	NA
334	5.37E-07	1.35E-14	2.30E-09	1.36E-29	2.07E-29	3.21E-25	2.76E-09	8.37E-05	NA	NA	NA	NA	NA	NA
335	6.94E-04	2.20E-01	1.05E-10	1.58E-18	7.70E-09	5.43E-06	1.06E-19	1.86E-05	NA	NA	NA	NA	NA	NA
336	8.91E-02	7.18E-03	6.34E-04	1.06E-18	5.32E-03	1.24E-10	2.46E-01	3.54E-03	NA	NA	NA	NA	NA	NA
337	2.94E-08	1.62E-07	1.07E-09	5.22E-08	1.56E-09	9.07E-08	7.88E-20	7.59E-06	NA	NA	NA	NA	NA	NA
338	1.71E-06	2.42E-03	1.15E-04	2.58E-30	1.73E-28	1.85E-08	3.58E-06	7.89E-05	1.15E-27	3.49E-27	NA	NA	2.11E-03	5.98E-07
339	8.53E-05	1.74E-01	1.53E-04	1.20E-04	1.52E-05	1.93E-03	3.93E-24	1.06E-01	1.56E-27	0.00E+00	NA	NA	1.02E-01	3.30E-03
340	8.39E-11	5.64E-12	1.14E-09	1.64E-18	1.79E-19	1.20E-16	2.14E-16	8.74E-08	NA	NA	NA	NA	NA	NA

341	1.95E-15	6.21E-08	3.55E-21	5.44E-16	8.25E-13	3.60E-12	5.18E-13	5.87E-05	NA	NA	NA	NA	NA	NA
342	4.57E-08	1.35E-05	3.35E-09	6.35E-10	1.32E-13	1.95E-17	5.91E-13	NA	NA	NA	NA	NA	NA	NA
343	7.62E-01	1.11E-02	1.47E-04	5.59E-02	1.67E-01	1.91E-03	2.64E-06	NA	NA	NA	NA	NA	NA	NA
344	7.04E-08	3.00E-06	4.77E-07	2.39E-07	1.77E-06	7.40E-04	1.29E-03	NA	NA	NA	NA	NA	NA	NA
345	2.04E-29	3.34E-19	9.93E-29	5.67E-23	2.19E-16	8.84E-16	3.52E-27	2.81E-17	NA	NA	NA	NA	NA	NA
346	5.74E-14	6.61E-09	7.96E-07	2.31E-15	3.99E-11	1.99E-27	1.08E-23	7.80E-05	NA	NA	NA	NA	NA	NA
347	4.79E-08	5.02E-08	2.16E-09	1.16E-12	1.14E-11	1.51E-10	1.36E-12	8.75E-05	NA	NA	NA	NA	NA	NA
348	5.20E-05	2.59E-10	2.33E-06	6.34E-11	1.11E-20	2.32E-17	4.05E-18	1.67E-08	NA	NA	NA	NA	NA	NA
349	3.96E-03	5.10E-07	4.61E-03	4.92E-05	1.17E-06	1.38E-03	5.29E-11	1.27E-02	NA	NA	NA	NA	NA	NA
350	8.30E-12	4.84E-05	2.76E-08	8.64E-19	3.47E-19	4.38E-16	1.05E-12	1.01E-10	NA	NA	NA	NA	NA	NA
351	1.42E-05	6.56E-09	3.32E-12	2.38E-14	9.01E-23	7.22E-07	1.66E-07	2.06E-04	NA	NA	NA	NA	NA	NA
352	1.84E-11	5.78E-06	2.14E-10	5.97E-19	6.60E-27	6.60E-26	3.75E-16	1.56E-06	NA	NA	NA	NA	NA	NA
353	1.21E-05	7.43E-13	5.34E-06	5.15E-09	2.57E-16	4.60E-07	1.96E-23	3.78E-06	NA	NA	NA	NA	NA	NA
354	1.08E-20	3.35E-17	1.73E-15	1.94E-29	2.17E-30	2.39E-16	4.03E-10	1.49E-07	NA	NA	NA	NA	NA	NA
355	2.23E-03	2.27E-03	5.67E-02	7.86E-08	4.31E-12	8.35E-09	9.53E-06	1.51E-03	NA	NA	NA	NA	NA	NA
356	1.86E-07	6.89E-02	1.17E-06	5.90E-11	3.70E-06	1.68E-10	4.23E-08	9.27E-03	NA	NA	NA	NA	NA	NA
357	5.02E-14	6.20E-11	1.46E-15	2.83E-24	2.63E-11	1.55E-27	4.64E-29	2.42E-06	NA	NA	NA	NA	NA	NA
358	3.14E-10	2.13E-20	8.00E-13	3.87E-10	1.30E-12	6.41E-10	2.62E-15	2.34E-01	NA	NA	NA	NA	NA	NA
359	2.82E-17	8.98E-12	1.64E-13	1.62E-21	9.54E-26	6.32E-20	7.90E-16	3.39E-13	NA	NA	NA	NA	NA	NA
360	4.41E-10	1.31E-21	4.63E-13	1.12E-05	8.87E-06	2.88E-29	1.22E-20	1.82E-08	NA	NA	NA	NA	NA	NA
361	4.36E-07	1.65E-09	4.99E-03	8.67E-18	5.34E-16	2.41E-13	5.42E-26	4.26E-10	7.66E-30	9.57E-28	NA	NA	1.36E-12	8.60E-11
362	3.63E-09	4.89E-02	2.98E-12	1.07E-13	5.76E-13	3.35E-10	2.64E-20	NA	NA	NA	NA	NA	NA	NA
363	1.67E-04	2.31E-03	5.28E-01	1.96E-05	1.03E-08	1.36E-03	1.23E-06	3.34E-04	NA	NA	NA	NA	NA	NA
364	5.80E-13	8.21E-12	1.81E-07	8.77E-19	2.26E-18	3.30E-14	1.15E-20	2.11E-07	NA	NA	NA	NA	NA	NA
365	1.10E-14	3.40E-09	8.27E-13	1.14E-07	9.69E-16	1.82E-08	1.05E-07	9.41E-12	NA	NA	NA	NA	NA	NA
366	1.95E-01	1.14E-06	6.77E-02	2.56E-12	1.28E-18	5.23E-22	1.44E-09	6.90E-03	0.00E+00	3.76E-21	NA	NA	1.72E-05	8.94E-07
367	9.64E-10	1.26E-15	1.14E-09	6.56E-27	4.35E-30	2.75E-29	3.68E-15	NA	NA	NA	NA	NA	NA	NA
368	1.96E-19	7.63E-05	1.86E-09	5.85E-11	8.30E-12	1.74E-05	5.12E-04	1.25E-03	NA	NA	NA	NA	NA	NA
369	1.88E-06	9.31E-09	3.07E-05	1.40E-16	1.17E-09	2.19E-11	1.32E-06	8.54E-02	NA	NA	NA	NA	NA	NA
370	1.30E-05	8.79E-06	1.15E-05	1.67E-02	9.85E-06	1.43E-01	1.77E-04	2.94E-05	NA	NA	NA	NA	NA	NA
371	1.42E-08	2.00E-10	2.85E-06	1.67E-12	4.24E-08	1.43E-03	9.96E-14	1.39E-04	NA	NA	NA	NA	NA	NA
372	2.84E-10	1.54E-16	2.60E-15	2.88E-14	9.97E-13	1.62E-18	2.97E-15	5.49E-02	NA	NA	NA	NA	NA	NA
373	5.39E-10	2.70E-09	2.07E-11	2.28E-16	8.48E-23	6.59E-16	9.92E-29	5.97E-06	NA	NA	NA	NA	NA	NA
374	3.09E-11	1.32E-04	2.46E-09	1.23E-07	1.35E-06	1.78E-05	2.14E-17	4.64E-05	NA	NA	NA	NA	NA	NA
375	1.18E-12	2.51E-17	4.07E-17	0.00E+00	1.45E-28	2.17E-29	2.67E-21	NA	NA	NA	NA	NA	NA	NA
376	6.71E-13	4.96E-08	1.34E-10	1.22E-19	7.95E-21	6.55E-18	2.03E-16	1.66E-12	NA	NA	NA	NA	NA	NA
**p-values of KPSS test on original data**														
1	0.01	0.10	0.01	0.01	0.01	0.01	0.02	NA	NA	NA	NA	NA	NA	NA
2	0.04	0.01	0.01	0.01	0.01	0.01	0.01	NA	NA	NA	NA	NA	NA	NA
3	0.05	0.02	0.05	0.05	0.08	0.10	0.07	NA	NA	NA	NA	NA	NA	NA
4	0.10	0.01	0.01	0.01	0.02	0.10	0.01	0.01	0.01	0.08	0.03	0.05	0.01	0.01
5	0.01	0.01	0.01	0.10	0.01	0.01	0.01	NA	NA	NA	NA	NA	NA	NA
6	0.01	0.01	0.01	0.01	0.01	0.01	0.01	NA	NA	NA	NA	NA	NA	NA
7	0.01	0.01	0.01	0.10	0.01	0.01	0.01	NA	NA	NA	NA	NA	NA	NA
8	0.01	0.01	0.01	0.01	0.01	0.01	0.01	NA	NA	NA	NA	NA	NA	NA
9	0.01	0.01	0.01	0.01	0.01	0.01	0.01	NA	NA	NA	NA	NA	NA	NA
10	0.01	0.01	0.01	0.01	0.01	0.01	0.01	NA	NA	NA	NA	NA	NA	NA
11	0.01	0.05	0.01	0.10	0.10	0.01	0.01	NA	NA	NA	NA	NA	NA	NA
12	0.01	0.01	0.01	0.10	0.01	0.01	0.01	NA	NA	NA	NA	NA	NA	NA

13	0.01	0.01	0.01	0.01	0.01	0.01	0.01	NA	NA	NA	NA	NA	NA	NA
14	NA	0.01	NA	NA	NA	NA	0.01	NA	NA	NA	NA	NA	NA	NA
15	0.09	0.01	0.01	0.01	0.01	0.01	0.01	NA	NA	NA	NA	NA	NA	NA
16	0.01	0.01	0.01	0.10	0.03	0.01	0.01	NA	NA	NA	NA	NA	NA	NA
17	0.10	0.01	0.02	0.01	0.01	0.02	0.01	0.01	0.01	0.01	0.02	0.10	0.01	0.01
18	0.01	0.01	0.01	0.01	0.06	0.10	0.10	0.05	0.01	0.02	0.01	0.05	0.10	0.01
19	0.01	0.01	0.10	0.03	0.01	0.01	0.01	0.01	0.10	0.03	0.01	0.02	0.01	0.01
20	0.01	0.01	0.01	0.01	0.05	0.01	0.01	0.08	NA	0.01	NA	0.10	NA	0.01
21	0.01	0.01	0.01	0.10	0.01	0.10	0.09	0.01	0.01	0.07	0.01	0.01	0.10	0.04
22	0.01	0.01	0.01	0.01	0.01	0.08	0.05	0.10	0.01	0.02	0.01	0.07	0.01	0.01
23	0.02	0.01	0.01	0.01	0.01	0.01	0.01	NA	NA	NA	NA	NA	NA	NA
24	0.01	0.01	0.01	0.01	0.01	0.01	0.01	0.01	0.01	0.01	0.01	0.06	0.01	0.01
25	0.01	0.01	0.10	0.10	0.01	0.01	0.01	0.01	NA	NA	NA	NA	NA	NA
26	0.06	0.01	0.01	0.01	0.01	0.10	0.01	NA	0.01	0.10	0.04	0.10	0.01	0.01
27	0.01	0.01	0.01	0.02	0.10	0.10	0.08	0.01	0.01	0.01	0.01	0.01	0.05	0.01
28	0.01	0.01	0.05	0.01	0.01	0.01	0.01	0.01	0.10	0.02	0.10	0.04	0.10	0.01
29	0.10	0.08	0.10	0.10	0.01	0.01	0.09	NA	NA	NA	NA	NA	NA	NA
30	0.01	0.01	0.10	0.10	0.10	0.10	0.10	0.04	0.06	0.07	0.10	0.10	0.01	0.02
31	0.03	0.10	0.01	0.02	0.01	0.01	0.10	NA	0.10	0.10	0.10	0.10	0.01	0.01
32	0.01	NA	NA	NA	NA	NA	NA	NA	NA	NA	0.01	0.01	0.01	0.01
33	0.01	0.01	0.01	0.01	0.01	0.01	0.01	NA	NA	NA	NA	NA	NA	NA
34	0.01	0.06	0.01	0.01	0.01	0.01	0.01	NA	NA	NA	NA	NA	NA	NA
35	0.01	0.01	0.01	0.01	0.01	0.01	0.01	NA	NA	NA	NA	NA	NA	NA
36	0.01	0.01	0.01	0.01	0.01	0.01	0.01	NA	NA	NA	NA	NA	NA	NA
37	0.01	0.01	0.10	0.01	0.10	0.01	0.01	NA	NA	NA	NA	NA	NA	NA
38	0.01	0.05	0.01	0.01	0.01	0.01	0.01	NA	NA	NA	NA	NA	NA	NA
39	0.01	0.01	0.01	0.01	0.01	0.01	0.10	NA	NA	NA	NA	NA	NA	NA
40	0.01	0.01	0.01	0.01	0.01	0.01	0.01	NA	NA	NA	NA	NA	NA	NA
41	0.01	0.01	0.01	0.01	0.01	0.01	0.01	NA	NA	NA	NA	NA	NA	NA
42	0.05	0.01	0.01	0.10	0.10	0.10	0.10	NA	NA	NA	NA	NA	NA	NA
43	0.01	0.01	0.01	0.10	0.10	0.01	0.01	NA	NA	NA	NA	NA	NA	NA
44	0.01	0.01	0.01	0.01	0.03	0.02	0.02	NA	NA	NA	NA	NA	NA	NA
45	0.01	0.01	0.01	0.01	0.03	0.01	0.02	NA	NA	NA	NA	NA	NA	NA
46	0.01	0.04	0.01	0.02	0.10	0.01	0.01	NA	NA	NA	NA	NA	NA	NA
47	0.01	0.02	0.01	0.05	0.01	0.01	0.01	NA	NA	NA	NA	NA	NA	NA
48	0.01	0.01	0.01	0.02	0.10	0.01	0.05	NA	NA	NA	NA	NA	NA	NA
49	0.01	0.02	0.01	0.01	0.01	0.08	0.01	NA	NA	NA	NA	NA	NA	NA
50	0.01	0.01	0.01	0.01	0.01	0.01	0.10	NA	NA	NA	NA	NA	NA	NA
51	0.02	0.01	0.10	0.01	0.01	0.01	0.10	NA	NA	NA	NA	NA	NA	NA
52	0.01	0.01	0.01	0.01	0.01	0.01	0.06	NA	NA	NA	NA	NA	NA	NA
53	0.01	0.01	0.01	0.01	0.01	0.04	0.01	NA	NA	NA	NA	NA	NA	NA
54	0.01	0.04	0.01	0.01	0.01	0.01	0.01	NA	NA	NA	NA	NA	NA	NA
55	0.01	0.03	0.01	0.07	0.05	0.01	0.01	NA	NA	NA	NA	NA	NA	NA
56	0.10	0.01	0.01	0.01	0.01	0.01	0.10	NA	NA	NA	NA	NA	NA	NA
57	0.10	0.10	0.10	0.01	0.10	0.01	0.01	NA	NA	NA	NA	NA	NA	NA
58	0.02	0.01	0.02	0.10	0.01	0.01	0.03	NA	NA	NA	NA	NA	NA	NA
59	0.01	0.01	0.01	0.01	0.01	0.01	0.01	NA	NA	NA	NA	NA	NA	NA
60	0.01	0.03	0.01	0.01	0.01	0.01	0.01	NA	NA	NA	NA	NA	NA	NA
61	0.04	0.01	0.01	0.01	0.01	0.01	0.01	NA	NA	NA	NA	NA	NA	NA

62	0.01	0.01	0.10	0.01	0.01	0.01	0.01	NA	NA	NA	NA	NA	NA	NA
63	0.01	0.01	0.01	0.01	0.01	0.01	0.01	NA	NA	NA	NA	NA	NA	NA
64	0.01	0.01	0.01	0.01	0.01	0.01	0.01	NA	NA	NA	NA	NA	NA	NA
65	0.01	0.10	0.01	0.01	0.01	0.10	0.08	NA	NA	NA	NA	NA	NA	NA
66	0.01	0.01	0.01	0.03	0.01	0.01	0.01	NA	NA	NA	NA	NA	NA	NA
67	0.01	0.01	0.01	0.01	0.01	0.01	0.01	NA	NA	NA	NA	NA	NA	NA
68	0.01	0.01	0.02	0.01	0.01	0.01	0.01	NA	NA	NA	NA	NA	NA	NA
69	0.01	0.10	0.01	0.01	0.02	0.01	0.01	NA	NA	NA	NA	NA	NA	NA
70	0.10	0.01	0.01	0.01	0.10	0.10	0.10	NA	NA	NA	NA	NA	NA	NA
71	0.01	0.01	0.01	0.01	0.01	0.01	0.01	NA	NA	NA	NA	NA	NA	NA
72	0.01	0.01	0.01	0.01	0.01	0.01	0.01	NA	NA	NA	NA	NA	NA	NA
73	0.01	0.01	0.01	0.01	0.01	0.01	0.01	NA	NA	NA	NA	NA	NA	NA
74	0.01	0.01	0.01	0.01	0.01	0.01	0.01	NA	NA	NA	NA	NA	NA	NA
75	0.01	0.01	0.01	0.01	0.01	0.01	0.06	NA	NA	NA	NA	NA	NA	NA
76	0.01	0.01	0.01	0.01	0.01	0.01	0.01	NA	NA	NA	NA	NA	NA	NA
77	0.01	0.01	0.01	0.01	0.01	0.01	0.01	NA	NA	NA	NA	NA	NA	NA
78	0.01	0.01	0.10	0.01	0.01	0.01	0.01	NA	NA	NA	NA	NA	NA	NA
79	0.01	0.01	0.10	0.05	0.02	0.10	0.01	NA	NA	NA	NA	NA	NA	NA
80	0.01	0.01	0.01	0.01	0.01	0.01	0.01	NA	NA	NA	NA	NA	NA	NA
81	0.01	0.01	0.01	0.01	0.01	0.01	0.01	NA	NA	NA	NA	NA	NA	NA
82	0.01	0.01	0.01	0.01	0.01	0.01	0.01	NA	NA	NA	NA	NA	NA	NA
83	0.10	0.01	0.01	0.08	0.01	0.01	0.01	NA	NA	NA	NA	NA	NA	NA
84	0.01	0.01	0.01	0.03	0.01	0.01	0.01	NA	NA	NA	NA	NA	NA	NA
85	0.01	0.07	0.01	0.01	0.01	0.01	0.01	0.10	NA	NA	NA	NA	NA	NA
86	0.01	0.01	0.01	0.10	0.01	0.10	0.10	NA	NA	NA	NA	NA	NA	NA
87	0.01	0.01	0.01	0.01	0.01	0.10	0.01	NA	NA	NA	NA	NA	NA	NA
88	0.01	0.01	0.01	0.01	0.01	0.01	0.01	NA	NA	NA	NA	NA	NA	NA
89	0.03	0.01	0.01	0.01	0.01	0.01	0.01	NA	NA	NA	NA	NA	NA	NA
90	0.01	0.01	0.10	0.01	0.01	0.01	0.01	NA	NA	NA	NA	NA	NA	NA
91	0.01	0.01	0.01	0.01	0.01	0.01	0.01	NA	NA	NA	NA	NA	NA	NA
92	0.01	0.01	0.01	0.01	0.01	0.01	0.01	NA	NA	NA	NA	NA	NA	NA
93	0.01	0.02	0.10	0.01	0.10	0.10	0.01	NA	NA	NA	NA	NA	NA	NA
94	0.01	0.01	0.10	0.01	0.01	0.01	0.01	NA	NA	NA	NA	NA	NA	NA
95	0.01	0.01	0.01	0.01	0.01	0.01	0.01	NA	NA	NA	NA	NA	NA	NA
96	0.01	0.01	0.01	0.05	0.01	0.01	0.01	NA	NA	NA	NA	NA	NA	NA
97	0.10	0.01	0.01	0.10	0.10	0.01	0.01	NA	NA	NA	NA	NA	NA	NA
98	0.01	0.01	0.01	0.10	0.01	0.10	0.10	NA	NA	NA	NA	NA	NA	NA
99	0.01	0.01	0.01	0.04	0.01	0.01	0.01	0.07	NA	NA	NA	NA	NA	NA
100	0.01	0.01	0.01	0.01	0.01	0.01	0.04	0.03	NA	NA	NA	NA	NA	NA
101	0.01	0.01	0.02	0.01	0.10	0.01	0.01	0.10	NA	NA	NA	NA	NA	NA
102	0.01	0.01	0.01	0.01	0.01	0.01	0.01	0.01	NA	NA	NA	NA	NA	NA
103	0.01	0.01	0.01	0.01	0.01	0.01	0.01	0.10	NA	NA	NA	NA	NA	NA
104	0.10	0.04	0.10	0.01	0.02	0.10	0.10	NA	NA	NA	NA	NA	NA	NA
105	0.01	0.02	0.01	0.01	0.05	0.01	0.01	0.01	NA	NA	NA	NA	NA	NA
106	0.01	0.01	0.01	0.01	0.01	0.01	0.01	0.01	NA	NA	NA	NA	NA	NA
107	0.01	0.01	0.01	0.10	0.10	0.01	0.01	0.01	NA	NA	NA	NA	NA	NA
108	0.01	0.01	0.01	0.01	0.01	0.02	0.01	0.10	NA	NA	NA	NA	NA	NA
109	0.01	0.01	0.01	0.01	0.01	0.02	0.01	0.10	NA	NA	NA	NA	NA	NA
110	0.01	0.01	0.01	0.01	0.01	0.01	0.01	0.07	NA	NA	NA	NA	NA	NA

111	0.01	0.01	0.02	0.01	0.01	0.01	0.01	0.10	0.01	0.04	0.01	0.07	0.01	0.02
112	0.01	0.01	0.01	0.01	0.10	0.01	0.01	0.01	NA	0.01	NA	0.09	NA	0.01
113	0.01	0.01	0.01	0.01	0.01	0.01	0.01	NA	0.01	0.01	0.01	0.01	0.01	0.01
114	0.10	0.01	0.01	0.01	0.02	0.10	0.02	0.01	NA	NA	NA	NA	NA	NA
115	0.01	0.01	0.01	0.01	0.01	0.01	0.01	NA	NA	NA	NA	NA	NA	NA
116	0.01	0.01	0.01	0.03	0.01	0.01	0.01	0.01	NA	NA	NA	NA	NA	NA
117	0.01	0.01	0.01	0.07	0.01	0.10	0.10	0.04	NA	NA	NA	NA	NA	NA
118	0.01	0.01	0.01	0.03	0.01	0.01	0.04	0.01	NA	NA	NA	NA	NA	NA
119	0.01	0.01	0.10	0.02	0.01	0.01	0.10	0.01	NA	NA	NA	NA	NA	NA
120	0.01	0.01	0.01	0.01	0.01	0.01	0.01	0.01	0.10	0.10	0.01	0.10	0.01	0.01
121	0.01	0.03	0.01	0.02	0.01	0.03	0.01	0.10	0.01	0.01	0.01	0.01	0.01	0.01
122	0.01	0.01	0.01	0.01	0.01	0.01	0.05	0.01	0.01	0.01	0.01	0.10	0.01	0.01
123	0.01	0.01	0.01	0.01	0.10	0.01	0.01	0.01	0.01	0.01	0.01	0.01	0.01	0.06
124	0.10	0.01	0.02	0.01	0.01	0.01	0.01	NA	NA	NA	NA	NA	NA	NA
125	0.01	0.01	0.01	0.01	0.01	0.01	0.01	NA	0.01	0.05	0.01	0.08	0.01	0.01
126	0.01	0.01	0.01	0.01	0.06	0.01	0.01	NA	NA	0.10	NA	0.10	NA	0.01
127	0.10	0.01	0.04	0.01	0.01	0.01	0.01	NA	NA	NA	NA	NA	NA	NA
128	0.01	0.01	0.01	0.01	0.01	0.01	0.01	NA	NA	NA	NA	NA	NA	NA
129	0.01	0.01	0.01	0.01	0.01	0.01	0.01	NA	0.01	0.01	0.01	0.01	0.01	0.10
130	0.01	0.01	0.01	0.01	0.02	0.08	0.01	NA	0.10	0.10	0.10	0.10	0.01	0.02
131	0.01	0.01	0.01	0.01	0.01	0.01	0.01	NA	0.01	0.01	0.01	0.01	0.01	0.01
132	0.10	0.01	0.01	0.01	0.01	0.01	0.01	NA	NA	NA	NA	NA	NA	NA
133	0.01	0.01	0.01	0.01	0.01	0.01	0.06	NA	NA	NA	NA	NA	NA	NA
134	0.01	0.01	0.01	0.01	0.01	0.01	0.01	NA	NA	NA	NA	NA	NA	NA
135	0.01	0.01	0.02	0.01	0.01	0.01	0.10	NA	NA	NA	NA	NA	NA	NA
136	0.01	0.01	0.05	0.01	0.10	0.10	0.01	NA	NA	NA	NA	NA	NA	NA
137	0.01	0.01	0.01	0.01	0.01	0.01	0.01	NA	NA	NA	NA	NA	NA	NA
138	0.01	0.01	0.01	0.01	0.01	0.10	0.01	NA	NA	NA	NA	NA	NA	NA
139	0.01	0.01	0.01	0.05	0.05	0.02	0.01	NA	NA	NA	NA	NA	NA	NA
140	0.01	0.01	0.10	0.10	0.10	0.01	0.01	NA	NA	NA	NA	NA	NA	NA
141	0.08	0.10	0.10	0.01	0.01	0.01	0.01	NA	NA	NA	NA	NA	NA	NA
142	0.10	0.10	0.10	0.01	0.01	0.01	0.01	NA	NA	NA	NA	NA	NA	NA
143	0.01	0.01	0.01	0.01	0.01	0.01	0.01	NA	NA	NA	NA	NA	NA	NA
144	0.04	0.04	0.08	0.01	0.01	0.01	0.01	NA	NA	NA	NA	NA	NA	NA
145	0.01	0.01	0.01	0.02	0.10	0.01	0.01	NA	NA	NA	NA	NA	NA	NA
146	0.01	0.01	0.01	0.01	0.10	0.08	0.06	NA	NA	NA	NA	NA	NA	NA
147	0.01	0.01	0.01	0.01	0.01	0.02	0.05	NA	NA	NA	NA	NA	NA	NA
148	0.01	0.01	0.01	0.04	0.02	0.07	0.01	NA	NA	NA	NA	NA	NA	NA
149	0.01	0.02	0.01	0.01	0.01	0.01	0.01	NA	NA	NA	NA	NA	NA	NA
150	0.01	0.02	0.01	0.01	0.01	0.01	0.01	NA	NA	NA	NA	NA	NA	NA
151	0.01	0.01	0.01	0.01	0.01	0.01	0.02	NA	NA	NA	NA	NA	NA	NA
152	0.01	0.01	0.01	0.01	0.01	0.03	0.10	NA	NA	NA	NA	NA	NA	NA
153	0.01	0.01	0.01	0.07	0.01	0.01	0.01	NA	0.02	0.08	0.10	0.05	0.01	0.01
154	0.01	0.01	0.03	0.01	0.01	0.01	0.01	NA	NA	NA	NA	NA	NA	NA
155	0.01	0.01	0.09	0.01	0.01	0.01	0.01	NA	NA	NA	NA	NA	NA	NA
156	0.10	0.01	0.05	0.02	0.01	0.03	0.01	NA	NA	NA	NA	NA	NA	NA
157	0.01	0.01	0.10	0.01	0.01	0.01	0.01	NA	NA	NA	NA	NA	NA	NA
158	0.08	0.01	0.05	0.10	0.10	0.10	0.09	NA	0.10	0.10	0.10	0.10	0.10	0.10
159	0.10	0.01	0.10	0.10	0.10	0.10	0.03	NA	0.01	0.04	0.01	0.09	0.01	0.10

160	0.01	0.01	0.01	0.01	0.01	0.01	0.01	NA	NA	0.07	NA	0.05	NA	0.01
161	0.01	0.01	0.10	0.01	0.01	0.02	0.01	NA	NA	NA	NA	NA	NA	NA
162	0.01	0.01	0.01	0.01	0.01	0.01	0.10	NA	NA	NA	NA	NA	NA	NA
163	0.01	0.01	0.02	0.10	0.10	0.09	0.01	NA	NA	NA	NA	NA	NA	NA
164	0.01	0.01	0.01	0.02	0.01	0.01	0.01	NA	NA	NA	NA	NA	NA	NA
165	0.01	0.09	0.01	0.08	0.01	0.01	0.10	NA	NA	NA	NA	NA	NA	NA
166	0.01	0.01	0.01	0.01	0.01	0.01	0.01	NA	NA	NA	NA	NA	NA	NA
167	0.01	0.01	0.03	0.01	0.01	0.01	0.01	NA	0.01	0.10	0.01	0.10	0.01	0.01
168	0.07	0.01	0.07	0.10	0.10	0.10	0.10	NA	0.10	NA	0.10	NA	0.01	NA
169	0.01	0.01	0.10	0.01	0.01	0.01	0.01	NA	0.01	0.10	0.01	0.10	0.03	0.01
170	0.01	0.01	0.01	0.01	0.01	0.01	0.01	NA	NA	NA	NA	NA	NA	NA
171	0.01	0.01	0.10	0.01	0.01	0.01	0.08	NA	0.01	0.10	0.01	0.10	0.01	0.10
172	0.01	0.01	0.01	0.01	0.01	0.01	0.01	NA	0.01	0.10	0.01	0.10	0.01	0.01
173	0.02	0.01	0.04	0.10	0.01	0.01	0.01	NA	NA	NA	NA	NA	NA	NA
174	0.04	0.01	0.01	0.01	0.01	0.01	0.01	NA	0.02	0.10	0.01	0.10	0.01	0.01
175	0.01	0.01	0.01	0.01	0.01	0.01	0.01	NA	NA	NA	NA	NA	NA	NA
176	0.01	0.01	0.10	0.01	0.01	0.02	0.05	NA	NA	NA	NA	NA	NA	NA
177	0.01	0.01	0.01	0.01	0.01	0.01	0.01	NA	NA	NA	NA	NA	NA	NA
178	0.01	0.05	0.01	0.02	0.10	0.01	0.10	NA	NA	NA	NA	NA	NA	NA
179	0.01	0.01	0.01	0.01	0.01	0.01	0.01	NA	NA	NA	NA	NA	NA	NA
180	0.01	0.01	0.02	0.09	0.04	0.04	0.10	NA	NA	NA	NA	NA	NA	NA
181	0.06	0.01	0.01	0.09	0.10	0.09	0.04	NA	NA	NA	NA	NA	NA	NA
182	0.01	0.01	0.03	0.01	0.03	0.01	0.01	NA	NA	NA	NA	NA	NA	NA
183	0.01	0.10	0.01	0.10	0.10	0.10	0.01	NA	NA	NA	NA	NA	NA	NA
184	0.01	0.01	0.01	0.01	0.01	0.01	0.01	NA	NA	NA	NA	NA	NA	NA
185	0.10	0.01	0.08	0.10	0.10	0.03	0.10	NA	NA	NA	NA	NA	NA	NA
186	0.08	0.01	0.10	0.02	0.10	0.10	0.06	NA	NA	NA	NA	NA	NA	NA
187	0.01	0.01	0.01	0.01	0.01	0.01	0.01	NA	NA	0.01	NA	0.01	NA	0.01
188	0.01	0.01	0.01	0.01	0.01	0.01	0.01	NA	0.04	NA	0.07	NA	0.01	NA
189	0.01	0.01	0.01	0.01	0.01	0.01	0.01	NA	0.10	0.10	0.10	0.02	0.01	0.03
190	0.01	0.10	0.01	0.10	0.10	0.10	0.06	NA	NA	NA	NA	NA	NA	NA
191	0.01	0.01	0.01	0.10	0.01	0.01	0.01	NA	0.01	0.01	0.01	0.01	0.03	0.01
192	0.01	0.01	0.01	0.01	0.10	0.01	0.01	NA	0.10	0.06	0.10	0.04	0.01	0.01
193	0.01	0.01	0.01	0.01	0.01	0.01	0.01	NA	0.01	0.01	0.01	0.01	0.01	0.01
194	0.01	0.01	0.01	0.01	0.01	0.01	0.01	NA	NA	NA	NA	NA	NA	NA
195	0.01	0.01	0.01	0.01	0.01	0.01	0.01	NA	0.02	0.01	0.02	0.01	0.01	0.01
196	0.10	0.01	0.10	0.01	0.01	0.01	0.01	NA	NA	NA	NA	NA	NA	NA
197	0.02	0.10	0.04	0.02	0.01	0.01	0.01	NA	NA	0.10	NA	0.10	0.01	0.01
198	0.01	0.01	0.04	0.03	0.01	0.02	0.01	NA	0.05	0.10	0.08	0.10	0.04	0.01
199	0.01	0.04	0.01	0.01	0.01	0.01	0.01	NA	0.01	0.01	0.01	0.01	0.01	0.01
200	0.01	0.01	0.10	0.01	0.01	0.01	0.10	NA	0.10	0.10	0.01	0.10	0.01	0.01
201	0.10	0.01	0.01	0.01	0.09	0.01	0.01	NA	0.10	0.10	0.10	0.10	0.01	0.08
202	0.01	0.01	0.01	0.05	0.01	0.01	0.01	NA	0.01	0.01	0.01	0.01	0.01	0.01
203	0.01	0.02	0.01	0.05	0.05	0.10	0.10	NA	0.10	NA	0.01	NA	0.01	NA
204	0.10	0.02	0.01	0.10	0.01	0.10	0.01	NA	0.10	0.01	0.10	0.10	0.01	0.01
205	0.01	0.01	0.01	0.01	0.01	0.01	0.10	NA	0.10	0.02	0.06	0.02	0.09	0.01
206	0.01	0.01	0.01	0.01	0.01	0.01	0.04	NA	0.10	0.01	0.10	0.08	0.01	0.01
207	0.01	0.01	0.01	0.01	0.01	0.01	0.01	NA	0.10	0.10	0.09	0.10	0.01	0.01
208	0.01	0.01	0.01	0.01	0.01	0.01	0.01	NA	0.05	0.01	0.02	0.05	0.01	0.01

209	0.05	0.01	0.01	0.01	0.01	0.01	0.01	NA	0.05	0.01	0.10	0.04	0.01	0.01
210	0.09	0.01	0.01	0.01	0.05	0.01	0.01	NA	0.01	0.01	0.01	0.01	0.01	0.01
211	0.09	0.01	0.01	0.10	0.01	0.08	0.01	NA	0.01	0.01	0.01	0.10	0.01	0.01
212	0.07	0.01	0.10	0.01	0.01	0.01	0.10	NA	0.07	0.04	0.10	0.10	0.01	0.01
213	0.01	0.01	0.10	0.10	0.10	0.10	0.10	NA	0.01	0.01	0.01	0.01	0.10	0.01
214	0.02	0.01	0.01	0.01	0.10	0.02	0.02	NA	0.01	0.01	0.06	0.01	0.01	0.01
215	0.01	0.01	0.01	0.01	0.01	0.01	0.10	NA	0.10	0.10	0.10	0.10	0.01	0.10
216	0.01	0.01	0.01	0.01	0.05	0.10	0.10	NA	0.10	0.01	0.10	0.01	0.01	0.01
217	0.01	0.01	0.01	0.01	0.01	0.01	0.01	NA	0.01	0.02	0.01	0.01	0.01	0.01
218	0.01	0.01	0.01	0.02	0.01	0.10	0.10	NA	0.10	0.10	0.10	0.10	0.01	0.01
219	0.01	0.01	0.01	0.02	0.10	0.01	0.10	NA	0.01	0.01	0.01	0.01	0.01	0.01
220	0.01	0.01	0.01	0.03	0.08	0.10	0.02	NA	NA	NA	NA	NA	NA	NA
221	0.01	0.01	0.02	0.05	0.01	0.01	0.10	NA	0.02	0.10	0.10	0.10	0.01	0.01
222	0.01	0.01	0.08	0.10	0.03	0.10	0.04	NA	0.10	0.10	0.10	0.10	0.01	0.01
223	0.01	0.01	0.01	0.10	0.10	0.10	0.10	NA	0.10	0.02	0.10	0.01	0.01	0.01
224	0.01	0.01	0.10	0.10	0.01	0.01	0.01	NA	0.10	0.10	0.10	0.09	0.01	0.01
225	0.01	0.01	0.01	0.03	0.10	0.01	0.01	NA	0.10	0.01	0.10	0.01	0.01	0.01
226	0.01	0.02	0.01	0.06	0.09	0.01	0.10	NA	0.10	0.07	0.10	0.06	0.01	0.01
227	0.01	0.01	0.01	0.01	0.01	0.05	0.01	NA	0.01	0.01	0.01	0.01	0.01	0.01
228	0.01	0.02	0.10	0.01	0.02	0.10	0.09	NA	0.02	0.03	0.01	0.09	0.07	0.08
229	0.01	0.01	0.01	0.01	0.01	0.01	0.01	NA	0.01	0.02	0.01	0.01	0.01	0.01
230	0.01	0.01	0.04	0.01	0.04	0.10	0.01	NA	0.01	0.03	0.01	0.10	0.01	0.01
231	0.01	0.01	0.01	0.02	0.10	0.04	0.01	NA	0.04	0.08	0.09	0.09	0.01	0.01
232	0.01	0.01	0.01	0.01	0.01	0.01	0.02	NA	0.01	0.01	0.04	0.01	0.01	0.02
233	0.01	0.01	0.02	0.01	0.01	0.01	0.01	NA	0.10	NA	0.10	NA	0.02	NA
234	0.08	0.01	0.01	0.01	0.01	0.01	0.10	0.01	0.01	0.01	0.01	0.01	0.01	0.01
235	0.01	0.01	0.01	0.09	0.01	0.01	0.01	0.01	NA	NA	NA	NA	NA	NA
236	0.10	0.01	0.09	0.10	0.01	0.01	0.10	0.10	0.10	0.10	0.10	0.04	0.01	0.01
237	0.01	0.01	0.01	0.01	0.01	0.01	0.01	NA	0.01	NA	0.01	NA	0.01	NA
238	0.01	0.01	0.01	0.10	0.10	0.10	0.01	NA	0.10	0.02	0.10	0.03	0.01	0.01
239	0.01	0.10	0.01	0.10	0.10	0.10	0.10	0.01	0.10	0.10	0.09	0.10	0.04	0.10
240	0.01	0.01	0.10	0.01	0.01	0.01	0.01	NA	0.01	0.08	0.10	0.10	0.01	0.01
241	0.01	0.01	0.01	0.01	0.01	0.01	0.01	0.01	0.01	0.03	0.10	0.10	0.01	0.01
242	0.01	0.01	0.01	0.01	0.01	0.01	0.10	NA	0.10	0.10	0.05	0.08	0.02	0.10
243	0.01	0.01	0.01	0.01	0.01	0.01	0.01	NA	0.01	0.01	0.01	0.01	0.01	0.01
244	0.01	0.01	0.03	0.10	0.10	0.01	0.01	NA	0.10	0.10	0.10	0.10	0.01	0.01
245	0.01	0.01	0.01	0.03	0.01	0.01	0.01	0.01	0.01	0.01	0.01	0.01	0.01	0.01
246	0.01	0.01	0.01	0.01	0.01	0.01	0.01	NA	0.01	0.01	0.01	0.01	0.01	0.01
247	0.01	0.10	0.01	0.01	0.01	0.01	0.01	0.01	0.08	NA	0.10	NA	0.01	NA
248	0.01	0.01	0.01	0.10	0.04	0.01	0.06	NA	0.03	0.01	0.01	0.05	0.01	0.01
249	0.01	0.10	0.01	0.01	0.01	0.06	0.01	0.04	0.01	0.10	0.01	0.10	0.01	0.01
250	0.01	0.05	0.10	0.01	0.01	0.01	0.10	0.01	0.09	0.02	0.01	0.02	0.01	0.01
251	0.10	0.01	0.01	0.01	0.01	0.01	0.01	NA	NA	0.10	NA	0.10	NA	0.01
252	0.01	0.01	0.01	0.01	0.01	0.01	0.01	0.01	0.10	0.01	0.08	0.01	0.01	0.01
253	0.01	0.01	0.01	0.01	0.01	0.01	0.02	0.01	0.01	0.10	0.01	0.04	0.01	0.01
254	0.01	0.01	0.01	0.01	0.01	0.01	0.01	NA	0.04	0.10	0.04	0.10	0.01	0.01
255	0.02	0.01	0.02	0.01	0.05	0.01	0.01	NA	0.10	NA	0.10	NA	0.01	NA
256	0.10	0.01	0.10	0.01	0.01	0.01	0.01	NA	0.10	0.01	0.10	0.01	0.01	0.01
257	0.01	0.01	0.10	0.01	0.01	0.01	0.03	NA	0.10	0.03	0.10	0.01	0.02	0.01

258	0.01	0.01	0.01	0.01	0.01	0.01	0.01	NA	0.01	0.01	0.10	0.01	0.01	0.01
259	0.01	0.01	0.01	0.01	0.01	0.01	0.03	NA	0.10	0.08	0.10	0.07	0.01	0.01
260	0.04	0.01	0.05	0.01	0.01	0.01	0.01	0.01	0.01	0.01	0.01	0.01	0.01	0.01
261	0.01	0.01	0.01	0.01	0.04	0.01	0.01	NA	0.10	0.02	0.08	0.06	0.01	0.01
262	0.01	0.01	0.01	0.01	0.09	0.01	0.01	NA	0.07	0.01	0.10	0.01	0.10	0.01
263	0.01	0.01	0.01	0.01	0.01	0.01	0.01	0.01	0.01	0.01	0.01	0.01	0.01	0.01
264	0.10	0.01	0.02	0.01	0.01	0.01	0.01	0.01	0.01	0.01	0.01	0.01	0.01	0.01
265	0.01	0.01	0.01	0.01	0.01	0.01	0.01	0.04	0.02	0.01	0.01	0.01	0.01	0.01
266	0.10	0.01	0.10	0.10	0.10	0.01	0.01	NA	0.01	NA	0.10	NA	0.01	NA
267	0.01	0.01	0.03	0.01	0.01	0.01	0.01	NA	0.01	0.01	0.01	0.01	0.01	0.01
268	0.02	0.10	0.01	0.07	0.10	0.10	0.01	NA	0.08	0.10	0.10	0.05	0.01	0.01
269	0.01	0.01	0.01	0.01	0.08	0.01	0.01	NA	0.10	0.10	0.02	0.10	0.01	0.02
270	0.01	0.01	0.01	0.10	0.01	0.01	0.01	NA	0.01	0.09	0.01	0.10	0.01	0.01
271	0.01	0.02	0.01	0.01	0.01	0.01	0.10	NA	0.01	0.01	0.01	0.01	0.01	0.01
272	0.01	0.04	0.01	0.08	0.03	0.02	0.01	NA	0.01	0.05	0.01	0.01	0.01	0.08
273	0.10	0.01	0.01	0.01	0.10	0.01	0.01	NA	0.10	0.01	0.10	0.01	0.04	0.06
274	0.06	0.01	0.05	0.01	0.01	0.01	0.01	NA	0.10	0.01	0.10	0.01	0.01	0.01
275	0.01	0.01	0.01	0.01	0.01	0.01	0.01	0.01	0.01	0.01	0.01	0.01	0.01	0.01
276	0.10	0.01	0.01	0.02	0.02	0.01	0.01	NA	0.01	0.02	0.01	0.01	0.01	0.05
277	0.01	0.01	0.01	0.01	0.01	0.01	0.01	NA	0.01	0.01	0.08	0.10	0.05	0.04
278	0.01	0.01	0.01	0.01	0.01	0.01	0.10	NA	0.01	0.01	0.02	0.01	0.01	0.01
279	0.07	0.01	0.01	0.01	0.02	0.01	0.01	NA	0.10	0.05	0.10	0.05	0.01	0.01
280	0.01	0.01	0.01	0.01	0.07	0.01	0.01	NA	0.01	0.09	0.01	0.10	0.01	0.01
281	0.01	0.10	0.01	0.10	0.10	0.10	0.01	NA	0.10	0.10	0.10	0.10	0.01	0.01
282	0.01	0.01	0.08	0.01	0.01	0.01	0.04	NA	0.01	0.01	0.01	0.01	0.01	0.01
283	0.01	0.01	0.04	0.01	0.04	0.01	0.09	NA	NA	NA	NA	NA	NA	NA
284	0.01	0.01	0.01	0.01	0.01	0.01	0.01	NA	NA	NA	NA	NA	NA	NA
285	0.01	0.01	0.01	0.01	0.01	0.01	0.01	NA	0.01	0.01	0.01	0.01	0.01	0.01
286	0.01	0.10	0.01	0.01	0.01	0.01	0.09	NA	0.01	0.10	0.01	0.01	0.01	0.01
287	0.01	0.01	0.01	0.01	0.01	0.01	0.01	NA	0.01	0.01	0.01	0.01	0.01	0.01
288	0.10	0.01	0.01	0.01	0.01	0.01	0.01	NA	0.01	0.06	0.01	0.01	0.10	0.02
289	0.10	0.10	0.10	0.01	0.01	0.01	0.01	NA	0.01	0.01	0.02	0.01	0.01	0.01
290	0.01	0.01	0.10	0.06	0.10	0.10	0.10	NA	0.01	0.01	0.01	0.01	0.10	0.07
291	0.10	0.01	0.04	0.01	0.01	0.01	0.01	NA	0.01	0.04	0.01	0.03	0.01	0.01
292	0.01	0.04	0.01	0.01	0.01	0.01	0.01	NA	NA	0.01	NA	0.01	NA	0.01
293	0.01	0.01	0.01	0.01	0.01	0.01	0.02	NA	0.01	0.01	0.06	0.10	0.01	0.01
294	0.01	0.01	0.01	0.01	0.01	0.01	0.01	NA	0.01	0.01	0.04	0.02	0.01	0.01
295	0.01	0.10	0.10	0.01	0.01	0.01	0.01	NA	0.01	0.07	0.05	0.10	0.01	0.01
296	0.10	0.10	0.10	0.01	0.02	0.01	0.10	NA	0.03	0.01	0.04	0.01	0.01	0.01
297	0.01	0.01	0.01	0.10	0.10	0.05	0.03	NA	0.01	0.03	0.01	0.06	0.01	0.01
298	0.10	0.01	0.10	0.04	0.01	0.01	0.09	NA	0.04	0.01	0.10	0.01	0.01	0.01
299	0.01	0.01	0.01	0.01	0.01	0.01	0.01	NA	0.01	NA	0.01	NA	0.10	NA
300	0.01	0.05	0.01	0.01	0.01	0.01	0.01	NA	NA	NA	NA	NA	NA	NA
301	0.01	0.07	0.01	0.01	0.01	0.01	0.02	NA	NA	NA	NA	NA	NA	NA
302	0.01	0.01	0.10	0.01	0.01	0.01	0.01	NA	NA	NA	NA	NA	NA	NA
303	0.01	0.01	0.01	0.02	0.01	0.01	0.01	NA	NA	NA	NA	NA	NA	NA
304	0.01	0.01	0.01	0.02	0.10	0.10	0.10	NA	NA	NA	NA	NA	NA	NA
305	0.01	0.01	0.01	0.10	0.10	0.01	0.01	0.01	NA	NA	NA	NA	NA	NA
306	0.01	0.01	0.01	0.01	0.10	0.10	0.03	0.01	NA	NA	NA	NA	NA	NA

307	0.01	0.05	0.01	0.01	0.01	0.01	0.01	0.10	NA	NA	NA	NA	NA	NA
308	0.10	0.01	0.01	0.01	0.01	0.01	0.01	0.01	NA	NA	NA	NA	NA	NA
309	0.01	0.02	0.01	0.10	0.01	0.01	0.10	0.01	NA	NA	NA	NA	NA	NA
310	0.01	0.01	0.01	0.10	0.10	0.04	0.10	0.01	NA	NA	NA	NA	NA	NA
311	0.01	0.02	0.01	0.10	0.09	0.10	0.04	0.01	NA	NA	NA	NA	NA	NA
312	0.01	0.01	0.01	0.01	0.01	0.01	0.10	0.01	NA	NA	NA	NA	NA	NA
313	0.01	0.07	0.01	0.01	0.03	0.01	0.01	0.01	NA	NA	NA	NA	NA	NA
314	0.01	0.01	0.01	0.01	0.01	0.01	0.01	0.01	NA	NA	NA	NA	NA	NA
315	0.01	0.01	0.01	0.10	0.01	0.01	0.04	NA	NA	NA	NA	NA	NA	NA
316	0.01	0.10	0.01	0.04	0.07	0.01	0.01	0.01	NA	NA	NA	NA	NA	NA
317	0.08	0.01	0.01	0.04	0.06	0.01	0.03	0.01	NA	NA	NA	NA	NA	NA
318	0.01	0.09	0.01	0.10	0.01	0.01	0.01	0.01	NA	NA	NA	NA	NA	NA
319	0.01	0.01	0.08	0.10	0.01	0.01	0.01	0.01	NA	NA	NA	NA	NA	NA
320	0.01	0.02	0.01	0.01	0.01	0.01	0.01	0.01	NA	NA	NA	NA	NA	NA
321	0.01	0.01	0.04	0.01	0.01	0.01	0.01	0.01	NA	NA	NA	NA	NA	NA
322	0.01	0.01	0.01	0.01	0.01	0.01	0.01	0.01	NA	NA	NA	NA	NA	NA
323	0.01	0.01	0.01	0.01	0.01	0.01	0.10	0.01	NA	NA	NA	NA	NA	NA
324	0.01	0.02	0.01	0.01	0.10	0.01	0.01	0.01	NA	NA	NA	NA	NA	NA
325	0.01	0.01	0.04	0.10	0.01	0.01	0.01	0.08	NA	NA	NA	NA	NA	NA
326	0.01	0.01	0.02	0.01	0.01	0.01	0.01	0.01	NA	NA	NA	NA	NA	NA
327	0.01	0.10	0.03	0.01	0.01	0.01	0.01	0.01	NA	NA	NA	NA	NA	NA
328	0.10	0.01	0.08	0.01	0.10	0.01	0.01	0.01	NA	NA	NA	NA	NA	NA
329	0.01	0.01	0.01	0.01	0.01	0.01	0.04	0.01	NA	NA	NA	NA	NA	NA
330	0.01	0.01	0.01	0.01	0.01	0.01	0.01	0.10	NA	NA	NA	NA	NA	NA
331	0.10	0.01	0.07	0.01	0.01	0.01	0.01	0.10	NA	NA	NA	NA	NA	NA
332	0.01	0.01	0.01	0.01	0.01	0.01	0.01	0.04	NA	NA	NA	NA	NA	NA
333	0.01	0.03	0.01	0.01	0.01	0.01	0.01	0.01	NA	NA	NA	NA	NA	NA
334	0.01	0.01	0.01	0.03	0.02	0.01	0.01	0.01	NA	NA	NA	NA	NA	NA
335	0.01	0.01	0.01	0.04	0.01	0.01	0.10	0.01	NA	NA	NA	NA	NA	NA
336	0.01	0.01	0.10	0.04	0.01	0.01	0.01	0.01	NA	NA	NA	NA	NA	NA
337	0.01	0.05	0.01	0.01	0.01	0.01	0.01	0.01	NA	NA	NA	NA	NA	NA
338	0.01	0.01	0.01	0.01	0.01	0.01	0.01	0.04	0.02	0.01	NA	NA	0.02	0.10
339	0.05	0.01	0.01	0.01	0.01	0.01	0.02	0.01	0.10	0.10	NA	NA	0.01	0.07
340	0.01	0.01	0.01	0.01	0.01	0.01	0.04	0.02	NA	NA	NA	NA	NA	NA
341	0.01	0.01	0.01	0.01	0.01	0.01	0.01	0.01	NA	NA	NA	NA	NA	NA
342	0.01	0.01	0.01	0.01	0.01	0.01	0.01	NA	NA	NA	NA	NA	NA	NA
343	0.01	0.01	0.10	0.04	0.03	0.01	0.01	NA	NA	NA	NA	NA	NA	NA
344	0.07	0.01	0.02	0.01	0.01	0.01	0.01	NA	NA	NA	NA	NA	NA	NA
345	0.01	0.01	0.09	0.01	0.01	0.01	0.10	0.01	NA	NA	NA	NA	NA	NA
346	0.01	0.01	0.01	0.01	0.01	0.10	0.01	0.01	NA	NA	NA	NA	NA	NA
347	0.02	0.01	0.01	0.01	0.01	0.01	0.01	0.01	NA	NA	NA	NA	NA	NA
348	0.01	0.01	0.01	0.01	0.03	0.01	0.01	0.01	NA	NA	NA	NA	NA	NA
349	0.01	0.01	0.01	0.01	0.01	0.01	0.01	0.01	NA	NA	NA	NA	NA	NA
350	0.04	0.07	0.10	0.10	0.01	0.01	0.07	0.10	NA	NA	NA	NA	NA	NA
351	0.01	0.01	0.01	0.01	0.01	0.01	0.01	0.01	NA	NA	NA	NA	NA	NA
352	0.01	0.01	0.01	0.03	0.01	0.01	0.01	0.01	NA	NA	NA	NA	NA	NA
353	0.01	0.01	0.01	0.01	0.01	0.01	0.01	0.02	NA	NA	NA	NA	NA	NA
354	0.01	0.01	0.01	0.01	0.01	0.01	0.01	0.01	NA	NA	NA	NA	NA	NA
355	0.01	0.01	0.01	0.01	0.01	0.01	0.01	0.01	NA	NA	NA	NA	NA	NA

356	0.10	0.10	0.04	0.01	0.01	0.01	0.01	0.01	NA	NA	NA	NA	NA	NA
357	0.01	0.08	0.03	0.01	0.01	0.01	0.01	0.01	NA	NA	NA	NA	NA	NA
358	0.01	0.01	0.01	0.01	0.01	0.01	0.01	0.01	NA	NA	NA	NA	NA	NA
359	0.01	0.01	0.01	0.01	0.01	0.02	0.01	0.01	NA	NA	NA	NA	NA	NA
360	0.01	0.01	0.09	0.01	0.01	0.10	0.01	0.10	NA	NA	NA	NA	NA	NA
361	0.01	0.01	0.01	0.01	0.01	0.10	0.04	0.01	0.01	0.01	NA	NA	0.01	0.01
362	0.01	0.01	0.01	0.01	0.01	0.01	0.01	NA	NA	NA	NA	NA	NA	NA
363	0.01	0.01	0.01	0.01	0.10	0.01	0.01	0.01	NA	NA	NA	NA	NA	NA
364	0.01	0.01	0.01	0.01	0.01	0.02	0.01	0.01	NA	NA	NA	NA	NA	NA
365	0.01	0.01	0.01	0.01	0.01	0.01	0.01	0.01	NA	NA	NA	NA	NA	NA
366	0.01	0.10	0.01	0.10	0.02	0.10	0.10	0.01	0.01	0.01	NA	NA	0.02	0.02
367	0.01	0.06	0.01	0.03	0.01	0.01	0.04	NA	NA	NA	NA	NA	NA	NA
368	0.10	0.01	0.01	0.01	0.04	0.01	0.01	0.01	NA	NA	NA	NA	NA	NA
369	0.02	0.02	0.01	0.01	0.01	0.02	0.01	0.01	NA	NA	NA	NA	NA	NA
370	0.01	0.10	0.02	0.01	0.03	0.01	0.01	0.10	NA	NA	NA	NA	NA	NA
371	0.01	0.01	0.01	0.01	0.01	0.01	0.01	0.01	NA	NA	NA	NA	NA	NA
372	0.01	0.02	0.09	0.01	0.01	0.01	0.01	0.01	NA	NA	NA	NA	NA	NA
373	0.01	0.03	0.01	0.01	0.01	0.01	0.03	0.01	NA	NA	NA	NA	NA	NA
374	0.02	0.09	0.03	0.01	0.01	0.01	0.01	0.01	NA	NA	NA	NA	NA	NA
375	0.01	0.10	0.01	0.01	0.01	0.01	0.01	NA	NA	NA	NA	NA	NA	NA
376	0.01	0.01	0.01	0.01	0.01	0.03	0.01	0.01	NA	NA	NA	NA	NA	NA

Table S2 - p-values of ADF and KPSS tests on first-order differenced data of all patients

p-values of ADF test on 1st-order differenced data														
**Patient**	**MAP**	**ICP**	**CPP**	**PRx**	**PAx**	**RAC**	**RAP**	**$PbtO_2$**	**COx_L**	**COx_R**	**COx-a_L**	**COx-a_R**	**rSO_2_L**	**rSO_2_R**
1	1.03E-17	4.71E-21	0	1.44E-24	1.07E-20	1.80E-19	1.25E-25	NA	NA	NA	NA	NA	NA	NA
2	0	7.49E-18	0	3.38E-20	2.99E-20	1.32E-21	2.57E-20	NA	NA	NA	NA	NA	NA	NA
3	0.007376	0.000592	1.89E-05	5.06E-14	5.87E-16	1.03E-11	5.09E-08	NA	NA	NA	NA	NA	NA	NA
4	7.53E-23	6.15E-24	3.66E-16	2.88E-14	2.20E-16	6.73E-15	2.71E-20	9.77E-26	4.01E-19	1.54E-18	3.61E-17	5.74E-23	0	0
5	6.18E-06	1.67E-10	8.41E-08	1.13E-15	6.71E-17	1.83E-13	3.78E-21	NA	NA	NA	NA	NA	NA	NA
6	4.37E-25	1.07E-26	3.93E-26	1.00E-26	0	2.63E-30	6.74E-25	NA	NA	NA	NA	NA	NA	NA
7	0	0	0	3.06E-25	2.17E-16	6.68E-26	9.31E-24	NA	NA	NA	NA	NA	NA	NA
8	0	0	0	2.02E-30	4.77E-29	2.55E-29	0	NA	NA	NA	NA	NA	NA	NA
9	4.17E-27	4.33E-30	5.81E-27	5.72E-28	2.62E-28	4.83E-28	4.08E-24	NA	NA	NA	NA	NA	NA	NA
10	1.61E-29	0	3.93E-30	0	0	2.82E-30	0	NA	NA	NA	NA	NA	NA	NA
11	5.26E-18	2.03E-16	8.55E-26	1.90E-14	5.56E-14	1.66E-08	1.08E-16	NA	NA	NA	NA	NA	NA	NA
12	6.32E-24	0	3.47E-26	1.63E-23	2.03E-24	6.85E-26	7.82E-23	NA	NA	NA	NA	NA	NA	NA
13	3.72E-30	3.01E-28	4.66E-29	0	0	0	7.26E-30	NA	NA	NA	NA	NA	NA	NA
14	NA	3.21E-24	NA	NA	NA	NA	1.48E-26	NA	NA	NA	NA	NA	NA	NA
15	3.34E-27	0	2.31E-27	0	0	0	0	NA	NA	NA	NA	NA	NA	NA
16	1.61E-24	1.44E-23	7.79E-22	2.86E-25	1.00E-18	3.19E-20	2.92E-22	NA	NA	NA	NA	NA	NA	NA
17	1.33E-28	2.39E-29	8.04E-23	2.47E-24	2.95E-23	5.48E-26	1.12E-21	1.28E-28	3.73E-14	5.71E-25	8.90E-23	1.58E-23	1.52E-12	1.13E-26
18	1.43E-29	2.00E-09	4.24E-30	1.61E-18	1.55E-22	9.48E-21	3.02E-21	4.43E-25	3.84E-28	7.70E-19	1.09E-23	2.05E-23	1.49E-27	2.01E-27
19	1.50E-29	9.12E-29	3.15E-28	0	2.40E-28	1.23E-29	2.17E-29	7.95E-27	2.96E-17	4.96E-19	5.50E-24	1.98E-24	3.28E-27	2.22E-30
20	2.27E-30	7.67E-28	0	0	0	4.96E-30	1.86E-24	NA	NA	1.96E-19	NA	0	NA	4.06E-10
21	3.99E-24	0	2.46E-20	4.03E-27	1.36E-26	9.43E-22	2.52E-24	5.03E-30	6.49E-30	3.19E-18	2.99E-26	4.63E-24	6.27E-30	0
22	0	0	0	3.19E-29	0	0	0	0.000933	0	0	0	0	3.43E-29	5.56E-24
23	1.41E-29	3.81E-16	0	0	0	0	0	NA	NA	NA	NA	NA	NA	NA

24	0	3.86E-30	1.12E-27	1.42E-26	6.79E-30	4.12E-30	6.11E-28	3.14E-22	7.84E-29	5.67E-30	4.35E-29	5.73E-30	1.28E-25	0
25	5.94E-18	1.84E-25	1.30E-19	2.30E-24	7.02E-21	8.64E-21	9.82E-18	3.58E-23	NA	NA	NA	NA	NA	NA
26	6.86E-18	2.51E-30	4.27E-20	2.21E-21	5.20E-24	2.45E-27	4.31E-27	NA	4.47E-20	8.85E-24	6.82E-21	3.07E-24	9.22E-17	2.06E-21
27	5.31E-25	5.62E-29	2.98E-24	3.52E-25	2.59E-24	1.69E-25	1.71E-20	0	3.10E-24	7.83E-26	1.35E-24	2.00E-26	2.95E-26	0
28	1.14E-20	2.17E-30	0	2.19E-26	3.94E-27	7.55E-27	2.14E-23	2.44E-26	3.75E-23	1.69E-08	5.26E-17	2.09E-16	1.11E-24	1.07E-29
29	1.07E-21	4.65E-25	3.64E-29	1.97E-22	3.03E-20	2.61E-22	2.82E-21	NA	NA	NA	NA	NA	NA	NA
30	0	0.249306	0.003289	0.013351	7.93E-06	0.411684	5.84E-05	0.087995	0.011684	0.00075	4.95E-25	5.67E-10	2.59E-30	0.26913
31	1.89E-11	3.48E-20	1.13E-24	7.19E-23	4.04E-07	1.95E-11	3.66E-11	NA	5.46E-08	3.01E-12	4.61E-22	7.63E-11	1.29E-06	5.17E-09
32	2.39E-25	NA	NA	NA	NA	NA	NA	NA	NA	NA	8.61E-27	2.35E-30	5.54E-28	1.48E-19
33	0	0	2.09E-30	0	0	0	0	NA	NA	NA	NA	NA	NA	NA
34	4.87E-28	1.82E-13	6.29E-24	3.61E-15	9.43E-24	6.12E-23	5.11E-22	NA	NA	NA	NA	NA	NA	NA
35	2.04E-27	2.93E-30	0	5.96E-26	1.80E-19	1.13E-22	5.00E-23	NA	NA	NA	NA	NA	NA	NA
36	0	4.07E-27	0	3.15E-27	1.52E-27	2.14E-27	7.88E-26	NA	NA	NA	NA	NA	NA	NA
37	6.06E-29	1.40E-24	7.63E-28	1.64E-23	1.41E-28	1.62E-27	1.48E-24	NA	NA	NA	NA	NA	NA	NA
38	3.05E-23	4.87E-23	2.20E-30	1.54E-24	2.10E-17	9.90E-17	1.05E-13	NA	NA	NA	NA	NA	NA	NA
39	2.14E-25	0	9.93E-28	8.84E-12	3.36E-22	2.73E-13	1.85E-19	NA	NA	NA	NA	NA	NA	NA
40	1.68E-29	0	0	2.36E-29	0	0	0	NA	NA	NA	NA	NA	NA	NA
41	3.63E-27	2.09E-30	2.73E-28	3.08E-30	2.64E-30	2.04E-28	0	NA	NA	NA	NA	NA	NA	NA
42	1.57E-19	1.20E-29	6.64E-15	2.88E-18	1.76E-15	1.08E-15	7.48E-19	NA	NA	NA	NA	NA	NA	NA
43	5.03E-21	2.93E-22	7.17E-24	1.64E-20	7.69E-07	1.51E-28	2.80E-16	NA	NA	NA	NA	NA	NA	NA
44	2.13E-17	2.17E-30	3.01E-21	6.95E-23	1.16E-15	4.42E-15	3.19E-27	NA	NA	NA	NA	NA	NA	NA
45	2.51E-30	7.17E-26	1.01E-29	0	5.12E-28	2.05E-30	9.09E-30	NA	NA	NA	NA	NA	NA	NA
46	0	2.48E-23	1.57E-27	2.68E-22	1.14E-26	4.52E-30	1.42E-29	NA	NA	NA	NA	NA	NA	NA
47	1.19E-26	1.32E-26	1.04E-27	3.53E-26	1.44E-29	1.73E-28	0	NA	NA	NA	NA	NA	NA	NA
48	1.65E-20	3.26E-19	2.09E-16	2.65E-23	7.21E-23	4.59E-23	7.16E-23	NA	NA	NA	NA	NA	NA	NA
49	8.69E-29	8.61E-27	9.49E-30	1.04E-29	2.06E-30	3.55E-30	4.29E-29	NA	NA	NA	NA	NA	NA	NA
50	8.02E-25	4.69E-27	6.71E-30	2.33E-25	1.33E-25	4.48E-26	6.20E-27	NA	NA	NA	NA	NA	NA	NA
51	2.83E-20	4.23E-13	1.07E-17	1.13E-13	5.27E-14	1.42E-15	2.57E-12	NA	NA	NA	NA	NA	NA	NA
52	2.18E-30	7.28E-30	6.30E-29	1.70E-28	3.25E-29	8.74E-29	3.70E-22	NA	NA	NA	NA	NA	NA	NA
53	3.74E-24	2.32E-26	1.64E-28	9.29E-27	1.54E-29	4.54E-27	3.62E-23	NA	NA	NA	NA	NA	NA	NA
54	7.35E-25	1.49E-18	9.66E-13	7.08E-22	9.16E-13	1.21E-17	8.40E-17	NA	NA	NA	NA	NA	NA	NA
55	4.13E-29	7.43E-27	1.59E-29	1.20E-29	2.07E-29	1.77E-29	2.35E-27	NA	NA	NA	NA	NA	NA	NA
56	7.09E-20	3.13E-12	2.65E-22	2.29E-06	5.60E-07	5.84E-29	1.91E-21	NA	NA	NA	NA	NA	NA	NA
57	4.13E-22	4.51E-21	1.87E-24	4.35E-27	2.22E-19	1.14E-22	1.29E-24	NA	NA	NA	NA	NA	NA	NA
58	1.87E-21	3.29E-24	4.68E-26	3.42E-28	1.38E-23	6.71E-20	0	NA	NA	NA	NA	NA	NA	NA
59	1.15E-29	7.22E-28	4.24E-30	2.12E-30	0	3.95E-27	0	NA	NA	NA	NA	NA	NA	NA
60	2.06E-25	9.97E-21	1.35E-27	1.01E-21	1.51E-23	2.93E-29	7.58E-26	NA	NA	NA	NA	NA	NA	NA
61	2.28E-21	2.29E-26	9.03E-21	2.42E-28	5.30E-25	7.48E-26	3.91E-17	NA	NA	NA	NA	NA	NA	NA
62	1.14E-22	7.39E-22	2.32E-24	3.37E-25	4.07E-24	2.62E-18	1.32E-23	NA	NA	NA	NA	NA	NA	NA
63	4.65E-30	3.35E-26	0	0	2.44E-30	2.66E-30	0	NA	NA	NA	NA	NA	NA	NA
64	8.84E-30	3.02E-29	0	7.67E-30	2.14E-30	8.05E-27	3.79E-30	NA	NA	NA	NA	NA	NA	NA
65	4.66E-27	0	5.83E-30	1.19E-29	6.99E-30	8.71E-29	0	NA	NA	NA	NA	NA	NA	NA
66	7.28E-24	1.71E-24	1.23E-22	1.73E-27	1.07E-27	1.19E-25	4.61E-27	NA	NA	NA	NA	NA	NA	NA
67	2.66E-30	6.62E-21	4.60E-27	5.49E-30	1.99E-28	4.20E-18	5.71E-30	NA	NA	NA	NA	NA	NA	NA
68	0	2.05E-28	9.64E-30	2.26E-22	8.25E-24	7.35E-26	4.16E-21	NA	NA	NA	NA	NA	NA	NA
69	2.56E-16	0	3.64E-16	0	5.45E-20	1.08E-15	3.58E-19	NA	NA	NA	NA	NA	NA	NA
70	2.11E-06	1.35E-29	3.91E-29	2.99E-12	5.80E-07	2.98E-07	2.22E-10	NA	NA	NA	NA	NA	NA	NA
71	0	1.52E-29	0	0	0	0	0	NA	NA	NA	NA	NA	NA	NA
72	2.15E-30	3.95E-30	0	0	0	0	0	NA	NA	NA	NA	NA	NA	NA

73	6.27E-30	4.07E-27	1.35E-29	1.78E-29	1.00E-28	0	0	NA	NA	NA	NA	NA	NA	NA
74	0	2.03E-30	4.26E-30	0	0	0	0	NA	NA	NA	NA	NA	NA	NA
75	6.02E-29	3.81E-20	1.13E-13	1.71E-21	1.43E-16	1.71E-16	2.45E-17	NA	NA	NA	NA	NA	NA	NA
76	5.73E-29	1.01E-26	1.66E-23	5.01E-25	5.17E-29	4.56E-30	4.65E-27	NA	NA	NA	NA	NA	NA	NA
77	0	3.38E-28	2.29E-20	0	0	6.00E-27	2.14E-30	NA	NA	NA	NA	NA	NA	NA
78	1.15E-12	6.78E-24	2.72E-11	2.06E-30	1.03E-27	1.77E-27	4.78E-26	NA	NA	NA	NA	NA	NA	NA
79	2.16E-30	1.55E-23	1.48E-26	1.21E-18	9.88E-28	3.59E-17	6.01E-25	NA	NA	NA	NA	NA	NA	NA
80	0	1.49E-17	0	0	0	0	0	NA	NA	NA	NA	NA	NA	NA
81	1.50E-20	8.96E-30	0	4.50E-25	2.75E-17	1.14E-19	1.83E-20	NA	NA	NA	NA	NA	NA	NA
82	7.01E-19	2.77E-14	3.54E-26	8.75E-24	1.19E-22	3.90E-24	6.13E-27	NA	NA	NA	NA	NA	NA	NA
83	1.31E-29	4.17E-28	6.85E-30	1.89E-27	1.29E-27	8.70E-28	3.19E-26	NA	NA	NA	NA	NA	NA	NA
84	0	5.66E-26	0	3.13E-29	2.13E-24	1.75E-24	4.39E-28	NA	NA	NA	NA	NA	NA	NA
85	1.83E-20	0	6.91E-20	2.69E-25	0	5.80E-30	1.71E-19	3.51E-18	NA	NA	NA	NA	NA	NA
86	8.45E-25	9.37E-20	3.26E-19	4.21E-28	3.24E-28	4.18E-21	3.05E-26	NA	NA	NA	NA	NA	NA	NA
87	1.97E-27	3.57E-27	3.20E-27	2.02E-30	0	0	2.72E-28	NA	NA	NA	NA	NA	NA	NA
88	3.06E-27	0	6.28E-30	7.14E-22	5.07E-26	5.36E-19	5.50E-21	NA	NA	NA	NA	NA	NA	NA
89	3.65E-28	7.70E-07	9.13E-08	2.37E-13	1.83E-21	1.03E-22	3.17E-14	NA	NA	NA	NA	NA	NA	NA
90	4.72E-24	2.10E-19	4.28E-23	2.80E-22	1.08E-23	5.90E-22	2.56E-22	NA	NA	NA	NA	NA	NA	NA
91	3.46E-29	8.29E-29	7.35E-30	5.54E-30	7.39E-30	7.00E-30	9.35E-30	NA	NA	NA	NA	NA	NA	NA
92	8.95E-24	1.95E-27	0	6.05E-29	0	0	0	NA	NA	NA	NA	NA	NA	NA
93	1.93E-18	2.39E-20	1.75E-13	3.58E-14	1.26E-15	2.24E-15	2.35E-17	NA	NA	NA	NA	NA	NA	NA
94	0	2.27E-27	0	1.39E-27	6.28E-29	2.57E-28	2.32E-29	NA	NA	NA	NA	NA	NA	NA
95	2.34E-30	2.59E-30	0	0	2.82E-30	0	0	NA	NA	NA	NA	NA	NA	NA
96	8.96E-30	0	4.91E-27	0	7.58E-30	1.86E-29	0	NA	NA	NA	NA	NA	NA	NA
97	1.11E-24	8.28E-06	1.28E-05	1.32E-09	8.51E-10	1.78E-07	3.14E-07	NA	NA	NA	NA	NA	NA	NA
98	1.09E-14	1.63E-27	1.37E-28	2.82E-23	8.95E-21	3.71E-15	1.92E-15	NA	NA	NA	NA	NA	NA	NA
99	1.20E-26	0	2.72E-24	7.06E-28	2.15E-27	2.04E-26	3.73E-20	0	NA	NA	NA	NA	NA	NA
100	1.21E-29	0	4.13E-29	2.14E-30	3.74E-28	6.26E-29	1.17E-28	5.84E-28	NA	NA	NA	NA	NA	NA
101	5.08E-28	0	8.51E-23	2.03E-30	9.82E-29	4.26E-29	1.36E-26	2.23E-27	NA	NA	NA	NA	NA	NA
102	0	3.59E-29	1.56E-16	2.32E-20	1.05E-26	3.17E-25	1.66E-29	5.48E-24	NA	NA	NA	NA	NA	NA
103	2.86E-26	0	9.68E-27	2.24E-27	5.56E-27	8.41E-27	2.66E-27	2.05E-30	NA	NA	NA	NA	NA	NA
104	1.30E-26	1.88E-24	3.45E-12	4.75E-26	1.01E-21	4.86E-18	3.59E-24	NA	NA	NA	NA	NA	NA	NA
105	1.80E-24	6.01E-26	0	0	0	1.88E-29	0	0	NA	NA	NA	NA	NA	NA
106	0	0	2.46E-30	0	0	0	0	2.02E-30	NA	NA	NA	NA	NA	NA
107	5.88E-21	0	2.68E-30	3.47E-30	2.43E-30	6.55E-30	2.55E-30	0	NA	NA	NA	NA	NA	NA
108	4.76E-25	5.23E-30	3.98E-30	0	0	3.78E-30	0	0	NA	NA	NA	NA	NA	NA
109	0	2.27E-26	0	4.13E-26	3.33E-27	1.70E-29	1.67E-21	NA	NA	NA	NA	NA	NA	NA
110	7.66E-30	0	2.10E-30	1.25E-29	5.33E-27	3.79E-29	7.36E-28	2.99E-25	NA	NA	NA	NA	NA	NA
111	7.80E-19	0	1.12E-23	0	2.69E-16	3.00E-29	2.27E-30	1.12E-26	5.38E-27	3.10E-30	2.78E-25	0	0	0
112	5.69E-26	6.00E-20	1.16E-20	0	1.92E-25	1.22E-21	1.77E-28	4.71E-30	NA	0	NA	2.60E-27	NA	6.56E-25
113	3.72E-27	7.46E-30	5.59E-28	1.59E-26	4.54E-27	2.16E-28	1.10E-22	NA	3.22E-24	1.20E-24	8.85E-25	3.32E-25	0	0
114	1.78E-27	1.15E-12	8.86E-06	3.33E-09	2.73E-09	8.36E-24	1.34E-11	6.19E-25	NA	NA	NA	NA	NA	NA
115	2.13E-30	0	3.65E-30	0	0	2.30E-30	0	NA	NA	NA	NA	NA	NA	NA
116	0	3.48E-28	3.87E-25	1.34E-27	7.05E-28	1.38E-26	0	8.75E-18	NA	NA	NA	NA	NA	NA
117	0	0.066324	2.22E-13	0	0	1.11E-12	2.02E-30	0	NA	NA	NA	NA	NA	NA
118	1.77E-08	0.000468	5.93E-06	0.016074	1.96E-14	2.62E-05	3.11E-07	0.569972	NA	NA	NA	NA	NA	NA
119	3.78E-26	6.00E-24	5.42E-26	6.29E-21	3.96E-28	1.89E-28	5.72E-19	1.94E-21	NA	NA	NA	NA	NA	NA
120	1.11E-28	3.75E-30	3.00E-30	0	7.64E-27	9.43E-30	0	0.030706	4.45E-27	1.24E-16	4.72E-24	1.32E-23	1.58E-22	0
121	1.20E-22	2.74E-30	9.80E-22	4.46E-23	2.11E-28	4.12E-30	2.22E-30	2.16E-19	1.80E-29	7.92E-30	5.46E-25	2.23E-30	0	0

122	1.27E-27	8.89E-30	3.64E-28	4.75E-30	2.28E-30	4.31E-30	5.40E-27	1.56E-24	1.52E-19	9.55E-29	1.08E-28	1.05E-28	3.66E-19	2.51E-21
123	4.92E-24	0	1.01E-22	0	9.64E-29	0	2.10E-30	8.66E-08	8.43E-25	2.73E-25	6.16E-27	2.12E-23	0	4.94E-19
124	3.15E-17	2.50E-13	7.03E-18	1.16E-16	8.30E-20	1.15E-15	2.72E-18	NA	NA	NA	NA	NA	NA	NA
125	3.90E-23	6.04E-20	4.08E-23	0	2.82E-29	5.93E-29	0	NA	1.85E-21	1.13E-23	9.92E-22	1.07E-19	2.64E-24	2.34E-27
126	2.45E-17	5.01E-24	2.08E-25	1.10E-23	1.70E-27	4.45E-29	1.40E-28	NA	NA	2.08E-18	NA	0	NA	5.97E-27
127	2.70E-14	2.71E-06	1.01E-09	1.82E-15	4.53E-25	1.88E-25	5.39E-16	NA	NA	NA	NA	NA	NA	NA
128	1.27E-23	9.25E-23	2.38E-23	7.21E-24	2.49E-27	4.10E-27	7.89E-23	NA	NA	NA	NA	NA	NA	NA
129	0	1.19E-14	2.09E-30	4.77E-25	4.92E-20	1.11E-21	4.26E-17	NA	2.41E-23	1.04E-14	2.17E-17	8.12E-16	0	2.93E-18
130	2.23E-15	1.28E-23	5.74E-25	3.05E-23	9.76E-23	1.29E-21	1.28E-25	NA	1.46E-10	2.45E-16	3.71E-16	3.85E-11	1.08E-22	5.75E-24
131	0	2.11E-30	0	3.32E-30	0	0	0	NA	0	0	2.19E-30	0	0	0
132	1.50E-20	1.89E-11	4.16E-20	4.66E-18	6.61E-24	1.13E-24	1.87E-23	NA	NA	NA	NA	NA	NA	NA
133	5.58E-29	4.75E-18	3.33E-27	7.68E-24	2.34E-23	3.07E-15	3.37E-15	NA	NA	NA	NA	NA	NA	NA
134	0	5.24E-23	0	1.27E-18	7.83E-24	1.75E-22	8.24E-23	NA	NA	NA	NA	NA	NA	NA
135	7.08E-19	3.33E-14	2.72E-20	1.33E-24	1.56E-28	0	1.14E-13	NA	NA	NA	NA	NA	NA	NA
136	2.32E-27	4.41E-24	1.06E-26	1.46E-23	2.38E-17	5.78E-20	2.20E-24	NA	NA	NA	NA	NA	NA	NA
137	1.75E-10	2.50E-27	0	4.46E-22	1.25E-11	1.60E-07	4.53E-05	NA	NA	NA	NA	NA	NA	NA
138	2.32E-30	0	0	6.15E-30	0	0	0	NA	NA	NA	NA	NA	NA	NA
139	2.16E-14	0.006379	8.52E-15	4.10E-07	0.009671	6.67E-14	5.43E-08	NA	NA	NA	NA	NA	NA	NA
140	5.35E-27	5.77E-11	8.28E-27	2.36E-29	1.19E-23	1.71E-27	2.04E-30	NA	NA	NA	NA	NA	NA	NA
141	3.04E-09	2.71E-12	1.26E-10	4.36E-24	8.76E-23	6.13E-24	6.84E-20	NA	NA	NA	NA	NA	NA	NA
142	1.17E-26	4.10E-12	5.97E-26	1.01E-13	1.82E-13	7.00E-10	0.172732	NA	NA	NA	NA	NA	NA	NA
143	0	7.01E-22	3.75E-30	7.65E-29	5.90E-29	4.04E-26	0	NA	NA	NA	NA	NA	NA	NA
144	5.26E-26	2.62E-22	2.61E-28	6.95E-29	8.22E-24	2.47E-27	2.22E-25	NA	NA	NA	NA	NA	NA	NA
145	5.50E-26	2.55E-22	0	1.23E-25	9.57E-25	4.44E-25	5.40E-27	NA	NA	NA	NA	NA	NA	NA
146	3.87E-29	1.73E-23	6.72E-23	3.70E-30	9.09E-30	2.80E-29	4.46E-29	NA	NA	NA	NA	NA	NA	NA
147	0.000145	1.50E-05	2.84E-06	8.33E-12	2.57E-24	2.51E-13	0.003796	NA	NA	NA	NA	NA	NA	NA
148	1.45E-26	6.60E-18	1.13E-27	1.45E-19	3.92E-29	4.27E-28	0	NA	NA	NA	NA	NA	NA	NA
149	8.32E-12	8.64E-26	3.39E-12	1.35E-27	1.70E-10	8.78E-17	1.67E-26	NA	NA	NA	NA	NA	NA	NA
150	6.77E-26	2.06E-17	0	1.95E-19	1.74E-28	5.38E-29	0	NA	NA	NA	NA	NA	NA	NA
151	2.77E-07	1.02E-28	2.78E-24	6.47E-14	5.52E-10	3.83E-10	1.47E-11	NA	NA	NA	NA	NA	NA	NA
152	7.54E-21	2.30E-08	4.27E-29	7.28E-19	1.69E-20	4.30E-18	2.15E-17	NA	NA	NA	NA	NA	NA	NA
153	6.80E-25	2.21E-18	0	1.46E-20	1.87E-20	2.18E-20	2.96E-30	NA	1.68E-27	1.11E-23	1.91E-29	3.46E-29	0	4.03E-30
154	4.81E-19	0	5.07E-17	4.44E-23	8.56E-24	5.35E-25	1.87E-24	NA	NA	NA	NA	NA	NA	NA
155	1.27E-23	6.96E-19	3.61E-21	7.83E-20	9.19E-15	5.84E-16	6.36E-30	NA	NA	NA	NA	NA	NA	NA
156	5.25E-21	5.91E-06	1.14E-10	7.20E-10	0.000298	4.12E-12	5.10E-13	NA	NA	NA	NA	NA	NA	NA
157	1.26E-17	4.38E-12	8.46E-23	2.71E-18	2.21E-22	1.83E-24	7.82E-21	NA	NA	NA	NA	NA	NA	NA
158	0.02092	0.994036	0.213321	0.831482	0.891314	0.697335	0.957126	NA	0.127715	0.019083	0.873906	0.434384	0.224137	0.000506
159	3.77E-13	0.00012	3.43E-11	1.02E-09	2.97E-07	5.84E-06	0.000345	NA	3.80E-14	9.33E-15	7.70E-15	5.57E-13	0.014967	1.61E-06
160	8.30E-16	1.16E-16	5.30E-15	2.97E-30	7.51E-20	4.71E-20	1.21E-19	NA	NA	2.35E-25	NA	2.74E-29	NA	2.16E-30
161	6.02E-30	2.18E-21	6.61E-29	7.77E-14	4.85E-24	5.16E-28	3.30E-28	NA	NA	NA	NA	NA	NA	NA
162	3.57E-28	1.03E-28	1.25E-27	1.68E-18	2.18E-17	1.14E-17	3.91E-15	NA	NA	NA	NA	NA	NA	NA
163	3.33E-19	2.43E-11	9.72E-30	4.82E-23	4.05E-21	1.46E-26	1.84E-09	NA	NA	NA	NA	NA	NA	NA
164	0	8.28E-07	0	2.61E-12	7.91E-16	3.87E-16	6.08E-19	NA	NA	NA	NA	NA	NA	NA
165	9.38E-29	5.25E-29	1.04E-18	5.62E-09	1.30E-08	5.96E-17	3.85E-13	NA	NA	NA	NA	NA	NA	NA
166	4.75E-26	5.30E-25	4.33E-24	6.14E-24	0	0	1.32E-23	NA	NA	NA	NA	NA	NA	NA
167	5.72E-30	4.07E-29	8.72E-30	4.12E-27	2.44E-27	0	1.25E-27	NA	1.18E-25	5.79E-26	1.69E-25	3.28E-28	0	0
168	1.65E-21	1.73E-08	6.74E-19	5.50E-14	3.38E-23	3.67E-25	1.29E-10	NA	2.59E-20	NA	8.00E-21	NA	9.94E-30	NA
169	1.38E-12	9.64E-11	4.03E-15	3.06E-10	9.58E-27	2.71E-21	4.76E-30	NA	1.65E-28	7.23E-14	4.59E-29	2.72E-19	7.04E-26	3.33E-19
170	2.79E-14	4.18E-13	1.21E-13	2.61E-26	2.70E-28	5.60E-28	2.08E-13	NA	NA	NA	NA	NA	NA	NA

171	4.27E-09	3.40E-30	0	6.88E-10	1.65E-09	2.29E-11	9.10E-07	NA	3.78E-16	4.35E-17	6.66E-27	2.87E-16	0.003992	2.72E-12
172	4.27E-29	1.19E-24	0	9.24E-22	1.28E-16	8.43E-19	2.00E-29	NA	1.39E-28	1.59E-22	4.15E-21	1.01E-22	7.01E-26	6.14E-30
173	3.27E-19	2.53E-20	8.10E-30	4.69E-24	2.63E-23	1.95E-23	2.88E-14	NA	NA	NA	NA	NA	NA	NA
174	0	9.81E-26	8.53E-26	2.58E-19	2.81E-28	2.60E-30	9.16E-23	NA	3.74E-29	7.48E-29	0	7.27E-23	1.67E-26	0
175	0	7.69E-25	0	0	1.57E-27	7.76E-26	7.72E-30	NA	NA	NA	NA	NA	NA	NA
176	1.18E-11	2.09E-12	1.75E-10	3.87E-15	1.72E-17	5.54E-15	1.44E-17	NA	NA	NA	NA	NA	NA	NA
177	1.68E-28	1.80E-28	1.40E-29	1.39E-19	3.28E-25	2.35E-13	2.81E-19	NA	NA	NA	NA	NA	NA	NA
178	5.52E-07	0.264256	2.06E-07	0.488587	0.001893	2.14E-05	0.063295	NA	NA	NA	NA	NA	NA	NA
179	0	1.25E-20	0	3.87E-25	3.04E-26	2.06E-25	5.10E-28	NA	NA	NA	NA	NA	NA	NA
180	1.45E-12	4.90E-16	1.76E-16	3.99E-13	3.37E-13	2.06E-09	1.63E-20	NA	NA	NA	NA	NA	NA	NA
181	7.60E-24	0	2.80E-17	6.31E-25	1.32E-11	4.49E-13	3.38E-14	NA	NA	NA	NA	NA	NA	NA
182	1.51E-23	2.23E-24	1.52E-23	1.87E-29	2.31E-24	1.05E-26	1.57E-26	NA	NA	NA	NA	NA	NA	NA
183	5.37E-27	4.37E-10	5.91E-28	1.35E-08	7.57E-24	3.69E-19	1.28E-16	NA	NA	NA	NA	NA	NA	NA
184	3.32E-28	3.51E-25	2.39E-29	2.21E-26	5.58E-23	2.47E-25	3.53E-26	NA	NA	NA	NA	NA	NA	NA
185	2.68E-13	2.30E-11	9.42E-30	1.94E-20	5.26E-17	5.28E-22	2.01E-16	NA	NA	NA	NA	NA	NA	NA
186	1.09E-12	4.88E-21	9.48E-13	7.60E-22	2.05E-18	1.14E-17	1.47E-07	NA	NA	NA	NA	NA	NA	NA
187	3.31E-30	9.57E-11	1.21E-16	1.48E-11	3.92E-16	2.69E-20	6.03E-16	NA	NA	6.65E-25	NA	1.25E-17	NA	0
188	1.33E-29	3.66E-17	8.77E-29	2.50E-20	1.20E-20	2.69E-23	1.48E-26	NA	8.15E-21	NA	1.10E-25	NA	5.79E-20	NA
189	6.10E-28	1.49E-16	9.17E-15	5.30E-16	2.07E-10	1.35E-20	1.02E-17	NA	6.14E-14	2.67E-26	2.02E-21	2.05E-25	3.05E-08	1.17E-09
190	9.47E-11	2.04E-14	2.28E-20	8.74E-15	3.68E-11	4.44E-14	7.41E-11	NA	NA	NA	NA	NA	NA	NA
191	2.86E-10	5.71E-17	7.46E-10	8.28E-10	3.10E-13	5.42E-15	1.06E-12	NA	1.99E-20	1.95E-13	2.85E-14	9.28E-11	1.77E-15	2.51E-07
192	2.91E-13	1.19E-12	2.80E-14	1.05E-12	2.07E-10	3.91E-14	2.48E-16	NA	1.39E-15	7.24E-10	8.17E-06	3.87E-10	1.94E-16	7.55E-15
193	0	5.58E-27	0	2.19E-30	2.35E-30	5.21E-30	4.50E-24	NA	0	5.84E-28	0	2.22E-26	7.72E-15	1.48E-13
194	3.04E-27	0	9.13E-27	4.94E-28	1.78E-25	1.95E-25	3.43E-21	NA	NA	NA	NA	NA	NA	NA
195	3.58E-29	4.56E-21	6.31E-17	5.21E-29	0	0	2.99E-23	NA	2.58E-08	7.68E-18	2.97E-07	1.79E-13	1.26E-06	5.43E-29
196	0	2.26E-18	0	3.41E-25	7.77E-24	8.91E-22	9.69E-25	NA	NA	NA	NA	NA	NA	NA
197	9.40E-22	1.37E-25	5.33E-14	1.69E-25	1.59E-19	8.22E-20	1.63E-29	NA	NA	0.000934	NA	0.000837	0.000722	2.99E-07
198	9.62E-16	1.49E-25	1.21E-19	2.07E-20	1.82E-17	1.22E-18	1.96E-24	NA	2.91E-10	2.21E-25	5.21E-12	1.07E-24	1.86E-06	2.16E-23
199	3.87E-28	0	3.54E-28	9.15E-25	4.26E-28	1.65E-28	7.50E-21	NA	6.47E-17	7.02E-16	1.29E-15	2.68E-20	2.12E-08	1.40E-11
200	3.00E-18	3.91E-17	1.79E-07	1.69E-07	3.79E-12	1.71E-08	2.81E-20	NA	5.12E-06	1.14E-07	8.68E-23	2.41E-15	0	0
201	1.13E-19	1.57E-21	5.54E-24	1.66E-19	7.82E-20	3.03E-21	3.02E-19	NA	6.59E-26	3.90E-22	2.21E-24	6.18E-23	7.49E-29	2.40E-30
202	4.36E-14	2.64E-14	6.83E-21	0	0	0	1.81E-29	NA	1.55E-25	0	1.47E-25	7.07E-30	0	0
203	0	2.60E-17	0	2.15E-16	3.72E-26	1.50E-16	7.08E-24	NA	1.69E-17	NA	4.01E-20	NA	2.91E-17	NA
204	1.38E-10	1.78E-05	6.04E-07	6.46E-11	0.078502	1.78E-07	1.89E-11	NA	0.434353	3.33E-06	7.73E-15	6.03E-17	2.51E-22	1.49E-29
205	4.06E-27	2.25E-06	4.98E-26	2.05E-22	8.65E-21	2.26E-24	1.71E-21	NA	2.40E-29	5.07E-18	1.51E-13	3.00E-15	1.44E-26	5.32E-28
206	5.36E-11	1.39E-13	3.51E-10	2.37E-16	8.58E-12	6.35E-12	3.33E-11	NA	3.60E-18	1.38E-12	3.90E-18	1.13E-19	3.42E-30	1.86E-20
207	3.53E-29	9.64E-17	2.48E-30	8.01E-13	8.36E-20	3.59E-20	9.81E-22	NA	1.01E-13	4.03E-23	1.12E-16	2.83E-14	3.15E-15	2.32E-30
208	8.95E-25	6.44E-30	7.87E-30	6.28E-30	7.72E-26	5.14E-30	2.54E-29	NA	0	5.21E-18	4.51E-23	2.47E-20	8.37E-19	0
209	8.02E-23	5.73E-28	1.51E-21	1.59E-21	3.41E-26	4.64E-27	1.39E-19	NA	7.77E-24	8.88E-21	4.39E-25	7.98E-24	1.19E-23	9.30E-20
210	1.28E-22	2.58E-30	9.07E-30	6.96E-26	9.05E-24	2.16E-19	3.98E-30	NA	4.39E-26	1.11E-24	3.64E-26	4.26E-24	7.83E-26	0
211	6.20E-14	5.98E-21	2.98E-16	3.81E-23	2.04E-20	3.25E-21	9.59E-17	NA	7.85E-27	3.18E-24	1.06E-24	1.26E-22	1.11E-28	5.93E-15
212	1.42E-18	0	3.00E-18	1.08E-21	1.55E-26	6.41E-20	2.82E-21	NA	9.45E-23	2.04E-30	4.64E-21	2.21E-30	2.07E-24	2.74E-10
213	3.96E-14	2.90E-30	3.01E-14	5.43E-30	2.54E-29	7.67E-29	3.88E-19	NA	4.77E-22	4.74E-19	3.24E-22	5.46E-25	0	0
214	8.93E-16	2.61E-29	1.36E-20	2.04E-23	6.10E-23	2.04E-18	8.21E-10	NA	1.65E-19	1.96E-17	5.81E-20	1.70E-23	3.22E-28	1.80E-15
215	6.95E-15	1.09E-29	1.90E-15	1.76E-19	5.25E-19	4.24E-19	2.92E-16	NA	7.52E-16	1.63E-14	1.31E-23	1.85E-20	2.78E-16	7.43E-19
216	2.75E-20	2.92E-22	2.59E-30	2.42E-17	9.76E-22	3.51E-18	4.14E-12	NA	2.06E-11	1.73E-19	2.21E-24	5.75E-20	1.27E-28	2.50E-28
217	3.37E-19	2.49E-19	9.69E-20	7.32E-16	3.76E-17	2.75E-20	1.80E-15	NA	2.00E-24	3.07E-24	1.48E-21	8.87E-23	2.46E-30	2.62E-30
218	8.68E-29	7.68E-15	5.12E-11	2.93E-11	5.16E-14	1.13E-13	2.43E-09	NA	2.94E-12	4.76E-14	3.37E-11	1.05E-12	7.40E-22	4.11E-20
219	0	1.05E-09	0	1.14E-23	6.99E-15	1.46E-22	2.89E-10	NA	1.45E-16	4.49E-13	4.38E-14	8.70E-12	2.45E-09	1.89E-07

220	5.32E-15	8.28E-10	5.16E-18	4.85E-08	8.99E-08	2.41E-16	5.39E-13	NA	NA	NA	NA	NA	NA	NA
221	4.60E-24	1.62E-15	1.04E-23	3.15E-27	2.61E-22	2.83E-22	6.37E-21	NA	9.13E-27	5.17E-22	6.29E-20	2.09E-26	0	5.77E-24
222	9.43E-17	1.53E-22	2.27E-25	2.47E-07	1.47E-14	1.96E-18	5.32E-18	NA	5.16E-13	2.47E-13	2.12E-23	2.19E-13	6.13E-17	7.24E-25
223	1.32E-17	1.53E-06	3.24E-19	2.17E-19	1.43E-12	2.88E-12	9.58E-09	NA	6.71E-12	1.27E-13	5.81E-12	1.84E-14	9.44E-22	4.20E-30
224	1.08E-16	6.00E-28	6.47E-19	7.93E-15	4.22E-16	4.96E-16	1.27E-10	NA	3.17E-13	5.98E-22	3.21E-12	1.08E-21	5.50E-06	1.88E-21
225	1.61E-21	5.58E-18	7.57E-21	9.39E-25	9.65E-18	1.00E-18	4.89E-18	NA	7.54E-17	2.66E-20	2.50E-16	5.18E-21	2.90E-15	9.31E-24
226	7.50E-16	9.26E-15	1.20E-08	2.19E-06	2.61E-12	1.35E-14	3.61E-14	NA	5.26E-14	9.11E-09	4.02E-15	2.06E-11	8.39E-17	3.44E-22
227	1.70E-18	1.49E-21	5.99E-20	9.56E-28	2.79E-24	1.32E-24	5.37E-20	NA	3.74E-30	0	2.20E-30	1.65E-18	5.71E-16	2.06E-15
228	4.95E-30	0.214717	0.002714	0.632682	0.540164	6.11E-06	0.97978	NA	4.14E-25	0.974232	0.026525	1.60E-09	2.25E-12	2.97E-08
229	2.61E-29	0	0	0	0	0	0	NA	0	2.94E-30	6.56E-30	1.53E-27	3.36E-29	0
230	1.35E-23	1.96E-29	4.80E-14	5.01E-22	5.86E-22	3.01E-26	2.41E-27	NA	1.42E-28	1.04E-27	1.76E-29	1.19E-21	0	0
231	2.94E-09	1.14E-23	8.23E-08	3.12E-14	1.55E-17	1.12E-17	1.30E-24	NA	1.69E-10	1.68E-13	1.10E-16	5.22E-11	7.24E-26	0
232	6.02E-15	2.75E-16	7.53E-20	5.99E-13	1.96E-14	7.25E-16	1.49E-21	NA	1.76E-22	1.49E-19	1.11E-15	1.73E-14	0	2.12E-29
233	3.37E-22	2.93E-21	4.08E-14	3.64E-11	8.69E-11	3.90E-09	4.72E-12	NA	7.48E-14	NA	1.13E-13	NA	1.32E-26	NA
234	5.42E-13	6.64E-17	1.72E-08	4.99E-15	2.62E-13	3.11E-27	1.45E-15	5.34E-26	1.88E-10	1.64E-11	1.92E-09	1.22E-10	1.80E-19	2.28E-20
235	7.13E-29	4.88E-23	5.51E-24	8.95E-18	0	6.23E-15	1.13E-22	9.94E-29	NA	NA	NA	NA	NA	NA
236	1.52E-25	6.81E-19	6.13E-10	7.10E-14	1.89E-10	2.79E-07	1.01E-07	2.90E-14	1.43E-20	1.21E-08	0.002327	2.48E-25	2.71E-22	1.89E-26
237	5.61E-17	2.61E-10	1.98E-17	1.52E-21	4.60E-25	7.80E-24	6.98E-09	NA	1.06E-14	NA	1.54E-18	NA	6.56E-20	NA
238	1.12E-22	3.60E-30	9.51E-23	6.07E-24	5.33E-18	5.37E-25	3.83E-22	NA	1.73E-29	1.14E-24	1.57E-20	1.06E-23	0	0
239	1.20E-26	5.29E-17	9.20E-26	2.55E-14	6.39E-12	3.63E-17	6.71E-12	5.09E-15	1.51E-13	8.34E-13	2.31E-06	1.86E-14	9.70E-17	8.53E-22
240	7.99E-19	2.02E-30	1.32E-18	4.85E-24	1.43E-19	1.50E-25	3.80E-19	NA	1.04E-28	1.32E-19	2.53E-29	2.78E-19	0	0
241	3.45E-24	4.74E-27	2.74E-30	7.24E-29	4.44E-30	2.78E-28	2.18E-26	6.65E-26	1.05E-24	3.13E-28	3.24E-24	3.93E-24	0	0
242	4.31E-15	1.01E-10	1.07E-14	1.01E-08	6.56E-07	9.41E-08	3.97E-06	NA	9.59E-12	8.63E-10	2.42E-13	3.93E-10	1.90E-08	3.21E-09
243	0	1.11E-28	3.22E-30	1.52E-28	2.23E-29	3.39E-29	5.68E-30	NA	0	3.54E-30	3.56E-29	3.01E-30	5.76E-26	0
244	3.78E-08	2.02E-30	9.00E-19	5.08E-18	1.17E-26	4.16E-26	4.04E-16	NA	9.41E-17	7.55E-19	2.97E-19	4.07E-14	1.87E-13	2.40E-15
245	7.32E-23	1.91E-29	0	8.57E-24	1.35E-29	6.87E-29	2.79E-29	6.24E-28	6.78E-14	3.25E-29	4.03E-12	3.62E-30	6.54E-24	0
246	3.16E-18	1.69E-17	6.76E-19	2.43E-22	5.60E-19	3.12E-18	4.25E-24	NA	4.24E-26	5.67E-25	5.88E-26	4.24E-25	8.15E-07	0
247	1.12E-24	0	7.25E-22	5.29E-22	1.18E-14	9.63E-14	2.41E-14	2.57E-15	4.06E-13	NA	3.19E-12	NA	1.76E-23	NA
248	1.06E-21	0	0	3.95E-28	9.53E-23	3.50E-22	1.30E-26	NA	1.16E-28	1.81E-29	2.83E-28	6.22E-30	1.75E-26	1.16E-26
249	7.16E-19	7.88E-25	3.06E-21	9.96E-21	3.52E-22	3.32E-14	3.00E-19	0.053221	6.51E-19	3.85E-22	2.56E-17	6.51E-10	1.01E-16	3.40E-10
250	3.30E-27	5.86E-26	1.14E-18	1.00E-08	1.95E-11	2.98E-22	2.21E-16	0	1.52E-12	1.50E-06	2.63E-17	1.87E-18	0	3.79E-25
251	7.22E-29	3.19E-27	2.17E-29	2.73E-15	9.82E-18	1.67E-17	1.71E-17	NA	NA	8.97E-29	NA	8.89E-28	NA	5.72E-29
252	2.28E-30	3.18E-28	0	9.87E-28	3.87E-29	9.91E-29	4.72E-26	3.43E-28	1.05E-26	2.16E-28	1.05E-21	3.10E-26	5.68E-28	0
253	1.75E-28	2.34E-25	3.88E-27	7.17E-26	1.86E-28	5.74E-26	6.10E-30	9.79E-24	6.89E-30	6.96E-29	6.99E-30	1.14E-28	0	6.79E-30
254	1.32E-12	6.86E-25	8.79E-24	1.84E-06	5.84E-07	1.08E-09	3.78E-14	NA	2.58E-12	5.33E-17	1.82E-12	3.60E-17	8.69E-08	2.15E-19
255	2.02E-30	9.01E-15	1.59E-27	4.17E-23	1.44E-13	1.86E-18	2.93E-18	NA	9.70E-15	NA	4.47E-17	NA	8.69E-17	NA
256	1.46E-18	0	1.21E-19	1.20E-18	6.26E-24	2.08E-18	1.64E-17	NA	5.29E-26	4.49E-18	6.94E-10	6.41E-18	1.17E-08	2.76E-25
257	6.12E-29	1.34E-26	1.75E-26	1.29E-26	2.59E-26	8.60E-25	3.64E-23	NA	1.48E-14	2.61E-25	5.78E-13	3.37E-25	1.72E-20	0
258	1.11E-28	7.37E-24	1.08E-27	8.75E-29	3.47E-28	3.74E-28	1.02E-28	NA	1.31E-29	7.19E-29	2.17E-29	5.98E-30	3.26E-15	0
259	1.13E-24	0	5.85E-29	4.09E-30	8.07E-28	2.02E-28	2.97E-27	NA	7.84E-26	5.98E-28	7.97E-26	3.32E-27	0	1.05E-22
260	7.63E-30	3.98E-30	5.91E-30	9.77E-30	8.31E-30	3.31E-30	1.15E-29	2.68E-30	1.34E-28	4.97E-30	1.82E-28	3.63E-30	5.24E-27	0
261	3.54E-30	0	2.39E-30	4.36E-16	5.76E-25	2.34E-24	3.59E-28	NA	4.55E-27	5.59E-30	5.31E-13	5.63E-30	9.84E-21	5.72E-18
262	2.75E-20	3.65E-27	4.39E-23	6.13E-24	1.43E-26	3.90E-22	4.35E-27	NA	0.064155	2.61E-26	0	1.21E-16	NA	6.77E-23
263	1.96E-26	2.65E-27	2.73E-27	1.00E-25	7.65E-30	1.09E-28	5.51E-30	0	7.68E-29	5.24E-28	1.88E-28	1.83E-27	0	7.23E-26
264	2.71E-21	2.89E-28	5.83E-24	3.13E-28	1.39E-25	9.12E-30	3.39E-29	0	9.78E-26	2.57E-25	2.39E-26	2.45E-25	4.35E-23	1.19E-23
265	2.06E-30	5.88E-30	0	5.76E-29	6.92E-29	1.04E-28	7.91E-27	2.14E-26	3.12E-30	4.98E-22	2.35E-23	3.06E-28	0	6.49E-29
266	1.75E-25	3.42E-28	8.60E-26	7.23E-23	2.07E-18	2.49E-19	3.47E-13	NA	1.63E-06	NA	2.64E-11	NA	2.72E-21	NA
267	7.75E-26	0	8.38E-26	2.74E-24	2.46E-22	3.03E-22	9.56E-24	NA	1.00E-28	5.44E-28	1.53E-28	6.60E-28	9.90E-24	0
268	2.29E-29	0.001264	0.000298	1.11E-09	0.02743	0.035169	0.000806	NA	NA	NA	3.26E-21	9.85E-12	3.90E-15	5.07E-16

269	6.79E-21	2.54E-20	1.50E-20	2.25E-25	2.80E-25	8.07E-23	4.85E-27	NA	3.96E-21	2.26E-17	8.73E-21	3.88E-18	1.69E-20	0
270	4.78E-21	1.29E-21	3.49E-23	2.92E-23	4.66E-24	4.43E-25	2.56E-24	NA	1.34E-22	8.56E-22	4.31E-23	1.15E-21	2.63E-29	6.01E-30
271	1.20E-27	2.13E-30	7.73E-26	2.27E-23	1.42E-21	8.60E-23	9.91E-25	NA	1.73E-19	4.34E-18	5.76E-20	2.38E-19	8.71E-30	5.35E-29
272	2.97E-23	7.73E-24	7.57E-29	8.37E-27	5.77E-26	1.29E-23	0	NA	9.06E-24	5.93E-23	3.93E-29	1.87E-23	1.12E-21	8.01E-23
273	3.71E-30	1.95E-28	2.94E-30	1.90E-11	5.33E-16	3.46E-14	7.35E-14	NA	5.60E-11	1.34E-16	1.68E-13	7.27E-18	1.01E-14	1.52E-06
274	2.04E-30	3.56E-24	0	3.63E-28	1.16E-16	4.41E-18	2.30E-24	NA	5.94E-26	3.37E-16	1.39E-23	4.84E-20	5.85E-30	0
275	5.46E-24	2.93E-27	0	1.41E-29	6.29E-30	5.70E-27	4.27E-29	0	9.28E-30	2.44E-29	1.04E-29	1.85E-29	8.35E-30	0
276	8.51E-21	2.59E-25	2.38E-20	5.31E-20	1.25E-22	1.00E-22	2.10E-23	NA	7.17E-28	7.33E-25	1.03E-27	3.14E-16	2.20E-30	0
277	2.09E-16	1.08E-21	3.37E-20	5.68E-18	2.84E-22	3.37E-23	2.34E-30	NA	1.78E-24	5.38E-26	4.87E-25	5.50E-24	3.53E-25	0
278	1.48E-23	3.53E-27	8.91E-29	8.80E-26	3.90E-28	5.86E-20	7.30E-25	NA	1.96E-21	2.44E-28	1.49E-22	8.64E-29	2.45E-30	3.96E-07
279	1.09E-27	2.15E-30	9.62E-25	1.14E-20	2.29E-20	2.45E-18	2.11E-23	NA	1.68E-25	9.89E-18	4.89E-20	5.89E-28	2.21E-27	0
280	5.33E-20	9.69E-14	1.75E-19	6.84E-20	2.34E-20	5.38E-19	4.54E-19	NA	6.03E-16	8.89E-17	2.83E-13	6.43E-17	4.76E-30	1.69E-11
281	2.01E-07	9.74E-11	0.009298	0.782949	4.65E-07	5.50E-08	5.09E-08	NA	7.33E-11	0.009093	8.62E-07	0.003812	4.24E-13	0.096569
282	1.80E-27	3.41E-18	5.45E-27	2.54E-30	5.46E-29	0	1.71E-21	NA	3.49E-25	3.05E-29	5.78E-29	8.79E-17	4.61E-19	0
283	1.84E-18	1.13E-28	2.27E-18	1.52E-24	8.57E-22	2.36E-30	8.30E-25	NA	NA	NA	NA	NA	NA	NA
284	4.63E-27	7.85E-25	3.76E-26	2.27E-19	3.43E-26	1.34E-26	3.92E-27	NA	NA	NA	NA	NA	NA	NA
285	1.06E-25	1.40E-16	1.23E-29	0	2.90E-26	2.11E-26	2.37E-30	NA	7.43E-28	0	0	0	7.04E-29	0
286	1.35E-21	5.28E-26	7.64E-15	4.40E-19	3.71E-13	4.13E-20	3.93E-13	NA	1.84E-14	8.66E-14	4.65E-17	1.79E-29	7.24E-21	7.72E-23
287	1.28E-28	4.20E-29	3.74E-29	0	5.35E-25	0	0	NA	0	3.68E-25	4.29E-29	4.58E-26	5.48E-29	6.19E-28
288	3.40E-29	8.32E-28	7.41E-28	5.11E-25	2.85E-23	8.68E-25	1.17E-29	NA	1.45E-28	1.30E-28	6.54E-27	1.30E-28	1.28E-27	1.19E-23
289	1.73E-16	2.22E-18	2.03E-16	1.09E-26	1.26E-18	2.12E-19	4.92E-26	NA	5.75E-24	3.66E-24	3.08E-23	2.70E-24	1.44E-20	1.13E-20
290	2.09E-24	2.04E-30	4.05E-24	2.52E-20	3.40E-25	1.20E-25	3.55E-19	NA	2.02E-16	1.59E-16	1.14E-16	1.51E-15	1.10E-15	5.87E-16
291	5.80E-20	1.81E-23	2.34E-22	4.35E-20	1.02E-22	1.43E-23	6.93E-25	NA	2.23E-17	2.18E-21	2.14E-18	1.70E-21	8.93E-16	3.87E-16
292	1.16E-14	5.06E-29	2.27E-13	2.03E-25	1.50E-27	1.11E-27	1.92E-23	NA	NA	1.55E-28	NA	7.81E-20	NA	2.93E-21
293	7.31E-20	3.89E-28	3.81E-19	2.85E-22	5.85E-15	1.81E-15	9.99E-20	NA	3.16E-15	6.17E-16	5.74E-16	2.64E-27	7.11E-14	4.29E-11
294	2.12E-28	6.14E-27	1.24E-27	0	1.33E-27	3.95E-29	4.87E-29	NA	0	2.08E-30	1.00E-28	5.07E-29	2.75E-30	0
295	8.35E-25	8.14E-26	3.10E-26	1.35E-22	6.99E-29	7.47E-21	7.82E-27	NA	4.51E-27	0	5.78E-30	1.88E-26	3.75E-16	7.12E-21
296	7.31E-29	1.64E-26	2.59E-26	8.43E-27	7.41E-27	3.98E-26	4.28E-30	NA	1.58E-29	0	5.42E-23	1.22E-24	3.11E-25	0
297	1.77E-20	2.31E-20	4.24E-19	1.10E-20	7.74E-27	5.46E-18	3.96E-23	NA	2.00E-27	4.20E-19	2.13E-27	1.41E-19	2.52E-16	1.12E-16
298	7.59E-22	4.99E-29	1.41E-17	2.06E-21	1.57E-23	2.42E-22	1.30E-22	NA	2.69E-23	8.91E-24	1.61E-22	1.94E-23	1.19E-20	4.21E-19
299	4.19E-30	4.38E-23	4.34E-30	9.28E-26	7.58E-30	0	3.19E-20	NA	0	NA	1.93E-24	NA	0	NA
300	0	0	0	3.11E-27	1.88E-18	1.85E-21	1.02E-19	NA	NA	NA	NA	NA	NA	NA
301	4.77E-17	7.56E-20	2.67E-17	7.40E-22	2.15E-26	3.99E-15	1.34E-19	NA	NA	NA	NA	NA	NA	NA
302	5.53E-17	3.17E-09	4.14E-18	5.54E-21	6.91E-21	1.12E-19	7.04E-25	NA	NA	NA	NA	NA	NA	NA
303	1.34E-21	2.59E-25	0	8.68E-25	2.22E-30	2.12E-30	1.48E-24	NA	NA	NA	NA	NA	NA	NA
304	1.01E-12	5.84E-16	3.53E-13	4.86E-24	1.11E-13	1.68E-14	1.34E-15	NA	NA	NA	NA	NA	NA	NA
305	1.96E-19	3.48E-29	6.65E-20	3.74E-18	8.18E-21	5.05E-19	1.84E-22	8.09E-21	NA	NA	NA	NA	NA	NA
306	1.35E-16	1.13E-26	5.08E-16	2.94E-19	1.71E-23	8.58E-23	1.10E-18	2.96E-30	NA	NA	NA	NA	NA	NA
307	1.14E-23	0	3.31E-29	0	1.37E-29	1.01E-29	1.61E-27	3.66E-15	NA	NA	NA	NA	NA	NA
308	2.95E-23	2.30E-21	0	2.51E-29	2.06E-29	2.83E-28	4.34E-29	8.68E-29	NA	NA	NA	NA	NA	NA
309	0.000684	1.81E-19	2.63E-24	2.52E-12	6.28E-20	8.87E-17	6.79E-16	8.68E-29	NA	NA	NA	NA	NA	NA
310	2.76E-21	3.86E-11	3.58E-14	1.34E-06	0.000123	2.26E-16	5.36E-18	1.94E-06	NA	NA	NA	NA	NA	NA
311	8.90E-05	3.15E-05	4.38E-14	6.62E-27	5.47E-05	0.000132	1.60E-12	1.20E-15	NA	NA	NA	NA	NA	NA
312	2.69E-20	4.87E-24	1.68E-24	2.21E-24	7.53E-30	8.84E-24	3.17E-28	0	NA	NA	NA	NA	NA	NA
313	3.15E-30	1.18E-28	2.36E-16	0	3.06E-22	4.06E-23	1.97E-22	2.77E-30	NA	NA	NA	NA	NA	NA
314	1.14E-27	9.22E-22	2.44E-23	3.52E-24	6.01E-23	2.22E-30	2.83E-26	7.68E-24	NA	NA	NA	NA	NA	NA
315	3.03E-14	1.62E-24	2.50E-21	8.97E-27	5.70E-22	1.10E-26	2.15E-24	NA	NA	NA	NA	NA	NA	NA
316	9.59E-17	4.92E-16	1.07E-17	5.10E-24	1.26E-19	9.07E-20	6.33E-25	1.36E-12	NA	NA	NA	NA	NA	NA
317	6.11E-21	9.72E-27	8.59E-23	2.00E-26	2.73E-25	3.27E-23	1.14E-19	5.95E-24	NA	NA	NA	NA	NA	NA

318	1.91E-21	2.93E-29	1.34E-19	1.16E-29	5.25E-23	2.98E-22	1.14E-24	3.64E-13	NA	NA	NA	NA	NA	NA
319	6.99E-27	4.54E-22	1.43E-25	1.49E-25	5.36E-25	2.84E-24	4.29E-22	2.00E-26	NA	NA	NA	NA	NA	NA
320	4.41E-20	9.04E-21	6.84E-22	6.67E-25	0	1.41E-24	2.48E-25	3.74E-30	NA	NA	NA	NA	NA	NA
321	0	4.18E-19	1.68E-29	1.36E-17	3.57E-17	1.77E-20	9.21E-26	3.17E-19	NA	NA	NA	NA	NA	NA
322	2.23E-30	0	0	7.66E-25	3.00E-25	1.03E-18	7.05E-29	0	NA	NA	NA	NA	NA	NA
323	2.40E-19	4.23E-30	9.17E-23	3.99E-27	1.13E-23	5.99E-30	3.92E-27	1.65E-27	NA	NA	NA	NA	NA	NA
324	8.74E-30	0	2.11E-30	2.35E-28	3.54E-27	5.73E-28	0	1.10E-29	NA	NA	NA	NA	NA	NA
325	8.80E-09	2.43E-17	6.67E-12	6.41E-16	2.76E-17	1.14E-16	5.88E-12	1.46E-14	NA	NA	NA	NA	NA	NA
326	5.14E-24	2.82E-27	2.44E-24	2.69E-17	2.24E-25	7.30E-22	9.75E-29	0	NA	NA	NA	NA	NA	NA
327	5.46E-30	1.98E-25	5.52E-24	7.09E-26	2.83E-26	1.02E-25	3.14E-20	4.80E-26	NA	NA	NA	NA	NA	NA
328	1.20E-19	1.76E-20	1.41E-23	2.00E-19	6.72E-08	4.91E-09	1.54E-25	7.15E-13	NA	NA	NA	NA	NA	NA
329	1.61E-20	3.53E-11	5.19E-27	1.68E-21	1.05E-19	4.68E-27	3.64E-30	1.98E-20	NA	NA	NA	NA	NA	NA
330	0	5.39E-25	3.10E-29	7.55E-24	2.24E-30	3.81E-30	2.22E-28	1.59E-24	NA	NA	NA	NA	NA	NA
331	3.12E-29	6.85E-20	4.71E-28	7.04E-26	1.34E-16	1.46E-25	1.67E-22	1.59E-24	NA	NA	NA	NA	NA	NA
332	2.05E-30	1.04E-16	0	8.12E-21	5.96E-30	2.99E-21	1.96E-28	0	NA	NA	NA	NA	NA	NA
333	5.51E-12	3.82E-28	2.80E-23	1.96E-18	9.03E-17	7.28E-19	6.17E-17	0	NA	NA	NA	NA	NA	NA
334	2.30E-21	1.91E-18	4.70E-20	5.55E-22	1.50E-19	0	2.14E-19	1.95E-25	NA	NA	NA	NA	NA	NA
335	2.50E-22	2.38E-29	5.17E-22	1.00E-13	5.47E-14	7.56E-14	3.65E-22	4.99E-15	NA	NA	NA	NA	NA	NA
336	8.79E-29	8.04E-14	7.01E-07	2.74E-15	1.61E-15	3.66E-14	7.40E-22	5.46E-20	NA	NA	NA	NA	NA	NA
337	2.39E-23	1.06E-09	1.41E-25	3.18E-25	3.48E-15	1.88E-15	2.45E-16	4.69E-20	NA	NA	NA	NA	NA	NA
338	4.36E-15	1.05E-22	1.91E-15	7.68E-23	1.88E-18	6.58E-15	1.33E-18	5.98E-28	8.18E-16	2.80E-13	NA	NA	1.38E-24	7.50E-30
339	0	3.56E-22	0	2.05E-24	8.02E-28	3.71E-25	8.12E-25	2.63E-29	3.26E-18	7.28E-17	NA	NA	0	0
340	1.66E-25	4.22E-25	9.72E-27	2.44E-26	1.84E-24	6.51E-27	3.60E-27	1.03E-28	NA	NA	NA	NA	NA	NA
341	1.25E-28	1.45E-19	9.73E-29	3.76E-23	5.74E-24	5.47E-25	2.62E-21	5.01E-20	NA	NA	NA	NA	NA	NA
342	4.52E-23	2.69E-27	3.21E-18	9.88E-19	4.55E-27	2.47E-27	6.71E-20	NA	NA	NA	NA	NA	NA	NA
343	0.016756	7.04E-22	0.00017	3.83E-09	9.53E-17	6.41E-07	6.39E-14	NA	NA	NA	NA	NA	NA	NA
344	2.35E-25	2.67E-24	2.57E-25	8.73E-23	1.14E-17	2.09E-17	3.40E-21	NA	NA	NA	NA	NA	NA	NA
345	3.91E-19	2.33E-24	1.23E-19	2.16E-22	7.92E-22	4.25E-21	1.92E-19	1.57E-14	NA	NA	NA	NA	NA	NA
346	5.05E-20	9.75E-28	3.47E-19	2.23E-14	1.46E-19	1.57E-21	4.46E-22	5.94E-22	NA	NA	NA	NA	NA	NA
347	8.44E-20	1.28E-21	6.49E-21	6.85E-28	6.44E-26	1.01E-23	7.81E-25	0	NA	NA	NA	NA	NA	NA
348	3.74E-14	3.62E-21	1.57E-26	7.51E-27	2.81E-28	5.83E-20	4.43E-25	9.60E-24	NA	NA	NA	NA	NA	NA
349	4.53E-30	2.90E-19	9.40E-29	6.47E-28	8.01E-28	3.13E-27	9.20E-29	0	NA	NA	NA	NA	NA	NA
350	8.04E-17	3.71E-22	4.89E-15	5.70E-21	2.67E-16	1.09E-12	1.20E-19	1.53E-17	NA	NA	NA	NA	NA	NA
351	2.49E-29	1.01E-19	2.37E-28	1.36E-27	3.55E-24	3.14E-28	1.11E-28	2.75E-27	NA	NA	NA	NA	NA	NA
352	1.15E-27	8.77E-28	1.60E-27	5.36E-30	4.83E-24	1.08E-23	2.15E-22	0	NA	NA	NA	NA	NA	NA
353	5.89E-25	1.73E-24	3.58E-22	1.28E-25	1.24E-23	5.76E-23	4.12E-30	4.22E-22	NA	NA	NA	NA	NA	NA
354	4.40E-25	1.65E-20	2.62E-19	8.27E-26	4.39E-26	3.60E-21	3.98E-23	8.91E-24	NA	NA	NA	NA	NA	NA
355	5.14E-23	2.31E-30	6.07E-27	2.31E-30	0	3.53E-23	1.13E-29	1.27E-21	NA	NA	NA	NA	NA	NA
356	4.17E-29	8.79E-29	1.74E-19	5.46E-12	9.36E-19	1.37E-17	2.52E-16	7.96E-12	NA	NA	NA	NA	NA	NA
357	4.20E-19	1.68E-18	2.52E-20	5.33E-20	3.41E-20	1.16E-20	3.99E-19	2.79E-27	NA	NA	NA	NA	NA	NA
358	0	6.98E-30	0	0	8.27E-30	0	1.13E-28	2.37E-30	NA	NA	NA	NA	NA	NA
359	9.59E-28	6.92E-27	5.78E-27	3.75E-29	8.04E-28	8.14E-27	1.07E-24	1.42E-28	NA	NA	NA	NA	NA	NA
360	8.77E-25	1.38E-19	2.33E-23	6.20E-21	2.15E-21	1.94E-26	4.90E-22	2.79E-25	NA	NA	NA	NA	NA	NA
361	2.53E-25	2.15E-27	1.62E-27	8.72E-26	3.04E-24	4.25E-30	6.43E-27	1.63E-16	1.13E-28	5.77E-29	NA	NA	4.98E-27	4.54E-28
362	8.84E-21	1.01E-12	9.32E-21	6.54E-23	6.41E-18	1.80E-17	5.71E-19	NA	NA	NA	NA	NA	NA	NA
363	1.13E-29	4.29E-28	1.95E-22	8.26E-30	4.33E-25	4.26E-18	4.73E-15	1.64E-26	NA	NA	NA	NA	NA	NA
364	9.14E-25	2.50E-19	8.81E-30	4.99E-30	2.60E-22	3.11E-29	7.94E-25	1.07E-21	NA	NA	NA	NA	NA	NA
365	6.05E-29	3.17E-25	3.31E-19	9.55E-30	2.00E-27	8.71E-28	3.00E-30	8.41E-25	NA	NA	NA	NA	NA	NA
366	0	0.405617	1.22E-13	5.46E-26	3.82E-13	3.95E-11	8.45E-15	0.000257	5.95E-28	1.10E-29	NA	NA	5.57E-21	8.54E-23

367	6.84E-28	2.28E-26	4.71E-28	4.45E-23	6.32E-27	5.50E-28	1.58E-29	NA	NA	NA	NA	NA	NA	NA
368	2.86E-24	0	4.03E-25	4.58E-23	1.82E-26	5.16E-22	3.86E-18	0	NA	NA	NA	NA	NA	NA
369	6.47E-29	1.37E-21	7.98E-21	2.05E-21	1.93E-20	9.66E-28	9.12E-22	0	NA	NA	NA	NA	NA	NA
370	0.000482	3.27E-12	8.18E-20	3.11E-19	1.25E-21	8.50E-11	4.29E-10	0.012917	NA	NA	NA	NA	NA	NA
371	2.39E-30	3.30E-30	6.73E-25	6.13E-22	2.54E-20	2.13E-27	4.50E-26	4.20E-30	NA	NA	NA	NA	NA	NA
372	6.51E-22	0	0	2.20E-29	1.04E-29	6.34E-21	4.24E-26	0	NA	NA	NA	NA	NA	NA
373	3.07E-20	3.75E-23	1.57E-24	1.02E-21	5.20E-25	8.21E-24	2.46E-25	0	NA	NA	NA	NA	NA	NA
374	8.76E-21	1.43E-22	1.40E-18	3.65E-16	2.37E-23	1.25E-25	2.93E-19	4.16E-28	NA	NA	NA	NA	NA	NA
375	1.74E-28	0	1.78E-27	2.32E-30	2.15E-28	2.43E-29	1.95E-27	NA	NA	NA	NA	NA	NA	NA
376	7.19E-29	0	0	5.16E-26	0	1.00E-28	0	1.69E-29	NA	NA	NA	NA	NA	NA
**p-values of KPSS test on 1st-order differenced data**														
1	0.10	0.10	0.10	0.10	0.10	0.10	0.10	NA	NA	NA	NA	NA	NA	NA
2	0.10	0.10	0.10	0.10	0.10	0.10	0.10	NA	NA	NA	NA	NA	NA	NA
3	0.10	0.10	0.10	0.10	0.10	0.10	0.10	NA	NA	NA	NA	NA	NA	NA
4	0.10	0.10	0.10	0.10	0.10	0.10	0.10	0.10	0.10	0.10	0.07	0.10	0.10	0.10
5	0.10	0.10	0.10	0.10	0.10	0.10	0.10	NA	NA	NA	NA	NA	NA	NA
6	0.10	0.10	0.10	0.10	0.10	0.10	0.10	NA	NA	NA	NA	NA	NA	NA
7	0.07	0.10	0.10	0.10	0.04	0.10	0.10	NA	NA	NA	NA	NA	NA	NA
8	0.10	0.10	0.10	0.10	0.10	0.10	0.10	NA	NA	NA	NA	NA	NA	NA
9	0.10	0.10	0.10	0.10	0.10	0.10	0.10	NA	NA	NA	NA	NA	NA	NA
10	0.10	0.10	0.10	0.10	0.10	0.10	0.10	NA	NA	NA	NA	NA	NA	NA
11	0.10	0.10	0.10	0.10	0.10	0.10	0.10	NA	NA	NA	NA	NA	NA	NA
12	0.10	0.10	0.10	0.09	0.10	0.10	0.10	NA	NA	NA	NA	NA	NA	NA
13	0.10	0.10	0.10	0.10	0.10	0.10	0.10	NA	NA	NA	NA	NA	NA	NA
14	NA	0.10	NA	NA	NA	NA	0.10	NA	NA	NA	NA	NA	NA	NA
15	0.10	0.10	0.10	0.10	0.10	0.10	0.10	NA	NA	NA	NA	NA	NA	NA
16	0.10	0.10	0.10	0.10	0.10	0.10	0.10	NA	NA	NA	NA	NA	NA	NA
17	0.10	0.10	0.10	0.10	0.10	0.10	0.10	0.10	0.10	0.10	0.10	0.10	0.10	0.10
18	0.10	0.10	0.10	0.10	0.10	0.10	0.10	0.10	0.10	0.10	0.10	0.10	0.10	0.10
19	0.10	0.10	0.10	0.10	0.10	0.10	0.10	0.10	0.10	0.10	0.10	0.10	0.10	0.10
20	0.10	0.10	0.10	0.10	0.10	0.10	0.10	NA	NA	0.10	NA	0.10	NA	0.10
21	0.10	0.10	0.10	0.07	0.10	0.10	0.10	0.10	0.10	0.10	0.10	0.10	0.10	0.10
22	0.10	0.04	0.10	0.06	0.10	0.10	0.10	0.04	0.10	0.10	0.10	0.10	0.10	0.10
23	0.10	0.01	0.01	0.01	0.02	0.10	0.10	NA	NA	NA	NA	NA	NA	NA
24	0.10	0.10	0.10	0.10	0.10	0.10	0.10	0.10	0.10	0.10	0.10	0.10	0.10	0.10
25	0.10	0.04	0.10	0.10	0.10	0.10	0.10	0.10	NA	NA	NA	NA	NA	NA
26	0.10	0.10	0.10	0.10	0.10	0.10	0.10	NA	0.10	0.10	0.10	0.10	0.10	0.10
27	0.10	0.10	0.10	0.10	0.10	0.10	0.10	0.10	0.10	0.10	0.10	0.10	0.10	0.10
28	0.10	0.10	0.10	0.10	0.10	0.10	0.10	0.10	0.10	0.10	0.10	0.10	0.10	0.10
29	0.10	0.10	0.10	0.10	0.10	0.10	0.10	NA	NA	NA	NA	NA	NA	NA
30	0.10	0.10	0.10	0.10	0.10	0.10	0.10	0.09	0.10	0.10	0.04	0.10	0.10	0.10
31	0.10	0.10	0.10	0.10	0.10	0.10	0.10	NA	0.10	0.10	0.10	0.10	0.10	0.10
32	0.10	NA	NA	NA	NA	NA	NA	NA	NA	NA	0.10	0.10	0.10	0.10
33	0.10	0.10	0.10	0.10	0.10	0.10	0.10	NA	NA	NA	NA	NA	NA	NA
34	0.10	0.10	0.10	0.10	0.10	0.10	0.10	NA	NA	NA	NA	NA	NA	NA
35	0.10	0.10	0.10	0.10	0.10	0.09	0.07	NA	NA	NA	NA	NA	NA	NA
36	0.05	0.10	0.06	0.10	0.10	0.10	0.10	NA	NA	NA	NA	NA	NA	NA
37	0.10	0.10	0.10	0.10	0.10	0.10	0.10	NA	NA	NA	NA	NA	NA	NA
38	0.10	0.10	0.10	0.10	0.10	0.10	0.10	NA	NA	NA	NA	NA	NA	NA

39	0.10	0.10	0.10	0.10	0.10	0.10	0.10	NA	NA	NA	NA	NA	NA	NA
40	0.10	0.01	0.02	0.10	0.10	0.10	0.10	NA	NA	NA	NA	NA	NA	NA
41	0.10	0.10	0.10	0.10	0.10	0.10	0.10	NA	NA	NA	NA	NA	NA	NA
42	0.10	0.10	0.10	0.10	0.10	0.10	0.04	NA	NA	NA	NA	NA	NA	NA
43	0.10	0.10	0.10	0.10	0.10	0.10	0.10	NA	NA	NA	NA	NA	NA	NA
44	0.10	0.10	0.10	0.10	0.10	0.10	0.10	NA	NA	NA	NA	NA	NA	NA
45	0.10	0.10	0.10	0.10	0.10	0.10	0.10	NA	NA	NA	NA	NA	NA	NA
46	0.10	0.10	0.10	0.10	0.10	0.10	0.10	NA	NA	NA	NA	NA	NA	NA
47	0.10	0.10	0.10	0.10	0.10	0.10	0.10	NA	NA	NA	NA	NA	NA	NA
48	0.10	0.10	0.10	0.10	0.10	0.10	0.10	NA	NA	NA	NA	NA	NA	NA
49	0.10	0.10	0.10	0.10	0.10	0.10	0.10	NA	NA	NA	NA	NA	NA	NA
50	0.10	0.10	0.10	0.10	0.10	0.10	0.10	NA	NA	NA	NA	NA	NA	NA
51	0.10	0.10	0.04	0.09	0.10	0.10	0.08	NA	NA	NA	NA	NA	NA	NA
52	0.10	0.10	0.10	0.10	0.10	0.10	0.10	NA	NA	NA	NA	NA	NA	NA
53	0.10	0.10	0.10	0.10	0.10	0.10	0.10	NA	NA	NA	NA	NA	NA	NA
54	0.10	0.10	0.10	0.10	0.10	0.10	0.10	NA	NA	NA	NA	NA	NA	NA
55	0.10	0.10	0.10	0.10	0.10	0.10	0.10	NA	NA	NA	NA	NA	NA	NA
56	0.10	0.10	0.10	0.10	0.10	0.10	0.07	NA	NA	NA	NA	NA	NA	NA
57	0.10	0.10	0.10	0.10	0.10	0.10	0.10	NA	NA	NA	NA	NA	NA	NA
58	0.10	0.10	0.10	0.04	0.07	0.10	0.10	NA	NA	NA	NA	NA	NA	NA
59	0.10	0.10	0.10	0.10	0.10	0.10	0.10	NA	NA	NA	NA	NA	NA	NA
60	0.10	0.10	0.10	0.10	0.10	0.10	0.10	NA	NA	NA	NA	NA	NA	NA
61	0.10	0.10	0.10	0.04	0.10	0.10	0.10	NA	NA	NA	NA	NA	NA	NA
62	0.10	0.10	0.10	0.04	0.10	0.10	0.10	NA	NA	NA	NA	NA	NA	NA
63	0.10	0.04	0.02	0.10	0.10	0.10	0.10	NA	NA	NA	NA	NA	NA	NA
64	0.10	0.10	0.10	0.10	0.10	0.10	0.10	NA	NA	NA	NA	NA	NA	NA
65	0.10	0.01	0.01	0.07	0.10	0.02	0.10	NA	NA	NA	NA	NA	NA	NA
66	0.10	0.10	0.10	0.10	0.10	0.10	0.10	NA	NA	NA	NA	NA	NA	NA
67	0.10	0.10	0.10	0.10	0.10	0.10	0.10	NA	NA	NA	NA	NA	NA	NA
68	0.10	0.01	0.03	0.10	0.04	0.10	0.10	NA	NA	NA	NA	NA	NA	NA
69	0.10	0.10	0.10	0.10	0.10	0.10	0.05	NA	NA	NA	NA	NA	NA	NA
70	0.10	0.10	0.10	0.10	0.04	0.10	0.04	NA	NA	NA	NA	NA	NA	NA
71	0.10	0.06	0.10	0.10	0.10	0.10	0.10	NA	NA	NA	NA	NA	NA	NA
72	0.10	0.10	0.10	0.10	0.10	0.10	0.10	NA	NA	NA	NA	NA	NA	NA
73	0.10	0.10	0.10	0.10	0.10	0.10	0.10	NA	NA	NA	NA	NA	NA	NA
74	0.10	0.01	0.01	0.10	0.10	0.10	0.10	NA	NA	NA	NA	NA	NA	NA
75	0.10	0.10	0.10	0.10	0.10	0.10	0.10	NA	NA	NA	NA	NA	NA	NA
76	0.10	0.10	0.10	0.10	0.10	0.10	0.10	NA	NA	NA	NA	NA	NA	NA
77	0.10	0.10	0.10	0.10	0.07	0.10	0.10	NA	NA	NA	NA	NA	NA	NA
78	0.10	0.10	0.10	0.10	0.10	0.10	0.10	NA	NA	NA	NA	NA	NA	NA
79	0.10	0.10	0.10	0.10	0.10	0.10	0.10	NA	NA	NA	NA	NA	NA	NA
80	0.10	0.01	0.10	0.10	0.10	0.10	0.10	NA	NA	NA	NA	NA	NA	NA
81	0.10	0.10	0.10	0.10	0.10	0.10	0.10	NA	NA	NA	NA	NA	NA	NA
82	0.10	0.10	0.10	0.10	0.04	0.10	0.10	NA	NA	NA	NA	NA	NA	NA
83	0.10	0.10	0.10	0.10	0.10	0.10	0.10	NA	NA	NA	NA	NA	NA	NA
84	0.10	0.10	0.10	0.10	0.10	0.10	0.10	NA	NA	NA	NA	NA	NA	NA
85	0.10	0.10	0.10	0.10	0.10	0.10	0.10	0.10	NA	NA	NA	NA	NA	NA
86	0.10	0.10	0.10	0.10	0.10	0.10	0.10	NA	NA	NA	NA	NA	NA	NA
87	0.10	0.01	0.06	0.10	0.10	0.10	0.10	NA	NA	NA	NA	NA	NA	NA

88	0.10	0.10	0.10	0.10	0.10	0.10	0.10	NA	NA	NA	NA	NA	NA	NA
89	0.10	0.10	0.10	0.10	0.10	0.10	0.04	NA	NA	NA	NA	NA	NA	NA
90	0.10	0.10	0.10	0.10	0.10	0.10	0.10	NA	NA	NA	NA	NA	NA	NA
91	0.10	0.10	0.10	0.10	0.10	0.10	0.10	NA	NA	NA	NA	NA	NA	NA
92	0.10	0.10	0.10	0.10	0.10	0.10	0.10	NA	NA	NA	NA	NA	NA	NA
93	0.10	0.10	0.10	0.10	0.10	0.10	0.10	NA	NA	NA	NA	NA	NA	NA
94	0.10	0.10	0.10	0.10	0.10	0.10	0.10	NA	NA	NA	NA	NA	NA	NA
95	0.10	0.10	0.10	0.10	0.10	0.10	0.10	NA	NA	NA	NA	NA	NA	NA
96	0.10	0.10	0.10	0.10	0.10	0.10	0.10	NA	NA	NA	NA	NA	NA	NA
97	0.10	0.10	0.10	0.10	0.10	0.10	0.10	NA	NA	NA	NA	NA	NA	NA
98	0.10	0.10	0.10	0.10	0.04	0.10	0.10	NA	NA	NA	NA	NA	NA	NA
99	0.10	0.10	0.10	0.09	0.10	0.08	0.10	0.10	NA	NA	NA	NA	NA	NA
100	0.10	0.10	0.10	0.10	0.10	0.10	0.10	0.10	NA	NA	NA	NA	NA	NA
101	0.10	0.01	0.01	0.10	0.10	0.01	0.10	0.10	NA	NA	NA	NA	NA	NA
102	0.10	0.03	0.02	0.10	0.10	0.10	0.10	0.10	NA	NA	NA	NA	NA	NA
103	0.10	0.10	0.10	0.10	0.10	0.10	0.10	0.10	NA	NA	NA	NA	NA	NA
104	0.10	0.10	0.10	0.10	0.10	0.10	0.10	NA	NA	NA	NA	NA	NA	NA
105	0.10	0.10	0.10	0.10	0.10	0.10	0.10	0.10	NA	NA	NA	NA	NA	NA
106	0.10	0.01	0.09	0.01	0.01	0.01	0.10	0.10	NA	NA	NA	NA	NA	NA
107	0.10	0.10	0.10	0.09	0.10	0.10	0.10	0.10	NA	NA	NA	NA	NA	NA
108	0.10	0.10	0.10	0.10	0.10	0.10	0.10	0.10	NA	NA	NA	NA	NA	NA
109	0.10	0.10	0.10	0.10	0.10	0.10	0.10	NA	NA	NA	NA	NA	NA	NA
110	0.10	0.10	0.10	0.10	0.10	0.10	0.10	0.10	NA	NA	NA	NA	NA	NA
111	0.10	0.10	0.10	0.10	0.10	0.10	0.10	0.10	0.10	0.07	0.10	0.10	0.10	0.10
112	0.10	0.10	0.10	0.10	0.10	0.10	0.09	0.10	NA	0.10	NA	0.10	NA	0.10
113	0.10	0.10	0.10	0.10	0.10	0.10	0.10	NA	0.10	0.10	0.10	0.10	0.10	0.10
114	0.10	0.10	0.10	0.10	0.10	0.10	0.10	0.10	NA	NA	NA	NA	NA	NA
115	0.10	0.02	0.10	0.10	0.10	0.10	0.10	NA	NA	NA	NA	NA	NA	NA
116	0.10	0.08	0.10	0.10	0.10	0.10	0.10	0.10	NA	NA	NA	NA	NA	NA
117	0.10	0.01	0.10	0.10	0.10	0.10	0.10	0.10	NA	NA	NA	NA	NA	NA
118	0.04	0.04	0.10	0.10	0.10	0.10	0.04	0.07	NA	NA	NA	NA	NA	NA
119	0.10	0.10	0.10	0.10	0.10	0.10	0.09	0.10	NA	NA	NA	NA	NA	NA
120	0.10	0.10	0.08	0.07	0.06	0.10	0.10	0.10	0.10	0.10	0.10	0.05	0.10	0.10
121	0.10	0.10	0.10	0.10	0.10	0.10	0.10	0.10	0.10	0.10	0.10	0.09	0.10	0.10
122	0.10	0.10	0.10	0.10	0.10	0.10	0.10	0.10	0.10	0.10	0.07	0.10	0.10	0.10
123	0.10	0.10	0.10	0.10	0.10	0.10	0.10	0.10	0.10	0.10	0.10	0.10	0.10	0.10
124	0.10	0.10	0.10	0.10	0.04	0.10	0.10	NA	NA	NA	NA	NA	NA	NA
125	0.10	0.06	0.10	0.10	0.10	0.10	0.10	NA	0.10	0.10	0.10	0.10	0.10	0.10
126	0.10	0.10	0.10	0.10	0.10	0.10	0.08	NA	NA	0.05	NA	0.10	NA	0.10
127	0.10	0.10	0.10	0.10	0.10	0.10	0.10	NA	NA	NA	NA	NA	NA	NA
128	0.10	0.10	0.10	0.10	0.10	0.10	0.10	NA	NA	NA	NA	NA	NA	NA
129	0.10	0.10	0.10	0.10	0.10	0.10	0.10	NA	0.10	0.10	0.10	0.10	0.10	0.10
130	0.10	0.10	0.10	0.10	0.10	0.10	0.10	NA	0.10	0.10	0.10	0.10	0.10	0.10
131	0.10	0.10	0.10	0.10	0.10	0.10	0.10	NA	0.10	0.10	0.10	0.10	0.10	0.10
132	0.10	0.10	0.10	0.10	0.10	0.10	0.10	NA	NA	NA	NA	NA	NA	NA
133	0.10	0.10	0.10	0.10	0.10	0.10	0.10	NA	NA	NA	NA	NA	NA	NA
134	0.10	0.10	0.10	0.10	0.10	0.10	0.10	NA	NA	NA	NA	NA	NA	NA
135	0.10	0.10	0.10	0.10	0.10	0.10	0.10	NA	NA	NA	NA	NA	NA	NA
136	0.10	0.10	0.10	0.07	0.10	0.10	0.10	NA	NA	NA	NA	NA	NA	NA

137	0.10	0.10	0.10	0.10	0.10	0.10	0.10	NA	NA	NA	NA	NA	NA	NA
138	0.07	0.10	0.10	0.10	0.10	0.10	0.07	NA	NA	NA	NA	NA	NA	NA
139	0.06	0.08	0.10	0.04	0.10	0.10	0.10	NA	NA	NA	NA	NA	NA	NA
140	0.10	0.10	0.10	0.10	0.10	0.10	0.10	NA	NA	NA	NA	NA	NA	NA
141	0.10	0.10	0.10	0.10	0.10	0.10	0.10	NA	NA	NA	NA	NA	NA	NA
142	0.10	0.10	0.10	0.10	0.10	0.10	0.10	NA	NA	NA	NA	NA	NA	NA
143	0.10	0.10	0.10	0.10	0.10	0.10	0.10	NA	NA	NA	NA	NA	NA	NA
144	0.10	0.10	0.10	0.10	0.10	0.10	0.10	NA	NA	NA	NA	NA	NA	NA
145	0.10	0.10	0.10	0.10	0.10	0.10	0.10	NA	NA	NA	NA	NA	NA	NA
146	0.10	0.10	0.10	0.10	0.10	0.10	0.10	NA	NA	NA	NA	NA	NA	NA
147	0.10	0.10	0.10	0.10	0.10	0.10	0.10	NA	NA	NA	NA	NA	NA	NA
148	0.10	0.02	0.05	0.10	0.10	0.10	0.04	NA	NA	NA	NA	NA	NA	NA
149	0.10	0.10	0.10	0.10	0.10	0.10	0.09	NA	NA	NA	NA	NA	NA	NA
150	0.10	0.10	0.10	0.10	0.10	0.10	0.10	NA	NA	NA	NA	NA	NA	NA
151	0.10	0.10	0.10	0.09	0.10	0.10	0.10	NA	NA	NA	NA	NA	NA	NA
152	0.10	0.10	0.10	0.10	0.10	0.10	0.10	NA	NA	NA	NA	NA	NA	NA
153	0.10	0.10	0.10	0.10	0.10	0.10	0.09	NA	0.10	0.10	0.10	0.10	0.09	0.10
154	0.10	0.10	0.10	0.10	0.10	0.10	0.10	NA	NA	NA	NA	NA	NA	NA
155	0.10	0.10	0.10	0.10	0.10	0.07	0.10	NA	NA	NA	NA	NA	NA	NA
156	0.10	0.10	0.10	0.10	0.10	0.10	0.10	NA	NA	NA	NA	NA	NA	NA
157	0.10	0.10	0.10	0.10	0.10	0.10	0.10	NA	NA	NA	NA	NA	NA	NA
158	0.04	0.08	0.10	0.10	0.10	0.10	0.10	NA	0.10	0.04	0.10	0.10	0.10	0.10
159	0.10	0.10	0.10	0.10	0.04	0.10	0.10	NA	0.10	0.10	0.10	0.10	0.10	0.10
160	0.10	0.10	0.10	0.10	0.10	0.10	0.10	NA	NA	0.10	NA	0.10	NA	0.10
161	0.09	0.10	0.10	0.10	0.10	0.10	0.10	NA	NA	NA	NA	NA	NA	NA
162	0.10	0.10	0.10	0.10	0.10	0.10	0.10	NA	NA	NA	NA	NA	NA	NA
163	0.10	0.10	0.10	0.10	0.10	0.10	0.10	NA	NA	NA	NA	NA	NA	NA
164	0.10	0.10	0.10	0.10	0.10	0.04	0.10	NA	NA	NA	NA	NA	NA	NA
165	0.10	0.10	0.10	0.10	0.10	0.10	0.10	NA	NA	NA	NA	NA	NA	NA
166	0.10	0.10	0.10	0.10	0.10	0.10	0.10	NA	NA	NA	NA	NA	NA	NA
167	0.10	0.10	0.10	0.10	0.10	0.04	0.10	NA	0.10	0.10	0.10	0.04	0.10	0.10
168	0.10	0.10	0.10	0.10	0.10	0.10	0.10	NA	0.10	NA	0.10	NA	0.03	NA
169	0.10	0.10	0.10	0.10	0.10	0.10	0.06	NA	0.10	0.10	0.10	0.10	0.10	0.10
170	0.10	0.10	0.10	0.10	0.10	0.10	0.10	NA	NA	NA	NA	NA	NA	NA
171	0.10	0.10	0.10	0.04	0.10	0.10	0.10	NA	0.10	0.10	0.10	0.10	0.10	0.10
172	0.10	0.10	0.10	0.10	0.10	0.10	0.10	NA	0.10	0.10	0.10	0.10	0.10	0.10
173	0.10	0.10	0.10	0.04	0.10	0.10	0.10	NA	NA	NA	NA	NA	NA	NA
174	0.10	0.10	0.10	0.10	0.10	0.08	0.10	NA	0.10	0.10	0.10	0.10	0.01	0.10
175	0.10	0.10	0.10	0.10	0.10	0.10	0.10	NA	NA	NA	NA	NA	NA	NA
176	0.10	0.10	0.10	0.10	0.10	0.10	0.10	NA	NA	NA	NA	NA	NA	NA
177	0.10	0.10	0.10	0.10	0.10	0.10	0.10	NA	NA	NA	NA	NA	NA	NA
178	0.04	0.10	0.10	0.10	0.10	0.10	0.03	NA	NA	NA	NA	NA	NA	NA
179	0.10	0.10	0.10	0.08	0.10	0.10	0.09	NA	NA	NA	NA	NA	NA	NA
180	0.10	0.10	0.10	0.10	0.10	0.10	0.10	NA	NA	NA	NA	NA	NA	NA
181	0.10	0.10	0.10	0.06	0.10	0.08	0.10	NA	NA	NA	NA	NA	NA	NA
182	0.10	0.10	0.10	0.10	0.10	0.10	0.09	NA	NA	NA	NA	NA	NA	NA
183	0.10	0.10	0.10	0.10	0.10	0.10	0.04	NA	NA	NA	NA	NA	NA	NA
184	0.10	0.10	0.10	0.10	0.10	0.10	0.10	NA	NA	NA	NA	NA	NA	NA
185	0.10	0.10	0.10	0.10	0.10	0.10	0.10	NA	NA	NA	NA	NA	NA	NA

186	0.10	0.10	0.10	0.10	0.10	0.10	0.10	NA	NA	NA	NA	NA	NA	NA
187	0.10	0.10	0.10	0.10	0.10	0.10	0.10	NA	NA	0.10	NA	0.10	NA	0.10
188	0.10	0.10	0.10	0.10	0.10	0.10	0.10	NA	0.10	NA	0.10	NA	0.10	NA
189	0.10	0.10	0.10	0.10	0.10	0.10	0.10	NA	0.10	0.10	0.10	0.10	0.10	0.02
190	0.10	0.10	0.10	0.10	0.10	0.04	0.10	NA	NA	NA	NA	NA	NA	NA
191	0.10	0.10	0.10	0.10	0.10	0.10	0.10	NA	0.08	0.10	0.10	0.10	0.10	0.10
192	0.10	0.10	0.10	0.10	0.10	0.10	0.10	NA	0.10	0.10	0.10	0.10	0.06	0.10
193	0.10	0.10	0.10	0.10	0.10	0.10	0.10	NA	0.10	0.10	0.10	0.10	0.10	0.10
194	0.01	0.10	0.02	0.10	0.10	0.10	0.10	NA	NA	NA	NA	NA	NA	NA
195	0.01	0.10	0.03	0.10	0.10	0.10	0.10	NA	0.04	0.10	0.10	0.04	0.10	0.10
196	0.10	0.10	0.10	0.10	0.10	0.05	0.08	NA	NA	NA	NA	NA	NA	NA
197	0.10	0.10	0.10	0.10	0.10	0.10	0.10	NA	NA	0.10	NA	0.10	0.07	0.01
198	0.10	0.10	0.10	0.04	0.10	0.10	0.10	NA	0.10	0.10	0.10	0.10	0.10	0.10
199	0.10	0.10	0.10	0.10	0.10	0.10	0.10	NA	0.10	0.10	0.10	0.10	0.10	0.10
200	0.10	0.10	0.10	0.10	0.10	0.10	0.10	NA	0.04	0.10	0.04	0.10	0.10	0.10
201	0.10	0.10	0.10	0.10	0.10	0.10	0.10	NA	0.10	0.10	0.10	0.04	0.10	0.10
202	0.10	0.10	0.10	0.10	0.10	0.10	0.10	NA	0.10	0.10	0.10	0.10	0.10	0.10
203	0.10	0.10	0.10	0.10	0.10	0.10	0.10	NA	0.10	NA	0.10	NA	0.10	NA
204	0.10	0.10	0.10	0.08	0.10	0.09	0.10	NA	0.10	0.10	0.10	0.10	0.10	0.10
205	0.10	0.10	0.10	0.10	0.10	0.10	0.10	NA	0.10	0.10	0.10	0.10	0.10	0.10
206	0.10	0.10	0.10	0.10	0.10	0.05	0.10	NA	0.10	0.08	0.10	0.10	0.10	0.10
207	0.10	0.10	0.10	0.10	0.10	0.10	0.10	NA	0.10	0.10	0.10	0.10	0.10	0.10
208	0.08	0.10	0.10	0.10	0.10	0.10	0.06	NA	0.10	0.04	0.10	0.10	0.10	0.10
209	0.10	0.10	0.10	0.10	0.10	0.10	0.10	NA	0.10	0.10	0.10	0.10	0.10	0.10
210	0.10	0.10	0.10	0.10	0.10	0.10	0.10	NA	0.10	0.10	0.10	0.10	0.10	0.10
211	0.10	0.10	0.10	0.10	0.10	0.10	0.10	NA	0.10	0.10	0.10	0.10	0.10	0.10
212	0.10	0.10	0.10	0.10	0.10	0.10	0.10	NA	0.10	0.10	0.10	0.10	0.10	0.10
213	0.10	0.10	0.10	0.10	0.10	0.10	0.10	NA	0.10	0.10	0.10	0.10	0.10	0.10
214	0.10	0.10	0.10	0.10	0.10	0.10	0.10	NA	0.10	0.10	0.10	0.10	0.10	0.10
215	0.10	0.10	0.10	0.10	0.10	0.10	0.10	NA	0.10	0.10	0.10	0.10	0.10	0.10
216	0.10	0.10	0.10	0.10	0.10	0.10	0.10	NA	0.10	0.10	0.10	0.10	0.10	0.10
217	0.10	0.10	0.10	0.10	0.10	0.10	0.10	NA	0.10	0.10	0.10	0.10	0.10	0.10
218	0.10	0.10	0.10	0.10	0.10	0.10	0.10	NA	0.10	0.10	0.10	0.10	0.02	0.10
219	0.10	0.10	0.10	0.10	0.10	0.10	0.10	NA	0.10	0.10	0.10	0.10	0.10	0.10
220	0.06	0.10	0.10	0.10	0.07	0.10	0.09	NA	NA	NA	NA	NA	NA	NA
221	0.10	0.10	0.10	0.10	0.10	0.10	0.10	NA	0.10	0.10	0.10	0.10	0.10	0.10
222	0.10	0.10	0.10	0.10	0.10	0.10	0.10	NA	0.10	0.10	0.10	0.10	0.10	0.10
223	0.10	0.10	0.10	0.10	0.10	0.10	0.10	NA	0.10	0.10	0.10	0.10	0.10	0.10
224	0.10	0.10	0.10	0.10	0.10	0.10	0.10	NA	0.10	0.10	0.10	0.10	0.10	0.09
225	0.10	0.10	0.10	0.10	0.10	0.10	0.10	NA	0.10	0.10	0.10	0.10	0.10	0.10
226	0.10	0.10	0.10	0.10	0.04	0.10	0.10	NA	0.10	0.10	0.10	0.10	0.10	0.10
227	0.10	0.10	0.10	0.10	0.04	0.10	0.10	NA	0.10	0.10	0.10	0.10	0.10	0.10
228	0.10	0.10	0.10	0.10	0.10	0.10	0.10	NA	0.04	0.10	0.10	0.04	0.10	0.09
229	0.10	0.01	0.09	0.10	0.10	0.10	0.10	NA	0.10	0.10	0.10	0.10	0.10	0.10
230	0.10	0.10	0.10	0.10	0.10	0.10	0.10	NA	0.10	0.10	0.10	0.10	0.10	0.10
231	0.10	0.10	0.10	0.10	0.10	0.07	0.10	NA	0.10	0.10	0.10	0.10	0.05	0.10
232	0.10	0.10	0.10	0.10	0.10	0.10	0.04	NA	0.10	0.10	0.10	0.10	0.10	0.10
233	0.10	0.10	0.10	0.10	0.10	0.10	0.10	NA	0.10	NA	0.10	NA	0.10	NA
234	0.10	0.10	0.04	0.10	0.09	0.10	0.10	0.10	0.10	0.10	0.04	0.10	0.05	0.10

235	0.10	0.04	0.10	0.10	0.10	0.10	0.10	0.10	NA	NA	NA	NA	NA	NA
236	0.10	0.10	0.10	0.10	0.10	0.10	0.10	0.10	0.10	0.04	0.10	0.10	0.10	0.10
237	0.10	0.10	0.10	0.10	0.10	0.10	0.10	NA	0.10	NA	0.10	NA	0.10	NA
238	0.10	0.10	0.10	0.10	0.10	0.10	0.07	NA	0.10	0.10	0.10	0.10	0.10	0.10
239	0.10	0.10	0.10	0.10	0.10	0.10	0.10	0.10	0.10	0.10	0.10	0.05	0.10	0.10
240	0.10	0.10	0.10	0.10	0.10	0.10	0.10	NA	0.10	0.10	0.10	0.10	0.10	0.10
241	0.09	0.10	0.10	0.10	0.10	0.10	0.07	0.10	0.10	0.10	0.10	0.10	0.10	0.10
242	0.10	0.10	0.10	0.10	0.10	0.10	0.10	NA	0.10	0.10	0.10	0.10	0.10	0.10
243	0.10	0.10	0.10	0.10	0.10	0.10	0.10	NA	0.10	0.08	0.10	0.10	0.10	0.10
244	0.10	0.10	0.05	0.10	0.09	0.10	0.10	NA	0.10	0.10	0.05	0.10	0.10	0.09
245	0.10	0.10	0.10	0.10	0.10	0.05	0.10	0.10	0.10	0.03	0.10	0.10	0.10	0.10
246	0.10	0.10	0.10	0.10	0.10	0.10	0.10	NA	0.10	0.10	0.10	0.10	0.09	0.10
247	0.10	0.10	0.10	0.10	0.09	0.10	0.10	0.10	0.10	NA	0.06	NA	0.10	NA
248	0.10	0.10	0.10	0.10	0.10	0.10	0.07	NA	0.10	0.10	0.07	0.10	0.10	0.10
249	0.10	0.10	0.10	0.10	0.10	0.10	0.10	0.10	0.10	0.10	0.10	0.08	0.10	0.05
250	0.10	0.10	0.10	0.10	0.04	0.10	0.04	0.10	0.10	0.10	0.05	0.10	0.10	0.10
251	0.10	0.06	0.10	0.10	0.10	0.10	0.10	NA	NA	0.10	NA	0.10	NA	0.10
252	0.10	0.10	0.10	0.10	0.10	0.10	0.10	0.10	0.10	0.07	0.10	0.10	0.10	0.06
253	0.10	0.10	0.10	0.10	0.10	0.10	0.10	0.10	0.10	0.10	0.10	0.10	0.10	0.10
254	0.10	0.10	0.10	0.10	0.10	0.10	0.10	NA	0.05	0.10	0.10	0.10	0.10	0.10
255	0.10	0.06	0.10	0.10	0.10	0.10	0.10	NA	0.10	NA	0.10	NA	0.10	NA
256	0.10	0.10	0.10	0.05	0.10	0.10	0.10	NA	0.10	0.10	0.10	0.10	0.10	0.10
257	0.10	0.10	0.10	0.10	0.10	0.10	0.10	NA	0.10	0.10	0.10	0.10	0.10	0.10
258	0.10	0.10	0.10	0.10	0.10	0.10	0.10	NA	0.10	0.10	0.10	0.10	0.10	0.10
259	0.10	0.10	0.10	0.10	0.10	0.10	0.10	NA	0.10	0.10	0.10	0.10	0.10	0.10
260	0.10	0.10	0.10	0.10	0.10	0.10	0.10	0.10	0.10	0.10	0.10	0.10	0.10	0.10
261	0.10	0.10	0.10	0.10	0.10	0.10	0.10	NA	0.10	0.10	0.10	0.10	0.10	0.10
262	0.10	0.10	0.10	0.10	0.10	0.10	0.10	NA	0.04	0.10	0.10	0.10	NA	0.10
263	0.10	0.10	0.10	0.10	0.10	0.10	0.10	0.10	0.10	0.10	0.10	0.10	0.10	0.10
264	0.10	0.10	0.10	0.10	0.10	0.10	0.10	0.10	0.10	0.10	0.10	0.10	0.10	0.10
265	0.10	0.10	0.10	0.10	0.10	0.10	0.10	0.10	0.10	0.10	0.10	0.10	0.10	0.10
266	0.10	0.10	0.04	0.10	0.10	0.10	0.10	NA	0.10	NA	0.10	NA	0.10	NA
267	0.10	0.10	0.10	0.10	0.10	0.10	0.10	NA	0.10	0.10	0.10	0.10	0.10	0.10
268	0.10	0.10	0.06	0.07	0.10	0.10	0.04	NA	NA	NA	0.10	0.10	0.07	0.10
269	0.10	0.10	0.10	0.10	0.10	0.10	0.10	NA	0.10	0.10	0.10	0.10	0.10	0.10
270	0.10	0.10	0.10	0.06	0.10	0.10	0.10	NA	0.10	0.10	0.07	0.10	0.10	0.10
271	0.10	0.10	0.10	0.10	0.10	0.10	0.10	NA	0.10	0.10	0.10	0.10	0.10	0.10
272	0.10	0.10	0.10	0.10	0.10	0.10	0.10	NA	0.10	0.10	0.10	0.10	0.10	0.10
273	0.10	0.10	0.10	0.10	0.10	0.10	0.10	NA	0.07	0.10	0.10	0.10	0.10	0.10
274	0.10	0.10	0.10	0.10	0.04	0.10	0.10	NA	0.10	0.10	0.10	0.10	0.10	0.10
275	0.10	0.10	0.10	0.10	0.10	0.10	0.10	0.10	0.10	0.10	0.10	0.09	0.10	0.10
276	0.10	0.10	0.10	0.10	0.10	0.10	0.10	NA	0.10	0.10	0.10	0.10	0.10	0.10
277	0.10	0.10	0.10	0.10	0.10	0.10	0.10	NA	0.10	0.10	0.10	0.10	0.10	0.10
278	0.10	0.10	0.10	0.10	0.10	0.10	0.10	NA	0.10	0.10	0.10	0.10	0.10	0.10
279	0.10	0.10	0.10	0.10	0.10	0.10	0.10	NA	0.10	0.10	0.10	0.10	0.10	0.10
280	0.10	0.10	0.10	0.10	0.10	0.10	0.07	NA	0.10	0.10	0.10	0.10	0.10	0.10
281	0.10	0.10	0.10	0.10	0.04	0.05	0.10	NA	0.10	0.10	0.10	0.10	0.10	0.10
282	0.10	0.10	0.10	0.10	0.10	0.10	0.10	NA	0.10	0.10	0.10	0.10	0.10	0.10
283	0.10	0.10	0.10	0.10	0.10	0.10	0.10	NA	NA	NA	NA	NA	NA	NA

284	0.10	0.10	0.10	0.10	0.10	0.10	0.10	NA	NA	NA	NA	NA	NA	NA
285	0.10	0.09	0.10	0.10	0.10	0.10	0.10	NA	0.10	0.10	0.10	0.04	0.10	0.10
286	0.10	0.10	0.10	0.10	0.10	0.10	0.10	NA	0.10	0.10	0.10	0.10	0.10	0.10
287	0.10	0.10	0.10	0.10	0.10	0.10	0.10	NA	0.10	0.10	0.10	0.10	0.10	0.10
288	0.10	0.10	0.10	0.10	0.10	0.10	0.10	NA	0.10	0.08	0.10	0.10	0.10	0.10
289	0.10	0.10	0.10	0.10	0.10	0.10	0.10	NA	0.10	0.10	0.10	0.10	0.10	0.10
290	0.10	0.10	0.10	0.10	0.10	0.10	0.10	NA	0.10	0.10	0.10	0.10	0.10	0.10
291	0.10	0.10	0.10	0.10	0.10	0.10	0.10	NA	0.10	0.10	0.10	0.08	0.10	0.10
292	0.10	0.10	0.10	0.10	0.10	0.10	0.10	NA	NA	0.10	NA	0.10	NA	0.10
293	0.10	0.10	0.10	0.10	0.10	0.10	0.10	NA	0.10	0.04	0.10	0.10	0.10	0.10
294	0.10	0.10	0.10	0.10	0.10	0.10	0.10	NA	0.10	0.10	0.10	0.10	0.10	0.10
295	0.10	0.10	0.10	0.10	0.10	0.10	0.10	NA	0.10	0.10	0.10	0.10	0.10	0.10
296	0.10	0.10	0.10	0.10	0.10	0.10	0.10	NA	0.10	0.10	0.10	0.10	0.10	0.10
297	0.10	0.10	0.10	0.10	0.10	0.10	0.10	NA	0.10	0.10	0.10	0.10	0.10	0.10
298	0.10	0.10	0.10	0.10	0.10	0.10	0.10	NA	0.10	0.10	0.10	0.10	0.10	0.10
299	0.10	0.10	0.10	0.10	0.10	0.10	0.10	NA	0.10	NA	0.10	NA	0.10	NA
300	0.10	0.10	0.10	0.10	0.10	0.10	0.10	NA	NA	NA	NA	NA	NA	NA
301	0.10	0.10	0.10	0.10	0.10	0.10	0.10	NA	NA	NA	NA	NA	NA	NA
302	0.10	0.10	0.10	0.10	0.10	0.10	0.10	NA	NA	NA	NA	NA	NA	NA
303	0.10	0.10	0.10	0.10	0.10	0.10	0.10	NA	NA	NA	NA	NA	NA	NA
304	0.10	0.10	0.10	0.10	0.10	0.10	0.10	NA	NA	NA	NA	NA	NA	NA
305	0.10	0.10	0.10	0.04	0.10	0.10	0.10	0.07	NA	NA	NA	NA	NA	NA
306	0.10	0.10	0.10	0.10	0.10	0.10	0.10	0.10	NA	NA	NA	NA	NA	NA
307	0.10	0.10	0.10	0.10	0.10	0.10	0.10	0.10	NA	NA	NA	NA	NA	NA
308	0.10	0.10	0.10	0.10	0.10	0.10	0.10	0.10	NA	NA	NA	NA	NA	NA
309	0.10	0.10	0.10	0.10	0.10	0.10	0.10	0.10	NA	NA	NA	NA	NA	NA
310	0.10	0.09	0.10	0.10	0.10	0.10	0.04	0.10	NA	NA	NA	NA	NA	NA
311	0.10	0.10	0.10	0.10	0.10	0.10	0.10	0.10	NA	NA	NA	NA	NA	NA
312	0.10	0.10	0.10	0.09	0.10	0.10	0.10	0.10	NA	NA	NA	NA	NA	NA
313	0.10	0.10	0.10	0.10	0.10	0.10	0.10	0.10	NA	NA	NA	NA	NA	NA
314	0.10	0.10	0.10	0.10	0.10	0.10	0.10	0.10	NA	NA	NA	NA	NA	NA
315	0.10	0.10	0.10	0.10	0.10	0.02	0.10	NA	NA	NA	NA	NA	NA	NA
316	0.10	0.10	0.10	0.10	0.10	0.10	0.10	0.10	NA	NA	NA	NA	NA	NA
317	0.10	0.10	0.10	0.10	0.05	0.10	0.10	0.10	NA	NA	NA	NA	NA	NA
318	0.10	0.10	0.10	0.10	0.10	0.10	0.10	0.10	NA	NA	NA	NA	NA	NA
319	0.10	0.10	0.10	0.10	0.10	0.10	0.10	0.10	NA	NA	NA	NA	NA	NA
320	0.10	0.10	0.10	0.10	0.08	0.10	0.10	0.10	NA	NA	NA	NA	NA	NA
321	0.10	0.10	0.10	0.10	0.10	0.10	0.10	0.10	NA	NA	NA	NA	NA	NA
322	0.10	0.10	0.10	0.10	0.10	0.10	0.10	0.10	NA	NA	NA	NA	NA	NA
323	0.10	0.10	0.10	0.10	0.10	0.10	0.10	0.10	NA	NA	NA	NA	NA	NA
324	0.10	0.10	0.10	0.10	0.10	0.10	0.10	0.10	NA	NA	NA	NA	NA	NA
325	0.10	0.10	0.10	0.10	0.10	0.10	0.10	0.09	NA	NA	NA	NA	NA	NA
326	0.10	0.10	0.10	0.10	0.10	0.10	0.10	0.10	NA	NA	NA	NA	NA	NA
327	0.10	0.10	0.10	0.10	0.10	0.10	0.10	0.10	NA	NA	NA	NA	NA	NA
328	0.10	0.10	0.10	0.04	0.10	0.09	0.10	0.10	NA	NA	NA	NA	NA	NA
329	0.10	0.10	0.10	0.10	0.10	0.10	0.10	0.10	NA	NA	NA	NA	NA	NA
330	0.10	0.10	0.10	0.10	0.06	0.10	0.10	0.08	NA	NA	NA	NA	NA	NA
331	0.10	0.10	0.10	0.08	0.10	0.10	0.10	0.08	NA	NA	NA	NA	NA	NA
332	0.10	0.10	0.10	0.10	0.10	0.10	0.09	0.10	NA	NA	NA	NA	NA	NA

333	0.10	0.10	0.10	0.10	0.10	0.10	0.10	0.10	NA	NA	NA	NA	NA	NA
334	0.10	0.10	0.10	0.10	0.10	0.10	0.10	0.10	NA	NA	NA	NA	NA	NA
335	0.10	0.10	0.10	0.10	0.10	0.10	0.10	0.10	NA	NA	NA	NA	NA	NA
336	0.10	0.10	0.10	0.10	0.10	0.10	0.10	0.08	NA	NA	NA	NA	NA	NA
337	0.10	0.10	0.10	0.10	0.10	0.10	0.10	0.10	NA	NA	NA	NA	NA	NA
338	0.10	0.10	0.10	0.10	0.10	0.10	0.10	0.10	0.10	0.10	NA	NA	0.10	0.10
339	0.10	0.10	0.10	0.10	0.04	0.10	0.10	0.10	0.10	0.10	NA	NA	0.10	0.10
340	0.10	0.10	0.10	0.10	0.10	0.10	0.10	0.10	NA	NA	NA	NA	NA	NA
341	0.10	0.10	0.10	0.10	0.10	0.10	0.10	0.10	NA	NA	NA	NA	NA	NA
342	0.10	0.10	0.10	0.10	0.10	0.10	0.10	NA	NA	NA	NA	NA	NA	NA
343	0.10	0.10	0.10	0.10	0.10	0.10	0.10	NA	NA	NA	NA	NA	NA	NA
344	0.10	0.10	0.10	0.10	0.10	0.10	0.10	NA	NA	NA	NA	NA	NA	NA
345	0.10	0.10	0.10	0.10	0.10	0.10	0.10	0.10	NA	NA	NA	NA	NA	NA
346	0.10	0.10	0.10	0.10	0.10	0.10	0.10	0.10	NA	NA	NA	NA	NA	NA
347	0.10	0.10	0.10	0.09	0.10	0.10	0.10	0.10	NA	NA	NA	NA	NA	NA
348	0.10	0.10	0.10	0.10	0.10	0.10	0.08	0.10	NA	NA	NA	NA	NA	NA
349	0.10	0.10	0.10	0.10	0.10	0.10	0.10	0.10	NA	NA	NA	NA	NA	NA
350	0.10	0.10	0.10	0.10	0.10	0.10	0.04	0.10	NA	NA	NA	NA	NA	NA
351	0.10	0.10	0.10	0.10	0.10	0.10	0.08	0.10	NA	NA	NA	NA	NA	NA
352	0.10	0.10	0.10	0.10	0.09	0.10	0.10	0.10	NA	NA	NA	NA	NA	NA
353	0.10	0.10	0.10	0.10	0.10	0.10	0.10	0.10	NA	NA	NA	NA	NA	NA
354	0.10	0.10	0.10	0.07	0.10	0.10	0.10	0.10	NA	NA	NA	NA	NA	NA
355	0.10	0.10	0.10	0.10	0.10	0.10	0.10	0.10	NA	NA	NA	NA	NA	NA
356	0.10	0.10	0.10	0.10	0.10	0.10	0.10	0.10	NA	NA	NA	NA	NA	NA
357	0.10	0.10	0.10	0.10	0.10	0.10	0.10	0.10	NA	NA	NA	NA	NA	NA
358	0.10	0.10	0.10	0.10	0.10	0.10	0.10	0.10	NA	NA	NA	NA	NA	NA
359	0.10	0.10	0.10	0.10	0.10	0.08	0.10	0.10	NA	NA	NA	NA	NA	NA
360	0.10	0.10	0.10	0.10	0.10	0.10	0.10	0.10	NA	NA	NA	NA	NA	NA
361	0.10	0.10	0.10	0.10	0.10	0.10	0.04	0.10	0.10	0.10	NA	NA	0.10	0.10
362	0.10	0.10	0.10	0.10	0.04	0.10	0.10	NA	NA	NA	NA	NA	NA	NA
363	0.10	0.10	0.10	0.10	0.10	0.10	0.10	0.10	NA	NA	NA	NA	NA	NA
364	0.10	0.10	0.10	0.04	0.10	0.04	0.10	0.10	NA	NA	NA	NA	NA	NA
365	0.10	0.10	0.10	0.10	0.10	0.10	0.10	0.10	NA	NA	NA	NA	NA	NA
366	0.10	0.10	0.10	0.10	0.10	0.10	0.10	0.10	0.10	0.10	NA	NA	0.10	0.10
367	0.10	0.10	0.10	0.10	0.04	0.10	0.10	NA	NA	NA	NA	NA	NA	NA
368	0.10	0.10	0.10	0.10	0.10	0.10	0.10	0.10	NA	NA	NA	NA	NA	NA
369	0.10	0.10	0.10	0.10	0.10	0.10	0.10	0.10	NA	NA	NA	NA	NA	NA
370	0.10	0.10	0.10	0.10	0.10	0.10	0.05	0.10	NA	NA	NA	NA	NA	NA
371	0.10	0.10	0.10	0.10	0.10	0.10	0.10	0.10	NA	NA	NA	NA	NA	NA
372	0.10	0.10	0.10	0.10	0.10	0.10	0.10	0.10	NA	NA	NA	NA	NA	NA
373	0.10	0.10	0.10	0.10	0.10	0.10	0.10	0.10	NA	NA	NA	NA	NA	NA
374	0.10	0.10	0.10	0.10	0.10	0.10	0.10	0.10	NA	NA	NA	NA	NA	NA
375	0.10	0.10	0.10	0.10	0.10	0.10	0.10	NA	NA	NA	NA	NA	NA	NA
376	0.10	0.10	0.10	0.10	0.10	0.10	0.10	0.10	NA	NA	NA	NA	NA	NA

Table S3 - Grid search results of one patient for the raw physiologic signals with respect to AIC, BIC and LL values

ARIMA Model	MAP_AIC	MAP_BIC	MAP_LL	ICP_AIC	ICP_BIC	ICP_LL	CPP_AIC	CPP_BIC	CPP_LL	rSO_2_L_AIC	rSO_2_L_BIC	rSO_2_L_LL	rSO_2_R_AIC	rSO_2_R_BIC	rSO_2_R_LL	$PbtO_2$_AIC	$PbtO_2$_BIC	$PbtO_2$_LL
(1,1,0)	39873.96	39894.52	-19933.98	28444.06	28464.61	-14219.03	39280.46	39300.94	-19637.23	18937.73	18958.37	-9465.86	17851.28	17871.92	-8922.64	3198.84	3213.70	-1596.42
(1,1,1)	39794.90	39822.31	-19893.45	28409.59	28437.01	-14200.80	39172.42	39199.72	-19582.21	18819.33	18846.84	-9405.66	17500.83	17528.34	-8746.41	3169.26	3189.08	-1580.63
(1,1,2)	39796.56	39830.82	-19893.28	28154.31	28188.57	-14072.15	39160.46	39194.58	-19575.23	18396.32	18430.71	-9193.16	17106.13	17140.53	-8548.07	3170.14	3194.92	-1580.07
(1,1,3)	39793.00	39834.11	-19890.50	28120.40	28161.51	-14054.20	39155.54	39196.49	-19571.77	18385.83	18427.10	-9186.91	17100.43	17141.71	-8544.22	3166.84	3196.58	-1577.42
(1,1,4)	39794.32	39842.28	-19890.16	28122.26	28170.23	-14054.13	39157.25	39205.02	-19571.62	18380.55	18428.70	-9183.28	17100.30	17148.45	-8543.15	3168.84	3203.53	-1577.42
(1,1,5)	39795.12	39849.93	-19889.56	28123.73	28178.56	-14053.87	39158.80	39213.40	-19571.40	18368.06	18423.10	-9176.03	17106.52	17161.56	-8545.26	3170.28	3209.93	-1577.14
(1,1,6)	39791.15	39852.81	-19886.57	28124.70	28186.38	-14053.35	39153.63	39215.05	-19567.82	18366.78	18428.69	-9174.39	17100.19	17162.11	-8541.10	3172.25	3216.86	-1577.13
(1,1,7)	39788.26	39856.77	-19884.13	28126.88	28195.41	-14053.44	39146.31	39214.56	-19563.16	18346.23	18415.02	-9163.11	17107.15	17175.94	-8543.57	3173.45	3223.00	-1576.72
(1,1,8)	39784.91	39860.27	-19881.45	28128.86	28204.24	-14053.43	39143.12	39218.19	-19560.56	18332.94	18408.61	-9155.47	17102.92	17178.60	-8540.46	3174.45	3228.96	-1576.23
(1,1,9)	39784.23	39866.45	-19880.12	28125.36	28207.59	-14050.68	39143.61	39225.50	-19559.80	18324.45	18407.00	-9150.22	17096.17	17178.72	-8536.08	3176.41	3235.87	-1576.20
(1,1,10)	39781.73	39870.80	-19877.87	28126.66	28215.75	-14050.33	39135.58	39224.30	-19554.79	18322.88	18412.31	-9148.44	17098.16	17187.60	-8536.08	3177.51	3241.93	-1575.75
(1,2,0)	43239.70	43253.40	-21617.85	31386.51	31400.21	-15691.25	42498.67	42512.32	-21247.34	22174.57	22188.33	-11085.28	21491.34	21505.10	-10743.67	3655.50	3665.41	-1825.75
(1,2,1)	39878.32	39898.88	-19936.16	28449.55	28470.11	-14221.78	39284.90	39305.37	-19639.45	18944.65	18965.29	-9469.33	17858.54	17879.18	-8926.27	3203.84	3218.70	-1598.92
(1,2,2)	39799.67	39827.07	-19895.83	28415.14	28442.55	-14203.57	39294.70	39321.99	-19643.35	18826.30	18853.81	-9409.15	17743.11	17770.63	-8867.56	3206.00	3225.81	-1599.00
(1,2,3)	39864.81	39899.07	-19927.41	28426.49	28460.75	-14208.24	39282.41	39316.53	-19636.20	18865.94	18900.33	-9427.97	17119.16	17153.55	-8554.58	3207.68	3232.45	-1598.84
(1,2,4)	39820.89	39862.00	-19904.44	28399.30	28440.41	-14193.65	39204.27	39245.21	-19596.13	18396.13	18437.40	-9192.06	17109.86	17151.14	-8548.93	3175.03	3204.75	-1581.51
(1,2,5)	39800.54	39848.50	-19893.27	28139.38	28187.35	-14062.69	39168.35	39216.12	-19577.18	18393.63	18441.78	-9189.82	17150.32	17198.48	-8568.16	3179.30	3213.98	-1582.65
(1,2,6)	39794.31	39849.12	-19889.16	28168.05	28222.87	-14076.03	39156.40	39211.00	-19570.20	18435.87	18490.90	-9209.94	17122.31	17177.35	-8553.15	3182.54	3222.17	-1583.27
(1,2,7)	39797.05	39858.71	-19889.52	28150.83	28212.50	-14066.41	39158.41	39219.83	-19570.20	18422.18	18484.09	-9202.09	17116.05	17177.97	-8549.03	3182.44	3227.03	-1582.22
(1,2,8)	39793.04	39861.55	-19886.52	28134.35	28202.88	-14057.18	39151.16	39219.41	-19565.58	18422.05	18490.84	-9201.02	17117.78	17186.58	-8548.89	3183.93	3233.48	-1581.97
(1,2,9)	39789.74	39865.10	-19883.87	28135.70	28211.08	-14056.85	39147.94	39223.01	-19562.97	18403.57	18479.24	-9190.79	17118.20	17193.87	-8548.10	3182.16	3236.66	-1580.08
(1,2,10)	39789.02	39871.23	-19882.51	28137.70	28219.93	-14056.85	39148.45	39230.35	-19562.23	18370.17	18452.72	-9173.08	17120.06	17202.62	-8548.03	3183.14	3242.60	-1579.57
(2,1,0)	39851.96	39879.36	-19921.98	28351.37	28378.78	-14171.69	39239.17	39266.47	-19615.59	18642.78	18670.29	-9317.39	17424.09	17451.61	-8708.05	3200.02	3219.85	-1596.01
(2,1,1)	39796.52	39830.78	-19893.26	28123.97	28158.24	-14056.99	39159.14	39193.27	-19574.57	18417.12	18451.52	-9203.56	17120.12	17154.52	-8555.06	3169.95	3194.73	-1579.97
(2,1,2)	39785.94	39827.05	-19886.97	28125.17	28166.29	-14056.58	39157.55	39198.50	-19572.78	18390.24	18431.51	-9189.12	17102.09	17143.37	-8545.05	3170.38	3200.11	-1579.19
(2,1,3)	39787.15	39835.11	-19886.58	28122.29	28170.26	-14054.14	39157.10	39204.88	-19571.55	18386.52	18434.68	-9186.26	17100.36	17148.52	-8543.18	3168.84	3203.53	-1577.42
(2,1,4)	39795.66	39850.47	-19889.83	28122.29	28177.12	-14053.15	39159.10	39213.70	-19571.55	18376.89	18431.92	-9180.44	17101.85	17156.89	-8542.93	3170.81	3210.46	-1577.41
(2,1,5)	39793.26	39854.92	-19887.63	28124.19	28185.87	-14053.10	39161.09	39222.51	-19571.55	18369.06	18430.97	-9175.53	17103.21	17165.12	-8542.60	3172.27	3216.87	-1577.14
(2,1,6)	39792.96	39861.47	-19886.48	28126.19	28194.71	-14053.09	39154.75	39223.00	-19567.38	18339.52	18408.31	-9159.76	17099.69	17168.49	-8539.85	3174.11	3223.67	-1577.06
(2,1,7)	39777.85	39853.21	-19877.92	28128.26	28203.64	-14053.13	39127.10	39202.17	-19552.55	18321.69	18397.36	-9149.85	17101.98	17177.65	-8539.99	3175.30	3229.81	-1576.65
(2,1,8)	39770.91	39853.12	-19873.45	28127.03	28209.26	-14051.52	39127.28	39209.17	-19551.64	18320.29	18402.84	-9148.15	17103.80	17186.36	-8539.90	3175.54	3235.01	-1575.77
(2,1,9)	39770.75	39859.82	-19872.37	28117.56	28206.65	-14045.78	39126.38	39215.10	-19550.19	18321.54	18410.96	-9147.77	17098.16	17187.59	-8536.08	3178.45	3242.87	-1576.22
(2,1,10)	39765.32	39861.24	-19868.66	28116.73	28212.67	-14044.37	39113.88	39209.43	-19542.94	18324.12	18420.42	-9148.06	17100.16	17196.48	-8536.08	3177.37	3246.75	-1574.68
(2,2,0)	42401.77	42422.32	-21197.88	30849.13	30869.69	-15421.56	41740.93	41761.40	-20867.46	21446.49	21467.12	-10720.24	20486.54	20507.18	-10240.27	3567.23	3582.09	-1780.62
(2,2,1)	39856.43	39883.84	-19924.22	28357.11	28384.52	-14174.56	39243.48	39270.78	-19617.74	18650.10	18677.61	-9321.05	17431.85	17459.36	-8711.92	3205.08	3224.90	-1598.54
(2,2,2)	39877.51	39911.76	-19933.75	28131.08	28165.34	-14060.54	39285.44	39319.56	-19637.72	18425.98	18460.38	-9207.99	17129.29	17163.68	-8559.64	3207.83	3232.60	-1598.91
(2,2,3)	39929.12	39970.22	-19958.56	28145.45	28186.56	-14066.72	39290.39	39331.34	-19639.20	18400.72	18441.99	-9194.36	17112.70	17153.97	-8550.35	3199.48	3229.21	-1593.74
(2,2,4)	39848.84	39896.80	-19917.42	28421.41	28469.38	-14203.71	39169.31	39217.08	-19577.65	18870.82	18918.98	-9428.41	17119.85	17168.01	-8552.93	3208.07	3242.76	-1597.04
(2,2,5)	39811.37	39866.18	-19897.68	28362.35	28417.17	-14173.18	39189.45	39244.04	-19586.72	18613.97	18669.00	-9298.98	17347.53	17402.56	-8665.76	3207.62	3247.26	-1595.81
(2,2,6)	39791.76	39853.42	-19886.88	28220.39	28282.07	-14101.20	39141.91	39203.33	-19561.96	18441.53	18503.44	-9211.76	17140.92	17202.83	-8561.46	3195.76	3240.35	-1588.88
(2,2,7)	39792.05	39860.56	-19886.02	28165.31	28233.84	-14072.66	39154.68	39222.92	-19567.34	18433.39	18502.18	-9206.70	17117.52	17186.31	-8548.76	3183.70	3233.25	-1581.85
(2,2,8)	39776.58	39851.95	-19877.29	28137.16	28212.54	-14057.58	39131.98	39207.05	-19554.99	18424.17	18499.84	-9201.08	17112.45	17188.12	-8545.22	3182.76	3237.26	-1580.38
(2,2,9)	39775.73	39857.95	-19875.87	28123.04	28205.27	-14049.52	39132.23	39214.12	-19554.11	18421.37	18503.91	-9198.68	17113.65	17196.21	-8544.83	3187.82	3247.27	-1581.91
(2,2,10)	39775.54	39864.61	-19874.77	28124.79	28213.87	-14049.39	39131.33	39220.05	-19552.66	18411.24	18500.66	-9192.62	17115.54	17204.98	-8544.77	3182.54	3246.95	-1578.27
(3,1,0)	39810.49	39844.74	-19900.24	28245.05	28279.31	-14117.52	39184.28	39218.40	-19587.14	18526.67	18561.06	-9258.33	17312.26	17346.66	-8651.13	3189.49	3214.27	-1589.75
(3,1,1)	39793.01	39834.12	-19890.50	28124.92	28166.04	-14056.46	39156.15	39197.10	-19572.08	18376.39	18417.67	-9182.20	17098.57	17139.85	-8543.28	3167.37	3197.10	-1577.68

(3,1,2)	39787.11	39835.07	-19886.56	28127.96	28175.93	-14056.98	39149.95	39197.72	-19567.97	18366.18	18414.33	-9176.09	17098.42	17146.58	-8542.21	3169.19	3203.88	-1577.60
(3,1,3)	39788.49	39843.30	-19886.24	28124.08	28178.90	-14054.04	39159.12	39213.71	-19571.56	18353.47	18408.50	-9168.73	17103.20	17158.23	-8543.60	3169.12	3208.77	-1576.56
(3,1,4)	39790.49	39852.16	-19886.25	28124.35	28186.02	-14053.17	39161.11	39222.53	-19571.55	18334.01	18395.92	-9158.01	17103.52	17165.43	-8542.76	3169.44	3214.04	-1575.72
(3,1,5)	39799.04	39867.56	-19889.52	28120.75	28189.28	-14050.38	39162.81	39231.06	-19571.41	18357.04	18425.83	-9168.52	17105.35	17174.15	-8542.68	3168.27	3217.83	-1574.13
(3,1,6)	39792.26	39867.62	-19885.13	28124.22	28199.60	-14051.11	39138.25	39213.32	-19558.12	18320.26	18395.93	-9149.13	17101.78	17177.46	-8539.89	3167.90	3222.41	-1572.95
(3,1,7)	39787.28	39869.50	-19881.64	28117.80	28200.03	-14046.90	39143.42	39225.31	-19559.71	18320.34	18402.89	-9148.17	17108.98	17191.54	-8542.49	3172.11	3231.57	-1574.05
(3,1,8)	39789.42	39878.49	-19881.71	28122.07	28211.16	-14048.04	39143.98	39232.70	-19558.99	18322.00	18411.43	-9148.00	17087.77	17177.20	-8530.88	3170.79	3235.21	-1572.40
(3,1,9)	39790.11	39886.03	-19881.06	28119.26	28215.20	-14045.63	39118.15	39213.69	-19545.07	18323.25	18419.55	-9147.62	17099.66	17195.98	-8535.83	3171.45	3240.83	-1571.73
(3,1,10)	39762.20	39864.97	-19866.10	28119.01	28221.80	-14044.51	39109.79	39212.15	-19539.89	18325.63	18428.82	-9147.82	17101.09	17204.28	-8535.54	3176.96	3251.30	-1573.48
(3,2,0)	41799.07	41826.47	-20895.53	30357.91	30385.32	-15174.95	41165.75	41193.05	-20578.88	20820.74	20848.26	#######	19843.40	19870.92	-9917.70	3504.82	3524.64	-1748.41
(3,2,1)	39815.12	39849.38	-19902.56	28251.03	28285.29	-14120.51	39188.77	39222.89	-19589.38	18534.24	18568.64	-9262.12	17320.27	17354.66	-8655.13	3194.77	3219.54	-1592.38
(3,2,2)	39839.51	39880.62	-19913.76	28360.24	28401.36	-14174.12	39223.35	39264.29	-19605.67	18502.46	18543.74	-9245.23	17143.37	17184.65	-8565.68	3173.83	3203.56	-1580.92
(3,2,3)	39858.15	39906.11	-19922.08	28134.79	28182.76	-14060.39	39268.02	39315.79	-19627.01	18426.67	18474.83	-9206.34	17133.13	17181.28	-8559.56	3211.71	3246.39	-1598.85
(3,2,4)	39801.57	39856.38	-19892.78	28135.95	28190.77	-14059.97	39166.34	39220.93	-19575.17	18399.24	18454.27	-9191.62	17115.24	17170.27	-8549.62	3203.83	3243.47	-1593.92
(3,2,5)	39799.78	39861.44	-19890.89	28153.02	28214.69	-14067.51	39157.53	39218.95	-19569.77	18411.66	18473.57	-9196.83	17123.28	17185.20	-8552.64	3210.74	3255.33	-1596.37
(3,2,6)	39796.89	39865.41	-19888.45	28137.29	28205.82	-14058.65	39177.77	39246.01	-19578.88	18412.33	18481.12	-9196.16	17111.03	17179.83	-8545.52	3200.78	3250.33	-1590.39
(3,2,7)	39801.65	39877.02	-19889.83	28230.97	28306.35	-14104.49	39169.33	39244.40	-19573.67	18518.25	18593.92	-9248.13	17152.37	17228.04	-8565.18	3199.82	3254.32	-1588.91
(3,2,8)	39767.54	39849.75	-19871.77	28146.94	28229.17	-14061.47	39124.07	39205.96	-19550.03	18443.40	18525.95	-9209.70	17125.04	17207.59	-8550.52	3182.68	3242.14	-1579.34
(3,2,9)	39769.51	39858.58	-19871.76	28143.52	28232.61	-14058.76	39127.97	39216.68	-19550.98	18424.92	18514.35	-9199.46	17116.91	17206.35	-8545.46	3181.81	3246.22	-1577.91
(3,2,10)	39769.02	39864.94	-19870.51	28127.29	28223.22	-14049.64	39123.06	39218.60	-19547.53	18431.44	18527.75	-9201.72	17118.41	17214.72	-8545.21	3182.12	3251.48	-1577.06
(4,1,0)	39804.83	39845.94	-19896.42	28209.23	28250.35	-14098.62	39174.16	39215.11	-19581.08	18495.36	18536.64	-9241.68	17241.45	17282.73	-8614.73	3182.72	3212.46	-1585.36
(4,1,1)	39794.24	39842.20	-19890.12	28121.90	28169.87	-14053.95	39157.54	39205.31	-19571.77	18360.14	18408.29	-9173.07	17099.72	17147.88	-8542.86	3169.06	3203.75	-1577.53
(4,1,2)	39788.32	39843.13	-19886.16	28123.66	28178.48	-14053.83	39151.39	39205.99	-19567.70	18379.95	18434.98	-9181.97	17100.03	17155.07	-8542.02	3171.36	3211.00	-1577.68
(4,1,3)	39790.62	39852.28	-19886.31	28123.43	28185.10	-14052.71	39153.07	39214.49	-19567.54	18370.18	18432.09	-9176.09	17101.93	17163.84	-8541.96	3171.62	3216.22	-1576.81
(4,1,4)	39790.63	39859.14	-19885.31	28122.10	28190.63	-14051.05	39154.96	39223.20	-19567.48	18341.87	18410.66	-9160.93	17105.69	17174.48	-8542.84	3169.82	3219.37	-1574.91
(4,1,5)	39784.13	39859.49	-19881.06	28121.89	28197.27	-14049.95	39146.86	39221.93	-19562.43	18667.00	18742.67	-9322.50	17106.71	17182.38	-8542.35	3169.44	3223.95	-1573.72
(4,1,6)	39790.65	39872.87	-19883.33	28124.88	28207.11	-14050.44	39158.34	39240.23	-19567.17	18323.98	18406.53	-9149.99	17101.73	17184.28	-8538.86	3184.89	3244.35	-1580.44
(4,1,7)	39778.82	39867.89	-19876.41	28116.41	28205.49	-14045.20	39134.35	39223.07	-19554.18	18322.88	18412.31	-9148.44	17107.34	17196.77	-8540.67	3169.41	3233.83	-1571.71
(4,1,8)	39779.59	39875.51	-19875.80	28123.89	28219.83	-14047.95	39136.24	39231.78	-19554.12	18324.31	18420.61	-9148.15	17105.84	17202.15	-8538.92	3172.68	3242.05	-1572.34
(4,1,9)	39777.60	39880.37	-19873.80	28124.01	28226.81	-14047.01	39110.41	39212.78	-19540.21	18323.19	18426.38	-9146.60	17097.93	17201.12	-8533.96	3175.76	3250.09	-1572.88
(4,1,10)	39757.66	39867.28	-19862.83	28118.83	28228.48	-14043.42	39102.60	39211.79	-19535.30	18324.93	18435.00	-9146.47	17100.48	17210.56	-8534.24	3177.89	3257.18	-1572.95
(4,2,0)	41447.39	41481.65	-20718.69	30010.76	30045.02	-15000.38	40814.53	40848.66	-20402.27	20275.91	20310.30	-10132.96	19418.24	19452.63	-9704.12	3450.09	3474.86	-1720.05
(4,2,1)	39809.53	39850.64	-19898.77	28215.35	28256.47	-14101.68	39178.74	39219.68	-19583.37	18503.07	18544.35	-9245.54	17249.66	17290.93	-8618.83	3188.18	3217.91	-1588.09
(4,2,2)	39815.65	39863.61	-19900.83	28156.55	28204.52	-14071.27	39190.73	39238.50	-19588.37	18534.26	18582.41	-9260.13	17324.10	17372.25	-8655.05	3198.10	3232.78	-1592.05
(4,2,3)	39829.46	39884.27	-19906.73	28135.98	28190.80	-14059.99	39210.00	39264.60	-19597.00	18588.36	18643.40	-9286.18	17427.02	17482.06	-8705.51	3210.62	3250.26	-1597.31
(4,2,4)	39797.28	39858.94	-19889.64	28136.66	28198.33	-14059.33	39170.00	39231.41	-19576.00	18378.77	18440.68	-9180.39	17136.51	17198.43	-8559.26	3204.24	3248.83	-1593.12
(4,2,5)	39797.74	39866.26	-19888.87	NA	NA	NA	39166.07	39234.31	-19573.03	18406.98	18475.77	-9193.49	17121.87	17190.67	-8550.94	3207.97	3257.52	-1593.99
(4,2,6)	39788.80	39864.17	-19883.40	28420.56	28495.94	-14199.28	39144.27	39219.34	-19561.14	18405.18	18480.84	-9191.59	17118.14	17193.81	-8548.07	3210.10	3264.60	-1594.05
(4,2,7)	39801.65	39883.87	-19888.83	28138.00	28220.23	-14057.00	39160.77	39242.67	-19568.39	18406.39	18488.94	-9191.20	17137.59	17220.14	-8556.79	3181.19	3240.64	-1578.59
(4,2,8)	39785.40	39874.46	-19879.70	28186.64	28275.73	-14080.32	39130.17	39218.88	-19552.08	18429.66	18519.08	-9201.83	17156.06	17245.50	-8565.03	3198.70	3263.11	-1586.35
(4,2,9)	39786.68	39882.60	-19879.34	28161.38	28257.32	-14066.69	39128.81	39224.35	-19550.40	18436.62	18532.92	-9204.31	17126.68	17222.99	-8549.34	3186.08	3255.45	-1579.04
(4,2,10)	39769.88	39872.65	-19869.94	28143.80	28246.59	-14056.90	39115.95	39218.32	-19542.98	18432.99	18536.17	-9201.50	17122.24	17225.44	-8546.12	3184.13	3258.45	-1577.07
(5,1,0)	39797.77	39845.73	-19891.88	28185.56	28233.53	-14085.78	39165.31	39213.09	-19575.66	18496.81	18544.97	-9241.41	17181.83	17229.99	-8583.92	3181.63	3216.32	-1583.82
(5,1,1)	39795.17	39849.98	-19889.59	28123.80	28178.62	-14053.90	39158.26	39212.85	-19571.13	18361.68	18416.71	-9172.84	17100.55	17155.58	-8542.27	3170.52	3210.17	-1577.26
(5,1,2)	39790.24	39851.90	-19886.12	28124.32	28186.00	-14053.16	39153.17	39214.60	-19567.59	18362.28	18424.19	-9172.14	17101.99	17163.91	-8542.00	3172.52	3217.12	-1577.26
(5,1,3)	39793.32	39861.84	-19886.66	28130.35	28198.88	-14055.18	39150.33	39218.57	-19565.16	18330.85	18399.64	-9155.42	17103.85	17172.65	-8541.93	3170.97	3220.53	-1575.49
(5,1,4)	39801.00	39876.37	-19889.50	28122.60	28197.98	-14050.30	39152.95	39228.02	-19565.48	18325.82	18401.49	-9151.91	17101.75	17177.43	-8539.88	3174.60	3229.12	-1576.30
(5,1,5)	39783.31	39865.53	-19879.66	28124.38	28206.62	-14050.19	39149.34	39231.24	-19562.67	18342.26	18424.81	-9159.13	17106.31	17188.87	-8541.16	3172.80	3232.27	-1574.40
(5,1,6)	39796.55	39885.62	-19885.27	28119.55	28208.63	-14046.77	39148.71	39237.42	-19561.35	18326.91	18416.33	-9150.45	17100.67	17190.10	-8537.33	3182.97	3247.39	-1578.48

(5,1,7)	39780.01	39875.93	-19876.00	28117.64	28213.57	-14044.82	39134.40	39229.94	-19553.20	18324.40	18420.71	-9148.20	17106.07	17202.38	-8539.03	3178.08	3247.46	-1575.04
(5,1,8)	39780.95	39883.72	-19875.48	28118.40	28221.19	-14044.20	39136.23	39238.59	-19553.11	18327.93	18431.11	-9148.96	17101.95	17205.14	-8535.97	3173.40	3247.73	-1571.70
(5,1,9)	39766.92	39876.54	-19867.46	28122.85	28232.49	-14045.42	39101.72	39210.91	-19534.86	18325.23	18435.29	-9146.61	17100.23	17210.31	-8534.12	3177.35	3256.64	-1572.67
(5,1,10)	39757.17	39873.65	-19861.59	28122.39	28238.89	-14044.20	39102.56	39218.58	-19534.28	18326.25	18443.20	-9146.13	17103.43	17220.38	-8534.72	3176.73	3260.98	-1571.36
(5,2,0)	41190.23	41231.34	-20589.12	29780.25	29821.37	-14884.12	40572.16	40613.11	-20280.08	19979.57	20020.84	-9983.78	19038.05	19079.33	-9513.03	3408.91	3438.64	-1698.46
(5,2,1)	39802.54	39850.50	-19894.27	28191.80	28239.77	-14088.90	39169.97	39217.74	-19577.99	18504.54	18552.69	-9245.27	17190.22	17238.38	-8588.11	3187.20	3221.88	-1586.60
(5,2,2)	39802.21	39857.02	-19893.10	28219.34	28274.16	-14101.67	39169.95	39224.54	-19576.97	18503.01	18558.04	-9243.51	17164.41	17219.45	-8574.20	3192.18	3231.81	-1588.09
(5,2,3)	39804.83	39866.49	-19893.42	28238.33	28300.00	-14110.17	39177.62	39239.04	-19579.81	18537.26	18599.17	-9259.63	17312.19	17374.11	-8647.10	3201.88	3246.47	-1591.94
(5,2,4)	39829.84	39898.35	-19904.92	28136.96	28205.49	-14058.48	39202.12	39270.36	-19591.06	18392.56	18461.35	-9186.28	17304.81	17373.60	-8642.40	3178.94	3228.49	-1579.47
(5,2,5)	39805.31	39880.67	-19891.65	28135.62	28211.00	-14056.81	39170.40	39245.46	-19574.20	18375.47	18451.14	-9176.74	17135.10	17210.77	-8556.55	3175.71	3230.22	-1576.86
(5,2,6)	39770.66	39852.88	-19873.33	28133.35	28215.59	-14054.68	39132.74	39214.63	-19554.37	18410.88	18493.43	-9193.44	17120.67	17203.23	-8548.34	3182.14	3241.59	-1579.07
(5,2,7)	39836.37	39925.44	-19905.18	28178.37	28267.45	-14076.18	39130.70	39219.42	-19552.35	18408.81	18498.23	-9191.40	17116.72	17206.15	-8545.36	3186.97	3251.39	-1580.49
(5,2,8)	39784.14	39880.06	-19878.07	28142.24	28238.18	-14057.12	39129.58	39225.12	-19550.79	18406.30	18502.61	-9189.15	17127.69	17224.01	-8549.85	3182.37	3251.73	-1577.18
(5,2,9)	39780.10	39882.87	-19875.05	28226.69	28329.48	-14098.34	39132.40	39234.77	-19551.20	18435.89	18539.07	-9202.94	17142.58	17245.78	-8556.29	3188.62	3262.94	-1579.31
(5,2,10)	39760.96	39870.58	-19864.48	28149.05	28258.69	-14058.52	39106.67	39215.86	-19537.33	18444.75	18554.82	-9206.38	17130.57	17240.64	-8549.28	3187.60	3266.87	-1577.80
(6,1,0)	39795.10	39849.91	-19889.55	28158.55	28213.37	-14071.27	39158.41	39213.01	-19571.20	18494.90	18549.93	-9239.45	17162.02	17217.06	-8573.01	3182.05	3221.69	-1583.02
(6,1,1)	39796.86	39858.52	-19889.43	28125.71	28187.39	-14053.86	39159.11	39220.53	-19570.56	18343.66	18405.57	-9162.83	17096.73	17158.64	-8539.36	3172.52	3217.12	-1577.26
(6,1,2)	39797.21	39865.72	-19888.60	28126.91	28195.44	-14053.46	39161.06	39229.30	-19570.53	18329.89	18398.68	-9154.95	17099.65	17168.45	-8539.83	3174.50	3224.06	-1577.25
(6,1,3)	39796.10	39871.47	-19887.05	28114.66	28190.04	-14046.33	39151.92	39226.99	-19564.96	18362.04	18437.71	-9170.02	17102.35	17178.03	-8540.18	3172.35	3226.86	-1575.17
(6,1,4)	39789.17	39871.39	-19882.59	28124.31	28206.55	-14050.16	39156.22	39238.11	-19566.11	18323.54	18406.08	-9149.77	17100.10	17182.65	-8538.05	3172.10	3231.57	-1574.05
(6,1,5)	39785.33	39874.39	-19879.66	28119.03	28208.12	-14046.52	39150.61	39239.33	-19562.31	18673.19	18762.62	-9323.59	17103.82	17193.25	-8538.91	3175.29	3239.71	-1574.64
(6,1,6)	39781.85	39877.77	-19876.93	28126.70	28222.64	-14049.35	39137.66	39233.20	-19554.83	18325.14	18421.45	-9148.57	17103.20	17199.51	-8537.60	3191.27	3260.65	-1581.63
(6,1,7)	39771.79	39874.56	-19870.90	28129.26	28232.05	-14049.63	39142.76	39245.13	-19556.38	18326.12	18429.31	-9148.06	17099.98	17203.18	-8534.99	3175.60	3249.94	-1572.80
(6,1,8)	39771.48	39881.10	-19869.74	28110.04	28219.69	-14039.02	39137.30	39246.49	-19552.65	18328.69	18438.75	-9148.34	17100.73	17210.80	-8534.36	3173.34	3252.63	-1570.67
(6,1,9)	39771.68	39888.15	-19868.84	28128.11	28244.61	-14047.05	39102.53	39218.55	-19534.27	18327.38	18444.32	-9146.69	17102.84	17219.79	-8534.42	3179.32	3263.56	-1572.66
(6,1,10)	39759.73	39883.06	-19861.87	28130.33	28253.68	-14047.16	39104.87	39227.72	-19534.44	18328.05	18451.87	-9146.03	17105.16	17229.00	-8534.58	3175.63	3264.83	-1569.82
(6,2,0)	40971.46	41019.41	-20478.73	29524.84	29572.81	-14755.42	40361.23	40409.00	-20173.61	19840.32	19888.47	-9913.16	18753.30	18801.45	-9369.65	3389.18	3423.86	-1687.59
(6,2,1)	39799.92	39854.73	-19891.96	28164.92	28219.74	-14074.46	39163.14	39217.73	-19573.57	18502.67	18557.70	-9243.34	17170.52	17225.55	-8577.26	3187.69	3227.33	-1585.85
(6,2,2)	39801.52	39863.18	-19891.76	28150.78	28212.46	-14066.39	39168.30	39229.72	-19575.15	18504.26	18566.17	-9243.13	17194.35	17256.27	-8588.18	3190.95	3235.55	-1586.48
(6,2,3)	39792.07	39860.58	-19886.03	28213.22	28281.75	-14096.61	39166.46	39234.70	-19573.23	18504.62	18573.41	-9242.31	17245.03	17313.82	-8612.51	3191.52	3241.06	-1585.76
(6,2,4)	39815.89	39891.25	-19896.94	28251.79	28327.17	-14114.90	39185.69	39260.76	-19581.84	18432.50	18508.17	-9205.25	17112.48	17188.15	-8545.24	3190.39	3244.89	-1584.20
(6,2,5)	39805.46	39887.68	-19890.73	28141.62	28223.86	-14058.81	39166.29	39248.18	-19571.14	18395.74	18478.28	-9185.87	17386.56	17469.11	-8681.28	3182.12	3241.58	-1579.06
(6,2,6)	39806.91	39895.97	-19890.45	28127.40	28216.48	-14050.70	39144.02	39232.74	-19559.01	18394.87	18484.29	-9184.43	17143.29	17232.72	-8558.64	3186.60	3251.01	-1580.30
(6,2,7)	39785.21	39881.13	-19878.61	28146.87	28242.81	-14059.44	39163.81	39259.35	-19567.91	18411.18	18507.48	-9191.59	17125.18	17221.50	-8548.59	3187.02	3256.39	-1579.51
(6,2,8)	39781.88	39884.64	-19875.94	28138.79	28241.58	-14054.40	39137.95	39240.32	-19553.98	18403.37	18506.55	-9186.68	17118.35	17221.54	-8544.18	3182.54	3256.86	-1576.27
(6,2,9)	39793.36	39902.98	-19880.68	28154.87	28264.51	-14061.43	39145.62	39254.81	-19556.81	18412.88	18522.94	-9190.44	17127.53	17237.60	-8547.76	3202.07	3281.34	-1585.03
(6,2,10)	39762.42	39878.89	-19864.21	28138.86	28255.36	-14052.43	39107.54	39223.55	-19536.77	18399.87	18516.81	-9182.93	17125.23	17242.18	-8545.61	3199.15	3283.38	-1582.57
(7,1,0)	39796.78	39858.44	-19889.39	28157.13	28218.81	-14069.57	39158.66	39220.08	-19570.33	18472.12	18534.03	-9227.06	17151.22	17213.13	-8566.61	3178.85	3223.45	-1580.42
(7,1,1)	39798.82	39867.33	-19889.41	28124.35	28192.88	-14052.17	39144.46	39212.71	-19562.23	18326.85	18395.64	-9153.42	17098.42	17167.22	-8539.21	3174.32	3223.88	-1577.16
(7,1,2)	39793.91	39869.28	-19885.96	28127.26	28202.65	-14052.63	39150.77	39225.84	-19564.38	18325.52	18401.18	-9151.76	17099.60	17175.28	-8538.80	3176.21	3230.72	-1577.10
(7,1,3)	39799.77	39881.99	-19887.88	28122.09	28204.32	-14049.04	39150.35	39232.24	-19563.18	18333.09	18415.64	-9154.55	17101.03	17183.59	-8538.52	3174.30	3233.77	-1575.15
(7,1,4)	39793.02	39882.09	-19883.51	28120.46	28209.55	-14047.23	39144.01	39232.72	-19559.00	18323.96	18413.39	-9148.98	17100.27	17189.70	-8537.13	3175.70	3240.13	-1574.85
(7,1,5)	39781.83	39877.75	-19876.92	28130.57	28226.51	-14051.28	39146.59	39242.13	-19559.29	18326.80	18423.11	-9149.40	17105.60	17201.92	-8538.80	3178.07	3247.45	-1575.04
(7,1,6)	39775.11	39877.88	-19872.56	28128.61	28231.40	-14049.31	39131.91	39234.28	-19550.96	18327.32	18430.51	-9148.66	17101.50	17204.69	-8535.75	3175.74	3250.07	-1572.87
(7,1,7)	39779.61	39889.23	-19873.80	28138.07	28247.72	-14053.04	39135.61	39244.80	-19551.81	18329.98	18440.05	-9148.99	17101.19	17211.26	-8534.60	3180.72	3260.01	-1574.36
(7,1,8)	39775.67	39892.15	-19870.84	28116.59	28233.09	-14041.29	39128.31	39244.33	-19547.15	18330.19	18447.14	-9148.10	17104.59	17221.55	-8535.30	3176.89	3261.13	-1571.44
(7,1,9)	39761.67	39885.00	-19862.84	28121.11	28244.46	-14042.56	39105.21	39228.06	-19534.61	18329.35	18453.17	-9146.68	17104.74	17228.57	-8534.37	3176.50	3265.71	-1570.25
(7,1,10)	39761.54	39891.71	-19861.77	28128.34	28258.54	-14045.17	39106.44	39236.11	-19534.22	18329.95	18460.65	-9145.97	17106.89	17237.61	-8534.45	3178.90	3273.06	-1570.45
(7,2,0)	40789.31	40844.12	-20386.65	29366.11	29420.93	-14675.05	40164.86	40219.46	-20074.43	19753.36	19808.39	-9868.68	18503.44	18558.48	-9243.72	3371.69	3411.33	-1677.85

(7,2,1)	39801.60	39863.26	-19891.80	28163.55	28225.22	-14072.78	39163.42	39224.84	-19572.71	18480.01	18541.92	-9231.00	17159.80	17221.71	-8570.90	3184.63	3229.22	-1583.31
(7,2,2)	39790.64	39859.15	-19885.32	28168.88	28237.41	-14074.44	39149.23	39217.48	-19564.62	18502.93	18571.72	-9241.47	17173.58	17242.37	-8576.79	3191.65	3241.20	-1585.83
(7,2,3)	39784.83	39860.19	-19881.41	28178.34	28253.72	-14078.17	39132.49	39207.56	-19555.24	18506.10	18581.77	-9242.05	17188.79	17264.46	-8583.39	3192.55	3247.05	-1585.27
(7,2,4)	39781.55	39863.76	-19878.77	28212.51	28294.74	-14094.25	39160.16	39242.05	-19568.08	18506.30	18588.85	-9241.15	17115.82	17198.37	-8545.91	3190.58	3250.03	-1583.29
(7,2,5)	39793.66	39882.72	-19883.83	28133.99	28223.07	-14053.99	39154.01	39242.73	-19564.00	18375.62	18465.04	-9174.81	17277.20	17366.64	-8625.60	3192.75	3257.16	-1583.37
(7,2,6)	39796.61	39892.53	-19884.31	28133.29	28229.23	-14052.64	39161.42	39256.96	-19566.71	18404.29	18500.60	-9188.15	17315.77	17412.08	-8643.88	3184.24	3253.61	-1578.12
(7,2,7)	39783.75	39886.52	-19876.87	28143.44	28246.23	-14056.72	39148.71	39251.07	-19559.35	18434.40	18537.58	-9202.20	17141.28	17244.47	-8555.64	3181.01	3255.33	-1575.51
(7,2,8)	39779.91	39889.53	-19873.96	28139.38	28249.02	-14053.69	39133.73	39242.92	-19550.86	18454.39	18564.45	-9211.19	17124.75	17234.82	-8546.38	3185.81	3265.09	-1576.91
(7,2,9)	39788.71	39905.19	-19877.36	28151.72	28268.22	-14058.86	39143.05	39259.06	-19554.52	18414.78	18531.72	-9190.39	17125.04	17241.99	-8545.52	3193.34	3277.57	-1579.67
(7,2,10)	39767.19	39890.51	-19865.59	28140.48	28263.83	-14052.24	39111.34	39234.18	-19537.67	18409.64	18533.46	-9186.82	17124.66	17248.49	-8544.33	3198.85	3288.03	-1581.42
(8,1,0)	39798.47	39866.98	-19889.24	28151.28	28219.80	-14065.64	39160.24	39228.48	-19570.12	18432.23	18501.02	-9206.12	17150.79	17219.58	-8565.39	3176.81	3226.36	-1578.40
(8,1,1)	39800.53	39875.90	-19889.27	28125.14	28200.52	-14051.57	39145.38	39220.45	-19561.69	18324.30	18399.97	-9151.15	17100.42	17176.10	-8539.21	3178.79	3233.31	-1578.40
(8,1,2)	39789.80	39872.02	-19882.90	28128.13	28210.36	-14052.06	39148.40	39230.29	-19562.20	18326.27	18408.82	-9151.13	17099.81	17182.36	-8537.90	3178.31	3237.78	-1577.15
(8,1,3)	39772.94	39862.00	-19873.47	28125.04	28214.13	-14049.52	39130.81	39219.53	-19552.40	18329.20	18418.63	-9151.60	17097.90	17187.33	-8535.95	3175.60	3240.02	-1574.80
(8,1,4)	39791.45	39887.37	-19881.72	28126.83	28222.77	-14049.41	39147.99	39243.54	-19560.00	18326.41	18422.72	-9149.20	17100.01	17196.33	-8536.01	3177.93	3247.31	-1574.97
(8,1,5)	39789.34	39892.11	-19879.67	28136.02	28238.81	-14053.01	39147.96	39250.32	-19558.98	18328.06	18431.25	-9149.03	17102.41	17205.61	-8536.21	3179.83	3254.17	-1574.92
(8,1,6)	39773.41	39883.03	-19870.70	28129.39	28239.04	-14048.70	39132.49	39241.68	-19550.25	18330.15	18440.21	-9149.07	17101.28	17211.35	-8534.64	3181.76	3261.05	-1574.88
(8,1,7)	39775.92	39892.39	-19870.96	28124.46	28240.95	-14045.23	39133.44	39249.45	-19549.72	18331.73	18448.67	-9148.86	17102.71	17219.66	-8534.35	3174.90	3259.14	-1570.45
(8,1,8)	39779.21	39902.54	-19871.61	28133.82	28257.17	-14048.91	39130.64	39253.48	-19547.32	18331.58	18455.40	-9147.79	17106.10	17229.93	-8535.05	3179.55	3268.75	-1571.77
(8,1,9)	39761.88	39892.06	-19861.94	28132.94	28263.15	-14047.47	39107.32	39236.99	-19534.66	18331.23	18461.93	-9146.61	17107.76	17238.47	-8534.88	3182.37	3276.52	-1572.18
(8,1,10)	39762.17	39899.20	-19861.09	28127.75	28264.81	-14043.87	39108.37	39244.86	-19534.19	18332.36	18469.94	-9146.18	17108.88	17246.47	-8534.44	3180.64	3279.76	-1570.32
(8,2,0)	40683.79	40745.45	-20332.90	29265.31	29326.98	-14623.65	40050.91	40112.33	-20016.46	19680.85	19742.76	-9831.42	18375.07	18436.98	-9178.53	3340.81	3385.40	-1661.41
(8,2,1)	39803.30	39871.81	-19891.65	28157.76	28226.29	-14068.88	39164.98	39233.23	-19572.49	18440.28	18509.06	-9210.14	17159.41	17228.21	-8569.71	3182.72	3232.26	-1581.36
(8,2,2)	39790.45	39865.82	-19884.23	28167.52	28242.90	-14072.76	39150.13	39225.19	-19564.06	18478.76	18554.43	-9228.38	17163.71	17239.39	-8570.86	3187.95	3242.45	-1582.97
(8,2,3)	39786.25	39868.47	-19881.13	28157.98	28240.21	-14066.99	39149.04	39230.93	-19562.52	18507.71	18590.25	-9241.85	17168.77	17251.33	-8572.39	3190.04	3249.49	-1583.02
(8,2,4)	39798.56	39887.63	-19886.28	28184.80	28273.88	-14079.40	39156.10	39244.82	-19565.05	18509.76	18599.18	-9241.88	17123.49	17212.92	-8548.74	3195.89	3260.30	-1584.95
(8,2,5)	39793.37	39889.29	-19882.69	28132.38	28228.32	-14052.19	39156.88	39252.43	-19564.44	20177.36	20273.67	#######	17242.29	17338.60	-8607.14	3197.98	3267.35	-1584.99
(8,2,6)	39796.55	39899.32	-19883.28	28137.09	28239.88	-14053.55	39160.53	39262.90	-19565.27	18522.21	18625.40	-9246.11	17253.26	17356.45	-8611.63	3183.50	3257.82	-1576.75
(8,2,7)	39800.59	39910.21	-19884.30	28144.55	28254.19	-14056.27	39147.36	39256.55	-19557.68	18392.25	18502.32	-9180.13	17124.69	17234.76	-8546.34	3191.62	3270.90	-1579.81
(8,2,8)	39790.03	39906.51	-19878.02	28151.98	28268.48	-14058.99	39154.64	39270.65	-19560.32	18435.03	18551.97	-9200.51	17143.33	17260.28	-8554.66	3188.58	3272.81	-1577.29
(8,2,9)	39793.32	39916.65	-19878.66	28171.97	28295.32	-14067.99	39153.72	39276.56	-19558.86	18448.14	18571.96	-9206.07	17127.92	17251.75	-8545.96	3210.77	3299.96	-1587.39
(8,2,10)	39766.41	39896.58	-19864.20	28150.65	28280.85	-14056.32	39111.95	39241.61	-19536.98	18446.39	18577.09	-9204.19	17133.27	17263.98	-8547.63	3200.73	3294.87	-1581.37
(9,1,0)	39799.62	39874.98	-19888.81	28137.95	28213.33	-14057.98	39161.63	39236.70	-19569.81	18387.58	18463.25	-9182.79	17137.61	17213.29	-8557.81	3178.80	3233.31	-1578.40
(9,1,1)	39801.60	39883.81	-19888.80	28127.14	28209.37	-14051.57	39163.64	39245.54	-19569.82	18326.21	18408.75	-9151.10	17098.24	17180.80	-8537.12	3180.81	3240.27	-1578.40
(9,1,2)	39787.23	39876.30	-19880.61	28128.76	28217.84	-14051.38	39130.25	39218.97	-19552.12	18327.93	18417.36	-9150.97	17099.04	17188.48	-8536.52	3174.91	3239.34	-1574.46
(9,1,3)	39789.96	39885.88	-19880.98	28124.88	28220.82	-14048.44	39150.91	39246.45	-19561.46	18327.37	18423.68	-9149.69	17100.60	17196.92	-8536.30	3176.28	3245.66	-1574.14
(9,1,4)	39792.33	39895.10	-19881.17	28120.68	28223.47	-14045.34	39150.43	39252.80	-19560.21	18329.84	18433.03	-9149.92	17101.99	17205.18	-8536.00	3179.61	3253.94	-1574.81
(9,1,5)	39778.01	39887.63	-19873.00	28129.86	28239.51	-14048.93	39151.39	39260.58	-19559.69	18325.39	18435.45	-9146.69	17103.78	17213.85	-8535.89	3181.73	3261.02	-1574.86
(9,1,6)	39775.28	39891.75	-19870.64	28132.15	28248.65	-14049.08	39133.05	39249.06	-19549.52	18332.09	18449.03	-9149.04	17102.75	17219.71	-8534.38	3183.72	3267.96	-1574.86
(9,1,7)	39761.29	39884.61	-19862.64	28134.39	28257.74	-14049.20	39105.27	39228.11	-19534.63	18333.42	18457.25	-9148.71	17104.64	17228.47	-8534.32	3179.61	3268.81	-1571.81
(9,1,8)	39766.23	39896.41	-19864.12	28130.78	28260.98	-14046.39	39110.05	39239.72	-19536.03	18329.05	18459.75	-9145.52	17106.97	17237.68	-8534.48	3181.40	3275.55	-1571.70
(9,1,9)	39763.53	39900.56	-19861.76	28129.21	28266.26	-14044.60	39111.17	39247.66	-19535.58	18333.42	18471.00	-9146.71	17108.30	17245.89	-8534.15	3176.74	3275.86	-1568.37
(9,1,10)	39763.29	39907.17	-19860.65	28123.76	28267.67	-14040.88	39110.39	39253.70	-19534.19	18334.42	18478.88	-9146.21	17110.06	17254.53	-8534.03	3178.44	3282.51	-1568.22
(9,2,0)	40582.61	40651.13	-20281.31	29141.99	29210.52	-14561.00	39940.82	40009.06	-19960.41	19577.16	19645.95	-9778.58	18223.37	18292.16	-9101.68	3325.60	3375.15	-1652.80
(9,2,1)	39804.44	39879.80	-19891.22	28144.53	28219.91	-14061.26	39166.39	39241.46	-19572.19	18395.79	18471.46	-9186.89	17146.32	17222.00	-8562.16	3184.71	3239.21	-1581.35
(9,2,2)	39788.74	39870.96	-19882.37	28161.64	28243.88	-14068.82	39148.48	39230.37	-19562.24	18440.91	18523.45	-9208.45	17163.10	17245.65	-8569.55	3186.63	3246.08	-1581.31
(9,2,3)	39774.82	39863.89	-19874.41	28156.72	28245.80	-14065.36	39129.68	39218.39	-19551.84	18340.94	18430.37	-9157.47	17163.06	17252.50	-8568.53	3188.51	3252.92	-1581.25
(9,2,4)	39769.92	39865.84	-19870.96	28169.23	28265.17	-14070.61	39120.78	39216.32	-19546.39	18451.62	18547.93	-9211.81	17171.06	17267.37	-8571.53	3189.34	3258.70	-1580.67
(9,2,5)	39765.79	39868.56	-19867.90	28176.09	28278.88	-14073.04	39113.03	39215.39	-19541.51	18515.04	18618.22	-9242.52	17215.68	17318.87	-8592.84	3190.01	3264.33	-1580.00

(9,2,6)	39762.93	39872.55	-19865.46	28142.87	28252.52	-14055.44	39109.76	39218.95	-19538.88	18516.99	18627.05	-9242.49	17243.56	17353.63	-8605.78	3187.25	3266.52	-1577.62
(9,2,7)	39766.00	39882.48	-19866.00	28243.77	28360.27	-14104.89	39112.44	39228.46	-19539.22	18448.14	18565.08	-9207.07	17130.75	17247.70	-8548.38	3192.06	3276.29	-1579.03
(9,2,8)	39767.78	39891.10	-19865.89	28320.18	28443.52	-14142.09	39110.70	39233.54	-19537.35	18451.66	18575.48	-9207.83	17124.37	17248.20	-8544.18	3188.06	3277.24	-1576.03
(9,2,9)	39769.18	39899.36	-19865.59	28137.35	28267.55	-14049.68	39113.27	39242.93	-19537.64	18485.37	18616.07	-9223.68	17142.47	17273.18	-8552.24	3208.38	3302.51	-1585.19
(9,2,10)	39766.34	39903.36	-19863.17	28133.75	28270.80	-14046.87	39116.72	39253.20	-19538.36	18410.53	18548.11	-9185.26	17133.17	17270.76	-8546.59	3219.76	3318.85	-1589.88
(10,1,0)	39801.60	39883.82	-19888.80	28137.40	28219.63	-14056.70	39163.45	39245.35	-19569.73	18369.22	18451.77	-9172.61	17137.87	17220.42	-8556.93	3180.24	3239.71	-1578.12
(10,1,1)	39802.55	39891.62	-19888.27	28139.42	28228.50	-14056.71	39138.76	39227.48	-19556.38	18325.53	18414.95	-9149.76	17100.12	17189.55	-8537.06	3172.52	3236.94	-1573.26
(10,1,2)	39787.85	39883.77	-19879.93	28131.13	28227.06	-14051.56	39147.33	39242.87	-19559.66	18330.02	18426.33	-9151.01	17101.10	17197.42	-8536.55	3175.16	3244.54	-1573.58
(10,1,3)	39791.18	39893.96	-19880.59	28123.09	28225.88	-14046.55	39128.70	39231.07	-19549.35	18329.26	18432.45	-9149.63	17102.01	17205.20	-8536.00	3173.59	3247.92	-1571.79
(10,1,4)	39793.57	39903.19	-19880.78	28120.64	28230.28	-14044.32	39152.27	39261.47	-19560.14	18331.09	18441.15	-9149.54	17103.60	17213.67	-8535.80	3183.92	3263.21	-1575.96
(10,1,5)	39776.90	39893.38	-19871.45	28123.93	28240.43	-14044.97	39137.34	39253.35	-19551.67	18330.63	18447.57	-9148.31	17106.41	17223.36	-8536.20	3180.01	3264.25	-1573.00
(10,1,6)	39760.55	39883.88	-19862.28	28126.53	28249.88	-14045.27	39108.15	39230.99	-19536.08	18330.78	18454.60	-9147.39	17103.86	17227.69	-8533.93	3178.19	3267.39	-1571.10
(10,1,7)	39763.10	39893.28	-19862.55	28119.42	28249.62	-14040.71	39107.81	39237.48	-19534.91	18332.09	18462.79	-9147.04	17126.02	17256.74	-8544.01	3180.81	3274.97	-1571.40
(10,1,8)	39763.74	39900.77	-19861.87	28131.31	28268.37	-14045.65	39109.08	39245.57	-19534.54	18334.04	18471.62	-9147.02	17107.54	17245.13	-8533.77	3176.10	3275.21	-1568.05
(10,1,9)	39764.26	39908.14	-19861.13	28132.49	28276.40	-14045.24	39111.11	39254.42	-19534.55	18335.64	18480.10	-9146.82	17109.92	17254.40	-8533.96	3180.93	3285.00	-1569.47
(10,1,10)	39765.05	39915.78	-19860.52	28133.52	28284.28	-14044.76	39112.41	39262.55	-19534.21	18336.41	18487.75	-9146.21	17109.39	17260.74	-8532.70	3181.17	3290.19	-1568.59
(10,2,0)	40562.16	40637.52	-20270.08	29019.11	29094.49	-14498.55	39915.71	39990.77	-19946.85	19475.59	19551.25	-9726.79	18142.66	18218.34	-9060.33	3316.15	3370.65	-1647.08
(10,2,1)	39806.42	39888.64	-19891.21	28144.01	28226.24	-14060.01	39168.21	39250.10	-19572.10	18377.54	18460.08	-9176.77	17146.61	17229.16	-8561.30	3186.19	3245.65	-1581.10
(10,2,2)	39786.04	39875.10	-19880.02	28145.51	28234.60	-14059.76	39143.55	39232.27	-19558.78	18394.37	18483.79	-9184.18	17150.32	17239.76	-8562.16	3188.63	3253.04	-1581.32
(10,2,3)	39770.18	39866.09	-19871.09	28148.18	28244.12	-14060.09	39123.19	39218.73	-19547.60	18339.01	18435.31	-9155.50	17159.50	17255.82	-8565.75	3186.59	3255.95	-1579.29
(10,2,4)	39808.80	39911.57	-19889.40	28166.31	28269.10	-14068.16	39144.98	39247.35	-19557.49	18339.04	18442.23	-9154.52	17163.46	17266.65	-8566.73	3189.35	3263.67	-1579.67
(10,2,5)	39764.18	39873.80	-19866.09	28170.16	28279.80	-14069.08	39112.13	39221.33	-19540.07	18511.73	18621.79	-9239.87	17197.92	17307.99	-8582.96	3194.15	3273.43	-1581.08
(10,2,6)	39764.24	39880.71	-19865.12	28138.82	28255.31	-14052.41	39111.45	39227.46	-19538.72	18516.75	18633.69	-9241.37	17131.11	17248.06	-8548.55	3194.79	3279.02	-1580.39
(10,2,7)	39767.30	39890.62	-19865.65	28146.24	28269.59	-14055.12	39114.27	39237.11	-19539.13	18520.95	18644.77	-9242.48	17231.98	17355.81	-8597.99	3198.04	3287.22	-1581.02
(10,2,8)	39768.06	39898.23	-19865.03	28141.62	28271.82	-14051.81	39115.39	39245.05	-19538.70	18531.58	18662.28	-9246.79	17120.07	17250.78	-8541.03	3189.99	3284.13	-1576.00
(10,2,9)	39770.60	39907.63	-19865.30	28134.22	28271.28	-14047.11	39114.61	39251.10	-19537.31	18565.97	18703.55	-9262.99	17130.02	17267.61	-8545.01	3209.58	3308.68	-1584.79
(10,2,10)	39767.94	39911.81	-19862.97	28134.51	28278.42	-14046.26	39121.74	39265.06	-19539.87	18505.32	18649.77	-9231.66	17144.93	17289.40	-8551.47	3219.37	3323.42	-1588.69

Table S4 - Grid search results of one patient for the derived physiologic signals with respect to AIC, BIC and LL values

ARIMA Model	PRx_AIC	PRx_BIC	PRx_LL	PAx_AIC	PAx_BIC	PAx_LL	RAC_AIC	RAC_BIC	RAC_LL	RAP_AIC	RAP_BIC	RAP_LL	COx_L_AIC	COx_L_BIC	COx_L_LL	COx_R_AIC	COx_R_BIC	COx_R_LL	COx-a_L_AIC	COx-a_L_BIC	COx-a_L_LL	COx-a_R_AIC	COx-a_R_BIC	COx-a_R_LL
(1,1,0)	164.53	184.99	-79.27	-1873.88	-1853.42	939.94	-2380.59	-2360.13	1193.30	-2741.00	-2720.45	1373.50	-815.44	-794.99	410.72	-947.48	-927.02	476.74	-1518.78	-1498.24	762.39	-1601.87	-1581.33	803.94
(1,1,1)	105.04	132.32	-48.52	-2386.44	-2359.16	1197.22	-2829.95	-2802.67	1418.97	-3134.42	-3107.02	1571.21	-1433.18	-1405.91	720.59	-1589.00	-1561.73	798.50	-2214.34	-2186.96	1111.17	-2363.27	-2335.88	1185.63
(1,1,2)	-527.97	-493.87	268.98	-2566.32	-2532.22	1288.16	-3041.57	-3007.47	1525.78	-3330.29	-3296.04	1670.14	-1735.60	-1701.51	872.80	-1874.65	-1840.55	942.33	-2458.09	-2423.86	1234.05	-2641.61	-2607.37	1325.81
(1,1,3)	-535.01	-494.10	273.51	-2587.16	-2546.24	1299.58	-3059.00	-3018.09	1535.50	-3363.16	-3322.06	1687.58	-1753.99	-1713.08	883.00	-1888.80	-1847.89	950.40	-2484.30	-2443.21	1248.15	-2664.74	-2623.65	1338.37
(1,1,4)	-533.02	-485.28	273.51	-2585.37	-2537.63	1299.68	-3059.63	-3011.89	1536.81	-3369.12	-3321.17	1691.56	-1756.52	-1708.79	885.26	-1898.39	-1850.65	956.19	-2482.71	-2434.78	1248.35	-2663.71	-2615.77	1338.85
(1,1,5)	-531.80	-477.24	273.90	-2596.30	-2541.75	1306.15	-3076.55	-3022.00	1546.28	-3384.86	-3330.05	1700.43	-1755.20	-1700.65	885.60	-1895.70	-1841.14	955.85	-2489.75	-2434.98	1252.88	-2663.47	-2608.69	1339.74
(1,1,6)	-529.74	-468.36	273.87	-2592.61	-2531.24	1305.31	-3074.30	-3012.93	1546.15	-3382.57	-3320.91	1700.28	-1734.77	-1673.40	876.39	-1874.63	-1813.26	946.32	-2486.73	-2425.10	1252.36	-2663.65	-2602.03	1340.83
(1,1,7)	-518.62	-450.43	269.31	-2592.95	-2524.75	1306.47	-3072.74	-3004.55	1546.37	-3380.72	-3312.21	1700.36	-1752.93	-1684.74	886.47	-1884.47	-1816.27	952.23	-2485.21	-2416.74	1252.60	-2662.63	-2594.16	1341.32
(1,1,8)	-529.97	-454.95	275.98	-2597.27	-2522.25	1309.63	-3070.88	-2995.87	1546.44	-3378.89	-3303.54	1700.45	-1750.94	-1675.93	886.47	-1887.31	-1812.30	954.66	-2486.39	-2411.07	1254.19	-2665.01	-2589.68	1343.50
(1,1,9)	-527.72	-445.88	275.86	-2595.58	-2513.75	1309.79	-3068.93	-2987.09	1546.46	-3380.21	-3298.00	1702.11	-1749.34	-1667.51	886.67	-1888.22	-1806.38	956.11	-2485.30	-2403.14	1254.65	-2663.85	-2581.68	1343.93
(1,1,10)	-527.22	-438.56	276.61	-2594.69	-2506.04	1310.35	-3068.47	-2979.82	1547.24	-3382.33	-3293.28	1704.17	-1747.60	-1658.95	886.80	-1887.02	-1798.37	956.51	-2484.33	-2395.32	1255.17	-2661.91	-2572.90	1343.96
(1,2,0)	3312.33	3325.97	-1654.16	1312.05	1325.69	-654.02	756.46	770.10	-376.23	341.19	354.90	-168.60	2492.90	2506.54	-1244.45	2442.23	2455.87	-1219.12	1879.66	1893.36	-937.83	1813.79	1827.48	-904.89
(1,2,1)	174.16	194.61	-84.08	-1863.85	-1843.39	934.92	-2370.53	-2350.07	1188.26	-2730.79	-2710.24	1368.39	-805.62	-785.17	405.81	-937.62	-917.16	471.81	-1508.78	-1488.24	757.39	-1591.88	-1571.34	798.94
(1,2,2)	114.68	141.96	-53.34	-1858.01	-1830.73	933.01	-2816.03	-2788.76	1412.02	-2704.64	-2677.24	1356.32	-857.34	-830.06	432.67	-988.16	-960.88	498.08	-1498.48	-1471.09	753.24	-1576.57	-1549.18	792.29
(1,2,3)	-499.59	-465.50	254.80	-1864.14	-1830.05	937.07	-2373.05	-2338.96	1191.53	-2732.98	-2698.73	1371.49	-815.23	-781.14	412.62	-943.15	-909.05	476.57	-1508.88	-1474.65	759.44	-1595.28	-1561.04	802.64
(1,2,4)	-3.62	37.30	7.81	-2386.55	-2345.64	1199.28	-2983.32	-2942.41	1497.66	-2791.09	-2749.99	1401.54	-1699.71	-1658.80	855.85	-1394.87	-1353.96	703.44	-2192.05	-2150.97	1102.02	-2306.70	-2265.61	1159.35
(1,2,5)	-264.58	-216.85	139.29	-2347.53	-2299.80	1180.77	-2808.37	-2760.63	1411.18	-3041.03	-2993.07	1527.51	-1556.34	-1508.61	785.17	-1734.69	-1686.96	874.35	-2290.57	-2242.65	1152.29	-2448.13	-2400.20	1231.07
(1,2,6)	-419.44	-364.89	217.72	-2473.73	-2419.18	1244.87	-2946.88	-2892.32	1481.44	-3217.91	-3163.10	1616.95	-1665.98	-1611.43	840.99	-1815.85	-1761.30	915.93	-2401.85	-2347.07	1208.92	-2572.10	-2517.32	1294.05
(1,2,7)	-473.02	-411.64	245.51	-2581.15	-2519.78	1299.57	-3057.41	-2996.04	1537.71	-3355.74	-3294.09	1686.87	-1719.91	-1658.54	868.96	-1854.66	-1793.29	936.33	-2471.01	-2409.39	1244.51	-2646.52	-2584.89	1332.26

(1,2,8)	-497.05	-428.86	258.53	-2580.03	-2511.83	1300.01	-3058.89	-2990.70	1539.44	-3367.95	-3299.44	1693.97	-1727.73	-1659.54	873.86	-1864.63	-1796.43	942.31	-2471.83	-2403.36	1245.91	-2648.26	-2579.78	1334.13
(1,2,9)	-500.39	-425.37	261.19	-2583.16	-2508.15	1302.58	-3058.90	-2983.89	1540.45	-3366.29	-3290.93	1694.14	-1737.55	-1662.54	879.77	-1873.39	-1798.38	947.70	-2470.17	-2394.86	1246.09	-2647.74	-2572.42	1334.87
(1,2,10)	-514.89	-433.06	269.45	-2584.42	-2502.58	1304.21	-3055.83	-2974.00	1539.92	-3364.12	-3281.91	1694.06	-1735.55	-1653.72	879.77	-1873.59	-1791.76	948.80	-2470.92	-2388.76	1247.46	-2649.51	-2567.34	1336.75
(2,1,0)	-22.97	4.31	15.49	-1992.24	-1964.96	1000.12	-2501.38	-2474.10	1254.69	-2807.22	-2779.82	1407.61	-1071.26	-1043.99	539.63	-1239.80	-1212.52	623.90	-1718.63	-1691.24	863.32	-1833.86	-1806.47	920.93
(2,1,1)	-532.49	-498.39	271.25	-2586.67	-2552.57	1298.34	-3063.38	-3029.28	1536.69	-3360.75	-3326.49	1685.37	-1746.18	-1712.09	878.09	-1876.54	-1842.45	943.27	-2482.70	-2448.47	1246.35	-2662.26	-2628.02	1336.13
(2,1,2)	-534.35	-493.43	273.18	-2581.18	-2540.26	1296.59	-3004.25	-2963.33	1508.12	-3371.96	-3330.86	1691.98	-1748.07	-1707.16	880.04	-1882.12	-1841.20	947.06	-928.09	-887.01	470.05	-2624.78	-2583.70	1318.39
(2,1,3)	-525.06	-477.33	269.53	-2587.74	-2540.01	1300.87	-3054.89	-3007.16	1534.45	-3371.33	-3323.38	1692.66	-1758.76	-1711.03	886.38	-1896.08	-1848.34	955.04	-2485.83	-2437.91	1249.92	-2666.02	-2618.08	1340.01
(2,1,4)	-532.16	-477.60	274.08	-2587.33	-2532.78	1301.67	-3055.42	-3000.86	1535.71	-3366.19	-3311.38	1691.09	-1757.15	-1702.60	886.58	-1892.75	-1838.19	954.37	-2483.35	-2428.58	1249.68	-2664.83	-2610.05	1340.42
(2,1,5)	-531.20	-469.82	274.60	-2598.13	-2536.76	1308.07	-3074.59	-3013.21	1546.29	-3382.16	-3320.51	1700.08	-1754.91	-1693.54	886.46	-1895.15	-1833.78	956.58	-2487.60	-2425.98	1252.80	-2666.59	-2604.97	1342.30
(2,1,6)	-529.12	-460.93	274.56	-2595.22	-2527.03	1307.61	-3072.93	-3004.74	1546.47	-3381.02	-3312.52	1700.51	-1740.99	-1672.80	880.50	-1888.13	-1819.93	954.06	-2485.80	-2417.33	1252.90	-2664.19	-2595.71	1342.09
(2,1,7)	-525.55	-450.54	273.77	-2589.52	-2514.51	1305.76	-3070.68	-2995.67	1546.34	-3378.01	-3302.65	1700.00	-1750.03	-1675.02	886.01	-1882.28	-1807.26	952.14	-2482.71	-2407.40	1252.36	-2659.20	-2583.88	1340.60
(2,1,8)	-528.99	-447.16	276.50	-2595.66	-2513.83	1309.83	-3068.85	-2987.02	1546.43	-3376.71	-3294.50	1700.35	-1749.00	-1667.17	886.50	-1885.58	-1803.75	954.79	-2483.15	-2400.99	1253.57	-2661.17	-2579.00	1342.58
(2,1,9)	-527.07	-438.41	276.53	-2594.24	-2505.59	1310.12	-3066.93	-2978.28	1546.46	-3376.17	-3287.11	1701.08	-1748.74	-1660.10	887.37	-1885.28	-1796.62	955.64	-2480.89	-2391.88	1253.45	-2661.60	-2572.58	1343.80
(2,1,10)	-524.69	-429.21	276.34	-2593.50	-2498.03	1310.75	-3066.48	-2971.01	1547.24	-3380.86	-3284.95	1704.43	-1745.98	-1650.51	886.99	-1886.58	-1791.10	957.29	-2483.56	-2387.71	1255.78	-2662.20	-2566.34	1345.10
(2,2,0)	2617.50	2637.96	-1305.75	677.90	698.36	-335.95	108.71	129.17	-51.36	-231.37	-210.82	118.69	1779.39	1799.85	-886.69	1690.55	1711.00	-842.27	1191.07	1211.61	-592.53	1106.46	1127.00	-550.23
(2,2,1)	-13.02	14.26	10.51	-1981.94	-1954.67	994.97	-2491.05	-2463.77	1249.52	-2796.91	-2769.51	1402.45	-1061.05	-1033.78	534.53	-1229.52	-1202.24	618.76	-1708.29	-1680.91	858.15	-1823.50	-1796.11	915.75
(2,2,2)	-516.47	-482.37	263.24	-1861.92	-1827.83	935.96	-2367.51	-2333.41	1188.75	-2726.96	-2692.71	1368.48	-1721.97	-1687.87	865.98	-1152.44	-1118.34	581.22	-1504.85	-1470.62	757.43	-2635.55	-2601.31	1322.77
(2,2,3)	-483.42	-442.51	247.71	-1906.80	-1865.88	959.40	-2420.01	-2379.10	1216.01	-2844.89	-2803.79	1428.45	-1347.55	-1306.64	679.77	-1640.95	-1600.03	826.47	-1671.39	-1630.31	841.70	-2624.72	-2583.64	1318.36
(2,2,4)	125.57	173.30	-55.78	-1894.12	-1846.38	954.06	-2498.59	-2450.86	1256.30	-2736.85	-2688.89	1375.42	-1708.04	-1660.31	861.02	-1045.51	-997.77	529.75	-2374.82	-2326.89	1194.41	-2601.31	-2553.38	1307.66
(2,2,5)	-59.03	-4.47	37.51	-2568.72	-2514.17	1292.36	-2607.56	-2553.01	1311.78	-2803.63	-2748.83	1409.82	-1723.11	-1668.56	869.56	-1690.07	-1635.52	853.04	-2456.24	-2401.47	1236.12	-2613.37	-2558.59	1314.69

(2,2,6)	-264.51	-203.13	141.25	-2361.55	-2300.17	1189.77	-2809.79	-2748.41	1413.89	-3041.00	-2979.35	1529.50	-1556.60	-1495.23	787.30	-1748.98	-1687.61	883.49	-2284.81	-2223.19	1151.40	-2451.46	-2389.83	1234.73
(2,2,7)	-434.00	-365.81	227.00	-2503.28	-2435.09	1261.64	-2962.71	-2894.51	1491.35	-3331.03	-3262.52	1675.51	-1665.92	-1597.73	842.96	-1820.76	-1752.57	920.38	-2393.41	-2324.94	1206.70	-2578.60	-2510.12	1299.30
(2,2,8)	-478.51	-403.49	250.25	-2574.10	-2499.08	1298.05	-3054.25	-2979.24	1538.13	-3356.67	-3281.31	1689.33	-1714.42	-1639.41	868.21	-1853.17	-1778.16	937.59	-2467.83	-2392.52	1244.92	-2641.75	-2566.43	1331.88
(2,2,9)	-495.07	-413.23	259.53	-2573.98	-2492.15	1298.99	-3054.47	-2972.64	1539.23	-3363.84	-3281.64	1693.92	-1730.17	-1648.34	877.08	-1865.30	-1783.47	944.65	-2467.30	-2385.14	1245.65	-2647.01	-2564.84	1335.50
(2,2,10)	-503.95	-415.30	264.97	-2576.67	-2488.02	1301.33	-3055.06	-2966.41	1540.53	-3363.62	-3274.57	1694.81	-1730.96	-1642.32	878.48	-1872.88	-1784.23	949.44	-2466.86	-2377.85	1246.43	-2644.10	-2555.08	1335.05
(3,1,0)	-128.35	-94.25	69.18	-2127.96	-2093.87	1068.98	-2613.28	-2579.19	1311.64	-2922.48	-2888.23	1466.24	-1239.86	-1205.76	624.93	-1423.07	-1388.97	716.53	-1902.55	-1868.31	956.27	-2021.01	-1986.77	1015.50
(3,1,1)	-534.79	-493.87	273.39	-2588.15	-2547.23	1300.07	-3064.22	-3023.31	1538.11	-3372.91	-3331.81	1692.46	-1749.23	-1708.32	880.62	-1885.52	-1844.60	948.76	-2481.05	-2439.96	1246.52	-2660.63	-2619.55	1336.32
(3,1,2)	-528.72	-480.99	271.36	-2591.03	-2543.30	1302.52	-3063.16	-3015.43	1538.58	-3367.46	-3319.51	1690.73	-1743.78	-1696.05	878.89	-1873.65	-1825.91	943.82	-2482.65	-2434.73	1248.33	-2662.46	-2614.53	1338.23
(3,1,3)	-530.40	-475.84	273.20	-2579.90	-2525.35	1297.95	-3061.44	-3006.89	1538.72	-3369.13	-3314.33	1692.56	-1755.61	-1701.06	885.81	-1895.05	-1840.49	955.52	-2483.33	-2428.56	1249.67	-2659.10	-2604.32	1337.55
(3,1,4)	-530.10	-468.72	274.05	-2587.37	-2525.99	1302.68	-3062.06	-3000.69	1540.03	-3365.42	-3303.77	1691.71	-1752.40	-1691.03	885.20	-1890.07	-1828.70	954.04	-2481.28	-2419.66	1249.64	-2662.02	-2600.40	1340.01
(3,1,5)	-527.06	-458.86	273.53	-2598.74	-2530.55	1309.37	-3072.33	-3004.13	1546.16	-3380.01	-3311.50	1700.00	-1747.21	-1679.02	883.61	-1881.11	-1812.92	950.56	-2487.36	-2418.89	1253.68	-2668.57	-2600.10	1344.29
(3,1,6)	-525.03	-450.01	273.51	-2597.12	-2522.11	1309.56	-3069.80	-2994.79	1545.90	-3379.26	-3303.91	1700.63	-1751.22	-1676.21	886.61	-1890.44	-1815.42	956.22	-2486.93	-2411.62	1254.47	-2666.33	-2591.00	1344.16
(3,1,7)	-533.73	-451.90	278.87	-2592.12	-2510.28	1308.06	-3069.02	-2987.19	1546.51	-3377.94	-3295.74	1700.97	-1749.25	-1667.43	886.63	-1889.40	-1807.57	956.70	-2484.09	-2401.93	1254.04	-2659.82	-2577.65	1341.91
(3,1,8)	-526.73	-438.08	276.37	-2593.43	-2504.78	1309.71	-3067.03	-2978.38	1546.52	-3374.65	-3285.60	1700.33	-1745.56	-1656.91	885.78	-1888.58	-1799.92	957.29	-2479.55	-2390.54	1252.78	-2658.72	-2569.71	1342.36
(3,1,9)	-525.76	-430.29	276.88	-2593.44	-2497.97	1310.72	-3065.22	-2969.75	1546.61	-3372.81	-3276.90	1700.40	-1745.92	-1650.46	886.96	-1887.11	-1791.64	957.55	-2473.40	-2377.55	1250.70	-2658.37	-2562.51	1343.19
(3,1,10)	-522.78	-420.49	276.39	-2595.50	-2493.21	1312.75	-3065.93	-2963.64	1547.96	-3378.50	-3275.74	1704.25	-1743.98	-1641.69	886.99	-1886.57	-1784.28	958.29	-2480.03	-2377.32	1255.01	-2656.54	-2553.82	1343.27
(3,2,0)	2126.92	2154.19	-1059.46	166.35	193.63	-79.18	-332.95	-305.68	170.48	-626.94	-599.54	317.47	1134.08	1161.36	-563.04	982.15	1009.43	-487.07	576.95	604.34	-284.48	491.83	519.22	-241.92
(3,2,1)	-118.15	-84.05	64.07	-2117.38	-2083.28	1063.69	-2602.69	-2568.60	1306.35	-2911.91	-2877.65	1460.95	-1229.33	-1195.24	619.67	-1412.45	-1378.35	711.23	-1891.88	-1857.65	950.94	-2010.32	-1976.08	1010.16
(3,2,2)	-10.21	30.71	11.10	-1978.25	-1937.33	995.12	-2998.84	-2957.92	1505.42	-2794.65	-2753.55	1403.33	-1456.49	-1415.58	734.24	-1838.64	-1797.72	925.32	-1724.93	-1683.85	868.46	-1954.79	-1913.71	983.40
(3,2,3)	-515.89	-468.15	264.94	-1894.39	-1846.65	954.19	-2418.80	-2371.07	1216.40	-3159.27	-3111.31	1586.63	-1727.79	-1680.06	870.89	-1696.45	-1648.71	855.22	-2389.76	-2341.83	1201.88	-2641.21	-2593.28	1327.61

(3,2,4)	102.63	157.19	-43.32	-1904.21	-1849.65	960.10	-2415.96	-2361.40	1215.98	-2749.00	-2694.20	1382.50	-1655.53	-1600.98	835.77	-1199.62	-1145.07	607.81	-2228.68	-2173.91	1122.34	-1858.06	-1803.28	937.03
(3,2,5)	23.59	84.96	-2.79	-2313.21	-2251.84	1165.60	-2380.89	-2319.51	1199.44	-2741.15	-2679.50	1379.58	-1717.66	-1656.29	867.83	-1238.23	-1176.85	628.11	-2304.77	-2243.15	1161.39	-1612.65	-1551.03	815.33
(3,2,6)	-33.62	34.58	26.81	-2246.69	-2178.49	1133.34	-2537.75	-2469.56	1278.88	-2817.38	-2748.87	1418.69	-1257.38	-1189.20	638.69	-1855.49	-1787.30	937.75	-1861.11	-1792.64	940.55	-2612.47	-2543.99	1316.23
(3,2,7)	-501.94	-426.92	261.97	-2354.58	-2279.57	1188.29	-2819.15	-2744.14	1420.58	-3057.15	-2981.80	1539.58	-1542.65	-1467.65	782.33	-1728.25	-1653.24	875.12	-2288.50	-2213.19	1155.25	-2445.38	-2370.06	1233.69
(3,2,8)	-418.69	-336.86	221.35	-2473.03	-2391.20	1248.52	-2952.75	-2870.92	1488.37	-3214.86	-3132.65	1619.43	-1664.28	-1582.45	844.14	-1813.50	-1731.67	918.75	-2410.13	-2327.96	1217.06	-2563.36	-2481.19	1293.68
(3,2,9)	-470.58	-381.93	248.29	-2578.56	-2489.91	1302.28	-3052.52	-2963.87	1539.26	-3350.42	-3261.36	1688.21	-1713.53	-1624.89	869.77	-1847.04	-1758.39	936.52	-2462.05	-2373.04	1244.03	-2638.87	-2549.86	1332.44
(3,2,10)	-488.01	-392.54	258.00	-2562.49	-2467.02	1295.24	-3052.64	-2957.17	1540.32	-3360.13	-3264.23	1694.07	-1724.63	-1629.16	876.31	-1852.54	-1757.06	940.27	-2468.43	-2372.57	1248.21	-2616.37	-2520.51	1322.19
(4,1,0)	-199.05	-158.13	105.52	-2200.99	-2160.07	1106.49	-2707.72	-2666.80	1359.86	-3030.14	-2989.04	1521.07	-1294.33	-1253.42	653.17	-1469.64	-1428.72	740.82	-1979.87	-1938.78	995.93	-2106.06	-2064.98	1059.03
(4,1,1)	-533.56	-485.82	273.78	-2586.47	-2538.73	1300.23	-3062.69	-3014.96	1538.35	-3371.05	-3323.10	1692.53	-1752.19	-1704.46	883.10	-1893.58	-1845.84	953.79	-2480.94	-2433.01	1247.47	-2661.88	-2613.94	1337.94
(4,1,2)	-530.82	-476.26	273.41	-2588.29	-2533.73	1302.14	-2613.64	-2559.09	1314.82	-3368.98	-3314.18	1692.49	-1757.11	-1702.56	886.55	-1896.47	-1841.91	956.23	-2484.76	-2429.99	1250.38	-2664.52	-2609.74	1340.26
(4,1,3)	-527.78	-466.40	272.89	-2593.31	-2531.94	1305.66	-3063.18	-3001.81	1540.59	-3374.66	-3313.01	1696.33	-1747.87	-1686.50	882.93	-1889.01	-1827.63	953.50	-2488.64	-2427.01	1253.32	-2667.18	-2605.55	1342.59
(4,1,4)	-527.64	-459.45	273.82	-2594.56	-2526.37	1307.28	-3068.05	-2999.85	1544.02	-3365.90	-3297.39	1692.95	-1752.11	-1683.92	886.06	-1889.67	-1821.48	954.84	-2489.31	-2420.84	1254.65	-2660.96	-2592.48	1340.48
(4,1,5)	-525.27	-450.25	273.63	-2596.50	-2521.49	1309.25	-3070.52	-2995.51	1546.26	-3382.81	-3307.46	1702.41	-1751.81	-1676.81	886.91	-1891.00	-1815.99	956.50	-2484.02	-2408.71	1253.01	-2664.24	-2588.92	1343.12
(4,1,6)	-523.96	-442.12	273.98	-2593.73	-2511.90	1308.87	-3067.40	-2985.57	1545.70	-3379.63	-3297.42	1701.81	-1749.89	-1668.06	886.94	-1890.30	-1808.46	957.15	-2480.91	-2398.74	1252.45	-2661.78	-2579.61	1342.89
(4,1,7)	-522.01	-433.35	274.00	-2592.26	-2503.61	1309.13	-3067.05	-2978.40	1546.53	-3377.95	-3288.89	1701.97	-1748.23	-1659.58	887.11	-1891.85	-1803.19	958.92	-2482.48	-2393.47	1254.24	-2656.70	-2567.69	1341.35
(4,1,8)	-526.32	-430.85	277.16	-2594.31	-2498.84	1311.16	-3065.28	-2969.80	1546.64	-3374.85	-3278.94	1701.42	-1745.03	-1649.56	886.51	-1883.87	-1788.40	955.94	-2475.76	-2379.91	1251.88	-2646.22	-2550.35	1337.11
(4,1,9)	-520.47	-418.18	275.23	-2592.70	-2490.41	1311.35	-3063.09	-2960.80	1546.55	-3374.55	-3271.80	1702.28	-1744.19	-1641.91	887.09	-1887.49	-1785.20	958.74	-2475.60	-2372.90	1252.80	-2657.11	-2554.40	1343.55
(4,1,10)	-524.85	-415.74	278.43	-2593.94	-2484.83	1312.97	-3066.25	-2957.15	1549.13	-3376.71	-3267.11	1704.36	-1740.91	-1631.81	886.46	-1888.17	-1779.06	960.08	-2478.22	-2368.67	1255.11	-2654.52	-2544.96	1343.26
(4,2,0)	1756.54	1790.63	-873.27	-110.26	-76.16	60.13	-590.61	-556.51	300.30	-891.92	-857.66	450.96	807.28	841.38	-398.64	628.61	662.70	-309.30	260.96	295.19	-125.48	164.82	199.05	-77.41
(4,2,1)	-188.64	-147.72	100.32	-2190.19	-2149.28	1101.10	-2696.88	-2655.97	1354.44	-3019.32	-2978.22	1515.66	-1283.62	-1242.71	647.81	-1458.85	-1417.93	735.43	-1968.98	-1927.90	990.49	-2095.15	-2054.07	1053.58
(4,2,2)	-123.16	-75.42	68.58	-2116.06	-2068.32	1065.03	-2598.70	-2550.96	1306.35	-2909.10	-2861.15	1461.55	-1225.37	-1177.64	619.69	-1408.63	-1360.90	711.32	-1887.88	-1839.95	950.94	-2006.53	-1958.60	1010.26

(4,2,3)	-260.76	-206.21	138.38	-2006.06	-1951.50	1011.03	-2506.83	-2452.28	1261.42	-2838.43	-2783.63	1427.21	-1654.48	-1599.93	835.24	-1803.90	-1749.35	909.95	-2441.44	-2386.66	1228.72	-2124.94	-2070.16	1070.47
(4,2,4)	-477.27	-415.90	247.64	-2013.22	-1951.84	1015.61	-2377.51	-2316.14	1197.76	-2723.01	-2661.35	1370.50	-1707.51	-1646.15	862.76	-1857.56	-1796.18	937.78	-2458.37	-2396.75	1238.18	-2537.48	-2475.86	1277.74
(4,2,5)	-423.49	-355.30	221.75	-1894.87	-1826.68	957.44	-2408.60	-2340.41	1214.30	-2740.81	-2672.30	1380.40	-1087.23	-1019.04	553.61	-1759.97	-1691.78	889.99	-1848.42	-1779.96	934.21	-1634.31	-1565.84	827.16
(4,2,6)	54.90	129.91	-16.45	-1973.97	-1898.96	997.99	-3036.32	-2961.31	1529.16	-2740.08	-2664.73	1381.04	-1713.54	-1638.53	867.77	-1843.68	-1768.67	932.84	-1759.80	-1684.48	890.90	-2604.04	-2528.72	1313.02
(4,2,7)	-518.52	-436.69	271.26	-2511.77	-2429.93	1267.88	-3029.54	-2947.71	1526.77	-2893.32	-2811.12	1458.66	-1312.15	-1230.33	668.08	-1814.50	-1732.67	919.25	-2292.50	-2210.34	1158.25	-2227.82	-2145.66	1125.91
(4,2,8)	-291.98	-203.33	158.99	-2353.74	-2265.09	1189.87	-2846.56	-2757.91	1436.28	-3095.14	-3006.08	1560.57	-1575.70	-1487.05	800.85	-1772.23	-1683.58	899.11	-2307.76	-2218.75	1166.88	-2466.97	-2377.96	1246.49
(4,2,9)	-426.53	-331.06	227.27	-2516.53	-2421.06	1272.27	-2981.92	-2886.45	1504.96	-3303.84	-3207.94	1665.92	-1644.81	-1549.35	836.40	-1812.54	-1717.07	920.27	-2380.37	-2284.52	1204.19	-2559.86	-2464.00	1293.93
(4,2,10)	-475.81	-373.52	252.90	-2554.37	-2452.08	1292.18	-3047.78	-2945.49	1538.89	-3362.86	-3260.11	1696.43	-1723.43	-1621.15	876.71	-1839.59	-1737.30	934.79	-2465.77	-2363.07	1247.88	-2624.75	-2522.04	1327.37
(5,1,0)	-245.28	-197.54	129.64	-2322.07	-2274.33	1168.03	-2849.90	-2802.16	1431.95	-3162.41	-3114.46	1588.21	-1391.59	-1343.85	702.79	-1553.05	-1505.31	783.52	-2114.71	-2066.78	1064.35	-2243.58	-2195.64	1128.79
(5,1,1)	-531.72	-477.16	273.86	-2585.58	-2531.02	1300.79	-3060.70	-3006.15	1538.35	-3370.03	-3315.23	1693.02	-1753.70	-1699.15	884.85	-1895.41	-1840.86	955.71	-2481.75	-2426.97	1248.87	-2658.97	-2604.19	1337.49
(5,1,2)	-530.71	-469.34	274.36	-2588.46	-2527.09	1303.23	-3065.67	-3004.30	1541.84	-3368.00	-3306.34	1693.00	-1542.91	-1481.54	780.46	-1889.30	-1827.92	953.65	-2481.36	-2419.74	1249.68	-2663.39	-2601.76	1340.69
(5,1,3)	-530.92	-462.72	275.46	-2595.11	-2526.92	1307.56	-3068.01	-2999.82	1544.01	-3367.78	-3299.27	1693.89	-1746.96	-1678.77	883.48	-1893.48	-1825.28	956.74	-2488.78	-2420.31	1254.39	-2664.77	-2596.29	1342.38
(5,1,4)	-523.92	-448.90	272.96	-2586.51	-2511.49	1304.25	-3065.63	-2990.61	1543.81	-3371.94	-3296.58	1696.97	-1749.45	-1674.44	885.73	-1883.65	-1808.63	952.82	-2480.29	-2404.97	1251.14	-2659.35	-2584.02	1340.67
(5,1,5)	-523.49	-441.65	273.74	-2595.48	-2513.65	1309.74	-3069.91	-2988.07	1546.95	-3380.18	-3297.97	1702.09	-1749.71	-1667.88	886.85	-1889.98	-1808.14	956.99	-2481.72	-2399.55	1252.86	-2662.10	-2579.93	1343.05
(5,1,6)	-521.99	-433.33	273.99	-2590.34	-2501.69	1308.17	-3071.36	-2982.70	1548.68	-3379.82	-3290.76	1702.91	-1744.67	-1656.02	885.33	-1885.77	-1797.12	955.89	-2481.00	-2391.99	1253.50	-2658.36	-2569.34	1342.18
(5,1,7)	-530.60	-435.13	279.30	-2593.75	-2498.28	1310.87	-3069.98	-2974.51	1548.99	-3376.74	-3280.83	1702.37	-1743.70	-1648.24	885.85	-1884.79	-1789.31	956.39	-2483.20	-2387.34	1255.60	-2661.45	-2565.58	1344.72
(5,1,8)	-529.81	-427.52	279.91	-2593.58	-2491.29	1311.79	-3066.83	-2964.54	1548.42	-3373.41	-3270.65	1701.71	-1744.26	-1641.98	887.13	-1884.19	-1781.90	957.09	-2478.74	-2376.04	1254.37	-2652.72	-2550.00	1341.36
(5,1,9)	-521.98	-412.87	276.99	-2591.68	-2482.57	1311.84	-3067.96	-2958.85	1549.98	-3375.06	-3265.45	1703.53	-1742.68	-1633.58	887.34	-1883.15	-1774.03	957.57	-2475.92	-2366.37	1253.96	-2653.14	-2543.58	1342.57
(5,1,10)	-532.75	-416.82	283.38	-2586.54	-2470.61	1310.27	-3066.22	-2950.29	1550.11	-3375.98	-3259.53	1704.99	-1739.80	-1623.88	886.90	-1888.71	-1772.78	961.36	-2475.85	-2359.45	1254.93	-2651.39	-2534.98	1342.69
(5,2,0)	1492.97	1533.88	-740.48	-507.15	-466.23	259.57	-978.79	-937.88	495.40	-1272.56	-1231.46	642.28	492.26	533.17	-240.13	308.06	348.97	-148.03	-123.72	-82.64	67.86	-243.26	-202.17	127.63

(5,2,1)	-234.70	-186.97	124.35	-2311.01	-2263.27	1162.50	-2838.77	-2791.04	1426.39	-3151.31	-3103.36	1582.66	-1380.63	-1332.90	697.32	-1542.04	-1494.30	778.02	-2103.55	-2055.62	1058.77	-2232.38	-2184.45	1123.19
(5,2,2)	-184.72	-130.17	100.36	-2186.17	-2131.62	1101.09	-2698.96	-2644.41	1357.48	-3016.56	-2961.75	1516.28	-1302.15	-1247.60	659.08	-1457.64	-1403.08	736.82	-1979.38	-1924.61	997.69	-2091.15	-2036.37	1053.57
(5,2,3)	-121.35	-59.97	69.67	-2129.39	-2068.02	1073.70	-2620.74	-2559.37	1319.37	-2910.68	-2849.03	1464.34	-1225.22	-1163.85	621.61	-1417.47	-1356.10	717.74	-1902.12	-1840.50	960.06	-2007.80	-1946.17	1012.90
(5,2,4)	-513.92	-445.73	266.96	-2525.06	-2456.87	1272.53	-2539.22	-2471.03	1279.61	-2804.78	-2736.28	1412.39	-1662.95	-1594.77	841.48	-1423.57	-1355.38	721.79	-2248.87	-2180.41	1134.44	-2148.76	-2080.28	1084.38
(5,2,5)	-418.00	-342.99	220.00	-1913.87	-1838.86	967.93	-2408.47	-2333.46	1215.24	-2723.25	-2647.89	1372.62	-1669.50	-1594.50	845.75	-1806.90	-1731.89	914.45	-2406.76	-2331.45	1214.38	-1689.80	-1614.48	855.90
(5,2,6)	7.14	88.98	8.43	-1940.69	-1858.86	982.34	-2401.57	-2319.74	1212.78	-3288.81	-3206.60	1656.40	-1650.16	-1568.33	837.08	-1849.96	-1768.12	936.98	-1578.31	-1496.15	801.15	-2624.28	-2542.11	1324.14
(5,2,7)	-514.74	-426.09	270.37	-2535.16	-2446.51	1280.58	-2593.56	-2504.91	1309.78	-2756.86	-2667.80	1391.43	-1422.88	-1334.23	724.44	-1854.63	-1765.97	940.31	-2397.12	-2308.11	1211.56	-2598.07	-2509.06	1312.04
(5,2,8)	-504.16	-408.69	266.08	-2556.70	-2461.23	1292.35	-3027.91	-2932.45	1527.96	-3326.50	-3230.59	1677.25	-1694.00	-1598.54	861.00	-1568.52	-1473.05	798.26	-2434.62	-2338.77	1231.31	-2640.08	-2544.22	1334.04
(5,2,9)	-309.90	-207.61	169.95	-2526.45	-2424.16	1278.23	-2865.95	-2763.67	1447.98	-3345.54	-3242.78	1687.77	-1730.14	-1627.85	880.07	-1870.90	-1768.60	950.45	-2278.86	-2176.16	1154.43	-2636.72	-2534.01	1333.36
(5,2,10)	-428.17	-319.06	230.09	-2498.33	-2389.22	1265.17	-2973.22	-2864.12	1502.61	-3287.95	-3178.34	1659.97	-1729.08	-1619.98	880.54	-1808.83	-1699.72	920.42	-2462.98	-2353.43	1247.49	-2583.05	-2473.49	1307.52
(6,1,0)	-287.70	-233.14	151.85	-2340.67	-2286.12	1178.34	-2877.56	-2823.01	1446.78	-3189.95	-3135.14	1602.97	-1442.68	-1388.13	729.34	-1597.74	-1543.18	806.87	-2157.67	-2102.89	1086.83	-2281.75	-2226.97	1148.88
(6,1,1)	-529.81	-468.44	273.91	-2592.26	-2530.89	1305.13	-3077.41	-3016.03	1547.70	-3386.65	-3324.99	1702.32	-1754.23	-1692.86	886.11	-1894.78	-1833.40	956.39	-2486.55	-2424.93	1252.27	-2668.32	-2606.70	1343.16
(6,1,2)	-529.22	-461.03	274.61	-2593.81	-2525.62	1306.90	-3071.31	-3003.11	1545.65	-3378.34	-3309.84	1699.17	-1753.31	-1685.12	886.66	-1893.83	-1825.64	956.92	-2480.84	-2412.37	1250.42	-2661.37	-2592.90	1340.69
(6,1,3)	-527.87	-452.85	274.93	-2596.47	-2521.45	1309.23	-3068.76	-2993.75	1545.38	-2917.18	-2841.83	1469.59	-1748.35	-1673.34	885.17	-1887.51	-1812.50	954.76	-2483.42	-2408.11	1252.71	-2666.20	-2590.87	1344.10
(6,1,4)	-524.05	-442.22	274.03	-2591.33	-2509.49	1307.66	-3066.60	-2984.77	1545.30	-3369.89	-3287.68	1696.94	-1748.57	-1666.74	886.29	-1893.37	-1811.53	958.68	-2481.99	-2399.83	1253.00	-2662.55	-2580.38	1343.28
(6,1,5)	-520.08	-431.43	273.04	-2594.52	-2505.87	1310.26	-3071.51	-2982.86	1548.76	-3378.94	-3289.88	1702.47	-1746.35	-1657.70	886.17	-1885.49	-1796.84	955.75	-2484.42	-2395.41	1255.21	-2661.59	-2572.58	1343.80
(6,1,6)	-522.30	-426.83	275.15	-2591.34	-2495.87	1309.67	-3065.96	-2970.49	1546.98	-3380.63	-3284.72	1704.31	-1744.30	-1648.83	886.15	-1884.31	-1788.84	956.16	-2481.76	-2385.91	1254.88	-2655.81	-2559.95	1341.91
(6,1,7)	-519.24	-416.95	274.62	-2593.26	-2490.97	1311.63	-3071.14	-2968.85	1550.57	-3375.66	-3272.90	1702.83	-1743.71	-1641.43	886.86	-1884.57	-1782.28	957.28	-2479.09	-2376.39	1254.55	-2660.05	-2557.34	1345.03
(6,1,8)	-528.35	-419.24	280.18	-2591.68	-2482.57	1311.84	-3064.79	-2955.68	1548.40	-3376.22	-3266.61	1704.11	-1745.98	-1636.88	888.99	-1790.02	-1680.91	911.01	-2476.07	-2366.52	1254.03	-2656.45	-2546.89	1344.22
(6,1,9)	-521.24	-405.31	277.62	-2589.03	-2473.10	1311.51	-3065.14	-2949.21	1549.57	-3372.27	-3255.82	1703.14	-1741.73	-1625.81	887.87	-1880.74	-1764.81	957.37	-2476.71	-2360.31	1255.35	-2655.30	-2538.90	1344.65

(6,1,10)	-531.18	-408.43	283.59	-2587.52	-2464.77	1311.76	-3063.39	-2940.64	1549.70	-3372.11	-3248.80	1704.06	-1738.92	-1616.18	887.46	-1886.69	-1763.94	961.35	-2474.12	-2350.88	1255.06	-2653.85	-2530.59	1344.92
(6,2,0)	1275.83	1323.56	-630.91	-701.35	-653.62	357.68	-1221.39	-1173.65	617.69	-1561.08	-1513.13	787.54	287.71	335.44	-136.85	95.54	143.27	-40.77	-368.34	-320.41	191.17	-483.97	-436.04	248.98
(6,2,1)	-276.96	-222.40	146.48	-2329.50	-2274.95	1172.75	-2866.31	-2811.75	1441.15	-3178.72	-3123.91	1597.36	-1431.55	-1377.00	723.77	-1586.56	-1532.01	801.28	-2146.34	-2091.57	1081.17	-2270.40	-2215.63	1143.20
(6,2,2)	-231.26	-169.89	124.63	-2312.15	-2250.78	1165.07	-2834.89	-2773.51	1426.44	-3149.51	-3087.86	1583.75	-1376.78	-1315.41	697.39	-1538.06	-1476.69	778.03	-2099.83	-2038.21	1058.91	-2228.38	-2166.76	1123.19
(6,2,3)	-200.59	-132.39	110.29	-2205.16	-2136.96	1112.58	-3027.20	-2959.01	1523.60	-3286.92	-3218.41	1653.46	-1276.93	-1208.74	648.47	-1465.42	-1397.23	742.71	-2419.88	-2351.41	1219.94	-2272.93	-2204.46	1146.47
(6,2,4)	-131.55	-56.54	76.77	-2303.50	-2228.49	1162.75	-2647.77	-2572.76	1334.88	-3350.73	-3275.38	1686.37	-1224.29	-1149.28	623.14	-1407.24	-1332.23	714.62	-1918.22	-1842.91	970.11	-2230.05	-2154.73	1126.02
(6,2,5)	-234.40	-152.57	129.20	-2432.45	-2350.62	1228.22	-2623.92	-2542.09	1323.96	-2839.48	-2757.28	1431.74	-1118.77	-1036.95	571.39	-1292.31	-1210.48	658.16	-2071.54	-1989.37	1047.77	-1895.71	-1813.55	959.86
(6,2,6)	-319.88	-231.23	172.94	-2545.41	-2456.76	1285.71	-3044.32	-2955.67	1535.16	-3341.54	-3252.49	1683.77	-1704.97	-1616.32	865.48	-1837.66	-1749.01	931.83	-2343.36	-2254.35	1184.68	-2613.06	-2524.04	1319.53
(6,2,7)	-512.49	-417.02	270.25	-2398.78	-2303.32	1213.39	-2446.21	-2350.74	1237.11	-3289.60	-3193.70	1658.80	-1730.49	-1635.03	879.25	-1794.23	-1698.76	911.11	-2442.21	-2346.35	1235.10	-2561.72	-2465.86	1294.86
(6,2,8)	-500.28	-397.99	265.14	-2567.30	-2465.01	1298.65	-3048.70	-2946.42	1539.35	-3311.40	-3208.64	1670.70	-1658.49	-1556.21	844.25	-1866.50	-1764.21	948.25	-2444.53	-2341.83	1237.27	-2638.31	-2535.60	1334.16
(6,2,9)	-296.67	-187.56	164.33	-2549.20	-2440.09	1290.60	-3035.75	-2926.64	1533.88	-3351.74	-3242.13	1691.87	-1340.61	-1231.51	686.30	-1866.91	-1757.80	949.45	-1896.82	-1787.27	964.41	-2645.42	-2535.86	1338.71
(6,2,10)	-317.46	-201.53	175.73	-2417.02	-2301.09	1225.51	-3014.75	-2898.82	1524.37	-3258.69	-3142.23	1646.34	-1729.91	-1613.99	881.96	-1686.89	-1570.96	860.44	-2461.56	-2345.16	1247.78	-2416.62	-2300.22	1225.31
(7,1,0)	-318.17	-256.80	168.09	-2393.52	-2332.14	1205.76	-2913.50	-2852.13	1465.75	-3205.89	-3144.24	1611.95	-1509.65	-1448.28	763.82	-1652.59	-1591.22	835.30	-2212.27	-2150.65	1115.14	-2339.56	-2277.93	1178.78
(7,1,1)	-527.82	-459.63	273.91	-2597.06	-2528.86	1308.53	-3075.86	-3007.67	1547.93	-3384.83	-3316.32	1702.41	-1752.23	-1684.04	886.12	-1892.85	-1824.66	956.43	-2485.47	-2417.00	1252.73	-2664.26	-2595.79	1342.13
(7,1,2)	-527.59	-452.58	274.80	-2591.37	-2516.36	1306.69	-3074.19	-2999.17	1548.09	-3383.09	-3307.73	1702.54	-1751.56	-1676.56	886.78	-1891.87	-1816.86	956.94	-2482.55	-2407.23	1252.27	-2664.52	-2589.20	1343.26
(7,1,3)	-524.46	-442.62	274.23	-2594.01	-2512.18	1309.01	-3066.68	-2984.85	1545.34	-3375.95	-3293.75	1699.98	-1232.01	-1150.18	628.00	-1891.98	-1810.15	957.99	-2484.95	-2402.78	1254.47	-2657.70	-2575.53	1340.85
(7,1,4)	-522.72	-434.07	274.36	-2590.00	-2501.35	1308.00	-3059.36	-2970.70	1542.68	-3380.40	-3291.34	1703.20	-1747.72	-1659.07	886.86	-1888.90	-1800.25	957.45	-2477.92	-2388.91	1251.96	-2662.22	-2573.20	1344.11
(7,1,5)	-522.69	-427.22	275.34	-2593.97	-2498.50	1310.99	-3059.97	-2964.50	1543.99	-3377.56	-3281.66	1702.78	-1744.27	-1648.80	886.13	-1884.14	-1788.67	956.07	-2482.45	-2386.59	1255.22	-2660.20	-2564.34	1344.10
(7,1,6)	-519.79	-417.49	274.89	-2590.91	-2488.61	1310.45	-3063.78	-2961.49	1546.89	-3376.76	-3274.00	1703.38	-1731.55	-1629.27	880.77	-1869.63	-1767.33	949.81	-2480.81	-2378.10	1255.40	-2657.19	-2554.48	1343.60
(7,1,7)	-518.50	-409.39	275.25	-2592.14	-2483.03	1312.07	-3065.97	-2956.86	1548.99	-3377.05	-3267.44	1704.52	-1741.76	-1632.66	886.88	-1881.92	-1772.81	956.96	-2477.53	-2367.98	1254.76	-2657.73	-2548.17	1344.86

(7,1,8)	-523.76	-407.82	278.88	-2587.07	-2471.14	1310.53	-3066.07	-2950.14	1550.03	-3375.28	-3258.82	1704.64	-1744.89	-1628.97	889.45	-1875.07	-1759.14	954.53	-2475.12	-2358.72	1254.56	-2655.37	-2538.96	1344.68
(7,1,9)	-511.56	-388.80	273.78	-2586.30	-2463.56	1311.15	-3064.23	-2941.48	1550.11	-3372.53	-3249.22	1704.26	-1740.63	-1617.89	888.31	-1879.24	-1756.49	957.62	-2473.72	-2350.48	1254.86	-2653.67	-2530.42	1344.84
(7,1,10)	-530.19	-400.62	284.09	-2583.15	-2453.58	1310.57	-3063.20	-2933.63	1550.60	-3373.84	-3243.68	1705.92	-1736.76	-1607.20	887.38	-1878.18	-1748.61	958.09	-2472.13	-2342.04	1255.07	-2654.46	-2524.36	1346.23
(7,2,0)	1147.78	1202.33	-565.89	-862.27	-807.72	439.14	-1442.52	-1387.97	729.26	-1760.21	-1705.41	888.11	57.29	111.84	-20.65	-116.03	-61.47	66.01	-562.87	-508.10	289.43	-679.11	-624.33	347.55
(7,2,1)	-307.29	-245.92	162.65	-2382.16	-2320.79	1200.08	-2902.09	-2840.72	1460.05	-3194.55	-3132.90	1606.27	-1493.59	-1432.23	755.80	-1641.23	-1579.86	829.62	-2200.77	-2139.15	1109.38	-2328.02	-2266.40	1173.01
(7,2,2)	-273.43	-205.24	146.72	-2327.14	-2258.95	1173.57	-2862.99	-2794.79	1441.49	-3174.88	-3106.37	1597.44	-1430.39	-1362.20	725.19	-1583.79	-1515.60	801.89	-2144.13	-2075.66	1082.07	-2268.17	-2199.70	1144.09
(7,2,3)	-237.58	-162.57	129.79	-2307.16	-2232.14	1164.58	-2836.22	-2761.21	1429.11	-3148.86	-3073.51	1585.43	-1374.01	-1299.01	698.01	-1552.85	-1477.84	787.43	-2100.78	-2025.46	1061.39	-2225.32	-2150.00	1123.66
(7,2,4)	-188.49	-106.66	106.25	-2326.75	-2244.92	1175.38	-2989.72	-2907.89	1506.86	-3158.81	-3076.61	1591.41	-1353.89	-1272.07	688.95	-1664.53	-1582.70	844.26	-2275.61	-2193.45	1149.81	-2495.94	-2413.77	1259.97
(7,2,5)	-115.60	-26.95	70.80	-2174.38	-2085.73	1100.19	-2963.82	-2875.17	1494.91	-3061.64	-2972.59	1543.82	-1229.33	-1140.69	627.67	-1414.02	-1325.37	720.01	-1988.63	-1899.63	1007.32	-2029.37	-1940.36	1027.69
(7,2,6)	-18.64	76.83	23.32	-2509.48	-2414.02	1268.74	-2496.71	-2401.24	1262.35	-3180.04	-3084.13	1604.02	-1117.23	-1021.77	572.62	-1310.13	-1214.66	669.06	-1770.96	-1675.10	899.48	-2085.88	-1990.02	1056.94
(7,2,7)	-508.99	-406.70	269.49	-2551.76	-2449.47	1290.88	-2709.03	-2606.74	1369.52	-3343.70	-3240.94	1686.85	-1726.98	-1624.70	878.49	-1144.71	-1042.42	587.36	-2454.06	-2351.36	1242.03	-2560.27	-2457.56	1295.13
(7,2,8)	-401.25	-292.14	216.62	-2567.03	-2457.92	1299.51	-3003.68	-2894.57	1517.84	-3342.60	-3232.99	1687.30	-1371.96	-1262.86	701.98	-1844.63	-1735.52	938.32	-2449.38	-2339.83	1240.69	-2642.58	-2533.03	1337.29
(7,2,9)	-477.72	-361.79	255.86	-2562.41	-2446.48	1298.20	-3033.34	-2917.41	1533.67	-3348.39	-3231.93	1691.19	-1699.18	-1583.26	866.59	-1872.40	-1756.47	953.20	-1945.36	-1828.96	989.68	-2639.93	-2523.52	1336.96
(7,2,10)	-497.35	-374.60	266.68	-2565.20	-2442.45	1300.60	-3046.00	-2923.25	1541.00	-3315.97	-3192.66	1675.98	-1280.88	-1158.14	658.44	-1814.32	-1691.58	925.16	-2177.98	-2054.73	1106.99	-2623.73	-2500.48	1329.87
(8,1,0)	-373.14	-304.95	196.57	-2442.07	-2373.87	1231.03	-2930.57	-2862.37	1475.28	-3223.72	-3155.21	1621.86	-1536.34	-1468.16	778.17	-1680.85	-1612.65	850.42	-2262.34	-2193.87	1141.17	-2391.32	-2322.84	1205.66
(8,1,1)	-525.91	-450.89	273.95	-2595.54	-2520.53	1308.77	-3074.23	-2999.22	1548.12	-3384.71	-3309.36	1703.36	-1754.28	-1679.27	888.14	-1892.37	-1817.36	957.19	-2480.01	-2404.70	1251.01	-2654.31	-2578.99	1338.16
(8,1,2)	-526.59	-444.76	275.30	-2593.82	-2511.98	1308.91	-3072.19	-2990.36	1548.09	-3382.25	-3300.04	1703.13	-1750.49	-1668.66	887.24	-1890.16	-1808.32	957.08	-2482.42	-2400.25	1253.21	-2663.85	-2581.68	1343.92
(8,1,3)	-522.58	-433.93	274.29	-2592.64	-2503.99	1309.32	-3070.61	-2981.95	1548.30	-3378.87	-3289.81	1702.43	-1746.82	-1658.18	886.41	-1888.75	-1800.09	957.37	-2483.12	-2394.11	1254.56	-2664.76	-2575.74	1345.38
(8,1,4)	-521.26	-425.79	274.63	-2587.70	-2492.23	1307.85	-3066.24	-2970.77	1547.12	-3374.65	-3278.74	1701.33	-1746.45	-1650.99	887.23	-1888.92	-1793.45	958.46	-2476.49	-2380.63	1252.25	-2659.46	-2563.60	1343.73
(8,1,5)	-523.35	-421.05	276.67	-2592.64	-2490.35	1311.32	-3065.51	-2963.22	1547.75	-3378.02	-3275.27	1704.01	-1744.52	-1642.23	887.26	-1883.23	-1780.94	956.62	-2480.87	-2378.17	1255.44	-2658.26	-2555.55	1344.13

(8,1,6)	-520.56	-411.45	276.28	-2587.85	-2478.74	1309.92	-3059.83	-2950.72	1545.92	-3376.48	-3266.87	1704.24	-1741.86	-1632.76	886.93	-1882.16	-1773.05	957.08	-2480.06	-2370.51	1256.03	-2657.78	-2548.22	1344.89
(8,1,7)	-519.92	-403.99	276.96	-2590.25	-2474.32	1312.12	-3064.64	-2948.71	1549.32	-3376.42	-3259.97	1705.21	-1740.26	-1624.33	887.13	-1876.93	-1761.00	955.46	-2472.86	-2356.47	1253.43	-2652.41	-2536.00	1343.20
(8,1,8)	-518.46	-395.71	277.23	-2585.68	-2462.94	1310.84	-3063.97	-2941.22	1549.98	-3376.66	-3253.35	1706.33	-1738.20	-1615.46	887.10	-1875.87	-1753.12	955.93	-2473.52	-2350.27	1254.76	-2654.26	-2531.01	1345.13
(8,1,9)	-511.19	-381.62	274.59	-2587.64	-2458.07	1312.82	-3062.65	-2933.08	1550.32	-3372.23	-3242.07	1705.12	-1738.10	-1608.54	888.05	-1884.10	-1754.52	961.05	-2472.15	-2342.06	1255.07	-2651.97	-2521.87	1344.99
(8,1,10)	-527.71	-391.32	283.86	-2581.20	-2444.81	1310.60	-3061.07	-2924.68	1550.53	-3372.90	-3235.89	1706.45	-1734.56	-1598.18	887.28	-1800.40	-1664.01	920.20	-2470.30	-2333.36	1255.15	-2650.11	-2513.16	1345.06
(8,2,0)	967.07	1028.45	-474.54	-1035.64	-974.26	526.82	-1603.81	-1542.44	810.91	-1905.85	-1844.20	961.93	-104.72	-43.35	61.36	-293.09	-231.71	155.54	-777.30	-715.68	397.65	-892.21	-830.59	455.11
(8,2,1)	-362.08	-293.88	191.04	-2427.46	-2359.26	1223.73	-2919.05	-2850.86	1469.53	-3212.28	-3143.77	1616.14	-541.02	-472.83	280.51	-1669.35	-1601.16	844.67	-2250.66	-2182.19	1135.33	-2379.61	-2311.13	1199.80
(8,2,2)	-303.47	-228.46	162.73	-2389.08	-2314.06	1205.54	-2897.78	-2822.77	1459.89	-3191.33	-3115.97	1606.66	-1494.39	-1419.39	758.20	-1637.30	-1562.29	829.65	-2197.17	-2121.85	1109.58	-2324.18	-2248.86	1173.09
(8,2,3)	-280.61	-198.78	152.31	-2337.23	-2255.40	1180.61	-2867.02	-2785.19	1445.51	-3173.89	-3091.68	1598.94	-1434.49	-1352.67	729.25	-1594.21	-1512.38	809.11	-2152.89	-2070.73	1088.45	-2270.65	-2188.48	1147.32
(8,2,4)	-234.99	-146.33	130.49	-2565.76	-2477.11	1295.88	-2956.44	-2867.79	1491.22	-3142.61	-3053.56	1584.31	-1389.19	-1300.54	707.59	-1541.08	-1452.43	783.54	-2093.99	-2004.98	1059.99	-2223.39	-2134.37	1124.69
(8,2,5)	-438.73	-343.26	233.37	-2295.89	-2200.42	1161.94	-2977.38	-2881.92	1502.69	-3067.55	-2971.65	1547.78	-1304.13	-1208.66	666.06	-1470.57	-1375.10	749.29	-2100.41	-2004.56	1064.21	-2127.60	-2031.74	1077.80
(8,2,6)	-511.47	-409.18	270.74	-2318.70	-2216.41	1174.35	-2621.77	-2519.48	1325.89	-3339.56	-3236.81	1684.78	-1221.35	-1119.07	625.67	-1416.55	-1314.26	723.27	-1895.81	-1793.10	962.90	-2519.46	-2416.75	1274.73
(8,2,7)	-509.67	-400.56	270.83	-2387.13	-2278.02	1209.56	-3045.37	-2936.26	1538.69	-3344.21	-3234.61	1688.11	-1724.84	-1615.74	878.42	-1265.40	-1156.29	648.70	-2459.88	-2350.33	1245.94	-2616.57	-2507.02	1324.29
(8,2,8)	-484.03	-368.10	259.01	-2574.59	-2458.66	1304.29	-3026.29	-2910.37	1530.15	-3343.90	-3227.45	1688.95	-1600.16	-1484.24	817.08	-1832.07	-1716.14	933.04	-2437.19	-2320.80	1235.60	-2638.39	-2521.98	1336.19
(8,2,9)	109.78	232.53	-36.89	-2507.25	-2384.50	1271.62	-3020.11	-2897.36	1528.05	-3342.70	-3219.40	1689.35	-1634.34	-1511.61	835.17	-1870.76	-1748.01	953.38	-1553.91	-1430.66	794.95	-2638.76	-2515.51	1337.38
(8,2,10)	-511.82	-382.25	274.91	-2567.31	-2437.75	1302.66	-3040.56	-2911.00	1539.28	-3335.82	-3205.66	1686.91	-1636.38	-1506.83	837.19	-1860.84	-1731.27	949.42	-2459.00	-2328.91	1248.50	-2617.92	-2487.82	1327.96
(9,1,0)	-386.93	-311.91	204.46	-2465.40	-2390.38	1243.70	-2948.13	-2873.12	1485.07	-3243.62	-3168.26	1632.81	-1567.74	-1492.73	794.87	-1700.64	-1625.62	861.32	-2280.33	-2205.01	1151.17	-2411.29	-2335.97	1216.65
(9,1,1)	-529.78	-447.95	276.89	-2595.63	-2513.80	1309.81	-3072.67	-2990.84	1548.34	-3383.76	-3301.56	1703.88	-1752.31	-1670.48	888.15	-1890.37	-1808.54	957.19	-2482.39	-2400.23	1253.19	-2666.01	-2583.84	1345.01
(9,1,2)	-526.14	-437.49	276.07	-2595.04	-2506.38	1310.52	-3070.28	-2981.63	1548.14	-3380.79	-3291.74	1703.40	-1750.38	-1661.73	888.19	-1888.58	-1799.93	957.29	-2481.83	-2392.82	1253.91	-2663.65	-2574.63	1344.82
(9,1,3)	-527.43	-431.95	277.71	-2590.41	-2494.94	1309.20	-3069.49	-2974.02	1548.74	-3376.93	-3281.03	1702.47	-1747.07	-1651.60	887.53	-1885.01	-1789.54	956.51	-2482.43	-2386.57	1255.21	-2661.97	-2566.11	1344.99

(9,1,4)	-520.07	-417.77	275.03	-2586.58	-2484.29	1308.29	-3066.25	-2963.96	1548.12	-3375.17	-3272.41	1702.58	-1745.88	-1643.59	887.94	-1886.93	-1784.64	958.47	-2478.93	-2376.23	1254.46	-2658.57	-2555.86	1344.29
(9,1,5)	-522.88	-413.77	277.44	-2590.80	-2481.69	1311.40	-3062.33	-2953.22	1547.16	-3376.74	-3267.13	1704.37	-1742.46	-1633.36	887.23	-1881.13	-1772.02	956.57	-2478.50	-2368.95	1255.25	-2660.12	-2550.56	1346.06
(9,1,6)	-523.17	-407.24	278.58	-2587.19	-2471.26	1310.60	-3062.55	-2946.62	1548.27	-3373.66	-3257.20	1703.83	-1739.89	-1623.97	886.94	-1880.98	-1765.05	957.49	-2478.61	-2362.21	1256.30	-2657.88	-2541.47	1345.94
(9,1,7)	-521.57	-398.82	278.79	-2586.79	-2464.04	1311.40	-3060.75	-2938.00	1548.37	-3374.04	-3250.73	1705.02	-1738.40	-1615.66	887.20	-1879.91	-1757.16	957.96	-2473.38	-2350.13	1254.69	-2654.76	-2531.51	1345.38
(9,1,8)	-519.46	-389.89	278.73	-2581.67	-2452.11	1309.84	-3059.71	-2930.15	1548.86	-3374.79	-3244.63	1706.40	-1736.28	-1606.72	887.14	-1874.57	-1745.00	956.28	-2471.62	-2341.53	1254.81	-2651.79	-2521.69	1344.90
(9,1,9)	-473.08	-336.69	256.54	-2580.38	-2443.99	1310.19	-3061.03	-2924.64	1550.51	-3371.49	-3234.48	1705.75	-1737.38	-1601.00	888.69	-1881.66	-1745.27	960.83	-2470.28	-2333.34	1255.14	-2650.20	-2513.25	1345.10
(9,1,10)	-516.96	-373.75	279.48	-2581.69	-2438.49	1311.85	-3059.59	-2916.38	1550.79	-3371.43	-3227.57	1706.72	-1732.83	-1589.63	887.42	-1884.31	-1741.10	963.15	-2468.06	-2324.28	1255.03	-2648.38	-2504.58	1345.19
(9,2,0)	857.10	925.29	-418.55	-1165.62	-1097.43	592.81	-1721.26	-1653.07	870.63	-1999.81	-1931.30	1009.90	-259.36	-191.17	139.68	-438.26	-370.06	229.13	-893.04	-824.57	456.52	-1026.76	-958.29	523.38
(9,2,1)	-375.73	-300.72	198.87	-2453.75	-2378.74	1237.87	-2936.51	-2861.50	1479.26	-3232.06	-3156.70	1627.03	-1556.13	-1481.13	789.07	-1689.03	-1614.02	855.51	-2268.54	-2193.23	1145.27	-2399.47	-2324.14	1210.73
(9,2,2)	-359.02	-277.19	191.51	-2428.65	-2346.82	1226.32	-2915.21	-2833.38	1469.61	-3208.41	-3126.21	1616.21	-1520.89	-1439.06	772.44	-1665.35	-1583.52	844.68	-2248.33	-2166.16	1136.16	-2377.25	-2295.08	1200.63
(9,2,3)	-338.21	-249.56	182.11	-2382.80	-2294.15	1204.40	-2909.31	-2820.66	1467.66	-3191.34	-3102.29	1608.67	-1492.56	-1403.91	759.28	-1654.05	-1565.40	840.02	-2204.44	-2115.44	1115.22	-2361.91	-2272.89	1193.96
(9,2,4)	-276.71	-181.24	152.35	-2324.63	-2229.16	1176.32	-2862.71	-2767.24	1445.35	-3168.77	-3072.87	1598.39	-1431.82	-1336.36	729.91	-1597.23	-1501.76	812.61	-2148.94	-2053.08	1088.47	-2264.11	-2168.25	1146.06
(9,2,5)	-461.81	-359.52	245.91	-2295.17	-2192.88	1162.58	-2822.89	-2720.60	1426.45	-3049.66	-2946.91	1539.83	-1374.29	-1272.01	702.15	-1553.01	-1450.72	791.51	-2096.49	-1993.79	1063.24	-2232.00	-2129.29	1131.00
(9,2,6)	-510.51	-401.40	271.26	-2266.51	-2157.40	1149.25	-3039.90	-2930.80	1535.95	-3353.42	-3243.82	1692.71	-1283.83	-1174.73	657.92	-1468.80	-1359.69	750.40	-1993.76	-1884.21	1012.88	-2175.98	-2066.42	1103.99
(9,2,7)	-508.84	-392.91	271.42	-2142.60	-2026.68	1088.30	-3025.59	-2909.67	1529.80	-3332.85	-3216.39	1683.42	-1725.99	-1610.07	879.99	-1436.68	-1320.75	735.34	-2446.50	-2330.10	1240.25	-2569.13	-2452.72	1301.56
(9,2,8)	-492.91	-370.16	264.45	-2572.37	-2449.62	1304.18	-3023.87	-2901.12	1529.94	-3342.90	-3219.59	1689.45	-1727.50	-1604.76	881.75	-1351.36	-1228.61	693.68	-1852.57	-1729.33	944.28	-2574.20	-2450.95	1305.10
(9,2,9)	74.82	204.39	-18.41	-2504.12	-2374.56	1271.06	-3038.19	-2908.62	1538.09	-3337.75	-3207.59	1687.88	-1625.09	-1495.53	831.54	-1868.58	-1739.01	953.29	-1687.30	-1557.21	862.65	-2639.42	-2509.32	1338.71
(9,2,10)	-481.42	-345.03	260.71	-2554.43	-2418.04	1297.21	-2818.96	-2682.58	1429.48	-3315.99	-3178.98	1678.00	-1541.49	-1405.11	790.74	-1810.90	-1674.51	925.45	-2454.95	-2318.02	1247.48	-2636.85	-2499.90	1338.43
(10,1,0)	-414.08	-332.25	219.04	-2490.98	-2409.15	1257.49	-2969.50	-2887.67	1496.75	-3276.49	-3194.28	1650.24	-1585.50	-1503.67	804.75	-1716.12	-1634.29	870.06	-2323.76	-2241.60	1173.88	-2444.32	-2362.15	1234.16
(10,1,1)	-527.98	-439.33	276.99	-2593.52	-2504.87	1309.76	-3070.86	-2982.21	1548.43	-3382.09	-3293.03	1704.04	-1750.83	-1662.19	888.42	-1888.72	-1800.07	957.36	-2481.27	-2392.26	1253.64	-2663.50	-2574.49	1344.75

(10,1,2)	-526.05	-430.57	277.02	-2593.21	-2497.73	1310.60	-3068.84	-2973.37	1548.42	-3381.65	-3285.74	1704.82	-1748.38	-1652.91	888.19	-1886.63	-1791.15	957.31	-2480.24	-2384.38	1254.12	-2662.30	-2566.44	1345.15
(10,1,3)	-525.67	-423.38	277.84	-2588.46	-2486.17	1309.23	-3068.24	-2965.95	1549.12	-3379.69	-3276.94	1704.85	-1746.57	-1644.28	888.28	-1886.45	-1784.15	958.22	-2480.44	-2377.74	1255.22	-2661.18	-2558.47	1345.59
(10,1,4)	-518.57	-409.46	275.29	-2589.27	-2480.16	1310.63	-3064.46	-2955.35	1548.23	-3378.99	-3269.38	1705.50	-1742.58	-1633.48	887.29	-1884.87	-1775.76	958.44	-2477.51	-2367.96	1254.76	-2658.94	-2549.38	1345.47
(10,1,5)	-521.42	-405.49	277.71	-2588.57	-2472.65	1311.29	-3063.39	-2947.46	1548.69	-3375.76	-3259.30	1704.88	-1740.93	-1625.01	887.46	-1880.92	-1764.99	957.46	-2476.63	-2360.23	1255.31	-2657.53	-2541.12	1345.76
(10,1,6)	-516.77	-394.02	276.39	-2582.96	-2460.21	1309.48	-3062.18	-2939.43	1549.09	-3371.72	-3248.41	1703.86	-1737.93	-1615.19	886.96	-1880.16	-1757.41	958.08	-2475.82	-2352.58	1255.91	-2655.47	-2532.22	1345.74
(10,1,7)	-520.33	-390.75	279.16	-2585.20	-2455.63	1311.60	-3058.75	-2929.18	1548.37	-3374.70	-3244.54	1706.35	-1736.79	-1607.23	887.39	-1885.34	-1755.77	961.67	-2472.04	-2341.95	1255.02	-2653.77	-2523.66	1345.88
(10,1,8)	-519.52	-383.13	279.76	-2585.73	-2449.34	1312.87	-3058.01	-2921.63	1549.01	-3370.52	-3233.51	1705.26	-1736.68	-1600.30	888.34	-1887.03	-1750.64	963.51	-2470.26	-2333.32	1255.13	-2650.41	-2513.46	1345.21
(10,1,9)	-522.04	-378.83	282.02	-2586.10	-2442.89	1314.05	-3060.74	-2917.53	1551.37	-3369.91	-3226.05	1705.96	-1736.60	-1593.40	889.30	-1874.63	-1731.42	958.31	-2469.23	-2325.44	1255.61	-2648.52	-2504.72	1345.26
(10,1,10)	-515.41	-365.38	279.71	-2579.29	-2429.27	1311.65	-3059.79	-2909.76	1551.90	-3368.90	-3218.19	1706.45	-1731.82	-1581.81	887.91	-1871.10	-1721.07	957.55	-2466.03	-2315.40	1255.01	-2645.61	-2494.96	1344.80
(10,2,0)	727.97	802.98	-352.98	-1322.15	-1247.13	672.07	-1833.88	-1758.87	927.94	-2121.12	-2045.77	1071.56	-403.59	-328.58	212.79	-526.87	-451.85	274.43	-1024.48	-949.16	523.24	-1137.88	-1062.56	579.94
(10,2,1)	-402.61	-320.78	213.31	-2479.21	-2397.38	1251.60	-2957.76	-2875.93	1490.88	-3264.79	-3182.59	1644.40	-1573.78	-1491.96	798.89	-1704.42	-1622.58	864.21	-2311.82	-2229.66	1167.91	-2432.34	-2350.18	1228.17
(10,2,2)	-371.83	-283.17	198.91	-2457.95	-2369.30	1241.98	-2933.28	-2844.63	1479.64	-3229.95	-3140.89	1627.97	-1552.40	-1463.75	789.20	-1685.02	-1596.37	855.51	-2264.61	-2175.60	1145.31	-2395.47	-2306.46	1210.74
(10,2,3)	-355.74	-260.26	191.87	-2438.80	-2343.34	1233.40	-2913.70	-2818.23	1470.85	-3205.16	-3109.26	1616.58	-1523.57	-1428.11	775.79	-1677.28	-1581.81	852.64	-2243.08	-2147.22	1135.54	-2375.94	-2280.07	1201.97
(10,2,4)	-307.83	-205.54	168.92	-2386.21	-2283.93	1208.11	-2895.83	-2793.54	1462.92	-3186.46	-3083.70	1608.23	-1490.10	-1387.82	760.05	-1636.99	-1534.70	833.49	-2194.09	-2091.39	1112.04	-2326.44	-2223.73	1178.22
(10,2,5)	-280.40	-171.29	156.20	-2379.67	-2270.56	1205.83	-2861.31	-2752.20	1446.66	-3173.84	-3064.24	1602.92	-1424.02	-1314.92	728.01	-1582.69	-1473.58	807.35	-2142.35	-2032.80	1087.18	-2266.08	-2156.53	1149.04
(10,2,6)	-507.88	-391.95	270.94	2557.49	2673.42	-1261.75	-3054.47	-2938.55	1544.24	-3358.72	-3242.26	1696.36	-1726.18	-1610.26	880.09	-1637.73	-1521.80	835.87	-2464.98	-2348.59	1249.49	-2231.18	-2114.78	1132.59
(10,2,7)	-507.22	-384.47	271.61	-2248.05	-2125.30	1142.02	-2920.75	-2798.00	1478.37	-3072.04	-2948.73	1554.02	-1725.07	-1602.33	880.54	-1472.52	-1349.77	754.26	-2402.85	-2279.61	1219.42	-2372.63	-2249.38	1204.32
(10,2,8)	-135.54	-5.97	86.77	-2569.46	-2439.90	1303.73	-3043.23	-2913.66	1540.61	-3347.79	-3217.64	1692.90	-1724.01	-1594.45	881.00	-1864.15	-1734.58	951.08	-2459.18	-2329.10	1248.59	-2636.77	-2506.67	1337.39
(10,2,9)	-506.12	-369.73	273.06	-2572.25	-2435.87	1306.13	-3028.94	-2892.56	1534.47	-3290.01	-3153.00	1665.00	-1327.44	-1191.07	683.72	-1866.81	-1730.42	953.41	-2457.36	-2320.42	1248.68	-2637.78	-2500.83	1338.89
(10,2,10)	-487.75	-344.55	264.88	-2565.65	-2422.44	1303.82	-3040.50	-2897.30	1541.25	-3343.91	-3200.05	1692.96	-1715.49	-1572.29	878.74	-1833.06	-1689.86	937.53	-2454.95	-2311.16	1248.47	-2361.85	-2218.06	1201.93

Table S5 - Median computational time of each (*p*,*d*,*q*) parameter combination across all patients for all physiologic signals at 1-min resolution

ARIMA Model	Median Computation Time (s)	ARIMA Model	Median Computation Time (s)	ARIMA Model	Median Computation Time (s)	ARIMA Model	Median Computation Time (s)	ARIMA Model	Median Computation Time (s)
(1,1,0)	1.322	(3,1,0)	3.389	(5,1,0)	5.326	(7,1,0)	6.855	(9,1,0)	10.691
(1,1,1)	5.994	(3,1,1)	13.414	(5,1,1)	22.478	(7,1,1)	29.005	(9,1,1)	42.046
(1,1,2)	11.437	(3,1,2)	17.768	(5,1,2)	26.923	(7,1,2)	33.420	(9,1,2)	47.688
(1,1,3)	14.488	(3,1,3)	24.209	(5,1,3)	32.641	(7,1,3)	39.323	(9,1,3)	54.477
(1,1,4)	19.774	(3,1,4)	30.200	(5,1,4)	36.445	(7,1,4)	43.405	(9,1,4)	59.907
(1,1,5)	21.018	(3,1,5)	30.700	(5,1,5)	37.038	(7,1,5)	47.145	(9,1,5)	65.114
(1,1,6)	24.765	(3,1,6)	37.227	(5,1,6)	44.271	(7,1,6)	50.238	(9,1,6)	69.669
(1,1,7)	25.138	(3,1,7)	38.715	(5,1,7)	45.424	(7,1,7)	51.698	(9,1,7)	73.188
(1,1,8)	33.715	(3,1,8)	51.777	(5,1,8)	60.744	(7,1,8)	68.372	(9,1,8)	77.186
(1,1,9)	36.739	(3,1,9)	56.587	(5,1,9)	66.771	(7,1,9)	75.335	(9,1,9)	84.928
(1,1,10)	44.856	(3,1,10)	68.543	(5,1,10)	82.063	(7,1,10)	92.059	(9,1,10)	103.211
(1,2,0)	0.663	(3,2,0)	1.916	(5,2,0)	2.863	(7,2,0)	4.231	(9,2,0)	6.202
(1,2,1)	6.998	(3,2,1)	13.984	(5,2,1)	19.536	(7,2,1)	26.800	(9,2,1)	37.992
(1,2,2)	10.830	(3,2,2)	16.521	(5,2,2)	21.499	(7,2,2)	28.510	(9,2,2)	42.399
(1,2,3)	14.447	(3,2,3)	22.908	(5,2,3)	26.404	(7,2,3)	34.249	(9,2,3)	48.706
(1,2,4)	17.408	(3,2,4)	25.049	(5,2,4)	29.896	(7,2,4)	37.979	(9,2,4)	52.137
(1,2,5)	20.756	(3,2,5)	30.030	(5,2,5)	34.849	(7,2,5)	40.125	(9,2,5)	56.442
(1,2,6)	23.846	(3,2,6)	32.292	(5,2,6)	37.245	(7,2,6)	43.112	(9,2,6)	59.974
(1,2,7)	30.427	(3,2,7)	39.620	(5,2,7)	45.914	(7,2,7)	52.519	(9,2,7)	62.564
(1,2,8)	35.223	(3,2,8)	44.808	(5,2,8)	50.446	(7,2,8)	57.514	(9,2,8)	65.401
(1,2,9)	44.082	(3,2,9)	55.400	(5,2,9)	61.465	(7,2,9)	69.378	(9,2,9)	78.716
(1,2,10)	48.353	(3,2,10)	59.564	(5,2,10)	65.445	(7,2,10)	72.790	(9,2,10)	82.512
(2,1,0)	2.782	(4,1,0)	4.330	(6,1,0)	5.704	(8,1,0)	7.553	(10,1,0)	12.861
(2,1,1)	9.944	(4,1,1)	17.817	(6,1,1)	23.961	(8,1,1)	30.933	(10,1,1)	48.409
(2,1,2)	16.096	(4,1,2)	21.953	(6,1,2)	28.238	(8,1,2)	34.802	(10,1,10)	108.613
(2,1,3)	19.313	(4,1,3)	27.275	(6,1,3)	33.678	(8,1,3)	41.196	(10,1,2)	54.210
(2,1,4)	24.366	(4,1,4)	34.185	(6,1,4)	36.741	(8,1,4)	44.789	(10,1,3)	61.477
(2,1,5)	24.948	(4,1,5)	33.889	(6,1,5)	39.875	(8,1,5)	48.614	(10,1,4)	68.483
(2,1,6)	30.402	(4,1,6)	40.522	(6,1,6)	47.474	(8,1,6)	52.212	(10,1,5)	72.268
(2,1,7)	31.838	(4,1,7)	41.998	(6,1,7)	48.261	(8,1,7)	55.043	(10,1,6)	77.366
(2,1,8)	42.480	(4,1,8)	55.950	(6,1,8)	64.395	(8,1,8)	72.865	(10,1,7)	82.697
(2,1,9)	46.524	(4,1,9)	61.797	(6,1,9)	71.527	(8,1,9)	79.979	(10,1,8)	86.133
(2,1,10)	57.388	(4,1,10)	75.116	(6,1,10)	87.790	(8,1,10)	97.516	(10,1,9)	89.999
(2,2,0)	1.367	(4,2,0)	2.329	(6,2,0)	3.634	(8,2,0)	5.335	(10,2,0)	7.819
(2,2,1)	10.034	(4,2,1)	17.632	(6,2,1)	24.141	(8,2,1)	33.486	(10,2,1)	47.406
(2,2,2)	13.665	(4,2,2)	19.815	(6,2,2)	25.725	(8,2,2)	36.653	(10,2,2)	52.127
(2,2,3)	19.526	(4,2,3)	25.442	(6,2,3)	31.236	(8,2,3)	43.015	(10,2,3)	103.015
(2,2,4)	22.056	(4,2,4)	27.673	(6,2,4)	34.867	(8,2,4)	47.810	(10,2,4)	64.708
(2,2,5)	26.860	(4,2,5)	32.095	(6,2,5)	37.320	(8,2,5)	51.065	(10,2,5)	68.641
(2,2,6)	29.178	(4,2,6)	35.293	(6,2,6)	40.304	(8,2,6)	53.738	(10,2,6)	75.207
(2,2,7)	36.446	(4,2,7)	42.953	(6,2,7)	49.244	(8,2,7)	56.604	(10,2,7)	77.719
(2,2,8)	42.051	(4,2,8)	47.495	(6,2,8)	53.881	(8,2,8)	62.181	(10,2,8)	81.867
(2,2,9)	51.932	(4,2,9)	57.893	(6,2,9)	64.779	(8,2,9)	74.198	(10,2,9)	85.539
(2,2,10)	55.802	(4,2,10)	62.600	(6,2,10)	69.303	(8,2,10)	77.774	(10,2,10)	89.131

Table S6 - Percentage comparison of stationarity and non-stationarity based on ADF test on the original data

Temporal Resolution		1-min	5-min	10-min	30-min	1-hour	2-hour	3-hour	4-hour	5-hour	6-hour	12-hour	1-day
MAP	Stationarity	94.2	83	75.4	63.7	56.9	44.9	41.8	35.9	34.7	30.8	22.3	23.1
	Non-stationary	5.6	16.8	24.4	36	42.8	54.8	57.9	63.8	65	68.8	77.3	76.4
	NA	0.3	0.3	0.3	0.3	0.3	0.3	0.3	0.3	0.3	0.3	0.4	0.5
ICP	Stationarity	84.1	74.3	70.3	57.5	53.2	43.7	40.3	36.2	32.2	34.3	29.1	28.7
	Non-stationary	15.9	25.7	29.7	42.5	46.8	56.3	59.7	63.8	67.8	65.7	70.9	71.3
	NA	0	0	0	0	0	0	0	0	0	0	0	0
CPP	Stationarity	91.4	83.2	76.2	64.6	56.9	48.4	41.2	37.4	32.5	33	27.7	28.7
	Non-stationary	8.4	16.5	23.5	35.1	42.8	51.3	58.5	62.3	67.2	66.7	72	70.8
	NA	0.3	0.3	0.3	0.3	0.3	0.3	0.3	0.3	0.3	0.3	0.4	0.5
PRx	Stationarity	98.9	89.4	80.4	62.6	57.5	47.8	40	33.8	36.5	28.7	24.8	25.9
	Non-stationary	0.8	10.3	19.3	37.1	42.2	51.9	59.7	65.9	63.2	71	74.8	73.6
	NA	0.3	0.3	0.3	0.3	0.3	0.3	0.3	0.3	0.3	0.3	0.4	0.5
PAx	Stationarity	98.1	91.9	82.9	68.8	62.1	52.2	40.6	36.5	33.1	30.5	30.1	23.1
	Non-stationary	1.7	7.8	16.8	30.9	37.6	47.5	59.1	63.2	66.6	69.2	69.5	76.4
	NA	0.3	0.3	0.3	0.3	0.3	0.3	0.3	0.3	0.3	0.3	0.4	0.5
RAC	Stationarity	99.2	89.7	83.2	66.9	55.8	47.2	39.1	36.5	35.9	33.6	30.5	29.6
	Non-stationary	0.6	10.1	16.5	32.9	43.9	52.5	60.6	63.2	63.8	66	69.1	69.9
	NA	0.3	0.3	0.3	0.3	0.3	0.3	0.3	0.3	0.3	0.3	0.4	0.5
RAP	Stationarity	97.8	89.9	81	67.1	58.7	49.9	46.8	41.6	38.6	36.8	31.9	29.2
	Non-stationary	2.2	10.1	19	32.9	41.3	50.1	53.2	58.4	61.4	63.2	68.1	70.8
	NA	0	0	0	0	0	0	0	0	0	0	0	0
COx_L	Stationarity	36.2	34.9	32.5	28.9	24.9	21.3	20	15.6	14.3	13.4	8.2	8.3
	Non-stationary	0.8	2.2	4.5	7.4	10.7	14.3	15.3	19.8	21.3	21.5	23.4	21.8
	NA	63	62.8	63	63.7	64.5	64.4	64.7	64.7	64.4	65.1	68.4	69.9
COx_R	Stationarity	36.2	34.9	33.6	26.6	22.8	19	17.9	16.2	13.4	13.1	8.9	8.8
	Non-stationary	0.8	2.2	3.4	9.6	12.7	16.3	17.1	18.9	21.9	21.5	23	21.3
	NA	63	62.8	63	63.7	64.5	64.7	65	65	64.7	65.4	68.1	69.9
COx-a_L	Stationarity	35.9	35.5	33.1	27.5	23.1	19.8	14.1	12.9	12.8	13.1	6	7.9
	Non-stationary	0	0.6	2.8	7.6	11.3	14.6	20	21.3	21.6	20.6	24.5	21.8
	NA	64.1	64	64.1	64.9	65.6	65.6	65.9	65.9	65.7	66.4	69.5	70.4
COx-a_R	Stationarity	35.7	35.2	34.5	26.6	23.1	19	17.4	15.9	11.2	11.2	9.2	7.4
	Non-stationary	0.3	0.8	1.4	8.5	11.3	15.2	16.5	18	22.8	22.1	21.6	22.2
	NA	64.1	64	64.1	64.9	65.6	65.9	66.2	66.2	66	66.7	69.1	70.4
rSO_2_L	Stationarity	25.1	20.9	17.9	17.3	15.3	13.7	11.5	11.4	8.2	10.6	10.3	9.7
	Non-stationary	12.3	16.5	19.3	19.3	20.5	21.9	23.8	24	27.4	24.3	21.3	20.4
	NA	62.7	62.6	62.7	63.5	64.2	64.4	64.7	64.7	64.4	65.1	68.4	69.9
rSO_2_R	Stationarity	25.1	21.2	19.3	15.3	13.9	11.4	10.6	10.8	9.1	11.5	8.5	8.8
	Non-stationary	12	15.9	17.6	21	21.7	23.9	24.4	24.3	26.1	23.1	23.4	21.3
	NA	63	62.8	63	63.7	64.5	64.7	65	65	64.7	65.4	68.1	69.9
$PbtO_2$	Stationarity	23.1	20.4	19	17	15.9	17.2	13.5	15.3	10.6	10.6	9.9	10.2
	Non-stationary	6.7	9.2	10.6	12.5	13.3	12.2	15.9	13.2	17.6	18.1	19.1	21.3
	NA	70.2	70.4	70.3	70.5	70.8	70.6	70.6	71.6	71.7	71.3	70.9	68.5

Table S7 - Percentage comparison of stationarity and non-stationarity based on ADF test on the first-order differenced data

Temporal Resolution		1-min	5-min	10-min	30-min	1-hour	2-hour	3-hour	4-hour	5-hour	6-hour	12-hour	1-day
	Stationarity	99.7	99.7	98.4	96.0	88.8	81.6	72.6	64.1	59.0	56.9	41.8	24.2
	Non-stationary	0.0	0.0	1.1	3.5	9.6	15.7	23.7	30.6	33.5	33.8	37.5	32.2
MAP	NA	0.3	0.3	0.5	0.5	1.6	2.7	3.7	5.3	7.4	9.3	20.7	43.6
	Stationarity	99.5	98.1	98.1	94.9	88.8	78.2	71.8	64.1	61.4	51.6	38.0	23.1
	Non-stationary	0.3	1.6	1.3	4.3	8.8	18.6	23.9	29.5	30.6	38.0	39.4	31.6
ICP	NA	0.3	0.3	0.5	0.8	2.4	3.2	4.3	6.4	8.0	10.4	22.6	45.2
	Stationarity	99.5	99.2	98.4	93.1	88.3	80.6	69.1	62.2	56.9	57.2	37.8	22.6
	Non-stationary	0.0	0.3	0.8	5.9	8.8	15.2	25.5	30.3	34.0	31.1	38.8	31.6
CPP	NA	0.5	0.5	0.8	1.1	2.9	4.3	5.3	7.4	9.0	11.7	23.4	45.7
	Stationarity	99.2	98.4	97.6	95.5	90.4	79.3	69.4	64.1	59.8	52.1	36.7	20.2
	Non-stationary	0.3	1.1	1.6	3.5	6.6	16.5	25.3	28.2	31.1	36.4	39.9	33.5
PRx	NA	0.5	0.5	0.8	1.1	2.9	4.3	5.3	7.7	9.0	11.4	23.4	46.3
	Stationarity	99.2	98.7	98.4	95.7	91.0	79.5	77.4	65.7	60.1	53.7	40.4	22.3
	Non-stationary	0.3	0.8	0.8	3.2	6.1	16.2	17.3	26.6	30.9	34.8	36.2	31.4
PAx	NA	0.5	0.5	0.8	1.1	2.9	4.3	5.3	7.7	9.0	11.4	23.4	46.3
	Stationarity	99.5	98.9	98.9	93.4	89.9	79.0	72.3	65.4	58.8	55.6	38.6	21.3
	Non-stationary	0.0	0.5	0.3	5.6	7.2	16.8	22.3	26.9	32.2	33.0	38.0	32.4
RAC	NA	0.5	0.5	0.8	1.1	2.9	4.3	5.3	7.7	9.0	11.4	23.4	46.3
	Stationarity	99.7	98.7	98.7	96.3	89.4	79.8	73.7	62.0	56.9	55.3	39.4	21.5
	Non-stationary	0.0	1.1	0.8	2.9	8.2	17.0	22.1	31.6	34.8	34.3	38.0	33.0
RAP	NA	0.3	0.3	0.5	0.8	2.4	3.2	4.3	6.4	8.2	10.4	22.6	45.5
	Stationarity	30.1	29.5	28.5	27.4	25.0	22.6	21.0	18.9	17.8	14.6	11.7	9.8
	Non-stationary	0.5	0.8	1.3	2.1	4.0	5.9	6.9	8.5	8.5	11.2	11.4	8.2
COx_L	NA	69.4	69.7	70.2	70.5	71.0	71.5	72.1	72.6	73.7	74.2	76.9	81.9
	Stationarity	36.2	35.4	34.8	33.8	30.1	28.2	23.1	20.5	21.0	19.4	10.6	5.6
	Non-stationary	0.3	0.8	0.8	1.6	4.3	5.6	10.6	12.2	10.6	10.9	13.6	9.3
COx_R	NA	63.6	63.8	64.4	64.6	65.7	66.2	66.2	67.3	68.4	69.7	75.8	85.1
	Stationarity	36.2	35.6	35.1	34.3	31.4	26.9	27.9	22.3	18.9	20.7	12.5	7.2
	Non-stationary	0.0	0.3	0.5	1.1	2.9	6.6	5.3	10.1	12.5	9.0	12.8	9.3
COx-a_L	NA	63.8	64.1	64.4	64.6	65.7	66.5	66.8	67.6	68.6	70.2	74.7	83.5
	Stationarity	35.1	35.4	35.1	32.7	30.9	27.9	22.6	21.8	19.4	19.9	10.6	6.1
	Non-stationary	0.5	0.3	0.0	2.4	3.7	6.1	11.4	11.4	12.5	10.6	13.8	8.8
COx-a_R	NA	64.4	64.4	64.9	64.9	65.4	66.0	66.0	66.8	68.1	69.4	75.5	85.1
	Stationarity	35.4	35.1	35.1	33.8	30.3	26.6	24.2	20.7	17.6	18.6	13.6	6.4
	Non-stationary	0.0	0.3	0.0	1.3	4.3	6.9	9.0	11.7	13.8	11.2	11.7	10.4
rSO_2_L	NA	64.6	64.6	64.9	64.9	65.4	66.5	66.8	67.6	68.6	70.2	74.7	83.2
	Stationarity	36.7	36.4	35.6	33.2	31.4	27.1	24.5	22.3	21.0	19.9	15.4	8.5
	Non-stationary	0.3	0.3	0.8	3.2	4.8	8.5	11.2	12.8	12.2	12.5	10.4	7.4
rSO_2_R	NA	63.0	63.3	63.6	63.6	63.8	64.4	64.4	64.9	66.8	67.6	74.2	84.0
	Stationarity	36.4	35.9	34.6	33.8	30.6	29.0	25.3	22.6	18.6	19.4	12.8	5.6
	Non-stationary	0.0	0.5	1.6	2.4	5.1	5.6	9.0	11.2	13.8	12.5	14.1	12.0
$PbtO_2$	NA	63.6	63.6	63.8	63.8	64.4	65.4	65.7	66.2	67.6	68.1	73.1	82.4
